# Biomolecular Interaction, Anti-Cancer and Anti-Angiogenic Properties of Cobalt(III) Schiff Base Complexes

**DOI:** 10.1038/s41598-019-39179-1

**Published:** 2019-02-25

**Authors:** Subramanian Ambika, Yesaiyan Manojkumar, Sankaralingam Arunachalam, Balakrishnan Gowdhami, Kishore Kumar Meenakshi Sundaram, Rajadurai Vijay Solomon, Ponnambalam Venuvanalingam, Mohammad Abdulkader Akbarsha, Muthuraman Sundararaman

**Affiliations:** 1 Department of Chemistry, Bharathidasan University, Tiruchirappalli, 620 024 India; 2 Mahatma Gandhi-Doerenkamp Centre, Bharathidasan University, Tiruchirappalli, 620 024 India; 3 Centre for Environmental Research and Development (CERD), Loyola Institute of Frontier Energy (LIFE), Loyola College, Chennai, 600 034 India; 4 Department of Chemistry, Madras Christian College (Autonomous), East Tambaram, Chennai, 600 059 India; 5 Department of Marine Biotechnology, Bharathidasan University, Tiruchirappalli, 620 024 India; 6 Present Address: Department of Chemistry, Gebze Technical University, Gebze, 41400 Kocaeli Turkey; 7 Present Address: Plot-46, Nagappa Nagar, Airport (Post), Tiruchirappalli, 620007 India; 8 Present Address: National Center for Alternatives to Animal Experiments, Bharathidasan University, Tiruchirappalli, 620 024 India; 9 Present Address: Research Coordinator, National College (Autonomous), Tiruchirappalli, 620001 India

## Abstract

Two cobalt(III) Schiff base complexes, trans-[Co(salen)(DA)_2_](ClO_4_) (**1**) and trans-[Co(salophen)(DA)_2_](ClO_4_) (**2**) (where salen: N,N’-bis(salicylidene)ethylenediamine, salopen: N,N’-bis(salicylidene)-1,2-phenylenediamine, DA: dodecylamine) were synthesised and characterised using various spectroscopic and analytical techniques. The binding affinity of both the complexes with CT-DNA was explored adopting UV-visible, fluorescence, circular dichroism spectroscopy and cyclic voltammetry techniques. The results revealed that both the complexes interacted with DNA via intercalation as well as notable groove binding. Protein (BSA) binding ability of these complexes was investigated by absorption and emission spectroscopy which indicate that these complexes engage in strong hydrophobic interaction with BSA. The mode of interaction between these complexes and CT-DNA/BSA was studied by molecular docking analysis. The *in vitro* cytotoxic property of the complexes was evaluated in A549 (human small cell lung carcinoma) and VERO (African green monkey kidney cells). The results revealed that the complexes affect viability of the cells. AO and EB staining and cell cycle analysis revealed that the mode of cell death is apoptosis. Both the complexes showed profound inhibition of angiogenesis as revealed in *in-vivo* chicken chorioallantoic membrane (CAM) assay. Of the two complexes, the complex **2** proved to be much more efficient in affecting the viability of lung cancer cells than complex **1**. These results indicate that the cobalt(III) Schiff base complexes in this study can be potentially used for cancer chemotherapy and as inhibitor of angiogenesis, in general, and lung cancer in particular, for which there is need for substantiation at the level of signalling mechanisms and gene expressions.

## Introduction

Metal-based therapeutics have become a viable area of research in medicinal chemistry after the serendipitous discovery of *cis*-platin. At present nearly half the number of cancer patients are treated with platinum-based drugs^1,2^. However, platinum-based drugs are associated with (i) adverse side effects, (ii) lack of selectivity and (iii) intrinsic or acquired resistance, which prompted search for effective non-platinum drugs. Over the years, complexes of Ru, Ir, Cu, Ni, Zn, Co, etc., have been reported to posses much better anticancer property than *cis*-platin^3–6^. Cobalt is an essential trace element present in the human body. It is involved in important biological functions such as fatty acid and amino acid metabolism, haematopoiesis, and, in the form of vitamin B_12_ it is indirectly involved in synthesis of DNA. Interestingly, one cobalt complex containing Schiff base ligand (Doxovir) has recently passed phase II clinical trial for anti-viral treatment^7^. Several *in vitro* studies suggest that cobalt complexes possess promising anti-cancer activity^8^. Especially, cobalt complexes containing Schiff base ligands have been shown to possess more efficient anti-cancer activity against cancer cells such as MCF-7, A431 and HeLa than cis-platin^9–11^. Metal complexes containing tetradentate Schiff bases, salen and salophen, show a broad spectrum of biological activities, particularly in the context of cancer^12–15^. These Schiff bases provide platform to tune their anti-cancer activity *via* substitution of various moieties in their salicylaldehyde aromatic ring. For example, Tshuva *et al*. reported that salan titanium(IV) complexes bearing two differently substituted aromatic rings show 30-fold anti-cancer activity compared to cis-platin against colon HT-29 and OVCAR-1 cancer cells^9^. Some fluorine-substituted iron(III) salophen complexes are found to be highly cytotoxic to HT-29, MCF-7 and MDA-MB-231 cells^16^. Some methoxy group-substituted salophen containing iron(III) complexes are very active against cis-platin-resistant cancer cell lines^17^. Gust *et al*., studied the structure-activity relationship of cobalt salen complexes with regard to anti-proliferation property^18^. The results showed that the cytotoxic activity of these salen complexes is independent of the position of substitution rather than nature of the substituent in the aromatic rings present on the ethylenediamine bridge. Here, the methoxy substituent was equipotent to *cis*-platin, but hydroxy-substituted complexes did not show significant cytotoxic activity in cancer cells. This class of compounds kills cancer cells via oxidative DNA damage. In the case of cobalt(III) octahedral complexes, rather the salen and salophen Schiff bases occupying the square planar positions than the remaining two axial positions can be utilized to tune their properties for bio-molecule interaction and biological applications. Previously, we have reported that changing the number and length of aliphatic chains in the coordinated ligands of some cobalt(III) complexes would strongly influence the mode of biomolecule interactions and anticancer activity^19,20^. Lippard *et al*. have reported that the ligands directly alter the binding and cytotoxic properties of metal complexes^21^.

Angiogenesis (new blood vessel formation) plays an important role in tumour development. In general, tumour cannot grow beyond 2 to 3 mm without angiogenesis^22^. Essential nutrients and oxygen are supplied to the tumour microenvironment via the newly formed blood vessels. Many a times the metastatic cells use these blood vessels for migration from one place to another in the body. Hence, blocking angiogenesis is one of the promising strategies to inhibit cancer cell growth and metastasis. In the recent years, complexes of metals such as Ru, Cu, Pt and Ir have been reported to possess anti-angiogenesis property^23–26^. Even cobalt plays a notable role in the progression of angiogenesis^27^. To the best of our knowledge, the anti-angiogenic properties of cobalt Schiff base complexes have not been investigated yet. Besides, in the previous reports there is no combined experimental and theoretical evidence for the interaction of salen and salophen Schiff base complexes with DNA and serum proteins. The above background information encouraged us to synthesize long alkyl chain-containing cobalt(III) salen/salophen Schiff base complexes and study their bio-molecular interaction adopting both experimental and theoretical approaches. The anti-cancer and anti-angiogenic potentials of the complexes have also been investigated.

## Results and Discussion

### Synthesis and characterizations

**Figure 1:**
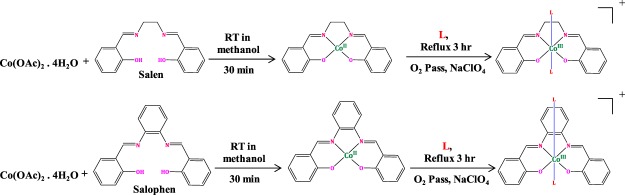
Scheme of synthesis of complexes **1** and **2**.

The Schiff base cobalt(III) complexes, [trans-[Co(salen)(DA)_2_]ClO_4_**(1)** and trans-[Co(salophen)(DA)_2_]ClO_4_**(2)** (DA – dodecylamine)] were synthesized as per the following scheme (Fig. 1), where L is dodecylamine.

The IR spectra of complexes **1** and **2** are given in Fig. S1. The IR spectrum of the salen ligand shows a band around 1636 cm^−1^ for the free γ(C=N) group. This band is shifted to lower wave number 1605 cm^−1^ in complex **1**, indicating that the two nitrogen atoms of salen ligand are coordinated to the cobalt metal centre^28^. The band observed around 1282 cm^−1^ in the IR region is attributed to the ring stretching frequencies of phenolic γ(C-O) of the free salen ligand which is shifted to the higher frequency region 1312 cm^−1^ for complex **2**. It proves that the other coordination site is phenolic oxygen atom^29^. The complex **2** shows the bands around 1610 cm^−1^ and 1328 cm^−1^ in the IR region which can be attributed to the ring stretching frequencies [γ(C=N) and phenolic γ(C-O)] of the salophen ligand whose values at the free state are 1634 cm^−1^ and 1275 cm^−1^, respectively. The shifts are due to the coordination of nitrogen atoms and phenolic oxygen of salophen ligand to the central cobalt(III). In the complexes **1** and **2**, the carbon-hydrogen asymmetrical and symmetrical stretching vibrations of CH_2_ of long chain aliphatic amine appeared around 2852–2860 cm^−1^ and 2922–2930 cm^−1^. Another band around 3420–3435 cm^−1^ can be attributed to N-H for alkylamine. A previous report shows that non-coordinated perchlorate ion peak comes around 1086–1089 cm^−1^. Both our complexes show their respective peaks around this region, which clearly indicate the presence of perchlorate as a counter ion^30^.

The ^1^H NMR and ^13^C NMR spectra of the complexes **1** and **2** are given in Fig. S2 and S3. In the ^1^H NMR spectra, the - CH_2_ protons of the aliphatic amine appeared as mutiplet in the range of 0.8–0.9 ppm. The two CH_2_ protons appeared at 2.25 and 1.8 ppm, respectively. Both the complexes showed triplet at around 0.8 ppm that corresponds to the terminal methyl group of the long chain aliphatic amine. The ethylene diamine protons of complex **1** appeared at 4.05 ppm. The peaks at 7.9 and 8.6 ppm are assigned to two imine protons of the complexes **1** and **2**, respectively. The aromatic protons of the complex **1** and **2** appeared in the region 6.5 to 8.6 ppm. The ^13^C NMR spectra of both the complexes gave signals around 38–40 ppm because of merging of signals of long chain aliphatic amine. The peak at 58 ppm indicates the ethylenediamine carbon of complex **1**. The imine carbon of both the complexes appeared around 166 ppm. The peaks around 110 to 140 are assigned as aromatic carbon of the complexes.

The electrospray ionization (ESI) mass spectra of the complexes in methanol were recorded (Fig. S4). The ESI-MS spectra of cobalt complexes showed good intensity peak related to the molecular ion [Co(salen)(DA)_2_]^+^, [Co(salophen)(DA)_2_]^+^ and the fragment ion [Co(salen)]^+^, [Co(salen)(DA)]^+^ for complex **1** and [Co(salophen)]^+^, [Co(salopen)(DA)]^+^ for complex **2**, respectively.

The absorption spectra of both the complexes in DMSO were recorded at room temperature. These spectra show bands at 250–263 nm due to π-π* transition related to aromatic ring, and 368–388 nm, which correspond to n-π* transitions. Besides, both the complexes showed a shoulder peak around 470–490 nm due to ligand to metal charge transfer (LMCT) transition.

### DNA binding studies

#### Absorption spectral studies

**Figure 2:**
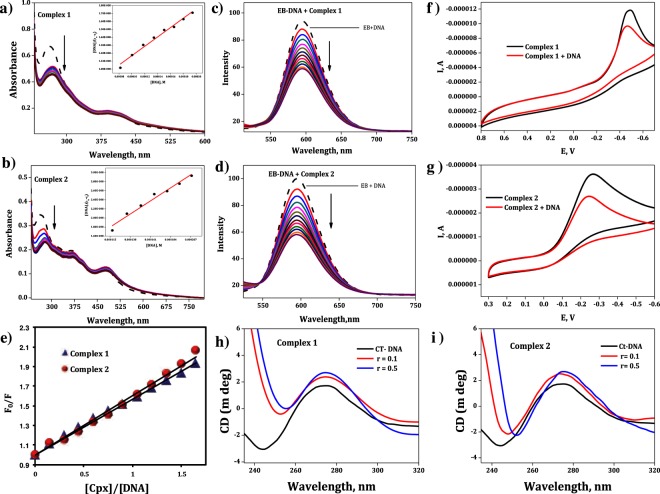
Electronic absorption spectra of cobalt(III) complexes **1 (a)** and **2 (b)** in the absence (dashed line) and presence (solid line) of increasing amounts of DNA. Insert: Plot of [DNA]/(ε_a_ − ε_f_) vs [DNA]. [Complex] = 2 × 10^−5^ M^−1^, [DNA] = 0–2.1 × 10^−4^ M^−1^. Emission spectra (λ_ex_ = 450 nm) of EB - DNA: in the absence (dotted line) and in the presence of (solid line) of the complexes **1 (c)** and **2 (d)**. Stern-Volmer (CV) plot **(e)** of fluorescence quenching of EB - DNA by complexes **1** and **2**. Cyclic Voltammetry of complexes **1 (f)** and **2 (g)** in the absence ( black line) and in the presence (red line) of DNA. Circular dichroism spectra of CT-DNA (4 × 10^−5^ M) in the presence of complex **1 (h)** and **2 (i)**, r = [complex]/[DNA] = 0.0, 0.1 and 0.5).

The absorption spectra of the complexes **1** and **2** were measured in presence and absence of CT-DNA, and the results are shown in Fig. 2. Incremental additions of DNA to the solution of complexes **1** and **2** resulted in hypochromism, from which it is inferred that the complexes bind to DNA through intercalation^31^. The percentage of hypochromism (H %) observed for the complexes **1** and **2** are 31.54 and 36.81% with red shift of 10 and 13 nm, respectively. The magnitude of observed hypochromism and red shift for complex **2** is higher compared to that for complex **1**.

The value of DNA binding constant (K_b_) of the complexes **1** and **2** were calculated using the equation^32^,

$$[{\rm{DNA}}]/{({\rm{\varepsilon }}}_{{\rm{a}}}-{{\rm{\varepsilon }}}_{{\rm{f}}})=[{\rm{DNA}}]/({{\rm{\varepsilon }}}_{0}-{{\rm{\varepsilon }}}_{{\rm{f}}})+1/{{\rm{K}}}_{{\rm{b}}}({{\rm{\varepsilon }}}_{0}-{{\rm{\varepsilon }}}_{{\rm{f}}})$$

where [DNA] is the concentration of DNA expressed in base pairs; ε_a_ is called as apparent extinction coefficient which is determined by calculating A_obs_/[complex], ε_f_ and ε_0_ are the apparent free and fully-bound cobalt(III) complex extinction coefficients.

The intrinsic binding constants K_b_ of complexes **1** and **2** were calculated from the plot of [DNA]/ (ε_a_-ε_f_) versus [DNA] the values are shown in Table 1. As seen from this table, the K_b_ values are lower than those observed for typical classical intercalator (EB = K_b_ = 4.94 × 10^5^ M^−1^)^33^. As both the complexes intercalated with DNA, it is expected that salicylaldehyde aromatic moiety is involved in the intercalation. From the results, it is inferred that complex **2** shows slightly higher K_b_ and percentage of hypochromism than complex **1**. This is due to the presence of additional aromatic phenyl ring in the salophen Schiff base ligand in complex **2** which enhanced stacking interaction between salicylaldehyde aromatic moiety and DNA bases. This results in an increased DNA binding propensity of complex **2**.

**Table 1:** The percentage of hypochromism, binding constant (K_b_) and Stern–Volmer constant (K_sv_) of the complexes **1** and **2** with CT-DNA.

Complexes	% H	K_b_ (M^−1^)	K_sv_
Complex **1**	30.34	1.31 × 10^4^	0.5694
Complex **2**	36.81	3.15 × 10^4^	0.6067

In addition to intercalation, the presence of long aliphatic chain (dodecylamine) in the complexes can make some notable hydrophobic interaction with DNA. This combined effect of strong intercalation and notable hydrophobic interaction is responsible for the better binding affinity of complex **2** with DNA than some other long chain aliphatic amine-containing complexes such as *cis-*[Co(bpy)_2_(C_11_H_23_NH_2_)Cl](ClO_4_)_2_ (K_b_ = 2.21 × 10^4^ M^−1^)^34^, *cis-*[Co(en)_2_(C_14_H_29_NH_2_)Cl](ClO_4_)_2_ (K_b_ = 2.59 × 10^4^ M^−1^)^35^, *cis*-[Co(bpy)_2_(C_14_H_29_NH_2_)Cl](ClO_4_)_2_ (K_b_ = 2.86 × 10^4^ M^−1^)^35^, [Co(dien)(DA)Cl_2_](ClO_4_) (K_b_ = 3.1108 × 10^3^ M^−1^)^20^, [Co(dien)(CA)Cl_2_](ClO_4_) (K_b_ = 9.0052 × 10^3^ M^−1^)^20^, and [Co(dien)(DA)_2_Cl](ClO_4_)_2_ (K_b_ = 1.4318 × 10^4^ M^−1^), reported in the literature^20^.

#### Emission studies

The complexes **1** and **2** are non-emissive in nature. Hence, competitive binding studies were performed using ethidium bromide (EB). In this study, changes in the intensity of the fluorescence emission band of EB - DNA complex were monitored after the addition of metal complexes **1** and **2** (Fig. 2). Upon increasing complex concentration in the DNA-EB solution, EB emission intensity was decreased. As the binding sites of the DNA available for EB decreased, some EB molecules were released into aqueous solution outside the DNA molecule, which resulted in decrease of fluorescence of EB^36,37^. The resulting data (Table 1) of the complexes **1** and **2** with EB-DNA was processed from the standard Stern-Volmer equation^38^,

$${{\rm{F}}}_{0}/{\rm{F}}=1+{{\rm{K}}}_{{\rm{sv}}}{\rm{r}}$$

where F_0_ and F are the fluorescence emission intensities in the absence and presence of quencher (*i.e*. complexes **1** and **2**), r is the ratio of concentration of the quencher. K_sv_ is the Stern-Volmer quenching constant. The quenching plots of F_0_/F versus [complex]/[DNA] are shown in Fig. 2, and the K_sv_ values are given in Table 1. As seen from this table, the K_sv_ value of both complexes **1** and **2** are almost same. The results indicate that both the complexes interact with DNA via intercalation.

#### Cyclic voltammetry studies

Electrochemical studies of complexes **1** and **2** were recorded in 0.5% DMSO/PBS buffer solution, with glassy carbon as working electrode vs saturated calomel as reference electrode and Pt wire as auxiliary electrode. Fig. 2 shows the cyclic voltammograms of both the complexes (black line) and in the presence of DNA (red line). The cyclic voltammetric data of complexes show one irreversible wave due to the reduction of Co(III) to the Co(II) form at a cathodic peak potential, E_pc_ −0.490 V, −0.265 V and cathodic peak current, i_pc_ −1.184 × 10^−5^ A, − 3.622 × 10^−6^ A for complex **1** and **2**. On addition of CT-DNA to each cobalt complex solution, no new peak appeared. Upon adding CT-DNA, the complexes **1** and **2** exhibit considerable decrease in the voltammetric current, (i_pc_) and the cathodic peak potentials (E_pc_) show positive shift. The changes of the peak potentials observed for the complexes **1** and **2** are presented in the Table 2. The decrease in voltammetric current in the presence of CT- DNA is possibly due to the diffusion of the complexes **1** and **2** bound to the large, slowly diffusing DNA molecule^39,40^. The positive shifts indicate that complexes **1** and **2** bind to DNA through intercalation as observed in the spectroscopic studies^39,40^.

**Table 2:** Redox potentials of cobalt(III) complexes **1** and **2** with DNA.

Complexes	E_pc_ (V)	i_pc_ (A)
Complex 1	−0.490	−1.184 × 10^−5^
Complex 1 + DNA	−0.467	−9.674 × 10^−6^
Complex 2	−0.265	−3.622 × 10^−6^
Complex 2 + DNA	−0.243	−2.695 × 10^−6^

#### Circular Dichroism

CD spectroscopy is a sensitive technique to detect conformational changes of DNA during drug - DNA interactions. Circular dichroism (CD) spectrum of CT-DNA showed characteristic peak at 274 nm due to peak positive Cotton effect (base stacking) and a negative Cotton effect peak (right-handed helicity) at 244 nm in the ultraviolet region. These two peaks correspond to the base stacking and the helicity of normal B-DNA conformation^41,42^. The observed modifications in the presence of the metal complexes i.e., change in the positive and negative band position/intensity, or even both, in the spectra of DNA can be correlated to the mode of interactions between the DNA and the metal complexes.

After the addition of cobalt complexes, perceptible changes occurred in the CD spectra of B-DNA (Fig. 2). The intensities of negative band decreased along with a bathochromic effect and the positive band increased with slight red shift in the wavelength. The interaction between these cobalt complexes and DNA made partial unwinding of the DNA helix which reflects the decreased intensity of the negative band, which is attributed to a strong conformational change in DNA helix. From the literature^42,43^ it is observed that this type of change in the negative band (decreases to zero) in the CD spectra may be due to shift from B-form to A-form of DNA^44,45^. This phenomenon has been observed in the case of groove binding of some molecules with right-handed B form of DNA^46^. From the molecular docking analysis, it is observed that the long chain aliphatic amine ligands in the complexes **1** and **2** act as “hand to hug” the DNA base pair in the minor groove region. This kind of groove binding has been observed in some of the long aliphatic amine-containing metal complexes upon interaction with DNA. The enhancement of the positive band of DNA at 275 nm is due to distortions of DNA structure due to the metal-complex interaction^47^. However, in the presence of cobalt complexes the intensity of the positive band has been found to increase together with red shift. These observations are supportive of the intercalative mode of binding of the metal complexes^48^, where in the stacking of the metal complex molecules between the base pairs of DNA leads to an enhancement in the positive band^49,50^.

#### Viscosity measurement

The mode of interaction between DNA and complexes has been further confirmed using viscosity measurements. The intercalation between DNA and metal complex causes lengthening of the DNA helix and increases DNA’s relative viscosity^51^. Ethidium bromide (EB) is a well known DNA intercalator which was used as a standard to make sure our viscosity determination was working properly. The interaction between DNA and EB leads to significant increase in the viscosity of the DNA solutions. The influence of complexes **1** and **2** on the viscosity of DNA is given in Fig. S5. The results show that upon addition of both the complexes to DNA, the relative viscosity of DNA increased. The increase in relative viscosity follows the DNA-intercalating ability and the order of relative viscosity is EB > **2** > **1**. Hence, based on the previous reports and observation of mild increase in viscosity in the present study it can be considered that the interaction between the complexes and DNA may be via intercalation.

### BSA binding studies

#### Fluorescence quenching of BSA

Protein - drug interaction study is very important in medicinal chemistry as it is closely related to drug efficiency in the treatment of many diseases^52^. The absorption, transportation, distribution and metabolism of drugs strongly depend on their binding properties with the carrier protein^53,54^. Therefore, fluorescence quenching experiments were conducted to find the effect of binding of the complexes **1** and **2** with BSA.

**Figure 3:**
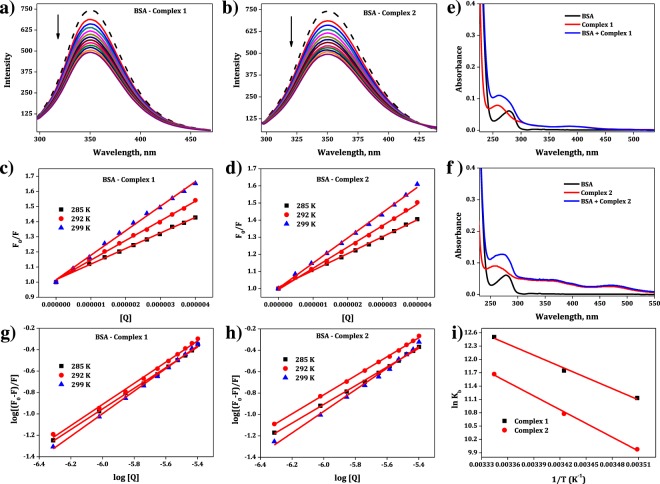
The fluorescence emission spectra of BSA in the presence of complexes **1 (a)** and **2 (b)**. The dashed line shows the intensity of BSA in the absence of complexes. After addition of the complexes **1** and **2** ([complex] = 0–4 × 10^−6^ M; [BSA] = 1.35 × 10^−6^ M) to BSA, the emission intensity of BSA was decreased which is indicated by “arrow”. Stern-Volmer plot for quenching of BSA by cobalt(III) complexes **1 (c)** and **2 (d)**. The UV-Vis absorption spectra of BSA and complexes **1 (e)** and **2 (f)**. Plot of log[(F_0_ − F)/F] vs log [Q] for BSA- cobalt(III) complexes **1 (g)** and **2 (h)**. van’t Hoff plot for the interaction of BSA with complexes **1** and **2 (i).**

In the present study, upon excitation at 280 nm, BSA gives a strong fluorescence emission peak at 350 nm due to the tryptophan residues. The decrease in emission spectra of BSA by increasing the concentration of complexes **1** and **2** are given in Fig. 3. It indicates that interaction of the complexes with BSA causes conformational changes in the protein structure around the tryptophan microenvironment of BSA^55^. The obtained fluorescence quenching results were analyzed using the Stern-Volmer eqation^56^,

$$\begin{array}{ccc}{{\rm{F}}}_{0}/{\rm{F}} & = & 1+{{\rm{K}}}_{{\rm{s}}{\rm{v}}}[{\rm{Q}}]\\ {{\rm{k}}}_{{\rm{q}}} & = & {{\rm{K}}}_{{\rm{s}}{\rm{v}}}/{\tau }_{0}\end{array}$$

where F_0_ and F are the fluorescence emission intensities of BSA in the absence and presence of complexes **1** and **2**. K_SV_ is the Stern-Volmer quenching constant, [Q] is concentration of the complexes **1** and **2**, k_q_ is the quenching rate constant of BSA and τ_0_ is the average lifetime of the emitter in the absence of quencher. The above equation was applied to determine K_SV_ by linear regression of a plot of F_0_/F against [Q].

Normally, quenching can take place either as dynamic quenching, static quenching or a combination of both mechanisms. Static quenching refers to fluorophore-quencher complex formation in the ground state while dynamic quenching refers to a process in which the fluorophores and the quencher come into contact during the transient existence of the excited state^57,58^. The plots of F_0_/F versus [Q] (Fig. 3) show that they are linear, indicating that a single quenching mechanism has operated for the complexes **1** and **2**. To find the nature of this single mechanism (static or dynamic), the K_sv_ values were obtained from the plots (Fig. 3), assuming the value of τ_o_ as 10^−8^ s^59^, wherein the quenching rate constants k_q_ values at corresponding temperature were calculated and shown in Table 3.

**Table 3:** The quenching constant, binding constant, number of binding sites and thermodynamic parameters for the interactions of the complexes **1** and **2** with BSA.

Complexes	Temp (K)	Quenching Constant (k_q_) (10^13^ M^−1^ s^−1^)	Binding Constant (K_b_)	Binding Site (n)	∆G° (kJ mol^−1^)	∆S° (J mol^−1^ K^−1^)	∆H° (kJ mol^−1^)
Complex **1**	285	1.6763	6.8359 × 10^4^	0.9631	−26.100		
	292	1.3531	1.0363 × 10^5^	0.9878	−28.036	+335.928	+69.429
	299	1.0862	2.1217 × 10^5^	0.9982	−31.086		
Complex **2**	285	1.4802	2.1547 × 10^4^	0.8695	−23.642		
	292	1.2245	3.2151 × 10^4^	0.8732	−25.195	+383.182	+85.617
	299	1.0114	1.1752 × 10^5^	0.9937	−29.010		

The observed k_q_ values are greater than the maximum collision quenching constant for various kinds of quenchers including biopolymers (2 × 10^10^ M^−1^ s^−1^) reported in the literature^60,61^ and this suggests that the complexes **1** and **2** interact with BSA via static mechanism.

#### Absorption spectroscopic studies

Besides fluorescence method, the mechanism of quenching was confirmed by monitoring the effect of presence of cobalt(III) complexes (quencher) on the absorption spectra of BSA. Collisional quenching (dynamic) will only affect the excited state of the fluorophores, and thus no change in the absorption spectra of the fluorophores is observed whereas ground state complex formation (static quenching) will disturb the absorption spectrum of the fluorophores. The absorption spectrum of BSA in the presence of complexes **1** and **2** is shown in Fig. 3. As seen from the figure, the absorption spectrum of BSA shows significant changes around 280 nm in the presence of complexes **1** and **2**. The changes in absorption spectra of the present cobalt(III) complexes indicate that a ground state complex formation took place between BSA and the complexes **1** and **2**. This result supports the fluorescence binding study.

#### Binding constant (K_b_) and number of binding sites (n)

For the static quenching process, the binding constant (K_b_) and the number of binding sites (n) can be determined by the following equation^62^

$$\mathrm{log}[({{\rm{F}}}_{0}-{\rm{F}})]/{\rm{F}}=\,\mathrm{log}\,{{\rm{K}}}_{{\rm{b}}}+{\rm{n}}\,\mathrm{log}[{\rm{Q}}]$$

where F_0_ and F are the fluorescence intensities of the emitter in the absence and presence of the quencher, respectively, and [Q] the concentration of the quencher. Thus, a plot of log F_0_ − F/F versus log [Q] (Fig. 3) was used to determine K_b_. From Fig. 3, it is found that all the correlation coefficients of samples are almost equal to 0.98, indicating that the binding of both the cobalt(III) complexes to BSA agreed well with the site-binding model as described by the above equation. The K_b_ values along with “n” number of binding sites at three different temperatures (285, 292 and 299 K) thus obtained for these complexes are listed in Table 3.

As seen from this table, the values of “n” are approximately equal to 1 which indicates the existence of a single binding site for both the complexes with BSA. BSA contains two tryptophan residues viz., Trp 134 and Trp 213, in which Trp 134 is present at the surface of the protein and Trp 213 is located in domain IIA.

Therefore, the present complexes bind either with Trp 134 or Trp 213 of BSA. Many reports suggest that metal complexes containing these kinds of long chain aliphatic amine ligands prefer to interact with Trp 213 as it is present in the hydrophobic sub-domains IIA of BSA^63–65^. Hence, the complexes **1** and **2** also bind with Trp 213 and induce changes in the domain’s microenvironment.

From the K_b_ values it is observed that the binding affinity of complex **1** is stronger than that of complex **2** with BSA. The difference in the binding affinities of these two complexes is possibly due to the presence of two difference Schiff base ligands (salen and salophen)^66^. These two ligands differ in the three dimensional orientation which significantly influence their interaction with amino acids which are present in the hydrophobic sub-domain IIA of BSA. Hence, the observed results indicate that salen ligand-containing complex **1** strongly interacts with Trp 213 microenvironment (hydrophobic sub-domain IIA) than salophen ligand containing complex **2**.

#### Thermodynamic parameters and mode of interaction

In general, intermolecular interacting forces between a small molecule and a biomacromolecule include hydrogen bonding, van der Waals force, and electrostatic and hydrophobic interactions^67^. The thermodynamic parameters, enthalpy change (ΔH°) and entropy change (ΔS**°**) of the binding are important for confirming the binding mode. For this reason, these thermodynamic parameters have been calculated using the equation,

$$\begin{array}{rcl}{{\rm{lnK}}}_{{\rm{b}}} & = & \frac{-{\rm{\Delta }}{\rm{H}}^\circ }{{\rm{RT}}}+\frac{{\rm{\Delta }}{\rm{S}}^\circ }{{\rm{R}}}\\ {\rm{\Delta }}{\rm{G}}^\circ  & = & {\rm{\Delta }}{\rm{H}}^\circ -{\rm{T}}{\rm{\Delta }}{\rm{S}}^\circ =-\,{{\rm{RTInK}}}_{{\rm{b}}}\end{array}$$

where K_b_ is the binding constant at the corresponding temperature, and R is the gas constant. The ΔH° and ΔS° values are calculated from the slope and intercept from plotting ln K_b_ versus 1/T (Fig. 3).

Rose and Subramanian^67^ have reported that the various types of forces of interaction that act between any compound and the protein could be indicated by the sign and magnitude of the thermodynamic parameters as follows: (i) the positive values for both ΔH**°** and ΔS° correspond to the involvement of hydrophobic forces in protein binding, (ii) the negative values for both ΔH° and ΔS° correspond to van der Waals and hydrogen bonding interactions, and (iii) the negative value of ΔH° and the positive value of ΔS° indicate electrostatic interaction.

In Table 3 it is seen that all the ΔG° values for the binding of complexes **1** and **2** are negative, which indicate that the binding processes are spontaneous. The positive values obtained for both ΔH° and ΔS° support the presence of strong hydrophobic forces of interaction between the cobalt(III) complexes and BSA.

### DFT calculations

Over the years, density functional theory (DFT) calculations have been used extensively in exploring the electronic properties of organic and inorganic molecules^68–71^. Hence, DFT calculations are performed at B3LYP level with LANL2DZ ECP for Co and 6–31 g(d,p) for H, C, N and O atoms using Gaussian09 software^72^. The optimized geometries of the metal complexes **1** and **2** are shown in Fig. S6, and the same is used for molecular docking analysis. The theoretical calculations show that, in both the complexes, the two oxygen atoms and two nitrogen atoms connected to cobalt are in same plane (~0°) while the other two amine ligands attached to the metal atom are perpendicular to this plane. It is interesting to note that the salicylaldehyde aromatic rings are slightly twisted (~25°) from the plane. Similarly, in complex **2** the benzene ring of orthophenylenediamine is slightly deviated from the planarity (~10°).

The computed bond lengths indicate that these complexes show excellent delocalization. For instance, the calculated C-C bond lengths (~1.40–1.50 Å) lie between their single (1.54 Å) and double (1.32 Å) bond limits. Overall, both the complexes have rigid core moiety with flexible methyl chain units. The selected bond parameters from optimized geometry of the metal complexes **1** and **2** are given in Table S1.

#### Analysis of molecular docking of the complexes with DNA

**Figure 4:**
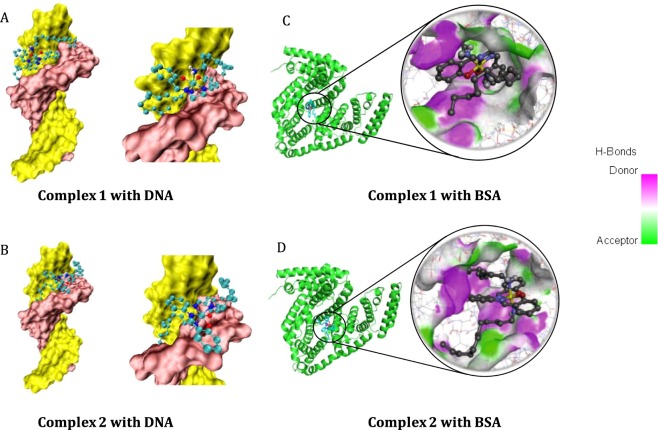
The binding poses of the metal complexes **1 (A)** and **2 (B)** into the DNA duplex obtained from molecular docking analysis.The binding poses of the metal complexes **1 (C)** and **2 (D)** into the BSA obtained from molecular docking analysis. (For clarity the hydrogen atoms of the metal complexes are omitted).

It is well known that docking analysis is often used to confirm the possible binding modes of metal complexes toward nucleic acids. Therefore, molecular docking studies have been carried out. The binding mode of these complexes towards DNA has been studied and the energetically most probable docked poses are given in Fig. 4, from, which it is clear that both the complexes bind with DNA near the minor groove. The salicylaldehyde aromatic rings interact with the guanine base pair through hydrogen bonding. For instance, the two oxygen atoms interact with the -NH_2_ group of guanine. Further, it is identified that the long aliphatic chain shows interactions with the backbone of the phosphate group near the minor groove region. Both complexes **1** and **2** are stabilized inside the DNA duplex through hydrogen bonding and as well as other non-covalent interactions. The docking studies show that binding strengths of complexes **1** and **2** are 5.3 and 5.5 kcal/mol respectively. The docking energies are not significantly different for either complexes or 0.2 kcal/mol difference cannot support the difference in binding constants measured.

#### Molecular docking analysis of the complexes with BSA

To confirm the nature of binding of the complexes **1** and **2** with BSA, molecular docking has been performed using the PDB id 4F5S. Autodock program was used, and docking was processed with setting of the grid sizes 60, 60 and 60 along the X-, Y- and Z- axes with 0.4 Å spacing. This docking grid covered all the active residues including the binding sites corresponding to Trp 213. These docking results show that binding of complex **1** with BSA is stronger than complex **2**. The binding energy of complexes **1** and **2** are 10 and 8.61 kcal/mol, respectively. The most probable docking poses are given in Fig. 4. It is interesting to note that upon binding, the flexible long aliphatic chain of complexes **1** and **2** undergoes twist and turn to fit into the active site of the sub domain IIA of BSA.

The binding pocket of complex **1** - BSA complex consists of the following residues: Trp213, Tyr451, Tyr149, Arg217, Leu237, Leu259, Ala260, Ala290, Glu152, His287 and Ser191. The neighbouring residues of complex **2** inside the BSA are Trp213, Arg217, Glu339, Ala341, Ser191, Arg194, Arg198, Lys294, Leu237, Pro338 and Glu291. The most possible binding site for both the complexes is in the proximity of Trp 213 in BSA.

### Anti-cancer studies

The anti-proliferative property of the complexes **1** and **2** at different concentrations on A549 lung carcinoma cell and monkey kidney epithelial cell Vero was examined by adopting MTT assay (Fig. S7). The IC_50_ of the complexes **1** and **2** for A549 cell are 80 μM and 65 μM, respectively, indicating that complex **2** is more efficient in affecting viability of A549 lung cancer cells than complex **1**. Neither of the complexes produced significant toxicity to Vero cells up to 150 μM concentration. The naked cobalt acetate and ligands salen and salophen were separately tested on A549 cells to understand if metal chelation is the principal basis of the anticancer activity of the complexes. Neither metal ion nor ligands, separately, was cytotoxic to the A549 lung cancer cells up to 200 μM concentration.

Besides, the cobalt(III) complexes containing these ligands show better cytotoxicity against lung cancer cells than previously reported long chain aliphatic amine-coordinated cobalt dien complexes^20^. Many reports suggest that there is a strong connection between DNA binding affinity and anti-cancer activity^66,73,74^. Therefore, the difference in DNA binding affinity is one of the possible reasons for the better activity of complex **2** than **1**. Yet, more biological study is needed to fully understand the mechanism for cell death.

#### Assessment of mode of cell death based on morphological features

**Figure 5:**
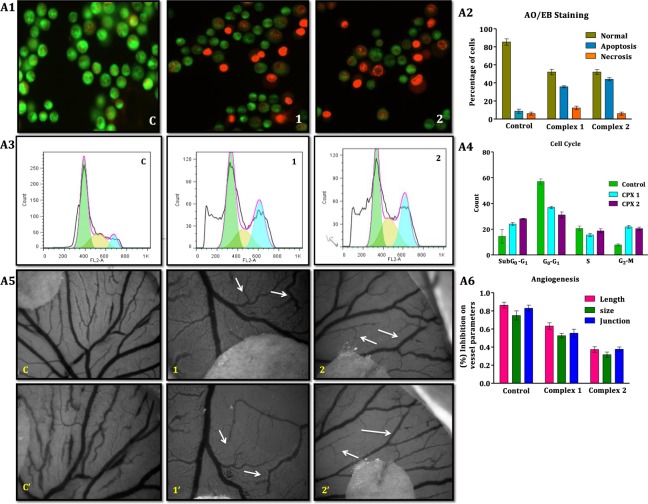
**(A1)**. Morphological assessment of control (A1 c; the cells are viable as inferred from the green fluorescence) and treated (A1, 1 complex 1; & A1, 2 complex 2) A549 lung cancer cells. **(A2)** The graph shows data on percentage of cells that are normal and those afflicted with apoptosis and necrosis in the control and 24 h treated groups. **(A3)** The effects of complexes **1** and **2** on the cell cycle progression in lung cancer A549 cells. **(A4)** The bar diagram displays higher percentage of cells in sub G_0_ − G_1_ and G_2_-M phases after treatment of A549 cancer cells with complexes **1** and **2**. The cell cycle distribution was analyzed using Dean-Jett-Fox software and depicted as the histogram. (**A5**) The vascular sprouting has been damaged (marked by arrows) on exposure to complexes **1** and **2** (10 μM) compared to control at 0 h (C, 1 and 2) after 6 h (C′, 1′ and 2′) of treatment. (**A6**) The different angiogenic parameters such as vessel length, vessel size and number of junctions decreased on exposure to complexes **1** and **2** compared to control. (Data are expressed in Mean ± SD).

After the treatment of A549 cells with the complexes **1** and **2** at the respective IC_50_ concentrations for 24 h the cells were observed in the fluorescent microscope. The cytological changes indicated that, apart from the normal green-fluorescing cells, most of the cells succumbed to apoptosis and a few to necrosis. Many treated cells showed early apoptotic morphological features such as peri-nuclear chromatin condensation with green patches and fragments of chromatin^75^. There were also cells in late stages of apoptosis such as chromatin fragmentation and apoptotic body formation (Fig. 5). A few necrotic cells, with orange to red fluorescing nuclei but no indication of chromatin fragmentation, were also seen. The percentages of viable, apoptotic and necrotic cells are given in Fig. 5 as bar diagram.

#### Cell cycle analysis

The possible cell cycle arrest was determined by flow cytometer which is used to distinguish and quantify the cells in different phases of the cell cycle. The cells were treated with the complexes **1** and **2** at the respective half maximal inhibitory concentrations. The cells were fixed, stained with propidium iodide and analysed for DNA/cell content as aspects of cell cycle distribution. The DNA histograms and the percentage of cells in each of the phases are given Fig. 5. Compared to the control cells, the complexes **1** and **2** treated cells showed gradual increase in sub G_0_ − G_1_ and G_2_ − M phases. The increase of cell accumulation in sub G_0_ - G_1_ phase indicated delay in progression of cell cycle. The cells thus inhibited in division end up in the apoptosis due to sub G_0_ − G_1_ check point arrest^76^. The increase of cells at G_2_ − M phase indicates that those cells which already passed S-phase before the treatment succumb to G_2_ − M phase check point arrest. In as much as apoptosis itself could be induced due to the DNA damage, necrosis is a clear indication of damage caused by the complexes to the cytosol.

### Anti-angiogenic property

Chicken chorioallantoic membrane (CAM) assay is a well-known *in vivo* angiogenesis assay. To confirm the anti-angiogenic properties of cobalt complexes **1** and **2**, CAM assay was performed. The complexes **1** and **2**, on incubation for four hours, produced excellent inhibitory activity on the developing blood vessels. The results are given in Fig. 5. Eggs incubated with the vehicle control, DMSO (<0.01%), showed remarkable development of vascular sprouting in the chick embryo after 6 h of treatment. However, the cobalt complexes **1** and **2** produced profound inhibition of vessel formation at the end of the incubation (Fig. 5). Several angiogenic parameters such as vessel size, vessel length and number of vessel branches were quantified using angioquant software as practiced in an earlier study^77^ and represented as bar diagram (Fig. 5). The cobalt complexes **1** and **2** produced reduction in all of these parameters compared to control. It clearly indicated that the cobalt complexes of interest possess anti-angiogenic potential. Among the two cobalt complexes, complex **2** produced higher anti-angiogenic activity than complex **1**. This trend is also in tune with the DNA binding ability and anti-cancer activity of the complexes **1** and **2**.

## Conclusion

Studies on binding of cobalt(III) Schiff base complexes **1** and **2**, containing long chain aliphatic amine with DNA and BSA, have been studied. Both the cobalt(III) complexes interact with DNA through intercalation as well as hydrophobic modes. In the case of protein binding studies, both the complexes showed strong affinity towards BSA. The thermodynamic parametric studies indicate that the nature of interaction between the complexes and BSA is mainly due to hydrophobic forces. DFT and molecular docking studies reveal that complex **2** shows slightly better binding affinity towards DNA and BSA compared to complex **1**. The cytotoxic property of complexes **1** and **2** was investigated against A549 lung cancer cells and Vero normal kidney cells. The complex **2** proved to be more efficient against A549 cells than complex **1**, with little, if any, effect caused to Vero cells. The AO/EB staining assay revealed that the mode of cell death is essentially apoptosis. Cell cycle analysis also indicated that both the complexes cause death of A549 cells through apoptosis and arrest of cell cycle greatly in G_2_-M phase but to a certain extent at G_2_-M. Both the complexes significantly inhibit the CAM development. Thus, the present investigation clearly indicates that the cobalt Schiff base metal complexes in this study are promising dual acting agents that could be developed into chemotherapeutics for cancers in general, and lung cancer in particular.

## Experimental Section

### Materials

All reagents and chemicals were of analytical grade, and purchased from commercial sources and used as received. Bovine serum albumin (BSA), CT DNA and dodecyl amine (DA) were obtained from Sigma Aldrich (India). Cobalt(II) acetate tetrahydrate and ethylenediamine (en) were obtained from Merck, India. Salicylaldehyde, methyl amine and orthophenylenediamine (OPDA) were obtained from Loba Chemie, India. Doubly distilled water was used throughout the experiments. The stock solutions of CT-DNA and BSA were prepared in PBS at pH 7.4, stored at 4 °C and used within 3 days.

The carbon, hydrogen and nitrogen content of the samples were determined on a Thermo Finnigan (FLASH EA 1112) microanalyzer. Electrospray ionisation mass spectrometry (ESI-MS) analysis was performed in the positive ion mode on a liquid chromatography-ion trap mass spectrometer (water Q-TOF micro mass spectrometer) at room temperature. Infra-red spectra were recorded on FT-IR Perkin Elmer spectrophotometer with samples prepared as KBr pellets. ^1^H and ^13^C NMR spectra were recorded on a BRUKER 400 MHz using CDCl_3_ as solvent. Absorption spectra were recorded on a UV-1800 Shimadzu spectrophotometer using cuvettes of 1 cm path length, and emission spectra were recorded on a JASCO FP 770 spectrofluorimeter. Cyclic voltammetry measurements were made on Princeton EG and G-PARC model potentiostat. Circular dichroism (CD) spectra were recorded on a JASCO - J810 spectropolarimeter with a cylindrical cuvette of 0.1 cm path length.

### Methods

#### Synthesis of ligands

The ligands salen and salophen were prepared by condensation of 1,2 - ethylenediamine/orthophenylenediamine with salicylaldehyde in the 1:2 molar ratio in methanol and the crude products were recrystallized in methanol^78^.

#### Synthesis of complex *trans*-[Co(salen)(DA)_2_]ClO_4_ [1]

To a refluxing solution of salen (1 mmol) in methanol (10 cm^3^), Co(OAc)_2_.4H_2_O (0.25 g, 1 mmol) was added. After 30 min, dodecylamine (0.2 cm^3^, 0.8 mmol) was added to the above reaction solution. The reaction mixture was refluxed for an hour. The cobalt(II) complex that formed was oxidized by blowing air into the solution for 2 h, and then the solution was filtered. An appropriate amount of sodium perchlorate was added to this filtrate. Brown-coloured crystals of the titled complex were formed in the solution after 48 h and were filtered off. The crude products were purified by silica gel column chromatography using hexane/ethyl acetate as eluent. Yield: 75%; Anal. for C_40_H_68_CoN_4_O_6_Cl (%): C, 60.40; H, 8.62; N, 7.04. Found (%): C, 59.89; H, 8.52; N, 7.30. FT – IR (KBr, cm^−1^): 3435 cm^−1^ (γ N-H), 1605 cm^−1^ (γ C=N), 1312 cm^−1^ (γ C-O), 1089 cm^−1^ (γ ClO4). UV–Vis, λmax (nm) (DMSO): 262 nm, 388 nm and 480 nm. ^1^H NMR (400 MHz, CDCl_3_, δ, ppm):0.80–0.90 (6H, CH_3_), 0.98–1.17 (36H, CH_2_), 1.80 (4H, CH_2_), 4.10 (4H, ethylene diamine CH_2_ proton), 6.57 (2H, aromatic), 7.14 (2H, aromatic), 7.26 (3H, aromatic), 7.96 (2H, imine). ESI-MS of complex **1** in MeOH displayed [M]^+^ peaks at m/z 695.35. Besides, ESI-MS showed [M-DA]^+^ and [M-2DA]^+^ peak at m/z 510.16 and 325.03, respectively.

#### Synthesis of complex *trans*-[Co(salophen)(DA)_2_]ClO_4_ [2]

The complex **2** was synthesised by adopting the same procedure as above with slight modification in the experimental condition, i.e., instead of salen ligand, salophen ligand was used. Yield: 85%; Anal. for C_44_H_68_CoN_4_O_6_Cl (%): C, 62.66; H, 8.13; N, 6.64. Found (%): C, 62.62; H, 7.58; N, 6.38. FT – IR (KBr, cm^−1^): 3434 cm^−1^ (γ N-H), 1610 cm^−1^ (γ C=N), 1328 cm^−1^ (γ C-O), 1086 cm^−1^ (γ ClO4). UV–Vis, λmax (nm) (DMSO): 263 nm, 368 nm and 472 nm. ^1^HNMR (400 MHz, CDCl_3_, δ, ppm): 0.83–0.89 (t, 6H, CH_3_), 0.89–1.26 (36H, CH_2_), 1.80 (4H, CH_2_), 2.49 (2H, CH_2_), 6.7 (2H, aromatic), 7.22 (2H, aromatic), 7.44 (4H, aromatic), 8.1 (2H, aromatic), 8.16 (2H, imine). ESI-MS of complex **2** in MeOH displayed [M]^+^ peak at m/z 743.31. Further ESI-MS indicates [M-DA]^+^ and [M-2DA]^+^ peaks at m/z 558.18 and 373.03 respectively.

#### DNA binding experiments

A solution of DNA in the PBS gave a ratio of UV absorbance at 260 nm to 280 nm of ~1.8–1.9:1, indicating that the DNA was sufficiently free of protein. Concentrated stock solution of DNA was prepared in PBS and sonicated for 25 cycles, where in each cycle consisted of 30 seconds with 1 min intervals. The DNA concentration per nucleotide was determined by electronic absorption spectroscopy using the known molar extinction coefficient value of 6600 M^−1^ cm^−1^ at 260 nm^79^.

The stock solutions were prepared by dissolving the complexes **1** and **2** in an aqueous solution of DMSO as the co-solvent, and then diluted suitably with the corresponding buffer to the required concentrations for all the experiments. The final DMSO concentration never exceeded 0.5%. For absorption and emission spectral experiments, the DNA solutions were pre-treated with the solutions of metal complexes to ensure no change in the metal complex concentrations.

Absorption studies were performed by maintaining the constant concentration of both the complexes and varying the CT-DNA concentration. An equal amount of CT-DNA was added to the complexes and the reference solution to eliminate the absorbance of CT-DNA itself.

For fluorescence quenching experiments, nucleic acids solution was pretreated with ethidium bromide (EB) for 30 min. Solutions of cobalt(III) complexes were then added to this mixture and their effect on the emission intensity was measured. The samples were excited at 450 nm and emission was observed between 500 and 750 nm.

For cyclic voltammetry experiments, the electrode surfaces were freshly polished with alumina powder and then sonicated in ethanol and distilled water for 1 min prior to each experiment and the electrode was rinsed with doubly distilled water thoroughly between each polishing step. Finally, each electrode was cleaned thoroughly in an ultrasonic cleaner again with doubly distilled water. Cyclic voltammetric experiments were performed at room temperature in Tris buffer in a single compartment cell with a three-electrode configuration, namely, glassy carbon working electrode, platinum wire auxiliary electrode and saturated calomel as reference electrode. Before experiments, all solutions were deaerated with dry nitrogen gas for 10 min to remove dissolved oxygen and kept in nitrogen atmosphere throughout the experiments.

CD spectra of CT–DNA in the absence and presence of cobalt(III) complexes **1** and **2** were recorded in phosphate buffer. Each sample solution was scanned in the range of 200–320 nm, and final CD spectra were generated after averaging three scans subtracting the buffer background. Viscosity experiments were performed using an Ubbelohde viscometer at a constant temperature (30.0 ± 0.2 °C).

#### BSA binding experiments

The concentration of BSA prepared in PBS buffer (pH = 7.4) was determined spectrophotometrically using the extinction coefficient of 43,800 M^−1^ cm^−1^ at 280 nm^80^. The fluorescence spectra of BSA were recorded at room temperature with an excitation wavelength of BSA at 280 nm and the emission at 350 nm by keeping the concentration of BSA constant (1.35 × 10^−6^ M^−1^) while increasing the cobalt(III) complex concentration regularly.

UV-Visible absorption spectra of BSA and BSA in the presence of cobalt(III) complexes were recorded at room temperature in the region between 200 and 800 nm.

#### Docking studies

For docking studies, B3LYP functional was used for the optimization of structures of complexes **1** and **2**. It is well known that B3LYP functional is used to give reasonably good results. Therefore, geometry of the synthesized complexes **1** and **2** was optimized at B3LYP/ (LANL2DZ(Co), 6–31 g(d,p) for other atoms) level using G09W program^81–86^. For the molecular docking analysis, the DFT optimized geometries were considered. AUTODOCK4.0 was used to understand how these metal complexes interact with DNA and BSA^87^. The docking parameters were set to include complex-DNA/BSA interactions and various non-covalent interactions as implemented in these programs. For the molecular docking of DNA duplex, d(CGCGAATTCGCG)_2_ dodecamer) was obtained from Protein Data Bank (PDB) and the PDB ID was 355D^88,89^. For the molecular docking of these metal complexes into the BSA protein, the available crystal structures of BSA, PDB ID: 4F5S, were considered^90^.

#### Anti-cancer studies

Culture: Human lung cancer cells (A549) were received from National Centre for Cell Science (NCCS) Pune, India. Vero cells were obtained from ATCC. The cells were grown at humidified atmosphere of 5% CO_2_ in an incubator at 37 °C (Forma, Thermo Scientific, USA) in DMEM medium (Invitrogen) containing 10% FBS (Sigma-Aldrich, St. Louis, Mo, USA), 100 μg/mL of penicillin and 100 μg/mL streptomycin were used as antibiotics (Himedia, Mumbai, India). The MTT tetrazolium salt colorimetric assay was performed to measure the cytotoxicity as revealed in cell viability^91^. The cells were seeded in 96 well plates (5000 A549 cells per well) and allowed to attach for 24 h. The cells were then incubated with different concentrations of the metal complexes **1** and **2** dissolved in 100% dimethyl sulfoxide (DMSO) (Sigma-Aldrich) and prepared to a final dilution of 0.02% of DMSO.

In the determination of IC_50_ value, 0.02% DMSO was used as the solvent control. After the end of incubation period (24 h), the medium was aspirated carefully and replaced with new medium. The formazan formed was dissolved in100 μL of 100% DMSO to each well. The absorbance was monitored at 570 nm using a 96 well plate reader (Bio-Rad, Hercules, California, USA). All experiments were repeated for a minimum of three times when each experiment was done in two replicates. The percentage inhibition was calculated, from these data using the formula:

$$=\,\frac{{\rm{Mean}}\,{\rm{OD}}\,{\rm{of}}\,{\rm{untreated}}\,{\rm{cells}}\,({\rm{control}})-{\rm{Mean}}\,{\rm{OD}}\,{\rm{of}}\,{\rm{treated}}\,{\rm{cells}}}{{\rm{Mean}}\,{\rm{OD}}\,{\rm{of}}\,{\rm{untreated}}\,{\rm{cells}}\,({\rm{control}})}\times 100$$

AO/EB Staining of A549 cells: The mode of cell death induced by the complexes was deciphered using acridine orange (AO) and ethidium bromide (EB) staining protocol^92^. Cells were cultured in 6 well plates, treated with the complexes and allowed for 24 h. Then the cells were incubated with AO and EB solutions (1 part of 100 μg/mL AO and 1 part of 100 μg/mL of EB in PBS) and examined in a fluorescent microscope (Carl Zeiss, Jena, Germany) using a UV filter (450–490 nm). Three hundred cells per sample were counted in triplicate for each dose point. The cells were scored as viable, apoptotic and necrotic as judged by the staining pattern, nuclear morphology and cytoplasmic integrity. The percentage of apoptotic and necrotic cells were calculated and the morphological changes were also photographed^93^.

Cell cycle analysis: Cell phase distribution was determined by flow cytometry with DNA staining to reveal the total amount of DNA^94^. Approximately 1 × 10^6^ non-small cell lung carcinoma (A549) cells were treated with IC_50_ concentrations of complexes **1** and **2** and incubated for 24 h. After incubation, the cells were trypsinized, harvested, fixed in 1 mL 80% cold ethanol and incubated at 4 °C for 15 min. Then, cells were centrifuged at 1500 rpm for 5 min and the cell pellets were resuspended in 500 μL propidium iodide (10 μg/mL) containing 300 μg/mL RNase (Sigma Chemical Co., St. Louis, MO, USA). The cells were then incubated on ice for 30 min and filtered in 53μm nylon mesh. Cell cycle distribution was analyzed using FACS (fluorescent activated cell sorter) (Becton-Dickinson, Sanjose, CA, USA) with 15 mW, 488 nm argon ion laser. The propidium iodide signals were collected using a 585/42 band pass filter. The data were acquired and analyzed using Dean-Jett-Fox algorithm.

#### Chicken chorioallantoic membrane assay (CAM)

The Institutional Animal Care and Use Committee, an Association of New England Medical Center and Tufts (IACUC) and the National Institute of Health, USA, had established that a chick embryo that has not reached the 14^th^ day of its developmental period would not experience pain and can, therefore, be used for experimentation without any ethical restrictions or prior protocol approval^95–97^. The anti-angiogenic activity was investigated on adopting the Chicken chorioallantoic membrane (CAM) assay^24^. Briefly, specific pathogen-free (SPF) fertile chicken eggs were obtained from a government poultry farm. The eggs were incubated at 37 °C in a humid atmosphere. On the 5^th^ day of incubation, the shell was cautiously broken using forceps. Precautions were taken to prevent puncture of any of the blood vessels during the transfer. The stock solutions were prepared by dissolving the complexes **1** and **2** in DMSO and then diluted suitably with the corresponding buffer to the required concentrations for all the experiments. The final DMSO concentration never exceeded 0.1%. Sterile filter paper discs (6 mm dia) soaked in solution of complexes (10 µM) were placed in 3 different positions over the generating blood vessels. For the control experiments sterile filter paper disks (6 mm dia) soaked with 10 µM of 0.1% of DMSO in PBS were used. Images of blood vessels (i.e., 0 h) were taken using stereo microscope (Nexius Zoom). The eggs were aseptically transferred in a humidified incubator at 37 °C and images were captured after 6 h incubation.

## Supplementary information

Supplementary information

## References

[1] Dilruba S, Kalayda GV (2016). Platinum-based drugs: past, present and future. Cancer Chemother. Pharmacol..

[2] Giaccone G (2004). Gefitinib in combination with gemcitabine and cisplatin in advanced non–small-cell lung cancer: a phase III trial—INTACT 1. J Clin Oncol..

[3] Zeng L (2016). Ruthenium (II) complexes with 2-phenylimidazo [4,5-f][1,10] phenanthroline derivatives that strongly combat cisplatin-resistant tumor cells. Scientific reports.

[4] Wang F-X (2016). Ester-modified cyclometalated iridium (III) complexes as mitochondria-targeting anticancer agents. Scientific reports.

[5] Khan RA (2017). Heteroleptic Copper (I) Complexes of “Scorpionate” Bis-pyrazolyl Carboxylate Ligand with Auxiliary Phosphine as Potential Anticancer Agents: An Insight into Cytotoxic Mode. Scientific reports.

[6] Qin J-L (2017). Oxoaporphine Metal Complexes (Co II, Ni II, Zn II) with High Antitumor Activity by Inducing Mitochondria-Mediated Apoptosis and S-phase Arrest in HepG2. Scientific reports.

[7] Schwartz JA, Lium EK, Silverstein SJ (2001). Herpes simplex virus type 1 entry is inhibited by the cobalt chelate complex CTC-96. J Virol..

[8] Munteanu CR, Suntharalingam K (2015). Advances in cobalt complexes as anticancer agents. Dalton Transactions.

[9] Glasner H, Tshuva EY (2011). A marked synergistic effect in antitumor activity of salan titanium (IV) complexes bearing two differently substituted aromatic rings. J. Am. Chem. Soc.

[10] Glasner H, Tshuva EY (2014). C 1-Symmetrical Titanium (IV) Complexes of Salan Ligands with Differently Substituted Aromatic Rings: Enhanced Cytotoxic Activity. Inorg. Chem..

[11] King AP, Gellineau HA, Ahn J-E, MacMillan SN, Wilson JJ (2017). Bis (thiosemicarbazone) Complexes of Cobalt (III). Synthesis, Characterization, and Anticancer Potential. Inorg. Chem..

[12] Lee S-Y (2010). [NiII (3-OMe-salophene)]: A Potent Agent with Antitumor Activity. J. Med. Chem..

[13] Matos CP (2013). New polydentate Ru (III)-Salan complexes: Synthesis, characterization, anti-tumour activity and interaction with human serum proteins. Inorganica Chim. Acta.

[14] Wu P (2009). Stabilization of G‐Quadruplex DNA with Platinum (II) Schiff Base Complexes: Luminescent Probe and Down‐Regulation of c‐myc Oncogene Expression. Chem. Eur. J.

[15] Meker S, Margulis‐Goshen K, Weiss E, Magdassi S, Tshuva EY (2012). High antitumor activity of highly resistant salan–titanium (IV) complexes in nanoparticles: An identified active species. Angew. Chem. Int. Ed.

[16] Würtenberger I (2014). Fluorinated Fe (III) Salophene Complexes: Optimization of Tumor Cell Specific Activity and Utilization of Fluorine Labeling for *in Vitro* Analysis. J. Med. Chem..

[17] Lange TS (2008). Iron (III)-salophene: an organometallic compound with selective cytotoxic and anti-proliferative properties in platinum-resistant ovarian cancer cells. PLoS One.

[18] Gust R, Ott I, Posselt D, Sommer K (2004). Development of cobalt (3,4-diarylsalen) complexes as tumor therapeutics. J. Med. Chem..

[19] Veeralakshmi S, Nehru S, Arunachalam S, Kumar P, Govindaraju M (2014). Study of single and double chain surfactant–cobalt (III) complexes and their hydrophobicity, micelle formation, interaction with serum albumins and antibacterial activities. Inorganic Chemistry Frontiers.

[20] Veeralakshmi S (2015). Single and double chain surfactant–cobalt (III) complexes: the impact of hydrophobicity on the interaction with calf thymus DNA, and their biological activities. RSC Adv..

[21] Zheng Y-R (2014). Pt (IV) prodrugs designed to bind non-covalently to human serum albumin for drug delivery. Journal of the American Chemical Society.

[22] Folkman J (1971). Tumor angiogenesis: therapeutic implications. N Engl J Med.

[23] Nowak-Sliwinska P (2011). Organometallic ruthenium (II) arene compounds with antiangiogenic activity. J. Med. Chem..

[24] Nagababu P (2015). Antiangiogenic activity of mononuclear copper (II) polypyridyl complexes for the treatment of cancers. Journal of medicinal chemistry.

[25] Zamora A (2015). Dual antitumor and antiangiogenic activity of organoplatinum (II) complexes. Journal of medicinal chemistry.

[26] Yellol J (2015). Novel C, N-Cyclometalated Benzimidazole Ruthenium (II) and Iridium (III) Complexes as Antitumor and Antiangiogenic Agents: A Structure–Activity Relationship Study. Journal of medicinal chemistry.

[27] Tanaka T (2005). Cobalt promotes angiogenesis via hypoxia-inducible factor and protects tubulointerstitium in the remnant kidney model. Laboratory investigation.

[28] Prabhakaran R (2004). Synthesis, characterization, EXAFS investigation and antibacterial activities of new ruthenium (III) complexes containing tetradentate Schiff base. Journal of inorganic biochemistry.

[29] Tamizh MM, Mereiter K, Kirchner K, Bhat BR, Karvembu R (2009). Synthesis, crystal structures and spectral studies of square planar nickel (II) complexes containing an ONS donor Schiff base and triphenylphosphine. Polyhedron.

[30] Rosenthal MR (1973). The myth of the non-coordinating anion. J. Chem. Educ.

[31] Ambika S, Arunachalam S, Arun R, Premkumar K (2013). Synthesis, nucleic acid binding, anticancer and antimicrobial activities of polymer–copper (II) complexes containing intercalative phenanthroline ligand (DPQ). RSC Advances.

[32] Pyle A (1989). Mixed-ligand complexes of ruthenium (II): factors governing binding to DNA. J. Am. Chem. Soc.

[33] Bloomfield, V., Crothers, D. & Tinoco, I. Jr. Physical Chemistry of Nucleic Acids Harper and Row. *New York* (1974).

[34] Kumar RS (2009). Surfactant–cobalt (III) complexes: synthesis, critical micelle concentration (CMC) determination, DNA binding, antimicrobial and cytotoxicity studies. J. Inorg. Biochem..

[35] Kumar RS, Arunachalam S (2008). Synthesis, micellar properties, DNA binding and antimicrobial studies of some surfactant–cobalt (III) complexes. Biophys. Chem..

[36] Ambika S, Manojkumar Y, Senthilkumar R, Sathiyaraj M, Arunachalam S (2016). Nucleic Acid Binding and Invitro Cytotoxicity Studies of Polymer Grafted Intercalating Copper (II) Complex. Journal of Inorganic and Organometallic Polymers and Materials.

[37] Manojkumar Y, Ambika S, Senthilkumar R, Arunachalam S (2017). Biophysical and biological studies of some polymer grafted metallo-intercalators. Colloids and Surfaces B: Biointerfaces.

[38] Lakowicz JR, Weber G (1973). Quenching of fluorescence by oxygen. Probe for structural fluctuations in macromolecules. Biochemistry.

[39] Carter MT, Bard AJ (1987). Voltammetric studies of the interaction of tris (1, 10-phenanthroline) cobalt (III) with DNA. Journal of the American Chemical Society.

[40] Carter MT, Rodriguez M, Bard AJ (1989). Voltammetric studies of the interaction of metal chelates with DNA. 2. Tris-chelated complexes of cobalt (III) and iron (II) with 1,10-phenanthroline and 2,2′-bipyridine. Journal of the American Chemical Society.

[41] Ivanov V, Minchenkova L, Schyolkina A, Poletayev A (1973). Different conformations of double‐stranded nucleic acid in solution as revealed by circular dichroism. Biopolymers.

[42] Shahabadi, N., Kashanian, S. & Fatahi, A. Identification of Binding Mode of a Platinum (II) Complex, PtCl. *Bioinorganic chemistry and applications* **2011** (2011). 10.1155/2011/687571 PMC3202101 22110411

[43] Selvakumar B, Rajendiran V, Maheswari PU, Stoeckli-Evans H, Palaniandavar M (2006). Structures, spectra, and DNA-binding properties of mixed ligand copper (II) complexes of iminodiacetic acid: The novel role of diimine co-ligands on DNA conformation and hydrolytic and oxidative double strand DNA cleavage. Journal of inorganic biochemistry.

[44] Poklar NA (1996). Influence of cisplatin intrastrand crosslinking on the conformation, thermal stability, and energetics of a 20-mer DNA duplex. Proceedings of the National Academy of Sciences.

[45] Li F-H (2006). Synthesis, characterization and biological activity of lanthanum (III) complexes containing 2-methylene–1, 10-phenanthroline units bridged by aliphatic diamines. Journal of inorganic biochemistry.

[46] Patra AK, Nethaji M, Chakravarty AR (2007). Synthesis, crystal structure, DNA binding and photo-induced DNA cleavage activity of (S-methyl-l-cysteine) copper (II) complexes of heterocyclic bases. Journal of inorganic biochemistry.

[47] Kasparkova J, Vrana O, Farrell N, Brabec V (2004). Effect of the geometry of the central coordination sphere in antitumor trinuclear platinum complexes on DNA binding. Journal of inorganic biochemistry.

[48] Woodson SA, Muller JG, Burrows CJ, Rokita SE (1993). A primer extension assay for modification of guanine by Ni (ll) complexes. Nucleic acids research.

[49] Chauhan M, Arjmand F (2006). Chiral and Achiral Macrocyclic Copper (II) Complexes: Synthesis, Characterization, and Comparative Binding Studies with Calf‐Thymus DNA. Chemistry & biodiversity.

[50] Vaidyanathan VG, Vijayalakshmi R, Subramanian V, Nair BU (2002). Synthesis, Characterization, and Binding of [Cr (naphen)(H_2_O) 2]+ with DNA: Experimental and Modeling Study. Bulletin of the Chemical Society of Japan.

[51] Liu F, Meadows KA, McMillin DR (1993). DNA-binding studies of Cu (bcp) 2+ and Cu (dmp) 2+: DNA elongation without intercalation of Cu (bcp) 2+. J. Am. Chem. Soc.

[52] Peters, T. Jr. *All about albumin: biochemistry, genetics, and medical applications*. (Academic press, 1995).

[53] Müller W, Wollert U (1979). Human serum albumin as a ‘silent receptor’for drugs and endogenous substances. Pharmacology.

[54] Tanimoto S, Takahashi D, Toshima K (2012). Chemical methods for degradation of target proteins using designed light-activatable organic molecules. Chem Comm..

[55] Senthilkumar R (2016). Plasma protein binding of anisomelic acid: Spectroscopy and molecular dynamic simulations. Journal of chemical information and modeling.

[56] Dewey, T. G. *Biophysical and biochemical aspects of fluorescence spectroscopy*. (Springer, 1991).

[57] Hu Y-J, Liu Y, Zhao R-M, Dong J-X, Qu S-S (2006). Spectroscopic studies on the interaction between methylene blue and bovine serum albumin. J. Photochem. Photobiol.

[58] Silva D, Cortez CM, Louro SR (2004). Chlorpromazine interactions to sera albumins: a study by the quenching of fluorescence. Spectrochim Acta A Mol Biomol Spectrosc.

[59] Pan X, Qin P, Liu R, Wang J (2011). Characterizing the interaction between tartrazine and two serum albumins by a hybrid spectroscopic approach. J Agric Food Chem..

[60] Lakowicz, J. R. *Principles of fluorescence spectroscopy*. (Springer Science & Business Media, 2013).

[61] Mandal G, Bardhan M, Ganguly T (2010). Interaction of bovine serum albumin and albumin-gold nanoconjugates with l-aspartic acid. A spectroscopic approach. Colloids and Surfaces B: Biointerfaces.

[62] Vignesh G, Manojkumar Y, Sugumar K, Arunachalam S (2015). Spectroscopic investigation on the interaction of some polymer–cobalt (III) complexes with serum albumins. Journal of Luminescence.

[63] Vignesh G, Sugumar K, Arunachalam S, Vignesh S, James RA (2013). A comparative study on the binding of single and double chain surfactant–cobalt (III) complexes with bovine serum albumin. Spectrochimica Acta Part A: Molecular and Biomolecular Spectroscopy.

[64] Vignesh, G. *et al*. Studies on the synthesis, characterization, human serum albumin binding and biological activity of single chain surfactant–cobalt (III) complexes. *Luminescence* (2015). 10.1002/bio.2991 26250655

[65] Vignesh G, Nehru S, Manojkumar Y, Arunachalam S (2014). Spectroscopic investigation on the interaction of some surfactant-cobalt (III) complexes with serum albumins. Journal of Luminescence.

[66] Zheng K (2014). Synthesis, structure and molecular docking studies of dicopper (II) complexes bridged by N-phenolato-N′-[2-(dimethylamino) ethyl] oxamide: the influence of terminal ligands on cytotoxicity and reactivity towards DNA and protein BSA. New J. Chem..

[67] Ross PD, Subramanian S (1981). Thermodynamics of protein association reactions: forces contributing to stability. Biochemistry.

[68] Sankarganesh M (2018). New bio-sensitive and biologically active single crystal of pyrimidine scaffold ligand and its gold and platinum complexes: DFT, antimicrobial, antioxidant, DNA interaction, molecular docking with DNA/BSA and anticancer studies. Bioorg. Chem..

[69] Lakshmipraba J, Arunachalam S, Vijay Solomon R, Venuvanalingam P (2015). Synthesis, DNA binding and docking studies of copper (II) complexes containing modified phenanthroline ligands. J. Coord. Chem..

[70] Lakshmipraba J (2015). Surfactant–copper (II) Schiff base complexes: synthesis, structural investigation, DNA interaction, docking studies, and cytotoxic activity. J. Biomol. Struct. Dyn..

[71] Naveenraj S (2018). A multispectroscopic and molecular docking investigation of the binding interaction between serum albumins and acid orange dye. Spectrochim Acta A Mol Biomol Spectrosc.

[72] Frisch, M. J. *et al*. Gaussian 09, Revision B.01. Gaussian Inc., Wallingford, CT (2010).

[73] Paul A (2015). Synthesis, DNA binding, cellular DNA lesion and cytotoxicity of a series of new benzimidazole-based Schiff base copper (II) complexes. Dalton Trans..

[74] Fu X-B (2014). Water-soluble DNA minor groove binders as potential chemotherapeutic agents: synthesis, characterization, DNA binding and cleavage, antioxidation, cytotoxicity and HSA interactions. Dalton Trans..

[75] Riyasdeen A (2014). Antiproliferative and apoptosis-induction studies of a metallosurfactant in human breast cancer cell MCF-7. RSC Advances.

[76] Tan C (2011). Synthesis, structures, cellular uptake and apoptosis-inducing properties of highly cytotoxic ruthenium-Norharman complexes. Dalton Trans..

[77] Barui AK (2012). Zinc oxide nanoflowers make new blood vessels. Nanoscale.

[78] Dalla Cort A, De Bernardin P, Forte G, Mihan FY (2010). Metal–salophen-based receptors for anions. Chem. Soc. Rev.

[79] Reichmann M, Rice S, Thomas C, Doty P (1954). A further examination of the molecular weight and size of desoxypentose nucleic acid. J. Am. Chem. Soc.

[80] Kaboudin B, Moradi K, Faghihi M, Mohammadi F (2013). The fluorescence spectroscopic studies on the interaction of novel aminophosphinic acids with bovine serum albumin. ‎J. Lumin.

[81] Senthilnathan D, Solomon RV, Venuvanalingam P (2012). Evidence for the powerful catalytic ability of imidozirconocene complex from its epoxide ring cleavage reactions–A DFT mechanistic view. J. Chem. Sci..

[82] Russo TV, Martin RL, Hay PJ (1995). Effective core potentials for DFT calculations. J. Phys. Chem..

[83] Hay PJ, Wadt WR (1985). Ab initio effective core potentials for molecular calculations. Potentials for K to Au including the outermost core orbitals. J. Chem. Phys..

[84] Wadt WR, Hay PJ (1985). Ab initio effective core potentials for molecular calculations. Potentials for main group elements Na to Bi. J. Chem. Phys..

[85] Becke AD (1993). Density‐functional thermochemistry. III. The role of exact exchange. J. Chem. Phys..

[86] Lee C, Yang W, Parr RG (1988). Development of the Colle-Salvetti correlation-energy formula into a functional of the electron density. Phys. Rev. B.

[87] Morris GM (1998). Automated docking using a Lamarckian genetic algorithm and an empirical binding free energy function. J Comput Chem..

[88] Lahiri D (2008). Anaerobic photocleavage of DNA in red light by dicopper (II) complexes of 3,3′-dithiodipropionic acid. Inorg. Chem..

[89] Lahiri D (2011). Remarkable photocytotoxicity in hypoxic HeLa cells by a dipyridophenazine copper (II) Schiff base thiolate. J. Inorg. Biochem..

[90] Bujacz A (2012). Structures of bovine, equine and leporine serum albumin. Acta Crystallogr. D. Biol. Crystallogr..

[91] Mosmann T (1983). Rapid colorimetric assay for cellular growth and survival: application to proliferation and cytotoxicity assays. Journal of immunological methods.

[92] Spector, D., Goldman, R. & Leinwand, L. (Cold Spring Harbor Laboratory Press, Cold Spring Harbor, NY, 1998).

[93] Kumar RS (2008). Synthesis, DNA binding and antitumor activities of some novel polymer–cobalt (III) complexes containing 1, 10-phenanthroline ligand. Polyhedron.

[94] Blagosklonny MV, El‐Deiry WS (1996). *In vitro* evaluation of a p53‐expressing adenovirus as an anti‐cancer drug. International journal of cancer.

[95] Kue CS, Tan KY, LaM ML, Lee HB (2015). Chick embryo chorioallantoic membrane (CAM): an alternative predictive model in acute toxicological studies for anti-cancer drugs. Exp. Anim..

[96] IACUC Policy on Protocol Approval for Use of Chicken Embryos and Eggs. 2001. An Association of New England Medical Center and Tufts.

[97] National Institute of Health1991. The Public Health Service Responds to Commonly asked Questions.” ILaR news 33.4: 68–70. Office of Laboratory Animal Welfare, http://grants.nih.gov/grants/olaw/references/ilar91.htm.

