# Supplementary material for: Biomolecular Interaction, Anti-Cancer and Anti-Angiogenic Properties of Cobalt(III) Schiff Base Complexes
Source: Sci Rep. 2019 Feb 25;9:2721. doi: 10.1038/s41598-019-39179-1 (PMC6389928; doi:10.1038/s41598-019-39179-1)
Supplement: Supplementary file 1 — Supplementary information [file 41598_2019_39179_MOESM1_ESM.doc]

**Supplementary information**

**BIOMOLECULAR INTERACTION, ANTI-CANCER AND ANTI-ANGIOGENIC PROPERTIES OF COBALT(III) SCHIFF BASE COMPLEXES**

Subramanian Ambika^1 Ϯ^, Yesaiyan Manojkumar^1 Ϯ^, Sankaralingam Arunachalam^1^*, Gowdhami Balakrishnan ^2#^, Kishore Kumar Meenakshi Sundaram ^3^, Rajadurai Vijay Solomon ^4^, Ponnambalam Venuvanalingam ^1^, Mohammad Abdulkader Akbarsha^2§^ , Muthuraman Sundararaman ^5^

*Corresponding author. E-mail address: [arunasurf@yahoo.com](mailto:arunasurf@yahoo.com)

**Figure S1**. FT-IR spectra of cobalt(III) complexes **1** and **2**

**Figure S2. ^1^**H and ^13^C –NMR spectra of complex **1** in CDCl_3_

**Figure S3. ^1^**H and ^13^C –NMR spectra of complex **2** in CDCl_3_

**Figure S4.**  ESI-MS spectra of complexes **1** and **2** in methanol

**Figure S5**. Effect of increasing amounts of EB and complexes **1** and **2** on the relative viscosity of DNA at 30.0 ± 0.2 °C

**Figure S6**. The optimized geometries of complexes **1** and **2** at B3LYP/LANL2DZ level of theory.

**Figure S7** Inhibitory effects of complexes **1** and **2** on A549 cells treated with different concentrations for 24 h. (Data are expressed in Mean ± SD of three experiments each with two replicates)

**Table S1** The selected bond parameters of Co(III) Schiff complexes **1** and **2** obtained at B3LYP level calculations with basis set LANL2DZ for Co atom while 6-31g(d,p) for C, H, O & N atoms. The bond lengths are in Å while bond angles and dihedral angles are in ˚

| **Bond Parameter** | **Complex 1** | **Complex 2** |
| --- | --- | --- |
| Co1-O2 | 1.898 | 1.895 |
| Co1-O3 | 1.898 | 1.893 |
| Co1-N4 | 1.927 | 1.929 |
| Co1-N5 | 1.928 | 1.931 |
| Co1-N6 | 2.006 | 2.001 |
| Co1-N9 | 2.007 | 2.000 |
| C21-C22 | 1.428 | 1.421 |
| C26-C35 | 1.438 | 1.438 |
| C13-H14 | 1.085 | 1.085 |
| C36-H114 | 1.095 | 1.090 |
| O2-Co1-O3 | 89.20 | 85.18 |
| O2-Co1-N4 | 92.59 | 93.81 |
| N4-Co1-N5 | 84.67 | 84.87 |
| N6-Co1-N9 | 168.53 | 170.02 |
| C26-O3-Co1-N4 | 29.55 | 89.80 |
| O3-Co1-O2-C12 | 164.03 | 171.91 |

**Table S2** Molecular orbital energy (eV) level values of complexes **1** and **2** from DFT calculations at B3LYP level with basis set LANL2DZ for Co atom while 6-31g(d,p) for C, H, O & N atoms.

| **Complex** | **HOMO -3** | **HOMO**  **-2** | **HOMO**  **-1** | **HOMO** | **LUMO** | **LUMO +1** | **LUMO+2** | **LUMO +3** | **HOMO-LUMO gap** |
| --- | --- | --- | --- | --- | --- | --- | --- | --- | --- |
| **1** | -9.235 | -9.228 | -8.388 | -8.249 | -4.782 | -4.571 | -4.422 | -4.401 | 3.466 |
| **2** | -9.226 | -8.970 | -8.321 | -8.227 | -5.008 | -4.737 | -4.667 | -4.462 | 3.219 |

**THEORETICAL ABSORPTION SPECTRA CALCULATIONS**

The B3LYP/(LANL2DZ(Co),6-31g(d,p) for other atoms) optimized geometries are used for TDDFT calculations to obtain absorption spectra in DMSO solvent. The effect of solvent is included from Polarizable Continuum Model (PCM) implemented in Gaussian09. It is important to note that PCM calculations have been identified as the most successful model for describing solvent effect in TDDFT calculations. Therefore, TDDFT calculations for the fifty lowest lying excited states were computed on these metal complexes and the results are summarized in table S3. Table S3 lists the absorption maxima, electronic transition energies (ΔE in eV), oscillator strength (*f*_0_) and transition assignments along with their percentage contributions. From the table, it is clear that the complex 1 is found to show three major peaks at 487 nm, 372nm and 269 nm. The oscillator strength for the 487 nm peak is 0.433 and is arising due to 36% and 35% contributions from HOMO🡪LUMO+3 and HOMO-1🡪LUMO+2 respectively. HOMO-2🡪LUMO+2 transitions are responsible for the peak at 269 nm with transition energy of 4.61eV. On the other hand, complex 2 also gives three major peaks at 477 nm, 368 nm and 267 nm. Peaks 477 nm and 368 nm are arising from HOMO🡪LUMO+6 and HOMO🡪LUMO+10 respectively. Oscillator strength of 0.24 is obtained for the occurance of 267 nm peak. Analyzing the frontier molecular orbitals suggest that the peaks around 470 nm for these metal complexes are due to LMCT and π- π* transitions. These results indicate that the computed absorption spectra are in total agreement with the experimental studies.

**Table S3:** Absorption maxima (λ_max_), electronic transition energies (∆E), oscillator strength (*f*_0_), Transition assignments of complexes 1 & 2 in DMSO solvent calculated at B3LYP level using TDDFT calculations. Experimental values are given in [ ] for comparison.

| **Metal Complex** | **λ_max (_nm)** | **eV** | *f*_0_ | **Transition assignments** |
| --- | --- | --- | --- | --- |
| **Complex 1** | 487 [ 494] | 2.54 | 0.4330 | HOMO 🡪 LUMO+3 (36%)  HOMO-1🡪LUMO+2 (35%) |
| 372 [389 ] | 3.33 | 0.2120 | HOMO-2 🡪 LUMO (42%) |
| 269 [263 ] | 4.61 | 0.3804 | HOMO-2à LUMO+2 (94%) |
| **Complex 2** | 477 [471 ] | 2.59 | 0.0886 | HOMOà LUMO+6 (65%) |
| 368 [ 367] | 3.37 | 0.1177 | HOMOàLUMO+10 (58%) |
| 267 [ 261] | 4.64 | 0.2400 | HOMO-7à LUMO (+28%) |

**3D coordinates files of the docked complexes**

**Complex 1-BSA**

HEADER 4F5S

REMARK 6

REMARK 6 PDB written by MVD (www.molegro.com)

ATOM 1 N ASP A 1 -15.623 24.749 73.372 1.00118.56

ATOM 2 CA ASP A 1 -15.370 25.428 74.669 1.00117.08

ATOM 3 C ASP A 1 -13.903 25.174 75.098 1.00112.71

ATOM 4 O ASP A 1 -13.585 24.957 76.290 1.00110.24

ATOM 5 CB ASP A 1 -16.376 24.925 75.715 1.00118.59

ATOM 6 CG ASP A 1 -17.009 26.061 76.526 1.00121.01

ATOM 7 OD1 ASP A 1 -16.267 26.830 77.200 1.00116.55

ATOM 8 OD2 ASP A 1 -18.266 26.170 76.489 1.00125.13

ATOM 9 H ASP A 1 -16.552 24.810 72.931 1.00 0.00

ATOM 10 H ASP A 1 -15.508 26.505 74.573 1.00 0.00

ATOM 11 H ASP A 1 -15.841 24.277 76.410 1.00 0.00

ATOM 12 H ASP A 1 -17.177 24.409 75.186 1.00 0.00

ATOM 13 N THR A 2 -13.008 25.247 74.112 1.00111.20

ATOM 14 CA THR A 2 -11.626 24.800 74.300 1.00107.19

ATOM 15 C THR A 2 -10.523 25.925 74.259 1.00105.12

ATOM 16 O THR A 2 -10.040 26.305 73.182 1.00106.09

ATOM 17 CB THR A 2 -11.342 23.495 73.419 1.00106.44

ATOM 18 OG1 THR A 2 -10.025 22.984 73.671 1.00102.94

ATOM 19 CG2 THR A 2 -11.556 23.759 71.916 1.00108.74

ATOM 20 H THR A 2 -13.295 25.626 73.198 1.00 0.00

ATOM 21 H THR A 2 -11.528 24.499 75.343 1.00 0.00

ATOM 22 H THR A 2 -12.065 22.736 73.719 1.00 0.00

ATOM 23 H THR A 2 -9.951 22.831 74.687 1.00 0.00

ATOM 24 H THR A 2 -11.343 22.826 71.395 1.00 0.00

ATOM 25 H THR A 2 -12.595 24.065 71.791 1.00 0.00

ATOM 26 H THR A 2 -10.863 24.550 71.630 1.00 0.00

ATOM 27 N HIS A 3 -10.070 26.341 75.445 1.00102.15

ATOM 28 CA HIS A 3 -8.946 27.260 75.608 1.00 98.74

ATOM 29 C HIS A 3 -7.685 26.424 75.542 1.00 94.37

ATOM 30 O HIS A 3 -7.196 25.934 76.541 1.00 92.82

ATOM 31 CB HIS A 3 -9.005 27.880 76.992 1.00 98.74

ATOM 32 CG HIS A 3 -9.754 29.171 77.057 1.00102.40

ATOM 33 ND1 HIS A 3 -10.339 29.627 78.216 1.00103.60

ATOM 34 CD2 HIS A 3 -10.008 30.106 76.113 1.00106.09

ATOM 35 CE1 HIS A 3 -10.929 30.783 77.981 1.00107.20

ATOM 36 NE2 HIS A 3 -10.744 31.095 76.712 1.00108.39

ATOM 37 H HIS A 3 -10.540 25.994 76.293 1.00 0.00

ATOM 38 H HIS A 3 -8.971 28.038 74.845 1.00 0.00

ATOM 39 H HIS A 3 -7.982 28.087 77.308 1.00 0.00

ATOM 40 H HIS A 3 -9.540 27.180 77.634 1.00 0.00

ATOM 41 H HIS A 3 -10.319 29.142 79.124 1.00 0.00

ATOM 42 H HIS A 3 -9.688 30.078 75.072 1.00 0.00

ATOM 43 H HIS A 3 -11.476 31.379 78.711 1.00 0.00

ATOM 44 N LYS A 4 -7.173 26.266 74.339 1.00 92.44

ATOM 45 CA LYS A 4 -6.331 25.150 73.979 1.00 87.69

ATOM 46 C LYS A 4 -4.946 24.874 74.548 1.00 82.52

ATOM 47 O LYS A 4 -4.629 23.727 74.761 1.00 81.73

ATOM 48 CB LYS A 4 -6.311 24.973 72.459 1.00 89.64

ATOM 49 CG LYS A 4 -6.256 26.240 71.668 1.00 91.49

ATOM 50 CD LYS A 4 -5.656 25.985 70.308 1.00 91.54

ATOM 51 CE LYS A 4 -6.454 24.963 69.542 1.00 91.53

ATOM 52 NZ LYS A 4 -5.669 24.389 68.438 1.00 90.74

ATOM 53 H LYS A 4 -7.384 26.973 73.620 1.00 0.00

ATOM 54 H LYS A 4 -6.866 24.400 74.562 1.00 0.00

ATOM 55 H LYS A 4 -7.233 24.467 72.173 1.00 0.00

ATOM 56 H LYS A 4 -5.392 24.438 72.220 1.00 0.00

ATOM 57 H LYS A 4 -5.633 26.957 72.202 1.00 0.00

ATOM 58 H LYS A 4 -7.267 26.629 71.550 1.00 0.00

ATOM 59 H LYS A 4 -4.642 25.607 70.440 1.00 0.00

ATOM 60 H LYS A 4 -5.659 26.919 69.746 1.00 0.00

ATOM 61 H LYS A 4 -7.345 25.434 69.126 1.00 0.00

ATOM 62 H LYS A 4 -6.715 24.149 70.219 1.00 0.00

ATOM 63 H LYS A 4 -6.274 23.704 67.963 1.00 0.00

ATOM 64 H LYS A 4 -5.410 25.168 67.816 1.00 0.00

ATOM 65 H LYS A 4 -4.841 23.943 68.858 1.00 0.00

ATOM 66 N SER A 5 -4.087 25.856 74.740 1.00 78.29

ATOM 67 CA SER A 5 -2.664 25.468 74.908 1.00 72.75

ATOM 68 C SER A 5 -2.098 25.822 76.250 1.00 69.55

ATOM 69 O SER A 5 -1.642 26.946 76.449 1.00 69.40

ATOM 70 CB SER A 5 -1.784 26.091 73.820 1.00 73.26

ATOM 71 OG SER A 5 -0.402 26.118 74.169 1.00 67.99

ATOM 72 H SER A 5 -4.388 26.841 74.772 1.00 0.00

ATOM 73 H SER A 5 -2.656 24.381 74.822 1.00 0.00

ATOM 74 H SER A 5 -2.091 27.116 73.613 1.00 0.00

ATOM 75 H SER A 5 -1.838 25.445 72.944 1.00 0.00

ATOM 76 H SER A 5 0.099 26.551 73.380 1.00 0.00

ATOM 77 N GLU A 6 -2.103 24.862 77.163 1.00 66.64

ATOM 78 CA GLU A 6 -1.686 25.107 78.517 1.00 63.88

ATOM 79 C GLU A 6 -0.322 25.767 78.695 1.00 61.32

ATOM 80 O GLU A 6 -0.208 26.645 79.547 1.00 59.89

ATOM 81 CB GLU A 6 -1.764 23.843 79.347 1.00 64.27

ATOM 82 CG GLU A 6 -3.109 23.682 80.152 1.00 69.38

ATOM 83 CD GLU A 6 -3.451 24.884 81.031 1.00 73.87

ATOM 84 OE1 GLU A 6 -2.755 25.100 82.088 1.00 74.97

ATOM 85 OE2 GLU A 6 -4.400 25.604 80.640 1.00 73.15

ATOM 86 H GLU A 6 -2.413 23.917 76.896 1.00 0.00

ATOM 87 H GLU A 6 -2.401 25.848 78.875 1.00 0.00

ATOM 88 H GLU A 6 -0.960 23.924 80.078 1.00 0.00

ATOM 89 H GLU A 6 -1.660 23.002 78.661 1.00 0.00

ATOM 90 H GLU A 6 -3.021 22.816 80.808 1.00 0.00

ATOM 91 H GLU A 6 -3.915 23.603 79.423 1.00 0.00

ATOM 92 N ILE A 7 0.700 25.428 77.910 1.00 59.67

ATOM 93 CA ILE A 7 1.939 26.190 78.085 1.00 59.14

ATOM 94 C ILE A 7 1.695 27.689 77.852 1.00 60.04

ATOM 95 O ILE A 7 2.061 28.524 78.680 1.00 60.10

ATOM 96 CB ILE A 7 3.203 25.592 77.379 1.00 58.87

ATOM 97 CG1 ILE A 7 4.464 26.179 77.965 1.00 57.34

ATOM 98 CG2 ILE A 7 3.222 25.845 75.898 1.00 62.48

ATOM 99 CD1 ILE A 7 5.708 25.335 77.683 1.00 60.77

ATOM 100 H ILE A 7 0.623 24.670 77.217 1.00 0.00

ATOM 101 H ILE A 7 2.225 26.083 79.131 1.00 0.00

ATOM 102 H ILE A 7 3.156 24.516 77.547 1.00 0.00

ATOM 103 H ILE A 7 4.328 26.216 79.046 1.00 0.00

ATOM 104 H ILE A 7 4.610 27.162 77.517 1.00 0.00

ATOM 105 H ILE A 7 4.134 25.392 75.509 1.00 0.00

ATOM 106 H ILE A 7 2.329 25.378 75.483 1.00 0.00

ATOM 107 H ILE A 7 3.215 26.926 75.759 1.00 0.00

ATOM 108 H ILE A 7 6.552 25.846 78.147 1.00 0.00

ATOM 109 H ILE A 7 5.535 24.354 78.126 1.00 0.00

ATOM 110 H ILE A 7 5.815 25.277 76.600 1.00 0.00

ATOM 111 N ALA A 8 0.970 28.010 76.797 1.00 61.66

ATOM 112 CA ALA A 8 0.673 29.398 76.450 1.00 63.27

ATOM 113 C ALA A 8 -0.231 30.141 77.448 1.00 64.12

ATOM 114 O ALA A 8 0.031 31.286 77.721 1.00 65.98

ATOM 115 CB ALA A 8 0.143 29.490 75.046 1.00 65.69

ATOM 116 H ALA A 8 0.601 27.257 76.198 1.00 0.00

ATOM 117 H ALA A 8 1.626 29.923 76.511 1.00 0.00

ATOM 118 H ALA A 8 -0.058 30.543 74.849 1.00 0.00

ATOM 119 H ALA A 8 0.916 29.095 74.387 1.00 0.00

ATOM 120 H ALA A 8 -0.766 28.890 75.009 1.00 0.00

ATOM 121 N HIS A 9 -1.254 29.524 78.019 1.00 64.02

ATOM 122 CA HIS A 9 -2.014 30.155 79.123 1.00 65.23

ATOM 123 C HIS A 9 -1.122 30.601 80.310 1.00 64.16

ATOM 124 O HIS A 9 -1.174 31.764 80.778 1.00 65.15

ATOM 125 CB HIS A 9 -3.112 29.222 79.612 1.00 65.74

ATOM 126 CG HIS A 9 -3.962 29.789 80.707 1.00 71.93

ATOM 127 ND1 HIS A 9 -4.206 29.113 81.889 1.00 73.61

ATOM 128 CD2 HIS A 9 -4.618 30.974 80.811 1.00 77.99

ATOM 129 CE1 HIS A 9 -4.961 29.862 82.678 1.00 75.30

ATOM 130 NE2 HIS A 9 -5.227 30.994 82.045 1.00 79.56

ATOM 131 H HIS A 9 -1.527 28.587 77.689 1.00 0.00

ATOM 132 H HIS A 9 -2.457 31.062 78.711 1.00 0.00

ATOM 133 H HIS A 9 -2.635 28.326 80.009 1.00 0.00

ATOM 134 H HIS A 9 -3.777 29.041 78.768 1.00 0.00

ATOM 135 H HIS A 9 -3.855 28.173 82.120 1.00 0.00

ATOM 136 H HIS A 9 -4.655 31.761 80.058 1.00 0.00

ATOM 137 H HIS A 9 -5.304 29.593 83.677 1.00 0.00

ATOM 138 N ARG A 10 -0.276 29.693 80.761 1.00 61.75

ATOM 139 CA ARG A 10 0.669 29.986 81.837 1.00 60.50

ATOM 140 C ARG A 10 1.776 31.015 81.512 1.00 61.01

ATOM 141 O ARG A 10 2.119 31.837 82.345 1.00 61.73

ATOM 142 CB ARG A 10 1.338 28.689 82.337 1.00 58.13

ATOM 143 CG ARG A 10 0.415 27.685 82.936 1.00 55.21

ATOM 144 CD ARG A 10 -0.644 28.285 83.779 1.00 56.13

ATOM 145 NE ARG A 10 -1.625 27.238 84.019 1.00 60.06

ATOM 146 CZ ARG A 10 -2.799 27.402 84.610 1.00 59.66

ATOM 147 NH1 ARG A 10 -3.157 28.577 85.062 1.00 56.78

ATOM 148 NH2 ARG A 10 -3.610 26.349 84.775 1.00 62.80

ATOM 149 H ARG A 10 -0.282 28.752 80.343 1.00 0.00

ATOM 150 H ARG A 10 0.048 30.449 82.604 1.00 0.00

ATOM 151 H ARG A 10 2.055 28.955 83.114 1.00 0.00

ATOM 152 H ARG A 10 1.761 28.199 81.460 1.00 0.00

ATOM 153 H ARG A 10 1.007 27.028 83.573 1.00 0.00

ATOM 154 H ARG A 10 -0.081 27.165 82.116 1.00 0.00

ATOM 155 H ARG A 10 -1.104 29.146 83.295 1.00 0.00

ATOM 156 H ARG A 10 -0.234 28.642 84.724 1.00 0.00

ATOM 157 H ARG A 10 -1.386 26.288 83.702 1.00 0.00

ATOM 158 H ARG A 10 -4.072 28.696 85.520 1.00 0.00

ATOM 159 H ARG A 10 -2.524 29.383 84.959 1.00 0.00

ATOM 160 H ARG A 10 -4.524 26.470 85.234 1.00 0.00

ATOM 161 H ARG A 10 -3.320 25.418 84.443 1.00 0.00

ATOM 162 N PHE A 11 2.362 30.962 80.326 1.00 62.05

ATOM 163 CA PHE A 11 3.451 31.877 80.047 1.00 62.95

ATOM 164 C PHE A 11 2.939 33.334 80.063 1.00 64.95

ATOM 165 O PHE A 11 3.574 34.191 80.634 1.00 64.88

ATOM 166 CB PHE A 11 4.106 31.554 78.713 1.00 63.86

ATOM 167 CG PHE A 11 5.267 32.407 78.436 1.00 65.29

ATOM 168 CD1 PHE A 11 5.131 33.551 77.654 1.00 67.72

ATOM 169 CD2 PHE A 11 6.505 32.124 79.021 1.00 64.78

ATOM 170 CE1 PHE A 11 6.228 34.416 77.419 1.00 68.46

ATOM 171 CE2 PHE A 11 7.620 32.963 78.768 1.00 67.77

ATOM 172 CZ PHE A 11 7.470 34.123 77.967 1.00 67.93

ATOM 173 H PHE A 11 2.050 30.284 79.616 1.00 0.00

ATOM 174 H PHE A 11 4.205 31.762 80.826 1.00 0.00

ATOM 175 H PHE A 11 3.384 31.698 77.909 1.00 0.00

ATOM 176 H PHE A 11 4.478 30.531 78.761 1.00 0.00

ATOM 177 H PHE A 11 4.162 33.786 77.214 1.00 0.00

ATOM 178 H PHE A 11 6.613 31.257 79.673 1.00 0.00

ATOM 179 H PHE A 11 6.097 35.310 76.809 1.00 0.00

ATOM 180 H PHE A 11 8.594 32.716 79.190 1.00 0.00

ATOM 181 H PHE A 11 8.321 34.779 77.783 1.00 0.00

ATOM 182 N LYS A 12 1.772 33.565 79.461 1.00 66.30

ATOM 183 CA LYS A 12 1.062 34.858 79.463 1.00 69.53

ATOM 184 C LYS A 12 0.750 35.386 80.852 1.00 68.43

ATOM 185 O LYS A 12 0.897 36.583 81.121 1.00 69.62

ATOM 186 CB LYS A 12 -0.279 34.781 78.709 1.00 70.80

ATOM 187 CG LYS A 12 -0.148 34.921 77.246 1.00 77.18

ATOM 188 CD LYS A 12 -1.497 34.712 76.473 1.00 84.28

ATOM 189 CE LYS A 12 -1.620 33.270 75.955 1.00 85.58

ATOM 190 NZ LYS A 12 -3.016 32.681 75.947 1.00 87.55

ATOM 191 H LYS A 12 1.333 32.782 78.957 1.00 0.00

ATOM 192 H LYS A 12 1.758 35.535 78.967 1.00 0.00

ATOM 193 H LYS A 12 -0.931 35.583 79.054 1.00 0.00

ATOM 194 H LYS A 12 -0.686 33.783 78.872 1.00 0.00

ATOM 195 H LYS A 12 0.526 34.119 76.946 1.00 0.00

ATOM 196 H LYS A 12 0.246 35.918 77.049 1.00 0.00

ATOM 197 H LYS A 12 -1.539 35.393 75.623 1.00 0.00

ATOM 198 H LYS A 12 -2.324 34.892 77.160 1.00 0.00

ATOM 199 H LYS A 12 -1.028 32.643 76.622 1.00 0.00

ATOM 200 H LYS A 12 -1.305 33.293 74.912 1.00 0.00

ATOM 201 H LYS A 12 -2.936 31.723 75.578 1.00 0.00

ATOM 202 H LYS A 12 -3.352 32.690 76.921 1.00 0.00

ATOM 203 H LYS A 12 -3.592 33.279 75.337 1.00 0.00

ATOM 204 N ASP A 13 0.275 34.488 81.708 1.00 66.68

ATOM 205 CA ASP A 13 -0.289 34.875 82.992 1.00 65.91

ATOM 206 C ASP A 13 0.806 35.191 83.990 1.00 63.48

ATOM 207 O ASP A 13 0.688 36.090 84.766 1.00 63.71

ATOM 208 CB ASP A 13 -1.235 33.791 83.513 1.00 64.96

ATOM 209 CG ASP A 13 -2.593 33.795 82.803 1.00 70.03

ATOM 210 OD1 ASP A 13 -3.444 33.006 83.230 1.00 75.72

ATOM 211 OD2 ASP A 13 -2.835 34.536 81.810 1.00 73.61

ATOM 212 H ASP A 13 0.307 33.490 81.456 1.00 0.00

ATOM 213 H ASP A 13 -0.874 35.784 82.853 1.00 0.00

ATOM 214 H ASP A 13 -1.411 33.984 84.571 1.00 0.00

ATOM 215 H ASP A 13 -0.766 32.824 83.332 1.00 0.00

ATOM 216 N LEU A 14 1.892 34.451 83.944 1.00 62.03

ATOM 217 CA LEU A 14 2.959 34.693 84.880 1.00 61.00

ATOM 218 C LEU A 14 3.926 35.727 84.346 1.00 61.39

ATOM 219 O LEU A 14 4.524 36.456 85.112 1.00 62.87

ATOM 220 CB LEU A 14 3.679 33.393 85.155 1.00 58.43

ATOM 221 CG LEU A 14 2.896 32.270 85.839 1.00 59.79

ATOM 222 CD1 LEU A 14 3.703 30.994 85.706 1.00 59.97

ATOM 223 CD2 LEU A 14 2.536 32.512 87.309 1.00 56.42

ATOM 224 H LEU A 14 1.979 33.702 83.242 1.00 0.00

ATOM 225 H LEU A 14 2.537 35.082 85.806 1.00 0.00

ATOM 226 H LEU A 14 4.475 33.656 85.851 1.00 0.00

ATOM 227 H LEU A 14 3.963 33.007 84.176 1.00 0.00

ATOM 228 H LEU A 14 1.932 32.211 85.333 1.00 0.00

ATOM 229 H LEU A 14 3.134 30.204 86.197 1.00 0.00

ATOM 230 H LEU A 14 3.824 30.803 84.640 1.00 0.00

ATOM 231 H LEU A 14 4.660 31.169 86.198 1.00 0.00

ATOM 232 H LEU A 14 1.987 31.635 87.651 1.00 0.00

ATOM 233 H LEU A 14 3.474 32.640 87.850 1.00 0.00

ATOM 234 H LEU A 14 1.923 33.412 87.343 1.00 0.00

ATOM 235 N GLY A 15 4.067 35.796 83.031 1.00 62.37

ATOM 236 CA GLY A 15 5.107 36.596 82.396 1.00 63.62

ATOM 237 C GLY A 15 6.377 35.792 82.465 1.00 62.41

ATOM 238 O GLY A 15 6.430 34.835 83.232 1.00 60.97

ATOM 239 H GLY A 15 3.417 35.264 82.434 1.00 0.00

ATOM 240 H GLY A 15 5.218 37.541 82.928 1.00 0.00

ATOM 241 H GLY A 15 4.833 36.843 81.370 1.00 0.00

ATOM 242 N GLU A 16 7.394 36.222 81.732 1.00 64.36

ATOM 243 CA GLU A 16 8.570 35.431 81.417 1.00 66.34

ATOM 244 C GLU A 16 9.501 35.170 82.550 1.00 66.50

ATOM 245 O GLU A 16 10.031 34.047 82.660 1.00 65.91

ATOM 246 CB GLU A 16 9.410 36.104 80.357 1.00 69.38

ATOM 247 CG GLU A 16 10.421 35.138 79.750 1.00 72.89

ATOM 248 CD GLU A 16 11.655 35.819 79.163 1.00 81.67

ATOM 249 OE1 GLU A 16 12.249 36.693 79.860 1.00 86.54

ATOM 250 OE2 GLU A 16 12.045 35.459 78.028 1.00 80.19

ATOM 251 H GLU A 16 7.347 37.182 81.361 1.00 0.00

ATOM 252 H GLU A 16 8.138 34.483 81.096 1.00 0.00

ATOM 253 H GLU A 16 9.954 36.924 80.826 1.00 0.00

ATOM 254 H GLU A 16 8.749 36.463 79.569 1.00 0.00

ATOM 255 H GLU A 16 9.920 34.618 78.934 1.00 0.00

ATOM 256 H GLU A 16 10.762 34.482 80.551 1.00 0.00

ATOM 257 N GLU A 17 9.695 36.179 83.403 1.00 68.59

ATOM 258 CA GLU A 17 10.637 36.045 84.493 1.00 68.82

ATOM 259 C GLU A 17 10.128 35.033 85.457 1.00 66.59

ATOM 260 O GLU A 17 10.853 34.179 85.904 1.00 67.11

ATOM 261 CB GLU A 17 10.946 37.382 85.173 1.00 70.09

ATOM 262 CG GLU A 17 11.603 38.457 84.243 1.00 76.12

ATOM 263 CD GLU A 17 12.579 37.884 83.185 1.00 81.70

ATOM 264 OE1 GLU A 17 13.556 37.153 83.528 1.00 81.91

ATOM 265 OE2 GLU A 17 12.353 38.171 81.987 1.00 86.23

ATOM 266 H GLU A 17 9.171 37.058 83.284 1.00 0.00

ATOM 267 H GLU A 17 11.588 35.702 84.086 1.00 0.00

ATOM 268 H GLU A 17 11.669 37.158 85.957 1.00 0.00

ATOM 269 H GLU A 17 9.996 37.783 85.527 1.00 0.00

ATOM 270 H GLU A 17 12.181 39.129 84.878 1.00 0.00

ATOM 271 H GLU A 17 10.795 38.937 83.691 1.00 0.00

ATOM 272 N HIS A 18 8.852 35.095 85.767 1.00 66.40

ATOM 273 CA HIS A 18 8.318 34.115 86.695 1.00 63.34

ATOM 274 C HIS A 18 8.183 32.768 86.066 1.00 62.24

ATOM 275 O HIS A 18 8.427 31.759 86.735 1.00 61.70

ATOM 276 CB HIS A 18 7.039 34.607 87.329 1.00 62.26

ATOM 277 CG HIS A 18 7.307 35.714 88.264 1.00 62.99

ATOM 278 ND1 HIS A 18 6.323 36.411 88.918 1.00 64.84

ATOM 279 CD2 HIS A 18 8.483 36.255 88.650 1.00 63.17

ATOM 280 CE1 HIS A 18 6.885 37.326 89.687 1.00 66.35

ATOM 281 NE2 HIS A 18 8.196 37.249 89.544 1.00 65.56

ATOM 282 H HIS A 18 8.246 35.822 85.361 1.00 0.00

ATOM 283 H HIS A 18 9.037 33.990 87.504 1.00 0.00

ATOM 284 H HIS A 18 6.563 33.794 87.877 1.00 0.00

ATOM 285 H HIS A 18 6.383 34.985 86.545 1.00 0.00

ATOM 286 H HIS A 18 5.310 36.247 88.825 1.00 0.00

ATOM 287 H HIS A 18 9.475 35.955 88.312 1.00 0.00

ATOM 288 H HIS A 18 6.356 38.027 90.332 1.00 0.00

ATOM 289 N PHE A 19 7.837 32.743 84.780 1.00 62.04

ATOM 290 CA PHE A 19 7.820 31.467 84.074 1.00 60.30

ATOM 291 C PHE A 19 9.197 30.814 84.196 1.00 59.72

ATOM 292 O PHE A 19 9.296 29.701 84.616 1.00 57.02

ATOM 293 CB PHE A 19 7.386 31.662 82.633 1.00 60.90

ATOM 294 CG PHE A 19 7.376 30.409 81.820 1.00 59.40

ATOM 295 CD1 PHE A 19 8.530 30.012 81.109 1.00 58.48

ATOM 296 CD2 PHE A 19 6.209 29.646 81.720 1.00 53.34

ATOM 297 CE1 PHE A 19 8.533 28.863 80.343 1.00 55.01

ATOM 298 CE2 PHE A 19 6.182 28.519 80.953 1.00 51.65

ATOM 299 CZ PHE A 19 7.364 28.099 80.267 1.00 55.26

ATOM 300 H PHE A 19 7.585 33.613 84.291 1.00 0.00

ATOM 301 H PHE A 19 7.089 30.795 84.524 1.00 0.00

ATOM 302 H PHE A 19 8.093 32.345 82.162 1.00 0.00

ATOM 303 H PHE A 19 6.358 32.025 82.654 1.00 0.00

ATOM 304 H PHE A 19 9.432 30.622 81.166 1.00 0.00

ATOM 305 H PHE A 19 5.313 29.954 82.259 1.00 0.00

ATOM 306 H PHE A 19 9.431 28.557 79.807 1.00 0.00

ATOM 307 H PHE A 19 5.263 27.940 80.863 1.00 0.00

ATOM 308 H PHE A 19 7.354 27.179 79.682 1.00 0.00

ATOM 309 N LYS A 20 10.265 31.542 83.874 1.00 62.03

ATOM 310 CA LYS A 20 11.615 30.964 83.906 1.00 62.18

ATOM 311 C LYS A 20 12.034 30.527 85.303 1.00 61.53

ATOM 312 O LYS A 20 12.503 29.376 85.514 1.00 60.70

ATOM 313 CB LYS A 20 12.622 31.959 83.356 1.00 65.09

ATOM 314 CG LYS A 20 12.734 31.964 81.822 1.00 65.92

ATOM 315 CD LYS A 20 13.678 33.020 81.400 1.00 69.61

ATOM 316 CE LYS A 20 15.115 32.486 81.313 1.00 75.52

ATOM 317 NZ LYS A 20 15.586 32.126 79.894 1.00 73.66

ATOM 318 H LYS A 20 10.142 32.527 83.598 1.00 0.00

ATOM 319 H LYS A 20 11.593 30.071 83.281 1.00 0.00

ATOM 320 H LYS A 20 13.596 31.665 83.748 1.00 0.00

ATOM 321 H LYS A 20 12.295 32.951 83.667 1.00 0.00

ATOM 322 H LYS A 20 11.763 32.139 81.358 1.00 0.00

ATOM 323 H LYS A 20 13.131 31.006 81.485 1.00 0.00

ATOM 324 H LYS A 20 13.645 33.799 82.162 1.00 0.00

ATOM 325 H LYS A 20 13.360 33.399 80.429 1.00 0.00

ATOM 326 H LYS A 20 15.145 31.567 81.899 1.00 0.00

ATOM 327 H LYS A 20 15.769 33.280 81.672 1.00 0.00

ATOM 328 H LYS A 20 16.554 31.785 79.976 1.00 0.00

ATOM 329 H LYS A 20 14.951 31.395 79.542 1.00 0.00

ATOM 330 H LYS A 20 15.529 32.986 79.331 1.00 0.00

ATOM 331 N GLY A 21 11.823 31.421 86.267 1.00 60.41

ATOM 332 CA GLY A 21 11.995 31.073 87.677 1.00 58.20

ATOM 333 C GLY A 21 11.347 29.761 88.084 1.00 56.07

ATOM 334 O GLY A 21 12.012 28.914 88.690 1.00 57.30

ATOM 335 H GLY A 21 11.533 32.377 86.015 1.00 0.00

ATOM 336 H GLY A 21 11.492 31.863 88.235 1.00 0.00

ATOM 337 H GLY A 21 13.067 31.004 87.860 1.00 0.00

ATOM 338 N LEU A 22 10.082 29.557 87.725 1.00 53.52

ATOM 339 CA LEU A 22 9.336 28.408 88.249 1.00 51.96

ATOM 340 C LEU A 22 9.755 27.119 87.552 1.00 51.03

ATOM 341 O LEU A 22 9.742 26.090 88.130 1.00 48.47

ATOM 342 CB LEU A 22 7.826 28.617 88.135 1.00 51.17

ATOM 343 CG LEU A 22 7.234 29.629 89.092 1.00 52.29

ATOM 344 CD1 LEU A 22 5.849 30.062 88.622 1.00 54.73

ATOM 345 CD2 LEU A 22 7.177 29.070 90.485 1.00 49.47

ATOM 346 H LEU A 22 9.623 30.211 87.074 1.00 0.00

ATOM 347 H LEU A 22 9.579 28.319 89.308 1.00 0.00

ATOM 348 H LEU A 22 7.354 27.661 88.363 1.00 0.00

ATOM 349 H LEU A 22 7.641 29.004 87.133 1.00 0.00

ATOM 350 H LEU A 22 7.878 30.508 89.107 1.00 0.00

ATOM 351 H LEU A 22 5.478 30.786 89.348 1.00 0.00

ATOM 352 H LEU A 22 5.972 30.505 87.634 1.00 0.00

ATOM 353 H LEU A 22 5.229 29.166 88.590 1.00 0.00

ATOM 354 H LEU A 22 6.744 29.842 91.121 1.00 0.00

ATOM 355 H LEU A 22 6.550 28.179 90.446 1.00 0.00

ATOM 356 H LEU A 22 8.200 28.833 90.775 1.00 0.00

ATOM 357 N VAL A 23 10.142 27.240 86.294 1.00 53.27

ATOM 358 CA VAL A 23 10.641 26.168 85.497 1.00 54.09

ATOM 359 C VAL A 23 11.998 25.714 86.019 1.00 54.92

ATOM 360 O VAL A 23 12.258 24.531 86.153 1.00 54.46

ATOM 361 CB VAL A 23 10.630 26.586 84.038 1.00 55.24

ATOM 362 CG1 VAL A 23 11.396 25.573 83.125 1.00 58.65

ATOM 363 CG2 VAL A 23 9.196 26.695 83.578 1.00 55.36

ATOM 364 H VAL A 23 10.079 28.172 85.860 1.00 0.00

ATOM 365 H VAL A 23 9.999 25.290 85.569 1.00 0.00

ATOM 366 H VAL A 23 11.141 27.546 83.956 1.00 0.00

ATOM 367 H VAL A 23 11.335 25.954 82.105 1.00 0.00

ATOM 368 H VAL A 23 12.425 25.537 83.483 1.00 0.00

ATOM 369 H VAL A 23 10.897 24.610 83.229 1.00 0.00

ATOM 370 H VAL A 23 9.219 26.996 82.531 1.00 0.00

ATOM 371 H VAL A 23 8.744 25.712 83.707 1.00 0.00

ATOM 372 H VAL A 23 8.717 27.447 84.205 1.00 0.00

ATOM 373 N LEU A 24 12.845 26.656 86.398 1.00 57.29

ATOM 374 CA LEU A 24 14.163 26.273 86.951 1.00 58.32

ATOM 375 C LEU A 24 14.005 25.554 88.296 1.00 57.37

ATOM 376 O LEU A 24 14.736 24.647 88.581 1.00 57.79

ATOM 377 CB LEU A 24 15.051 27.502 87.103 1.00 58.24

ATOM 378 CG LEU A 24 16.330 27.245 87.921 1.00 61.71

ATOM 379 CD1 LEU A 24 17.436 26.388 87.154 1.00 61.47

ATOM 380 CD2 LEU A 24 16.906 28.545 88.629 1.00 58.02

ATOM 381 H LEU A 24 12.589 27.650 86.309 1.00 0.00

ATOM 382 H LEU A 24 14.638 25.583 86.253 1.00 0.00

ATOM 383 H LEU A 24 14.468 28.251 87.638 1.00 0.00

ATOM 384 H LEU A 24 15.352 27.812 86.102 1.00 0.00

ATOM 385 H LEU A 24 16.006 26.599 88.737 1.00 0.00

ATOM 386 H LEU A 24 18.281 26.278 87.833 1.00 0.00

ATOM 387 H LEU A 24 16.983 25.427 86.912 1.00 0.00

ATOM 388 H LEU A 24 17.706 26.946 86.258 1.00 0.00

ATOM 389 H LEU A 24 17.801 28.242 89.172 1.00 0.00

ATOM 390 H LEU A 24 17.134 29.263 87.841 1.00 0.00

ATOM 391 H LEU A 24 16.130 28.913 89.300 1.00 0.00

ATOM 392 N ILE A 25 13.073 26.018 89.135 1.00 56.91

ATOM 393 CA ILE A 25 12.712 25.318 90.376 1.00 55.25

ATOM 394 C ILE A 25 12.202 23.930 90.049 1.00 55.59

ATOM 395 O ILE A 25 12.686 22.948 90.574 1.00 56.94

ATOM 396 CB ILE A 25 11.658 26.089 91.184 1.00 53.56

ATOM 397 CG1 ILE A 25 12.232 27.432 91.656 1.00 54.14

ATOM 398 CG2 ILE A 25 11.194 25.286 92.361 1.00 52.29

ATOM 399 CD1 ILE A 25 11.225 28.490 91.994 1.00 49.01

ATOM 400 H ILE A 25 12.590 26.898 88.903 1.00 0.00

ATOM 401 H ILE A 25 13.609 25.247 90.992 1.00 0.00

ATOM 402 H ILE A 25 10.801 26.275 90.537 1.00 0.00

ATOM 403 H ILE A 25 12.837 27.832 90.842 1.00 0.00

ATOM 404 H ILE A 25 12.762 27.229 92.587 1.00 0.00

ATOM 405 H ILE A 25 10.453 25.889 92.886 1.00 0.00

ATOM 406 H ILE A 25 10.763 24.365 91.968 1.00 0.00

ATOM 407 H ILE A 25 12.070 25.092 92.980 1.00 0.00

ATOM 408 H ILE A 25 11.783 29.372 92.308 1.00 0.00

ATOM 409 H ILE A 25 10.645 28.678 91.091 1.00 0.00

ATOM 410 H ILE A 25 10.605 28.095 92.799 1.00 0.00

ATOM 411 N ALA A 26 11.230 23.808 89.173 1.00 55.68

ATOM 412 CA ALA A 26 10.757 22.466 88.872 1.00 55.91

ATOM 413 C ALA A 26 11.880 21.529 88.394 1.00 56.54

ATOM 414 O ALA A 26 11.989 20.404 88.850 1.00 57.43

ATOM 415 CB ALA A 26 9.550 22.510 87.911 1.00 55.53

ATOM 416 H ALA A 26 10.818 24.634 88.716 1.00 0.00

ATOM 417 H ALA A 26 10.406 22.026 89.806 1.00 0.00

ATOM 418 H ALA A 26 9.249 21.478 87.730 1.00 0.00

ATOM 419 H ALA A 26 8.766 23.079 88.410 1.00 0.00

ATOM 420 H ALA A 26 9.887 23.000 86.998 1.00 0.00

ATOM 421 N PHE A 27 12.790 21.987 87.556 1.00 58.04

ATOM 422 CA PHE A 27 13.859 21.045 87.147 1.00 60.02

ATOM 423 C PHE A 27 14.788 20.742 88.294 1.00 60.44

ATOM 424 O PHE A 27 15.362 19.630 88.366 1.00 61.84

ATOM 425 CB PHE A 27 14.678 21.563 85.954 1.00 61.56

ATOM 426 CG PHE A 27 14.044 21.278 84.648 1.00 63.25

ATOM 427 CD1 PHE A 27 13.733 22.287 83.788 1.00 66.43

ATOM 428 CD2 PHE A 27 13.691 19.982 84.308 1.00 70.25

ATOM 429 CE1 PHE A 27 13.092 22.023 82.556 1.00 70.05

ATOM 430 CE2 PHE A 27 13.053 19.690 83.077 1.00 70.95

ATOM 431 CZ PHE A 27 12.761 20.719 82.206 1.00 70.75

ATOM 432 H PHE A 27 12.759 22.954 87.202 1.00 0.00

ATOM 433 H PHE A 27 13.352 20.131 86.838 1.00 0.00

ATOM 434 H PHE A 27 15.659 21.087 85.956 1.00 0.00

ATOM 435 H PHE A 27 14.730 22.649 86.035 1.00 0.00

ATOM 436 H PHE A 27 13.982 23.314 84.054 1.00 0.00

ATOM 437 H PHE A 27 13.909 19.170 85.002 1.00 0.00

ATOM 438 H PHE A 27 12.857 22.844 81.879 1.00 0.00

ATOM 439 H PHE A 27 12.795 18.662 82.821 1.00 0.00

ATOM 440 H PHE A 27 12.275 20.512 81.252 1.00 0.00

ATOM 441 N SER A 28 14.942 21.747 89.166 1.00 58.45

ATOM 442 CA SER A 28 15.852 21.663 90.287 1.00 59.26

ATOM 443 C SER A 28 15.431 20.657 91.299 1.00 58.72

ATOM 444 O SER A 28 16.262 19.969 91.845 1.00 60.44

ATOM 445 CB SER A 28 16.093 23.029 90.928 1.00 58.29

ATOM 446 OG SER A 28 17.210 23.613 90.222 1.00 61.56

ATOM 447 H SER A 28 14.394 22.609 89.034 1.00 0.00

ATOM 448 H SER A 28 16.801 21.316 89.879 1.00 0.00

ATOM 449 H SER A 28 16.291 22.976 91.999 1.00 0.00

ATOM 450 H SER A 28 15.223 23.675 90.812 1.00 0.00

ATOM 451 H SER A 28 17.384 24.536 90.645 1.00 0.00

ATOM 452 N GLN A 29 14.131 20.546 91.514 1.00 56.85

ATOM 453 CA GLN A 29 13.579 19.593 92.442 1.00 56.67

ATOM 454 C GLN A 29 13.490 18.172 91.854 1.00 57.31

ATOM 455 O GLN A 29 13.509 17.231 92.559 1.00 58.11

ATOM 456 CB GLN A 29 12.202 20.109 92.897 1.00 55.37

ATOM 457 CG GLN A 29 12.269 21.542 93.460 1.00 53.97

ATOM 458 CD GLN A 29 10.980 22.044 94.081 1.00 50.35

ATOM 459 OE1 GLN A 29 9.915 21.861 93.556 1.00 53.58

ATOM 460 NE2 GLN A 29 11.100 22.731 95.180 1.00 50.89

ATOM 461 H GLN A 29 13.489 21.165 90.998 1.00 0.00

ATOM 462 H GLN A 29 14.247 19.506 93.299 1.00 0.00

ATOM 463 H GLN A 29 11.841 19.452 93.688 1.00 0.00

ATOM 464 H GLN A 29 11.541 20.117 92.030 1.00 0.00

ATOM 465 H GLN A 29 12.501 22.210 92.631 1.00 0.00

ATOM 466 H GLN A 29 13.008 21.532 94.261 1.00 0.00

ATOM 467 H GLN A 29 10.262 23.100 95.651 1.00 0.00

ATOM 468 H GLN A 29 12.033 22.906 95.579 1.00 0.00

ATOM 469 N TYR A 30 13.392 18.029 90.547 1.00 58.80

ATOM 470 CA TYR A 30 13.281 16.699 89.927 1.00 59.28

ATOM 471 C TYR A 30 14.637 16.125 89.818 1.00 60.68

ATOM 472 O TYR A 30 14.798 14.925 90.059 1.00 62.67

ATOM 473 CB TYR A 30 12.633 16.775 88.547 1.00 59.13

ATOM 474 CG TYR A 30 11.134 16.888 88.670 1.00 61.16

ATOM 475 CD1 TYR A 30 10.295 15.894 88.160 1.00 62.78

ATOM 476 CD2 TYR A 30 10.553 17.934 89.408 1.00 60.71

ATOM 477 CE1 TYR A 30 8.894 15.985 88.286 1.00 65.87

ATOM 478 CE2 TYR A 30 9.147 18.022 89.565 1.00 63.38

ATOM 479 CZ TYR A 30 8.334 17.047 88.991 1.00 65.40

ATOM 480 OH TYR A 30 6.972 17.124 89.124 1.00 68.45

ATOM 481 H TYR A 30 13.393 18.866 89.947 1.00 0.00

ATOM 482 H TYR A 30 12.645 16.068 90.548 1.00 0.00

ATOM 483 H TYR A 30 12.874 15.874 87.984 1.00 0.00

ATOM 484 H TYR A 30 13.004 17.661 88.031 1.00 0.00

ATOM 485 H TYR A 30 10.732 15.032 87.655 1.00 0.00

ATOM 486 H TYR A 30 11.193 18.688 89.866 1.00 0.00

ATOM 487 H TYR A 30 8.253 15.228 87.834 1.00 0.00

ATOM 488 H TYR A 30 8.706 18.844 90.129 1.00 0.00

ATOM 489 H TYR A 30 6.575 16.309 88.634 1.00 0.00

ATOM 490 N LEU A 31 15.637 16.947 89.514 1.00 60.25

ATOM 491 CA LEU A 31 16.969 16.372 89.364 1.00 62.73

ATOM 492 C LEU A 31 17.996 17.096 90.195 1.00 63.67

ATOM 493 O LEU A 31 18.544 18.111 89.777 1.00 64.34

ATOM 494 CB LEU A 31 17.379 16.358 87.896 1.00 64.47

ATOM 495 CG LEU A 31 16.280 15.968 86.901 1.00 62.68

ATOM 496 CD1 LEU A 31 16.560 16.551 85.547 1.00 65.91

ATOM 497 CD2 LEU A 31 16.194 14.473 86.846 1.00 62.18

ATOM 498 H LEU A 31 15.476 17.957 89.389 1.00 0.00

ATOM 499 H LEU A 31 16.925 15.346 89.730 1.00 0.00

ATOM 500 H LEU A 31 18.159 15.602 87.806 1.00 0.00

ATOM 501 H LEU A 31 17.677 17.377 87.649 1.00 0.00

ATOM 502 H LEU A 31 15.322 16.370 87.229 1.00 0.00

ATOM 503 H LEU A 31 15.747 16.239 84.891 1.00 0.00

ATOM 504 H LEU A 31 16.591 17.634 85.666 1.00 0.00

ATOM 505 H LEU A 31 17.520 16.152 85.221 1.00 0.00

ATOM 506 H LEU A 31 15.407 14.225 86.133 1.00 0.00

ATOM 507 H LEU A 31 17.168 14.110 86.517 1.00 0.00

ATOM 508 H LEU A 31 15.952 14.130 87.852 1.00 0.00

ATOM 509 N GLN A 32 18.292 16.554 91.365 1.00 63.98

ATOM 510 CA GLN A 32 18.887 17.382 92.378 1.00 64.59

ATOM 511 C GLN A 32 20.385 17.273 92.312 1.00 68.46

ATOM 512 O GLN A 32 21.159 18.035 92.942 1.00 70.39

ATOM 513 CB GLN A 32 18.380 16.965 93.744 1.00 63.64

ATOM 514 CG GLN A 32 16.908 16.951 93.855 1.00 60.96

ATOM 515 CD GLN A 32 16.492 16.117 94.997 1.00 63.36

ATOM 516 OE1 GLN A 32 15.731 15.129 94.850 1.00 64.86

ATOM 517 NE2 GLN A 32 16.976 16.482 96.163 1.00 59.78

ATOM 518 H GLN A 32 18.100 15.558 91.546 1.00 0.00

ATOM 519 H GLN A 32 18.608 18.422 92.206 1.00 0.00

ATOM 520 H GLN A 32 18.754 17.674 94.482 1.00 0.00

ATOM 521 H GLN A 32 18.708 15.939 93.913 1.00 0.00

ATOM 522 H GLN A 32 16.484 16.539 92.939 1.00 0.00

ATOM 523 H GLN A 32 16.552 17.970 94.010 1.00 0.00

ATOM 524 H GLN A 32 16.733 15.950 97.011 1.00 0.00

ATOM 525 H GLN A 32 17.599 17.300 96.229 1.00 0.00

ATOM 526 N GLN A 33 20.849 16.346 91.531 1.00 70.02

ATOM 527 CA GLN A 33 22.258 16.239 91.518 1.00 73.61

ATOM 528 C GLN A 33 22.816 16.998 90.359 1.00 74.65

ATOM 529 O GLN A 33 23.997 17.312 90.317 1.00 77.88

ATOM 530 CB GLN A 33 22.619 14.789 91.521 1.00 76.04

ATOM 531 CG GLN A 33 22.010 14.088 92.724 1.00 74.46

ATOM 532 CD GLN A 33 22.371 12.633 92.704 1.00 78.80

ATOM 533 OE1 GLN A 33 23.528 12.294 92.509 1.00 83.62

ATOM 534 NE2 GLN A 33 21.387 11.758 92.856 1.00 77.97

ATOM 535 H GLN A 33 20.239 15.737 90.966 1.00 0.00

ATOM 536 H GLN A 33 22.702 16.688 92.407 1.00 0.00

ATOM 537 H GLN A 33 23.705 14.708 91.578 1.00 0.00

ATOM 538 H GLN A 33 22.247 14.331 90.604 1.00 0.00

ATOM 539 H GLN A 33 20.925 14.189 92.700 1.00 0.00

ATOM 540 H GLN A 33 22.405 14.533 93.637 1.00 0.00

ATOM 541 H GLN A 33 21.590 10.748 92.849 1.00 0.00

ATOM 542 H GLN A 33 20.419 12.088 92.982 1.00 0.00

ATOM 543 N CYS A 34 21.944 17.355 89.434 1.00 73.23

ATOM 544 CA CYS A 34 22.389 18.016 88.211 1.00 75.37

ATOM 545 C CYS A 34 23.094 19.389 88.349 1.00 76.11

ATOM 546 O CYS A 34 22.693 20.223 89.158 1.00 74.54

ATOM 547 CB CYS A 34 21.250 18.048 87.204 1.00 73.47

ATOM 548 SG CYS A 34 21.354 16.605 86.161 1.00 78.47

ATOM 549 H CYS A 34 20.942 17.166 89.577 1.00 0.00

ATOM 550 H CYS A 34 23.208 17.395 87.849 1.00 0.00

ATOM 551 H CYS A 34 21.461 18.934 86.604 1.00 0.00

ATOM 552 H CYS A 34 20.311 18.194 87.737 1.00 0.00

ATOM 553 H CYS A 34 20.594 16.657 85.513 1.00 0.00

ATOM 554 N PRO A 35 24.173 19.616 87.575 1.00 78.94

ATOM 555 CA PRO A 35 24.717 20.965 87.617 1.00 80.04

ATOM 556 C PRO A 35 23.703 22.068 87.212 1.00 77.81

ATOM 557 O PRO A 35 22.756 21.827 86.457 1.00 75.54

ATOM 558 CB PRO A 35 25.871 20.904 86.627 1.00 83.42

ATOM 559 CG PRO A 35 26.288 19.467 86.641 1.00 84.60

ATOM 560 CD PRO A 35 25.079 18.661 86.916 1.00 81.68

ATOM 561 H PRO A 35 25.007 21.241 88.631 1.00 0.00

ATOM 562 H PRO A 35 26.690 21.573 86.890 1.00 0.00

ATOM 563 H PRO A 35 25.575 21.226 85.629 1.00 0.00

ATOM 564 H PRO A 35 27.049 19.288 87.401 1.00 0.00

ATOM 565 H PRO A 35 26.717 19.183 85.680 1.00 0.00

ATOM 566 H PRO A 35 24.646 18.235 86.011 1.00 0.00

ATOM 567 H PRO A 35 25.287 17.787 87.534 1.00 0.00

ATOM 568 N PHE A 36 23.913 23.259 87.756 1.00 77.92

ATOM 569 CA PHE A 36 23.178 24.466 87.401 1.00 76.38

ATOM 570 C PHE A 36 23.089 24.755 85.883 1.00 77.98

ATOM 571 O PHE A 36 21.981 24.944 85.357 1.00 76.14

ATOM 572 CB PHE A 36 23.800 25.664 88.154 1.00 77.20

ATOM 573 CG PHE A 36 23.131 26.963 87.860 1.00 75.44

ATOM 574 CD1 PHE A 36 21.770 27.131 88.117 1.00 70.22

ATOM 575 CD2 PHE A 36 23.858 28.025 87.306 1.00 75.57

ATOM 576 CE1 PHE A 36 21.133 28.343 87.829 1.00 71.09

ATOM 577 CE2 PHE A 36 23.241 29.231 87.022 1.00 73.15

ATOM 578 CZ PHE A 36 21.873 29.401 87.272 1.00 72.11

ATOM 579 H PHE A 36 24.644 23.336 88.477 1.00 0.00

ATOM 580 H PHE A 36 22.144 24.304 87.705 1.00 0.00

ATOM 581 H PHE A 36 24.846 25.762 87.864 1.00 0.00

ATOM 582 H PHE A 36 23.659 25.488 89.221 1.00 0.00

ATOM 583 H PHE A 36 21.197 26.309 88.547 1.00 0.00

ATOM 584 H PHE A 36 24.920 27.900 87.097 1.00 0.00

ATOM 585 H PHE A 36 20.070 28.466 88.035 1.00 0.00

ATOM 586 H PHE A 36 23.822 30.052 86.602 1.00 0.00

ATOM 587 H PHE A 36 21.386 30.347 87.036 1.00 0.00

ATOM 588 N ASP A 37 24.244 24.773 85.198 1.00 81.37

ATOM 589 CA ASP A 37 24.351 25.071 83.756 1.00 83.05

ATOM 590 C ASP A 37 23.416 24.215 82.892 1.00 81.17

ATOM 591 O ASP A 37 22.822 24.697 81.922 1.00 80.77

ATOM 592 CB ASP A 37 25.816 24.902 83.274 1.00 87.48

ATOM 593 CG ASP A 37 26.707 26.142 83.570 1.00 93.26

ATOM 594 OD1 ASP A 37 27.937 26.122 83.252 1.00100.86

ATOM 595 OD2 ASP A 37 26.195 27.161 84.104 1.00 94.77

ATOM 596 H ASP A 37 25.112 24.565 85.712 1.00 0.00

ATOM 597 H ASP A 37 24.038 26.108 83.633 1.00 0.00

ATOM 598 H ASP A 37 25.792 24.764 82.193 1.00 0.00

ATOM 599 H ASP A 37 26.243 24.052 83.807 1.00 0.00

ATOM 600 N GLU A 38 23.280 22.942 83.238 1.00 80.05

ATOM 601 CA GLU A 38 22.416 22.054 82.469 1.00 79.06

ATOM 602 C GLU A 38 20.961 22.429 82.639 1.00 75.53

ATOM 603 O GLU A 38 20.181 22.297 81.718 1.00 74.81

ATOM 604 CB GLU A 38 22.605 20.604 82.876 1.00 79.01

ATOM 605 CG GLU A 38 24.064 20.223 83.229 1.00 85.38

ATOM 606 CD GLU A 38 25.130 20.791 82.285 1.00 89.70

ATOM 607 OE1 GLU A 38 25.015 20.582 81.041 1.00 92.57

ATOM 608 OE2 GLU A 38 26.099 21.422 82.799 1.00 91.08

ATOM 609 H GLU A 38 23.789 22.578 84.056 1.00 0.00

ATOM 610 H GLU A 38 22.699 22.167 81.422 1.00 0.00

ATOM 611 H GLU A 38 22.319 20.005 82.011 1.00 0.00

ATOM 612 H GLU A 38 21.992 20.438 83.762 1.00 0.00

ATOM 613 H GLU A 38 24.137 19.137 83.168 1.00 0.00

ATOM 614 H GLU A 38 24.270 20.645 84.213 1.00 0.00

ATOM 615 N HIS A 39 20.596 22.882 83.833 1.00 73.89

ATOM 616 CA HIS A 39 19.223 23.278 84.081 1.00 71.49

ATOM 617 C HIS A 39 18.880 24.587 83.324 1.00 70.75

ATOM 618 O HIS A 39 17.751 24.829 82.894 1.00 67.87

ATOM 619 CB HIS A 39 18.957 23.370 85.601 1.00 69.28

ATOM 620 CG HIS A 39 18.642 22.052 86.239 1.00 71.83

ATOM 621 ND1 HIS A 39 19.395 21.519 87.267 1.00 72.68

ATOM 622 CD2 HIS A 39 17.667 21.141 85.976 1.00 72.73

ATOM 623 CE1 HIS A 39 18.897 20.340 87.606 1.00 74.09

ATOM 624 NE2 HIS A 39 17.859 20.078 86.831 1.00 71.79

ATOM 625 H HIS A 39 21.293 22.953 84.588 1.00 0.00

ATOM 626 H HIS A 39 18.552 22.514 83.688 1.00 0.00

ATOM 627 H HIS A 39 18.101 24.024 85.768 1.00 0.00

ATOM 628 H HIS A 39 19.876 23.721 86.071 1.00 0.00

ATOM 629 H HIS A 39 20.214 21.968 87.701 1.00 0.00

ATOM 630 H HIS A 39 16.880 21.235 85.228 1.00 0.00

ATOM 631 H HIS A 39 19.280 19.692 88.394 1.00 0.00

ATOM 632 N VAL A 40 19.872 25.437 83.177 1.00 73.18

ATOM 633 CA VAL A 40 19.615 26.697 82.551 1.00 74.51

ATOM 634 C VAL A 40 19.316 26.451 81.088 1.00 76.11

ATOM 635 O VAL A 40 18.427 27.116 80.523 1.00 75.88

ATOM 636 CB VAL A 40 20.780 27.630 82.724 1.00 77.49

ATOM 637 CG1 VAL A 40 20.494 28.995 82.050 1.00 77.82

ATOM 638 CG2 VAL A 40 21.110 27.752 84.239 1.00 75.43

ATOM 639 H VAL A 40 20.818 25.200 83.507 1.00 0.00

ATOM 640 H VAL A 40 18.756 27.176 83.020 1.00 0.00

ATOM 641 H VAL A 40 21.662 27.230 82.224 1.00 0.00

ATOM 642 H VAL A 40 21.372 29.620 82.211 1.00 0.00

ATOM 643 H VAL A 40 20.325 28.799 80.991 1.00 0.00

ATOM 644 H VAL A 40 19.608 29.409 82.532 1.00 0.00

ATOM 645 H VAL A 40 21.956 28.433 84.327 1.00 0.00

ATOM 646 H VAL A 40 20.220 28.147 84.728 1.00 0.00

ATOM 647 H VAL A 40 21.357 26.751 84.593 1.00 0.00

ATOM 648 N LYS A 41 20.020 25.486 80.483 1.00 77.73

ATOM 649 CA LYS A 41 19.664 25.056 79.135 1.00 78.30

ATOM 650 C LYS A 41 18.236 24.475 79.084 1.00 75.28

ATOM 651 O LYS A 41 17.428 24.822 78.213 1.00 74.88

ATOM 652 CB LYS A 41 20.673 24.056 78.573 1.00 80.79

ATOM 653 CG LYS A 41 22.020 24.641 78.286 1.00 83.94

ATOM 654 CD LYS A 41 23.066 23.547 78.230 1.00 87.54

ATOM 655 CE LYS A 41 24.460 24.104 78.486 1.00 92.45

ATOM 656 NZ LYS A 41 24.663 24.489 79.925 1.00 92.04

ATOM 657 H LYS A 41 20.814 25.047 80.970 1.00 0.00

ATOM 658 H LYS A 41 19.690 25.944 78.504 1.00 0.00

ATOM 659 H LYS A 41 20.281 23.674 77.631 1.00 0.00

ATOM 660 H LYS A 41 20.827 23.292 79.335 1.00 0.00

ATOM 661 H LYS A 41 22.274 25.336 79.086 1.00 0.00

ATOM 662 H LYS A 41 21.985 25.162 77.329 1.00 0.00

ATOM 663 H LYS A 41 23.046 23.103 77.235 1.00 0.00

ATOM 664 H LYS A 41 22.837 22.807 78.997 1.00 0.00

ATOM 665 H LYS A 41 24.597 24.996 77.874 1.00 0.00

ATOM 666 H LYS A 41 25.185 23.326 78.248 1.00 0.00

ATOM 667 H LYS A 41 25.623 24.852 80.007 1.00 0.00

ATOM 668 H LYS A 41 23.961 25.210 80.146 1.00 0.00

ATOM 669 H LYS A 41 24.527 23.637 80.487 1.00 0.00

ATOM 670 N LEU A 42 17.896 23.602 80.004 1.00 72.99

ATOM 671 CA LEU A 42 16.574 23.032 79.854 1.00 71.82

ATOM 672 C LEU A 42 15.510 24.136 79.960 1.00 70.72

ATOM 673 O LEU A 42 14.579 24.158 79.148 1.00 70.66

ATOM 674 CB LEU A 42 16.315 21.812 80.774 1.00 69.70

ATOM 675 CG LEU A 42 17.397 20.721 80.895 1.00 72.26

ATOM 676 CD1 LEU A 42 16.783 19.495 81.486 1.00 69.38

ATOM 677 CD2 LEU A 42 18.195 20.324 79.598 1.00 73.23

ATOM 678 H LEU A 42 18.525 23.341 80.777 1.00 0.00

ATOM 679 H LEU A 42 16.504 22.607 78.853 1.00 0.00

ATOM 680 H LEU A 42 15.454 21.305 80.338 1.00 0.00

ATOM 681 H LEU A 42 16.232 22.230 81.777 1.00 0.00

ATOM 682 H LEU A 42 18.156 21.179 81.530 1.00 0.00

ATOM 683 H LEU A 42 17.572 18.746 81.558 1.00 0.00

ATOM 684 H LEU A 42 16.398 19.773 82.467 1.00 0.00

ATOM 685 H LEU A 42 15.986 19.181 80.812 1.00 0.00

ATOM 686 H LEU A 42 18.904 19.548 79.885 1.00 0.00

ATOM 687 H LEU A 42 17.466 19.959 78.874 1.00 0.00

ATOM 688 H LEU A 42 18.700 21.224 79.246 1.00 0.00

ATOM 689 N VAL A 43 15.639 25.033 80.952 1.00 70.36

ATOM 690 CA VAL A 43 14.733 26.199 81.083 1.00 69.49

ATOM 691 C VAL A 43 14.643 27.076 79.792 1.00 71.51

ATOM 692 O VAL A 43 13.534 27.313 79.308 1.00 70.88

ATOM 693 CB VAL A 43 15.046 27.124 82.342 1.00 69.74

ATOM 694 CG1 VAL A 43 14.213 28.447 82.312 1.00 67.13

ATOM 695 CG2 VAL A 43 14.843 26.387 83.678 1.00 65.86

ATOM 696 H VAL A 43 16.392 24.906 81.644 1.00 0.00

ATOM 697 H VAL A 43 13.759 25.738 81.244 1.00 0.00

ATOM 698 H VAL A 43 16.102 27.386 82.270 1.00 0.00

ATOM 699 H VAL A 43 14.486 29.016 83.201 1.00 0.00

ATOM 700 H VAL A 43 14.483 28.973 81.396 1.00 0.00

ATOM 701 H VAL A 43 13.161 28.162 82.321 1.00 0.00

ATOM 702 H VAL A 43 15.079 27.095 84.472 1.00 0.00

ATOM 703 H VAL A 43 13.800 26.072 83.713 1.00 0.00

ATOM 704 H VAL A 43 15.524 25.536 83.679 1.00 0.00

ATOM 705 N ASN A 44 15.774 27.554 79.255 1.00 73.80

ATOM 706 CA ASN A 44 15.761 28.398 78.049 1.00 76.33

ATOM 707 C ASN A 44 15.022 27.722 76.894 1.00 77.10

ATOM 708 O ASN A 44 14.078 28.308 76.347 1.00 77.87

ATOM 709 CB ASN A 44 17.181 28.797 77.600 1.00 79.05

ATOM 710 CG ASN A 44 17.811 29.873 78.492 1.00 80.96

ATOM 711 OD1 ASN A 44 17.120 30.626 79.176 1.00 79.51

ATOM 712 ND2 ASN A 44 19.143 29.951 78.468 1.00 84.89

ATOM 713 H ASN A 44 16.675 27.325 79.697 1.00 0.00

ATOM 714 H ASN A 44 15.225 29.307 78.322 1.00 0.00

ATOM 715 H ASN A 44 17.113 29.201 76.590 1.00 0.00

ATOM 716 H ASN A 44 17.811 27.909 77.662 1.00 0.00

ATOM 717 H ASN A 44 19.628 30.653 79.045 1.00 0.00

ATOM 718 H ASN A 44 19.686 29.309 77.873 1.00 0.00

ATOM 719 N GLU A 45 15.420 26.495 76.543 1.00 77.21

ATOM 720 CA GLU A 45 14.709 25.706 75.515 1.00 77.66

ATOM 721 C GLU A 45 13.182 25.698 75.658 1.00 74.76

ATOM 722 O GLU A 45 12.451 26.026 74.716 1.00 75.79

ATOM 723 CB GLU A 45 15.240 24.260 75.424 1.00 78.16

ATOM 724 CG GLU A 45 14.184 23.236 74.993 1.00 79.58

ATOM 725 CD GLU A 45 14.774 21.922 74.487 1.00 85.28

ATOM 726 OE1 GLU A 45 14.696 21.676 73.247 1.00 87.74

ATOM 727 OE2 GLU A 45 15.310 21.139 75.323 1.00 84.26

ATOM 728 H GLU A 45 16.247 26.087 77.003 1.00 0.00

ATOM 729 H GLU A 45 14.927 26.228 74.583 1.00 0.00

ATOM 730 H GLU A 45 15.584 23.972 76.418 1.00 0.00

ATOM 731 H GLU A 45 16.023 24.249 74.665 1.00 0.00

ATOM 732 H GLU A 45 13.615 23.675 74.173 1.00 0.00

ATOM 733 H GLU A 45 13.580 23.002 75.870 1.00 0.00

ATOM 734 N LEU A 46 12.717 25.299 76.828 1.00 72.28

ATOM 735 CA LEU A 46 11.283 25.131 77.101 1.00 70.27

ATOM 736 C LEU A 46 10.625 26.488 77.000 1.00 70.03

ATOM 737 O LEU A 46 9.471 26.615 76.544 1.00 69.73

ATOM 738 CB LEU A 46 11.074 24.550 78.492 1.00 67.86

ATOM 739 CG LEU A 46 9.765 23.783 78.567 1.00 69.40

ATOM 740 CD1 LEU A 46 9.811 22.579 77.586 1.00 71.32

ATOM 741 CD2 LEU A 46 9.429 23.325 80.009 1.00 64.87

ATOM 742 H LEU A 46 13.389 25.097 77.582 1.00 0.00

ATOM 743 H LEU A 46 10.843 24.443 76.379 1.00 0.00

ATOM 744 H LEU A 46 11.044 25.366 79.214 1.00 0.00

ATOM 745 H LEU A 46 11.893 23.866 78.713 1.00 0.00

ATOM 746 H LEU A 46 8.961 24.457 78.270 1.00 0.00

ATOM 747 H LEU A 46 8.856 22.060 77.673 1.00 0.00

ATOM 748 H LEU A 46 9.960 22.988 76.587 1.00 0.00

ATOM 749 H LEU A 46 10.645 21.948 77.895 1.00 0.00

ATOM 750 H LEU A 46 8.481 22.789 79.960 1.00 0.00

ATOM 751 H LEU A 46 10.244 22.679 80.336 1.00 0.00

ATOM 752 H LEU A 46 9.355 24.225 80.620 1.00 0.00

ATOM 753 N THR A 47 11.402 27.500 77.392 1.00 69.70

ATOM 754 CA THR A 47 10.989 28.862 77.332 1.00 69.89

ATOM 755 C THR A 47 10.877 29.315 75.878 1.00 72.17

ATOM 756 O THR A 47 9.837 29.855 75.521 1.00 72.47

ATOM 757 CB THR A 47 11.909 29.750 78.178 1.00 71.08

ATOM 758 OG1 THR A 47 11.623 29.520 79.570 1.00 69.52

ATOM 759 CG2 THR A 47 11.712 31.238 77.861 1.00 70.60

ATOM 760 H THR A 47 12.342 27.287 77.754 1.00 0.00

ATOM 761 H THR A 47 9.995 28.960 77.768 1.00 0.00

ATOM 762 H THR A 47 12.942 29.493 77.946 1.00 0.00

ATOM 763 H THR A 47 11.765 28.515 79.745 1.00 0.00

ATOM 764 H THR A 47 12.398 31.794 78.500 1.00 0.00

ATOM 765 H THR A 47 11.944 31.372 76.805 1.00 0.00

ATOM 766 H THR A 47 10.671 31.476 78.081 1.00 0.00

ATOM 767 N GLU A 48 11.871 29.087 75.018 1.00 73.61

ATOM 768 CA GLU A 48 11.620 29.358 73.574 1.00 77.09

ATOM 769 C GLU A 48 10.273 28.777 73.118 1.00 75.75

ATOM 770 O GLU A 48 9.410 29.495 72.589 1.00 76.53

ATOM 771 CB GLU A 48 12.734 28.858 72.651 1.00 78.98

ATOM 772 CG GLU A 48 13.923 29.866 72.540 1.00 85.83

ATOM 773 CD GLU A 48 15.228 29.382 73.216 1.00 89.53

ATOM 774 OE1 GLU A 48 16.130 30.207 73.497 1.00 92.02

ATOM 775 OE2 GLU A 48 15.364 28.165 73.462 1.00 92.22

ATOM 776 H GLU A 48 12.783 28.735 75.342 1.00 0.00

ATOM 777 H GLU A 48 11.596 30.444 73.489 1.00 0.00

ATOM 778 H GLU A 48 12.299 28.744 71.658 1.00 0.00

ATOM 779 H GLU A 48 13.108 27.918 73.057 1.00 0.00

ATOM 780 H GLU A 48 13.623 30.786 73.041 1.00 0.00

ATOM 781 H GLU A 48 14.144 29.987 71.480 1.00 0.00

ATOM 782 N PHE A 49 10.083 27.492 73.410 1.00 73.56

ATOM 783 CA PHE A 49 8.934 26.762 72.942 1.00 72.75

ATOM 784 C PHE A 49 7.652 27.350 73.496 1.00 71.49

ATOM 785 O PHE A 49 6.618 27.351 72.843 1.00 72.20

ATOM 786 CB PHE A 49 9.057 25.282 73.323 1.00 71.54

ATOM 787 CG PHE A 49 7.915 24.449 72.843 1.00 69.74

ATOM 788 CD1 PHE A 49 7.815 24.081 71.508 1.00 70.08

ATOM 789 CD2 PHE A 49 6.927 24.040 73.727 1.00 68.93

ATOM 790 CE1 PHE A 49 6.761 23.342 71.050 1.00 68.35

ATOM 791 CE2 PHE A 49 5.863 23.265 73.274 1.00 66.77

ATOM 792 CZ PHE A 49 5.791 22.924 71.929 1.00 70.25

ATOM 793 H PHE A 49 10.781 27.004 73.990 1.00 0.00

ATOM 794 H PHE A 49 8.897 26.843 71.856 1.00 0.00

ATOM 795 H PHE A 49 9.087 25.203 74.410 1.00 0.00

ATOM 796 H PHE A 49 9.950 24.891 72.836 1.00 0.00

ATOM 797 H PHE A 49 8.594 24.388 70.810 1.00 0.00

ATOM 798 H PHE A 49 6.984 24.326 74.777 1.00 0.00

ATOM 799 H PHE A 49 6.690 23.085 69.993 1.00 0.00

ATOM 800 H PHE A 49 5.093 22.929 73.968 1.00 0.00

ATOM 801 H PHE A 49 4.958 22.320 71.568 1.00 0.00

ATOM 802 N ALA A 50 7.713 27.809 74.729 1.00 70.40

ATOM 803 CA ALA A 50 6.622 28.592 75.253 1.00 70.27

ATOM 804 C ALA A 50 6.290 29.823 74.354 1.00 72.94

ATOM 805 O ALA A 50 5.126 30.025 74.052 1.00 73.52

ATOM 806 CB ALA A 50 6.927 29.006 76.644 1.00 68.53

ATOM 807 H ALA A 50 8.536 27.610 75.316 1.00 0.00

ATOM 808 H ALA A 50 5.729 27.967 75.255 1.00 0.00

ATOM 809 H ALA A 50 6.078 29.592 76.995 1.00 0.00

ATOM 810 H ALA A 50 7.059 28.094 77.226 1.00 0.00

ATOM 811 H ALA A 50 7.841 29.599 76.607 1.00 0.00

ATOM 812 N LYS A 51 7.283 30.619 73.916 1.00 74.61

ATOM 813 CA LYS A 51 6.989 31.844 73.166 1.00 77.49

ATOM 814 C LYS A 51 6.336 31.449 71.846 1.00 79.80

ATOM 815 O LYS A 51 5.379 32.124 71.375 1.00 81.98

ATOM 816 CB LYS A 51 8.259 32.682 72.868 1.00 79.95

ATOM 817 CG LYS A 51 8.945 33.420 74.082 1.00 78.93

ATOM 818 CD LYS A 51 10.474 33.585 73.790 1.00 80.10

ATOM 819 CE LYS A 51 11.352 33.913 75.045 1.00 78.42

ATOM 820 NZ LYS A 51 11.791 35.364 75.084 1.00 79.30

ATOM 821 H LYS A 51 8.262 30.364 74.110 1.00 0.00

ATOM 822 H LYS A 51 6.329 32.462 73.775 1.00 0.00

ATOM 823 H LYS A 51 7.922 33.472 72.197 1.00 0.00

ATOM 824 H LYS A 51 8.994 31.975 72.483 1.00 0.00

ATOM 825 H LYS A 51 8.808 32.839 74.994 1.00 0.00

ATOM 826 H LYS A 51 8.496 34.405 74.211 1.00 0.00

ATOM 827 H LYS A 51 10.575 34.427 73.105 1.00 0.00

ATOM 828 H LYS A 51 10.826 32.631 73.398 1.00 0.00

ATOM 829 H LYS A 51 12.248 33.292 75.031 1.00 0.00

ATOM 830 H LYS A 51 10.743 33.747 75.933 1.00 0.00

ATOM 831 H LYS A 51 12.358 35.484 75.936 1.00 0.00

ATOM 832 H LYS A 51 12.341 35.535 74.230 1.00 0.00

ATOM 833 H LYS A 51 10.935 35.937 75.109 1.00 0.00

ATOM 834 N THR A 52 6.844 30.356 71.246 1.00 78.40

ATOM 835 CA THR A 52 6.375 29.932 69.941 1.00 78.31

ATOM 836 C THR A 52 4.898 29.509 70.037 1.00 77.32

ATOM 837 O THR A 52 4.170 29.587 69.042 1.00 79.24

ATOM 838 CB THR A 52 7.247 28.776 69.383 1.00 78.39

ATOM 839 OG1 THR A 52 8.479 29.291 68.875 1.00 77.97

ATOM 840 CG2 THR A 52 6.563 28.080 68.275 1.00 78.16

ATOM 841 H THR A 52 7.578 29.812 71.722 1.00 0.00

ATOM 842 H THR A 52 6.460 30.769 69.248 1.00 0.00

ATOM 843 H THR A 52 7.426 28.080 70.203 1.00 0.00

ATOM 844 H THR A 52 8.948 29.774 69.655 1.00 0.00

ATOM 845 H THR A 52 7.230 27.287 67.936 1.00 0.00

ATOM 846 H THR A 52 5.631 27.682 68.676 1.00 0.00

ATOM 847 H THR A 52 6.385 28.820 67.495 1.00 0.00

ATOM 848 N CYS A 53 4.454 29.078 71.217 1.00 73.32

ATOM 849 CA CYS A 53 3.082 28.590 71.358 1.00 72.68

ATOM 850 C CYS A 53 2.129 29.719 71.649 1.00 72.51

ATOM 851 O CYS A 53 0.927 29.628 71.343 1.00 72.00

ATOM 852 CB CYS A 53 2.927 27.541 72.460 1.00 71.04

ATOM 853 SG CYS A 53 3.687 25.966 72.203 1.00 71.87

ATOM 854 H CYS A 53 5.080 29.088 72.035 1.00 0.00

ATOM 855 H CYS A 53 2.844 28.125 70.401 1.00 0.00

ATOM 856 H CYS A 53 1.857 27.348 72.384 1.00 0.00

ATOM 857 H CYS A 53 3.289 28.032 73.363 1.00 0.00

ATOM 858 N VAL A 54 2.682 30.763 72.262 1.00 72.06

ATOM 859 CA VAL A 54 2.036 32.077 72.372 1.00 74.04

ATOM 860 C VAL A 54 1.906 32.681 70.963 1.00 76.75

ATOM 861 O VAL A 54 0.805 33.047 70.545 1.00 78.35

ATOM 862 CB VAL A 54 2.790 33.030 73.372 1.00 74.28

ATOM 863 CG1 VAL A 54 2.629 34.447 72.978 1.00 78.29

ATOM 864 CG2 VAL A 54 2.304 32.868 74.823 1.00 72.55

ATOM 865 H VAL A 54 3.615 30.643 72.682 1.00 0.00

ATOM 866 H VAL A 54 1.040 31.954 72.796 1.00 0.00

ATOM 867 H VAL A 54 3.840 32.743 73.324 1.00 0.00

ATOM 868 H VAL A 54 3.173 35.045 73.709 1.00 0.00

ATOM 869 H VAL A 54 3.049 34.549 71.978 1.00 0.00

ATOM 870 H VAL A 54 1.561 34.662 72.993 1.00 0.00

ATOM 871 H VAL A 54 2.881 33.564 75.432 1.00 0.00

ATOM 872 H VAL A 54 1.241 33.108 74.832 1.00 0.00

ATOM 873 H VAL A 54 2.488 31.832 75.107 1.00 0.00

ATOM 874 N ALA A 55 2.985 32.718 70.193 1.00 77.68

ATOM 875 CA ALA A 55 2.854 33.222 68.821 1.00 81.93

ATOM 876 C ALA A 55 1.891 32.399 67.949 1.00 84.16

ATOM 877 O ALA A 55 0.989 32.971 67.318 1.00 87.15

ATOM 878 CB ALA A 55 4.211 33.409 68.142 1.00 82.18

ATOM 879 H ALA A 55 3.897 32.399 70.551 1.00 0.00

ATOM 880 H ALA A 55 2.397 34.207 68.920 1.00 0.00

ATOM 881 H ALA A 55 4.016 33.783 67.137 1.00 0.00

ATOM 882 H ALA A 55 4.770 34.128 68.740 1.00 0.00

ATOM 883 H ALA A 55 4.696 32.433 68.124 1.00 0.00

ATOM 884 N ASP A 56 2.056 31.073 67.932 1.00 82.66

ATOM 885 CA ASP A 56 1.211 30.198 67.110 1.00 84.29

ATOM 886 C ASP A 56 0.657 29.025 67.918 1.00 82.04

ATOM 887 O ASP A 56 1.417 28.132 68.268 1.00 78.95

ATOM 888 CB ASP A 56 2.014 29.615 65.928 1.00 85.56

ATOM 889 CG ASP A 56 1.196 28.625 65.105 1.00 89.57

ATOM 890 OD1 ASP A 56 -0.054 28.801 65.079 1.00 91.31

ATOM 891 OD2 ASP A 56 1.786 27.678 64.489 1.00 93.59

ATOM 892 H ASP A 56 2.797 30.653 68.512 1.00 0.00

ATOM 893 H ASP A 56 0.388 30.814 66.748 1.00 0.00

ATOM 894 H ASP A 56 2.882 29.089 66.324 1.00 0.00

ATOM 895 H ASP A 56 2.294 30.438 65.270 1.00 0.00

ATOM 896 N GLU A 57 -0.651 28.996 68.172 1.00 82.51

ATOM 897 CA GLU A 57 -1.186 28.011 69.133 1.00 81.02

ATOM 898 C GLU A 57 -1.382 26.572 68.608 1.00 79.47

ATOM 899 O GLU A 57 -1.203 25.573 69.341 1.00 78.69

ATOM 900 CB GLU A 57 -2.424 28.554 69.841 1.00 81.98

ATOM 901 CG GLU A 57 -2.030 29.136 71.193 1.00 83.11

ATOM 902 CD GLU A 57 -3.137 29.962 71.879 1.00 86.81

ATOM 903 OE1 GLU A 57 -3.954 30.619 71.179 1.00 92.13

ATOM 904 OE2 GLU A 57 -3.181 29.944 73.120 1.00 82.68

ATOM 905 H GLU A 57 -1.285 29.657 67.701 1.00 0.00

ATOM 906 H GLU A 57 -0.387 27.885 69.864 1.00 0.00

ATOM 907 H GLU A 57 -3.139 27.745 69.991 1.00 0.00

ATOM 908 H GLU A 57 -2.875 29.336 69.230 1.00 0.00

ATOM 909 H GLU A 57 -1.195 29.812 71.008 1.00 0.00

ATOM 910 H GLU A 57 -1.794 28.296 71.846 1.00 0.00

ATOM 911 N SER A 58 -1.737 26.512 67.343 1.00 80.21

ATOM 912 CA SER A 58 -1.806 25.335 66.499 1.00 79.44

ATOM 913 C SER A 58 -0.505 24.640 66.247 1.00 77.69

ATOM 914 O SER A 58 -0.521 23.621 65.653 1.00 78.50

ATOM 915 CB SER A 58 -2.226 25.799 65.101 1.00 81.73

ATOM 916 OG SER A 58 -3.570 26.161 65.062 1.00 82.89

ATOM 917 H SER A 58 -1.996 27.405 66.900 1.00 0.00

ATOM 918 H SER A 58 -2.479 24.655 67.021 1.00 0.00

ATOM 919 H SER A 58 -2.059 25.036 64.341 1.00 0.00

ATOM 920 H SER A 58 -1.687 26.718 64.873 1.00 0.00

ATOM 921 H SER A 58 -3.774 26.459 64.097 1.00 0.00

ATOM 922 N HIS A 59 0.625 25.226 66.568 1.00 77.34

ATOM 923 CA HIS A 59 1.884 24.615 66.226 1.00 78.14

ATOM 924 C HIS A 59 2.051 23.323 67.051 1.00 76.59

ATOM 925 O HIS A 59 1.573 23.230 68.183 1.00 76.01

ATOM 926 CB HIS A 59 3.005 25.643 66.411 1.00 79.24

ATOM 927 CG HIS A 59 4.374 25.115 66.129 1.00 83.78

ATOM 928 ND1 HIS A 59 5.150 24.498 67.094 1.00 85.11

ATOM 929 CD2 HIS A 59 5.108 25.099 64.990 1.00 88.85

ATOM 930 CE1 HIS A 59 6.291 24.102 66.555 1.00 87.14

ATOM 931 NE2 HIS A 59 6.294 24.456 65.278 1.00 90.04

ATOM 932 H HIS A 59 0.611 26.127 67.067 1.00 0.00

ATOM 933 H HIS A 59 1.924 24.314 65.179 1.00 0.00

ATOM 934 H HIS A 59 2.996 25.974 67.450 1.00 0.00

ATOM 935 H HIS A 59 2.833 26.439 65.687 1.00 0.00

ATOM 936 H HIS A 59 4.881 24.367 68.080 1.00 0.00

ATOM 937 H HIS A 59 4.816 25.516 64.026 1.00 0.00

ATOM 938 H HIS A 59 7.092 23.575 67.073 1.00 0.00

ATOM 939 N ALA A 60 2.669 22.306 66.460 1.00 76.77

ATOM 940 CA ALA A 60 2.778 20.962 67.031 1.00 75.50

ATOM 941 C ALA A 60 3.228 20.940 68.501 1.00 73.14

ATOM 942 O ALA A 60 4.116 21.680 68.890 1.00 74.03

ATOM 943 CB ALA A 60 3.722 20.105 66.158 1.00 76.15

ATOM 944 H ALA A 60 3.100 22.474 65.540 1.00 0.00

ATOM 945 H ALA A 60 1.773 20.539 67.030 1.00 0.00

ATOM 946 H ALA A 60 3.773 19.118 66.618 1.00 0.00

ATOM 947 H ALA A 60 3.285 20.065 65.160 1.00 0.00

ATOM 948 H ALA A 60 4.692 20.602 66.155 1.00 0.00

ATOM 949 N GLY A 61 2.604 20.126 69.328 1.00 70.92

ATOM 950 CA GLY A 61 3.049 20.015 70.725 1.00 68.78

ATOM 951 C GLY A 61 2.535 21.048 71.738 1.00 67.99

ATOM 952 O GLY A 61 2.563 20.824 72.951 1.00 66.24

ATOM 953 H GLY A 61 1.805 19.568 68.996 1.00 0.00

ATOM 954 H GLY A 61 4.123 20.188 70.662 1.00 0.00

ATOM 955 H GLY A 61 2.701 19.037 71.058 1.00 0.00

ATOM 956 N CYS A 62 2.068 22.185 71.237 1.00 68.47

ATOM 957 CA CYS A 62 1.525 23.230 72.045 1.00 67.31

ATOM 958 C CYS A 62 0.283 22.846 72.793 1.00 66.38

ATOM 959 O CYS A 62 -0.057 23.532 73.753 1.00 65.16

ATOM 960 CB CYS A 62 1.233 24.439 71.167 1.00 70.63

ATOM 961 SG CYS A 62 2.737 25.186 70.541 1.00 75.61

ATOM 962 H CYS A 62 2.099 22.320 70.216 1.00 0.00

ATOM 963 H CYS A 62 2.276 23.457 72.801 1.00 0.00

ATOM 964 H CYS A 62 0.793 25.137 71.879 1.00 0.00

ATOM 965 H CYS A 62 0.515 24.127 70.409 1.00 0.00

ATOM 966 N GLU A 63 -0.384 21.748 72.403 1.00 67.23

ATOM 967 CA GLU A 63 -1.500 21.181 73.230 1.00 68.42

ATOM 968 C GLU A 63 -1.140 20.076 74.235 1.00 67.32

ATOM 969 O GLU A 63 -2.016 19.652 74.992 1.00 69.17

ATOM 970 CB GLU A 63 -2.643 20.652 72.386 1.00 70.40

ATOM 971 CG GLU A 63 -2.425 19.195 71.907 1.00 73.06

ATOM 972 CD GLU A 63 -1.191 18.996 71.000 1.00 73.76

ATOM 973 OE1 GLU A 63 -1.144 17.945 70.361 1.00 78.63

ATOM 974 OE2 GLU A 63 -0.266 19.842 70.924 1.00 72.27

ATOM 975 H GLU A 63 -0.125 21.286 71.520 1.00 0.00

ATOM 976 H GLU A 63 -1.786 22.060 73.808 1.00 0.00

ATOM 977 H GLU A 63 -2.699 21.285 71.501 1.00 0.00

ATOM 978 H GLU A 63 -3.546 20.685 72.996 1.00 0.00

ATOM 979 H GLU A 63 -3.300 18.916 71.320 1.00 0.00

ATOM 980 H GLU A 63 -2.264 18.587 72.798 1.00 0.00

ATOM 981 N LYS A 64 0.100 19.576 74.238 1.00 66.17

ATOM 982 CA LYS A 64 0.498 18.486 75.185 1.00 64.27

ATOM 983 C LYS A 64 0.487 19.072 76.555 1.00 62.78

ATOM 984 O LYS A 64 0.748 20.273 76.653 1.00 63.22

ATOM 985 CB LYS A 64 1.897 18.006 74.887 1.00 63.08

ATOM 986 CG LYS A 64 1.919 17.034 73.686 1.00 65.93

ATOM 987 CD LYS A 64 3.330 16.776 73.158 1.00 67.10

ATOM 988 CE LYS A 64 3.316 15.729 72.032 1.00 65.20

ATOM 989 NZ LYS A 64 4.137 16.298 70.865 1.00 70.23

ATOM 990 H LYS A 64 0.796 19.948 73.576 1.00 0.00

ATOM 991 H LYS A 64 -0.188 17.644 75.091 1.00 0.00

ATOM 992 H LYS A 64 2.255 17.473 75.768 1.00 0.00

ATOM 993 H LYS A 64 2.519 18.873 74.664 1.00 0.00

ATOM 994 H LYS A 64 1.336 17.476 72.878 1.00 0.00

ATOM 995 H LYS A 64 1.518 16.078 74.023 1.00 0.00

ATOM 996 H LYS A 64 3.941 16.399 73.978 1.00 0.00

ATOM 997 H LYS A 64 3.733 17.710 72.767 1.00 0.00

ATOM 998 H LYS A 64 2.294 15.522 71.715 1.00 0.00

ATOM 999 H LYS A 64 3.747 14.790 72.379 1.00 0.00

ATOM 1000 H LYS A 64 4.123 15.592 70.115 1.00 0.00

ATOM 1001 H LYS A 64 3.680 17.174 70.575 1.00 0.00

ATOM 1002 H LYS A 64 5.090 16.462 71.219 1.00 0.00

ATOM 1003 N SER A 65 0.196 18.265 77.593 1.00 61.45

ATOM 1004 CA SER A 65 0.165 18.745 78.977 1.00 59.90

ATOM 1005 C SER A 65 1.499 19.324 79.413 1.00 58.36

ATOM 1006 O SER A 65 2.564 19.037 78.843 1.00 56.95

ATOM 1007 CB SER A 65 -0.198 17.601 79.930 1.00 59.83

ATOM 1008 OG SER A 65 -1.580 17.605 80.252 1.00 62.97

ATOM 1009 H SER A 65 -0.013 17.274 77.407 1.00 0.00

ATOM 1010 H SER A 65 -0.588 19.532 79.017 1.00 0.00

ATOM 1011 H SER A 65 0.368 17.657 80.860 1.00 0.00

ATOM 1012 H SER A 65 -0.027 16.658 79.410 1.00 0.00

ATOM 1013 H SER A 65 -1.743 16.813 80.891 1.00 0.00

ATOM 1014 N LEU A 66 1.464 20.100 80.472 1.00 59.31

ATOM 1015 CA LEU A 66 2.704 20.533 81.066 1.00 60.18

ATOM 1016 C LEU A 66 3.629 19.406 81.589 1.00 58.77

ATOM 1017 O LEU A 66 4.835 19.616 81.642 1.00 59.39

ATOM 1018 CB LEU A 66 2.426 21.617 82.116 1.00 60.96

ATOM 1019 CG LEU A 66 2.185 23.005 81.444 1.00 67.19

ATOM 1020 CD1 LEU A 66 1.592 24.095 82.418 1.00 65.28

ATOM 1021 CD2 LEU A 66 3.502 23.557 80.758 1.00 67.17

ATOM 1022 H LEU A 66 0.562 20.395 80.873 1.00 0.00

ATOM 1023 H LEU A 66 3.294 20.960 80.256 1.00 0.00

ATOM 1024 H LEU A 66 3.301 21.691 82.762 1.00 0.00

ATOM 1025 H LEU A 66 1.540 21.332 82.684 1.00 0.00

ATOM 1026 H LEU A 66 1.429 22.819 80.681 1.00 0.00

ATOM 1027 H LEU A 66 1.470 25.009 81.837 1.00 0.00

ATOM 1028 H LEU A 66 0.637 23.714 82.779 1.00 0.00

ATOM 1029 H LEU A 66 2.310 24.222 83.229 1.00 0.00

ATOM 1030 H LEU A 66 3.251 24.521 80.316 1.00 0.00

ATOM 1031 H LEU A 66 4.253 23.654 81.542 1.00 0.00

ATOM 1032 H LEU A 66 3.797 22.829 80.002 1.00 0.00

ATOM 1033 N HIS A 67 3.101 18.243 81.954 1.00 57.77

ATOM 1034 CA HIS A 67 3.955 17.136 82.474 1.00 59.36

ATOM 1035 C HIS A 67 4.692 16.493 81.326 1.00 59.83

ATOM 1036 O HIS A 67 5.885 16.159 81.432 1.00 59.21

ATOM 1037 CB HIS A 67 3.149 15.959 83.119 1.00 60.80

ATOM 1038 CG HIS A 67 2.345 16.318 84.334 1.00 63.23

ATOM 1039 ND1 HIS A 67 0.988 16.042 84.431 1.00 63.00

ATOM 1040 CD2 HIS A 67 2.702 16.900 85.514 1.00 66.27

ATOM 1041 CE1 HIS A 67 0.540 16.448 85.611 1.00 64.54

ATOM 1042 NE2 HIS A 67 1.552 16.982 86.287 1.00 68.27

ATOM 1043 H HIS A 67 2.083 18.103 81.877 1.00 0.00

ATOM 1044 H HIS A 67 4.596 17.603 83.221 1.00 0.00

ATOM 1045 H HIS A 67 3.854 15.189 83.431 1.00 0.00

ATOM 1046 H HIS A 67 2.412 15.647 82.379 1.00 0.00

ATOM 1047 H HIS A 67 0.418 15.592 83.700 1.00 0.00

ATOM 1048 H HIS A 67 3.700 17.237 85.796 1.00 0.00

ATOM 1049 H HIS A 67 -0.486 16.359 85.967 1.00 0.00

ATOM 1050 N THR A 68 3.935 16.279 80.242 1.00 60.01

ATOM 1051 CA THR A 68 4.444 15.601 79.087 1.00 60.09

ATOM 1052 C THR A 68 5.602 16.376 78.564 1.00 60.97

ATOM 1053 O THR A 68 6.643 15.797 78.307 1.00 61.46

ATOM 1054 CB THR A 68 3.401 15.561 78.012 1.00 61.74

ATOM 1055 OG1 THR A 68 2.173 15.078 78.563 1.00 61.59

ATOM 1056 CG2 THR A 68 3.854 14.754 76.835 1.00 56.05

ATOM 1057 H THR A 68 2.960 16.611 80.239 1.00 0.00

ATOM 1058 H THR A 68 4.731 14.586 79.363 1.00 0.00

ATOM 1059 H THR A 68 3.235 16.570 77.636 1.00 0.00

ATOM 1060 H THR A 68 1.940 15.687 79.360 1.00 0.00

ATOM 1061 H THR A 68 3.045 14.775 76.104 1.00 0.00

ATOM 1062 H THR A 68 4.758 15.228 76.452 1.00 0.00

ATOM 1063 H THR A 68 4.048 13.744 77.197 1.00 0.00

ATOM 1064 N LEU A 69 5.418 17.687 78.382 1.00 61.19

ATOM 1065 CA LEU A 69 6.530 18.520 77.948 1.00 61.99

ATOM 1066 C LEU A 69 7.647 18.585 78.978 1.00 61.65

ATOM 1067 O LEU A 69 8.803 18.388 78.601 1.00 62.14

ATOM 1068 CB LEU A 69 6.103 19.941 77.583 1.00 62.71

ATOM 1069 CG LEU A 69 5.020 20.122 76.545 1.00 63.05

ATOM 1070 CD1 LEU A 69 4.376 21.502 76.774 1.00 64.09

ATOM 1071 CD2 LEU A 69 5.617 19.944 75.155 1.00 63.60

ATOM 1072 H LEU A 69 4.492 18.107 78.548 1.00 0.00

ATOM 1073 H LEU A 69 6.907 18.031 77.049 1.00 0.00

ATOM 1074 H LEU A 69 6.988 20.415 77.158 1.00 0.00

ATOM 1075 H LEU A 69 5.681 20.356 78.498 1.00 0.00

ATOM 1076 H LEU A 69 4.234 19.372 76.632 1.00 0.00

ATOM 1077 H LEU A 69 3.598 21.620 76.020 1.00 0.00

ATOM 1078 H LEU A 69 3.965 21.498 77.783 1.00 0.00

ATOM 1079 H LEU A 69 5.166 22.245 76.661 1.00 0.00

ATOM 1080 H LEU A 69 4.806 20.082 74.440 1.00 0.00

ATOM 1081 H LEU A 69 6.391 20.703 75.041 1.00 0.00

ATOM 1082 H LEU A 69 6.028 18.936 75.112 1.00 0.00

ATOM 1083 N PHE A 70 7.339 18.886 80.250 1.00 60.15

ATOM 1084 CA PHE A 70 8.410 18.789 81.282 1.00 60.78

ATOM 1085 C PHE A 70 9.083 17.413 81.174 1.00 61.01

ATOM 1086 O PHE A 70 10.296 17.322 80.959 1.00 61.95

ATOM 1087 CB PHE A 70 7.892 18.930 82.715 1.00 59.69

ATOM 1088 CG PHE A 70 7.915 20.318 83.232 1.00 64.52

ATOM 1089 CD1 PHE A 70 9.082 20.864 83.749 1.00 64.86

ATOM 1090 CD2 PHE A 70 6.745 21.103 83.235 1.00 68.62

ATOM 1091 CE1 PHE A 70 9.082 22.175 84.254 1.00 66.03

ATOM 1092 CE2 PHE A 70 6.755 22.428 83.717 1.00 67.24

ATOM 1093 CZ PHE A 70 7.910 22.957 84.227 1.00 67.89

ATOM 1094 H PHE A 70 6.385 19.177 80.506 1.00 0.00

ATOM 1095 H PHE A 70 9.099 19.611 81.089 1.00 0.00

ATOM 1096 H PHE A 70 8.521 18.331 83.373 1.00 0.00

ATOM 1097 H PHE A 70 6.844 18.631 82.709 1.00 0.00

ATOM 1098 H PHE A 70 9.999 20.275 83.763 1.00 0.00

ATOM 1099 H PHE A 70 5.815 20.678 82.858 1.00 0.00

ATOM 1100 H PHE A 70 9.999 22.592 84.671 1.00 0.00

ATOM 1101 H PHE A 70 5.846 23.029 83.684 1.00 0.00

ATOM 1102 H PHE A 70 7.923 23.977 84.610 1.00 0.00

ATOM 1103 N GLY A 71 8.277 16.362 81.315 1.00 59.38

ATOM 1104 CA GLY A 71 8.758 15.000 81.208 1.00 61.02

ATOM 1105 C GLY A 71 9.539 14.635 79.977 1.00 62.52

ATOM 1106 O GLY A 71 10.521 13.932 80.071 1.00 64.44

ATOM 1107 H GLY A 71 7.278 16.523 81.507 1.00 0.00

ATOM 1108 H GLY A 71 7.848 14.401 81.166 1.00 0.00

ATOM 1109 H GLY A 71 9.417 14.854 82.064 1.00 0.00

ATOM 1110 N ASP A 72 9.125 15.095 78.808 1.00 63.41

ATOM 1111 CA ASP A 72 9.899 14.813 77.569 1.00 65.46

ATOM 1112 C ASP A 72 11.321 15.256 77.661 1.00 65.69

ATOM 1113 O ASP A 72 12.221 14.539 77.248 1.00 66.26

ATOM 1114 CB ASP A 72 9.248 15.483 76.374 1.00 65.67

ATOM 1115 CG ASP A 72 7.904 14.823 76.008 1.00 68.87

ATOM 1116 OD1 ASP A 72 7.537 13.732 76.570 1.00 72.65

ATOM 1117 OD2 ASP A 72 7.217 15.368 75.131 1.00 72.49

ATOM 1118 H ASP A 72 8.261 15.652 78.755 1.00 0.00

ATOM 1119 H ASP A 72 9.895 13.730 77.445 1.00 0.00

ATOM 1120 H ASP A 72 9.924 15.375 75.526 1.00 0.00

ATOM 1121 H ASP A 72 9.073 16.530 76.622 1.00 0.00

ATOM 1122 N GLU A 73 11.506 16.430 78.265 1.00 65.14

ATOM 1123 CA GLU A 73 12.814 17.023 78.418 1.00 66.99

ATOM 1124 C GLU A 73 13.670 16.323 79.474 1.00 66.94

ATOM 1125 O GLU A 73 14.888 16.240 79.321 1.00 67.76

ATOM 1126 CB GLU A 73 12.705 18.538 78.682 1.00 67.26

ATOM 1127 CG GLU A 73 12.071 19.405 77.511 1.00 72.41

ATOM 1128 CD GLU A 73 12.487 18.982 76.064 1.00 79.38

ATOM 1129 OE1 GLU A 73 13.701 18.750 75.818 1.00 83.47

ATOM 1130 OE2 GLU A 73 11.597 18.894 75.170 1.00 81.39

ATOM 1131 H GLU A 73 10.687 16.932 78.636 1.00 0.00

ATOM 1132 H GLU A 73 13.335 16.880 77.472 1.00 0.00

ATOM 1133 H GLU A 73 13.734 18.880 78.793 1.00 0.00

ATOM 1134 H GLU A 73 12.067 18.650 79.559 1.00 0.00

ATOM 1135 H GLU A 73 12.413 20.431 77.646 1.00 0.00

ATOM 1136 H GLU A 73 10.992 19.261 77.566 1.00 0.00

ATOM 1137 N LEU A 74 13.049 15.793 80.535 1.00 65.86

ATOM 1138 CA LEU A 74 13.849 15.059 81.528 1.00 66.72

ATOM 1139 C LEU A 74 14.386 13.787 80.867 1.00 69.15

ATOM 1140 O LEU A 74 15.508 13.349 81.123 1.00 71.09

ATOM 1141 CB LEU A 74 13.076 14.740 82.806 1.00 64.86

ATOM 1142 CG LEU A 74 12.477 15.806 83.783 1.00 63.25

ATOM 1143 CD1 LEU A 74 11.804 15.187 84.996 1.00 57.22

ATOM 1144 CD2 LEU A 74 13.498 16.776 84.301 1.00 64.14

ATOM 1145 H LEU A 74 12.031 15.897 80.654 1.00 0.00

ATOM 1146 H LEU A 74 14.673 15.697 81.846 1.00 0.00

ATOM 1147 H LEU A 74 13.854 14.279 83.415 1.00 0.00

ATOM 1148 H LEU A 74 12.196 14.225 82.422 1.00 0.00

ATOM 1149 H LEU A 74 11.744 16.329 83.169 1.00 0.00

ATOM 1150 H LEU A 74 11.425 16.008 85.604 1.00 0.00

ATOM 1151 H LEU A 74 10.999 14.553 84.625 1.00 0.00

ATOM 1152 H LEU A 74 12.564 14.610 85.522 1.00 0.00

ATOM 1153 H LEU A 74 12.975 17.465 84.965 1.00 0.00

ATOM 1154 H LEU A 74 14.250 16.193 84.833 1.00 0.00

ATOM 1155 H LEU A 74 13.919 17.288 83.436 1.00 0.00

ATOM 1156 N CYS A 75 13.599 13.206 79.982 1.00 69.40

ATOM 1157 CA CYS A 75 13.996 11.954 79.379 1.00 72.16

ATOM 1158 C CYS A 75 15.190 12.076 78.458 1.00 75.05

ATOM 1159 O CYS A 75 15.948 11.124 78.311 1.00 76.47

ATOM 1160 CB CYS A 75 12.800 11.306 78.702 1.00 71.31

ATOM 1161 SG CYS A 75 11.809 10.615 80.027 1.00 73.53

ATOM 1162 H CYS A 75 12.704 13.644 79.721 1.00 0.00

ATOM 1163 H CYS A 75 14.339 11.299 80.180 1.00 0.00

ATOM 1164 H CYS A 75 13.216 10.514 78.079 1.00 0.00

ATOM 1165 H CYS A 75 12.323 12.003 78.013 1.00 0.00

ATOM 1166 N LYS A 76 15.388 13.264 77.882 1.00 75.89

ATOM 1167 CA LYS A 76 16.525 13.476 76.977 1.00 79.05

ATOM 1168 C LYS A 76 17.863 13.780 77.704 1.00 80.50

ATOM 1169 O LYS A 76 18.864 14.068 77.057 1.00 82.67

ATOM 1170 CB LYS A 76 16.198 14.580 75.965 1.00 78.58

ATOM 1171 CG LYS A 76 14.823 14.490 75.364 1.00 77.67

ATOM 1172 CD LYS A 76 14.811 14.766 73.852 1.00 81.52

ATOM 1173 CE LYS A 76 15.377 16.167 73.421 1.00 84.04

ATOM 1174 NZ LYS A 76 15.005 17.317 74.326 1.00 83.00

ATOM 1175 H LYS A 76 14.737 14.039 78.074 1.00 0.00

ATOM 1176 H LYS A 76 16.679 12.530 76.457 1.00 0.00

ATOM 1177 H LYS A 76 16.910 14.501 75.143 1.00 0.00

ATOM 1178 H LYS A 76 16.232 15.526 76.505 1.00 0.00

ATOM 1179 H LYS A 76 14.216 15.256 75.847 1.00 0.00

ATOM 1180 H LYS A 76 14.450 13.481 75.538 1.00 0.00

ATOM 1181 H LYS A 76 13.762 14.749 73.557 1.00 0.00

ATOM 1182 H LYS A 76 15.436 14.002 73.390 1.00 0.00

ATOM 1183 H LYS A 76 14.984 16.408 72.433 1.00 0.00

ATOM 1184 H LYS A 76 16.463 16.098 73.484 1.00 0.00

ATOM 1185 H LYS A 76 15.437 18.164 73.930 1.00 0.00

ATOM 1186 H LYS A 76 13.977 17.377 74.329 1.00 0.00

ATOM 1187 H LYS A 76 15.378 17.098 75.261 1.00 0.00

ATOM 1188 N VAL A 77 17.886 13.728 79.032 1.00 79.15

ATOM 1189 CA VAL A 77 19.113 14.077 79.774 1.00 80.86

ATOM 1190 C VAL A 77 20.128 12.967 79.669 1.00 84.52

ATOM 1191 O VAL A 77 19.900 11.864 80.182 1.00 85.71

ATOM 1192 CB VAL A 77 18.862 14.383 81.287 1.00 78.35

ATOM 1193 CG1 VAL A 77 20.178 14.711 81.991 1.00 79.95

ATOM 1194 CG2 VAL A 77 17.900 15.527 81.458 1.00 74.20

ATOM 1195 H VAL A 77 17.041 13.442 79.547 1.00 0.00

ATOM 1196 H VAL A 77 19.487 14.990 79.310 1.00 0.00

ATOM 1197 H VAL A 77 18.425 13.492 81.737 1.00 0.00

ATOM 1198 H VAL A 77 19.939 14.914 83.035 1.00 0.00

ATOM 1199 H VAL A 77 20.822 13.838 81.885 1.00 0.00

ATOM 1200 H VAL A 77 20.596 15.586 81.494 1.00 0.00

ATOM 1201 H VAL A 77 17.775 15.681 82.530 1.00 0.00

ATOM 1202 H VAL A 77 18.347 16.395 80.973 1.00 0.00

ATOM 1203 H VAL A 77 16.966 15.232 80.979 1.00 0.00

ATOM 1204 N ALA A 78 21.257 13.260 79.029 1.00 88.01

ATOM 1205 CA ALA A 78 22.243 12.229 78.658 1.00 91.84

ATOM 1206 C ALA A 78 22.621 11.352 79.835 1.00 92.89

ATOM 1207 O ALA A 78 22.998 10.186 79.664 1.00 95.89

ATOM 1208 CB ALA A 78 23.498 12.869 78.053 1.00 95.08

ATOM 1209 H ALA A 78 21.449 14.242 78.784 1.00 0.00

ATOM 1210 H ALA A 78 21.771 11.593 77.909 1.00 0.00

ATOM 1211 H ALA A 78 24.181 12.057 77.803 1.00 0.00

ATOM 1212 H ALA A 78 23.180 13.419 77.167 1.00 0.00

ATOM 1213 H ALA A 78 23.916 13.530 78.812 1.00 0.00

ATOM 1214 N SER A 79 22.508 11.912 81.034 1.00 91.10

ATOM 1215 CA SER A 79 23.083 11.289 82.226 1.00 92.50

ATOM 1216 C SER A 79 22.114 10.813 83.314 1.00 90.27

ATOM 1217 O SER A 79 22.543 10.548 84.438 1.00 91.53

ATOM 1218 CB SER A 79 24.027 12.257 82.917 1.00 92.87

ATOM 1219 OG SER A 79 25.327 12.063 82.373 1.00 97.24

ATOM 1220 H SER A 79 22.004 12.805 81.127 1.00 0.00

ATOM 1221 H SER A 79 23.560 10.400 81.813 1.00 0.00

ATOM 1222 H SER A 79 24.050 12.128 83.999 1.00 0.00

ATOM 1223 H SER A 79 23.730 13.285 82.708 1.00 0.00

ATOM 1224 H SER A 79 25.955 12.725 82.850 1.00 0.00

ATOM 1225 N LEU A 80 20.837 10.667 82.965 1.00 88.15

ATOM 1226 CA LEU A 80 19.773 10.167 83.873 1.00 86.16

ATOM 1227 C LEU A 80 20.123 8.803 84.554 1.00 88.44

ATOM 1228 O LEU A 80 20.185 8.720 85.795 1.00 88.01

ATOM 1229 CB LEU A 80 18.459 10.086 83.072 1.00 83.91

ATOM 1230 CG LEU A 80 17.149 10.332 83.811 1.00 81.19

ATOM 1231 CD1 LEU A 80 17.201 11.650 84.599 1.00 77.46

ATOM 1232 CD2 LEU A 80 16.011 10.340 82.809 1.00 77.40

ATOM 1233 H LEU A 80 20.570 10.917 82.002 1.00 0.00

ATOM 1234 H LEU A 80 19.669 10.867 84.702 1.00 0.00

ATOM 1235 H LEU A 80 18.396 9.060 82.709 1.00 0.00

ATOM 1236 H LEU A 80 18.520 10.889 82.338 1.00 0.00

ATOM 1237 H LEU A 80 16.986 9.532 84.533 1.00 0.00

ATOM 1238 H LEU A 80 16.239 11.761 85.099 1.00 0.00

ATOM 1239 H LEU A 80 18.020 11.563 85.312 1.00 0.00

ATOM 1240 H LEU A 80 17.376 12.447 83.876 1.00 0.00

ATOM 1241 H LEU A 80 15.094 10.518 83.370 1.00 0.00

ATOM 1242 H LEU A 80 16.215 11.143 82.101 1.00 0.00

ATOM 1243 H LEU A 80 16.007 9.364 82.324 1.00 0.00

ATOM 1244 N ARG A 81 20.361 7.750 83.764 1.00 90.58

ATOM 1245 CA ARG A 81 20.718 6.444 84.323 1.00 92.74

ATOM 1246 C ARG A 81 21.981 6.524 85.188 1.00 95.64

ATOM 1247 O ARG A 81 21.901 6.420 86.423 1.00 95.15

ATOM 1248 CB ARG A 81 20.884 5.380 83.222 1.00 95.23

ATOM 1249 CG ARG A 81 21.120 3.957 83.747 1.00 95.31

ATOM 1250 CD ARG A 81 21.391 2.981 82.622 1.00 94.67

ATOM 1251 NE ARG A 81 22.221 1.856 83.052 1.00 94.83

ATOM 1252 CZ ARG A 81 23.492 1.692 82.700 1.00 96.69

ATOM 1253 NH1 ARG A 81 24.077 2.576 81.910 1.00 95.20

ATOM 1254 NH2 ARG A 81 24.174 0.644 83.137 1.00 99.55

ATOM 1255 H ARG A 81 20.292 7.860 82.742 1.00 0.00

ATOM 1256 H ARG A 81 19.890 6.140 84.963 1.00 0.00

ATOM 1257 H ARG A 81 21.762 5.655 82.637 1.00 0.00

ATOM 1258 H ARG A 81 19.954 5.359 82.654 1.00 0.00

ATOM 1259 H ARG A 81 20.230 3.625 84.281 1.00 0.00

ATOM 1260 H ARG A 81 21.998 3.971 84.393 1.00 0.00

ATOM 1261 H ARG A 81 21.910 3.493 81.812 1.00 0.00

ATOM 1262 H ARG A 81 20.437 2.562 82.302 1.00 0.00

ATOM 1263 H ARG A 81 21.794 1.147 83.665 1.00 0.00

ATOM 1264 H ARG A 81 25.062 2.450 81.637 1.00 0.00

ATOM 1265 H ARG A 81 23.548 3.391 81.568 1.00 0.00

ATOM 1266 H ARG A 81 25.159 0.520 82.862 1.00 0.00

ATOM 1267 H ARG A 81 23.719 -0.047 83.751 1.00 0.00

ATOM 1268 N GLU A 82 23.139 6.715 84.550 1.00 99.24

ATOM 1269 CA GLU A 82 24.430 6.616 85.247 1.00103.48

ATOM 1270 C GLU A 82 24.603 7.713 86.288 1.00102.24

ATOM 1271 O GLU A 82 25.640 7.791 86.951 1.00104.66

ATOM 1272 CB GLU A 82 25.601 6.613 84.261 1.00107.19

ATOM 1273 CG GLU A 82 25.775 7.919 83.479 1.00107.24

ATOM 1274 CD GLU A 82 26.750 7.774 82.318 1.00111.64

ATOM 1275 OE1 GLU A 82 27.850 7.194 82.522 1.00116.16

ATOM 1276 OE2 GLU A 82 26.407 8.235 81.200 1.00109.31

ATOM 1277 H GLU A 82 23.127 6.937 83.544 1.00 0.00

ATOM 1278 H GLU A 82 24.429 5.662 85.774 1.00 0.00

ATOM 1279 H GLU A 82 25.401 5.828 83.532 1.00 0.00

ATOM 1280 H GLU A 82 26.510 6.466 84.845 1.00 0.00

ATOM 1281 H GLU A 82 26.169 8.675 84.158 1.00 0.00

ATOM 1282 H GLU A 82 24.805 8.197 83.067 1.00 0.00

ATOM 1283 N THR A 83 23.578 8.554 86.413 1.00 98.67

ATOM 1284 CA THR A 83 23.516 9.564 87.476 1.00 97.33

ATOM 1285 C THR A 83 22.385 9.348 88.484 1.00 94.27

ATOM 1286 O THR A 83 22.573 9.661 89.660 1.00 93.78

ATOM 1287 CB THR A 83 23.469 11.016 86.906 1.00 95.88

ATOM 1288 OG1 THR A 83 24.677 11.265 86.157 1.00100.50

ATOM 1289 CG2 THR A 83 23.352 12.041 88.034 1.00 93.49

ATOM 1290 H THR A 83 22.801 8.493 85.740 1.00 0.00

ATOM 1291 H THR A 83 24.447 9.435 88.028 1.00 0.00

ATOM 1292 H THR A 83 22.596 11.112 86.261 1.00 0.00

ATOM 1293 H THR A 83 24.719 10.557 85.410 1.00 0.00

ATOM 1294 H THR A 83 23.324 13.026 87.568 1.00 0.00

ATOM 1295 H THR A 83 22.429 11.821 88.570 1.00 0.00

ATOM 1296 H THR A 83 24.229 11.918 88.669 1.00 0.00

ATOM 1297 N TYR A 84 21.238 8.806 88.025 1.00 92.69

ATOM 1298 CA TYR A 84 19.999 8.642 88.854 1.00 89.42

ATOM 1299 C TYR A 84 19.401 7.211 88.935 1.00 89.91

ATOM 1300 O TYR A 84 18.282 7.039 89.453 1.00 87.75

ATOM 1301 CB TYR A 84 18.901 9.633 88.413 1.00 85.83

ATOM 1302 CG TYR A 84 19.262 11.100 88.575 1.00 84.75

ATOM 1303 CD1 TYR A 84 18.907 11.818 89.723 1.00 83.32

ATOM 1304 CD2 TYR A 84 19.939 11.775 87.568 1.00 86.57

ATOM 1305 CE1 TYR A 84 19.251 13.184 89.867 1.00 83.99

ATOM 1306 CE2 TYR A 84 20.287 13.128 87.697 1.00 86.94

ATOM 1307 CZ TYR A 84 19.958 13.832 88.837 1.00 84.10

ATOM 1308 OH TYR A 84 20.315 15.170 88.901 1.00 80.01

ATOM 1309 H TYR A 84 21.212 8.486 87.046 1.00 0.00

ATOM 1310 H TYR A 84 20.346 8.860 89.864 1.00 0.00

ATOM 1311 H TYR A 84 18.026 9.452 89.037 1.00 0.00

ATOM 1312 H TYR A 84 18.743 9.475 87.346 1.00 0.00

ATOM 1313 H TYR A 84 18.357 11.317 90.519 1.00 0.00

ATOM 1314 H TYR A 84 20.206 11.241 86.656 1.00 0.00

ATOM 1315 H TYR A 84 18.971 13.729 90.768 1.00 0.00

ATOM 1316 H TYR A 84 20.822 13.627 86.889 1.00 0.00

ATOM 1317 H TYR A 84 19.967 15.532 89.801 1.00 0.00

ATOM 1318 N GLY A 85 20.136 6.213 88.419 1.00 92.44

ATOM 1319 CA GLY A 85 19.699 4.811 88.392 1.00 92.99

ATOM 1320 C GLY A 85 18.265 4.586 87.947 1.00 90.64

ATOM 1321 O GLY A 85 17.836 5.162 86.941 1.00 88.77

ATOM 1322 H GLY A 85 21.057 6.443 88.020 1.00 0.00

ATOM 1323 H GLY A 85 19.761 4.472 89.426 1.00 0.00

ATOM 1324 H GLY A 85 20.355 4.294 87.692 1.00 0.00

ATOM 1325 N ASP A 86 17.533 3.758 88.718 1.00 90.85

ATOM 1326 CA ASP A 86 16.123 3.324 88.462 1.00 88.72

ATOM 1327 C ASP A 86 15.184 4.448 87.947 1.00 84.68

ATOM 1328 O ASP A 86 14.172 4.169 87.314 1.00 83.68

ATOM 1329 CB ASP A 86 15.518 2.551 89.680 1.00 88.65

ATOM 1330 CG ASP A 86 14.906 3.492 90.783 1.00 88.69

ATOM 1331 OD1 ASP A 86 15.603 3.754 91.787 1.00 87.82

ATOM 1332 OD2 ASP A 86 13.724 3.975 90.658 1.00 87.62

ATOM 1333 H ASP A 86 17.987 3.388 89.565 1.00 0.00

ATOM 1334 H ASP A 86 16.190 2.624 87.629 1.00 0.00

ATOM 1335 H ASP A 86 16.331 1.992 90.142 1.00 0.00

ATOM 1336 H ASP A 86 14.715 1.920 89.300 1.00 0.00

ATOM 1337 N MET A 87 15.555 5.701 88.183 1.00 82.39

ATOM 1338 CA MET A 87 14.801 6.852 87.643 1.00 79.91

ATOM 1339 C MET A 87 14.578 6.877 86.091 1.00 79.11

ATOM 1340 O MET A 87 13.514 7.295 85.612 1.00 76.80

ATOM 1341 CB MET A 87 15.418 8.159 88.170 1.00 78.80

ATOM 1342 CG MET A 87 14.811 9.468 87.603 1.00 79.75

ATOM 1343 SD MET A 87 15.140 10.928 88.651 1.00 77.70

ATOM 1344 CE MET A 87 13.913 12.074 88.002 1.00 75.83

ATOM 1345 H MET A 87 16.391 5.876 88.758 1.00 0.00

ATOM 1346 H MET A 87 13.783 6.737 88.016 1.00 0.00

ATOM 1347 H MET A 87 16.463 8.145 87.861 1.00 0.00

ATOM 1348 H MET A 87 15.241 8.171 89.246 1.00 0.00

ATOM 1349 H MET A 87 13.744 9.260 87.681 1.00 0.00

ATOM 1350 H MET A 87 15.158 9.554 86.573 1.00 0.00

ATOM 1351 H MET A 87 14.016 13.003 88.563 1.00 0.00

ATOM 1352 H MET A 87 14.135 12.213 86.944 1.00 0.00

ATOM 1353 H MET A 87 12.936 11.615 88.154 1.00 0.00

ATOM 1354 N ALA A 88 15.564 6.411 85.325 1.00 81.12

ATOM 1355 CA ALA A 88 15.552 6.464 83.852 1.00 80.77

ATOM 1356 C ALA A 88 14.656 5.385 83.190 1.00 81.42

ATOM 1357 O ALA A 88 14.547 5.317 81.970 1.00 81.85

ATOM 1358 CB ALA A 88 16.967 6.354 83.349 1.00 84.19

ATOM 1359 H ALA A 88 16.381 5.990 85.789 1.00 0.00

ATOM 1360 H ALA A 88 15.112 7.420 83.569 1.00 0.00

ATOM 1361 H ALA A 88 16.923 6.396 82.261 1.00 0.00

ATOM 1362 H ALA A 88 17.520 7.196 83.765 1.00 0.00

ATOM 1363 H ALA A 88 17.357 5.399 83.702 1.00 0.00

ATOM 1364 N ASP A 89 14.048 4.530 84.017 1.00 80.82

ATOM 1365 CA ASP A 89 12.973 3.636 83.615 1.00 79.94

ATOM 1366 C ASP A 89 11.604 4.331 83.713 1.00 76.86

ATOM 1367 O ASP A 89 10.609 3.799 83.219 1.00 76.47

ATOM 1368 CB ASP A 89 12.981 2.412 84.524 1.00 81.79

ATOM 1369 CG ASP A 89 14.353 1.792 84.645 1.00 86.24

ATOM 1370 OD1 ASP A 89 15.059 1.621 83.625 1.00 87.94

ATOM 1371 OD2 ASP A 89 14.747 1.467 85.775 1.00 92.24

ATOM 1372 H ASP A 89 14.359 4.502 84.998 1.00 0.00

ATOM 1373 H ASP A 89 13.134 3.344 82.577 1.00 0.00

ATOM 1374 H ASP A 89 12.310 1.665 84.100 1.00 0.00

ATOM 1375 H ASP A 89 12.674 2.730 85.520 1.00 0.00

ATOM 1376 N CYS A 90 11.545 5.491 84.382 1.00 73.89

ATOM 1377 CA CYS A 90 10.369 6.349 84.312 1.00 71.31

ATOM 1378 C CYS A 90 10.134 6.761 82.866 1.00 70.63

ATOM 1379 O CYS A 90 9.002 6.930 82.441 1.00 69.01

ATOM 1380 CB CYS A 90 10.540 7.612 85.141 1.00 69.18

ATOM 1381 SG CYS A 90 10.723 7.449 86.942 1.00 72.67

ATOM 1382 H CYS A 90 12.348 5.782 84.958 1.00 0.00

ATOM 1383 H CYS A 90 9.525 5.783 84.706 1.00 0.00

ATOM 1384 H CYS A 90 9.536 8.018 85.016 1.00 0.00

ATOM 1385 H CYS A 90 11.373 8.128 84.662 1.00 0.00

ATOM 1386 N CYS A 91 11.226 6.890 82.117 1.00 71.47

ATOM 1387 CA CYS A 91 11.176 7.243 80.727 1.00 71.74

ATOM 1388 C CYS A 91 10.544 6.183 79.835 1.00 72.74

ATOM 1389 O CYS A 91 10.390 6.426 78.639 1.00 73.30

ATOM 1390 CB CYS A 91 12.584 7.591 80.223 1.00 73.47

ATOM 1391 SG CYS A 91 13.158 9.204 80.878 1.00 76.18

ATOM 1392 H CYS A 91 12.145 6.730 82.553 1.00 0.00

ATOM 1393 H CYS A 91 10.523 8.113 80.661 1.00 0.00

ATOM 1394 H CYS A 91 12.411 7.717 79.154 1.00 0.00

ATOM 1395 H CYS A 91 13.225 6.734 80.429 1.00 0.00

ATOM 1396 N GLU A 92 10.193 5.025 80.400 1.00 73.05

ATOM 1397 CA GLU A 92 9.618 3.905 79.649 1.00 74.55

ATOM 1398 C GLU A 92 8.133 3.893 79.823 1.00 72.33

ATOM 1399 O GLU A 92 7.438 3.085 79.207 1.00 73.55

ATOM 1400 CB GLU A 92 10.097 2.533 80.164 1.00 77.32

ATOM 1401 CG GLU A 92 11.573 2.342 80.378 1.00 81.99

ATOM 1402 CD GLU A 92 12.389 2.671 79.153 1.00 87.32

ATOM 1403 OE1 GLU A 92 13.057 1.747 78.609 1.00 89.51

ATOM 1404 OE2 GLU A 92 12.379 3.869 78.753 1.00 87.73

ATOM 1405 H GLU A 92 10.333 4.913 81.414 1.00 0.00

ATOM 1406 H GLU A 92 9.930 4.049 78.615 1.00 0.00

ATOM 1407 H GLU A 92 9.816 1.801 79.407 1.00 0.00

ATOM 1408 H GLU A 92 9.663 2.428 81.158 1.00 0.00

ATOM 1409 H GLU A 92 11.740 1.292 80.618 1.00 0.00

ATOM 1410 H GLU A 92 11.884 3.013 81.179 1.00 0.00

ATOM 1411 N LYS A 93 7.652 4.781 80.673 1.00 69.15

ATOM 1412 CA LYS A 93 6.252 4.829 81.019 1.00 67.30

ATOM 1413 C LYS A 93 5.513 5.923 80.235 1.00 66.14

ATOM 1414 O LYS A 93 6.112 6.855 79.679 1.00 63.62

ATOM 1415 CB LYS A 93 6.110 5.101 82.521 1.00 67.05

ATOM 1416 CG LYS A 93 6.818 4.096 83.483 1.00 66.89

ATOM 1417 CD LYS A 93 6.796 4.639 84.928 1.00 65.26

ATOM 1418 CE LYS A 93 7.157 3.535 85.955 1.00 65.10

ATOM 1419 NZ LYS A 93 6.220 2.388 85.720 1.00 66.87

ATOM 1420 H LYS A 93 8.296 5.461 81.103 1.00 0.00

ATOM 1421 H LYS A 93 5.807 3.868 80.762 1.00 0.00

ATOM 1422 H LYS A 93 5.043 5.015 82.729 1.00 0.00

ATOM 1423 H LYS A 93 6.564 6.077 82.694 1.00 0.00

ATOM 1424 H LYS A 93 7.852 3.956 83.169 1.00 0.00

ATOM 1425 H LYS A 93 6.287 3.144 83.459 1.00 0.00

ATOM 1426 H LYS A 93 5.789 4.996 85.144 1.00 0.00

ATOM 1427 H LYS A 93 7.529 5.442 85.007 1.00 0.00

ATOM 1428 H LYS A 93 7.077 3.891 86.982 1.00 0.00

ATOM 1429 H LYS A 93 8.183 3.198 85.811 1.00 0.00

ATOM 1430 H LYS A 93 6.462 1.656 86.403 1.00 0.00

ATOM 1431 H LYS A 93 5.265 2.745 85.864 1.00 0.00

ATOM 1432 H LYS A 93 6.369 2.071 84.751 1.00 0.00

ATOM 1433 N GLN A 94 4.196 5.791 80.218 1.00 66.12

ATOM 1434 CA GLN A 94 3.311 6.807 79.684 1.00 65.54

ATOM 1435 C GLN A 94 2.866 7.786 80.771 1.00 64.52

ATOM 1436 O GLN A 94 3.019 7.524 81.985 1.00 63.04

ATOM 1437 CB GLN A 94 2.052 6.186 79.097 1.00 66.45

ATOM 1438 CG GLN A 94 2.230 5.140 78.050 1.00 69.70

ATOM 1439 CD GLN A 94 0.924 4.403 77.891 1.00 75.53

ATOM 1440 OE1 GLN A 94 -0.122 4.910 78.361 1.00 73.91

ATOM 1441 NE2 GLN A 94 0.955 3.205 77.250 1.00 71.37

ATOM 1442 H GLN A 94 3.782 4.929 80.601 1.00 0.00

ATOM 1443 H GLN A 94 3.876 7.329 78.912 1.00 0.00

ATOM 1444 H GLN A 94 1.501 6.994 78.616 1.00 0.00

ATOM 1445 H GLN A 94 1.563 5.672 79.925 1.00 0.00

ATOM 1446 H GLN A 94 3.015 4.448 78.354 1.00 0.00

ATOM 1447 H GLN A 94 2.526 5.601 77.108 1.00 0.00

ATOM 1448 H GLN A 94 0.086 2.668 77.120 1.00 0.00

ATOM 1449 H GLN A 94 1.848 2.834 76.895 1.00 0.00

ATOM 1450 N GLU A 95 2.296 8.907 80.301 1.00 63.87

ATOM 1451 CA GLU A 95 1.675 9.874 81.184 1.00 64.00

ATOM 1452 C GLU A 95 0.360 9.292 81.701 1.00 64.54

ATOM 1453 O GLU A 95 -0.299 8.610 80.937 1.00 66.17

ATOM 1454 CB GLU A 95 1.517 11.212 80.454 1.00 63.52

ATOM 1455 CG GLU A 95 2.823 12.042 80.421 1.00 64.41

ATOM 1456 CD GLU A 95 3.478 12.164 81.813 1.00 72.09

ATOM 1457 OE1 GLU A 95 4.556 12.814 81.931 1.00 73.88

ATOM 1458 OE2 GLU A 95 2.912 11.606 82.809 1.00 71.82

ATOM 1459 H GLU A 95 2.300 9.084 79.286 1.00 0.00

ATOM 1460 H GLU A 95 2.298 10.078 82.055 1.00 0.00

ATOM 1461 H GLU A 95 0.770 11.791 80.997 1.00 0.00

ATOM 1462 H GLU A 95 1.226 10.997 79.426 1.00 0.00

ATOM 1463 H GLU A 95 2.578 13.046 80.074 1.00 0.00

ATOM 1464 H GLU A 95 3.528 11.535 79.762 1.00 0.00

ATOM 1465 N PRO A 96 -0.004 9.502 83.000 1.00 64.28

ATOM 1466 CA PRO A 96 0.655 10.212 84.141 1.00 62.03

ATOM 1467 C PRO A 96 1.591 9.343 85.024 1.00 61.33

ATOM 1468 O PRO A 96 2.170 9.866 85.968 1.00 59.17

ATOM 1469 CB PRO A 96 -0.538 10.649 85.006 1.00 62.30

ATOM 1470 CG PRO A 96 -1.509 9.521 84.894 1.00 64.12

ATOM 1471 CD PRO A 96 -1.317 8.951 83.429 1.00 65.41

ATOM 1472 H PRO A 96 1.300 11.000 83.753 1.00 0.00

ATOM 1473 H PRO A 96 -0.973 11.597 84.690 1.00 0.00

ATOM 1474 H PRO A 96 -0.252 10.816 86.044 1.00 0.00

ATOM 1475 H PRO A 96 -2.520 9.898 85.048 1.00 0.00

ATOM 1476 H PRO A 96 -1.356 8.782 85.680 1.00 0.00

ATOM 1477 H PRO A 96 -1.287 7.862 83.411 1.00 0.00

ATOM 1478 H PRO A 96 -2.117 9.202 82.733 1.00 0.00

ATOM 1479 N GLU A 97 1.700 8.043 84.736 1.00 62.55

ATOM 1480 CA GLU A 97 2.612 7.145 85.435 1.00 63.62

ATOM 1481 C GLU A 97 4.073 7.601 85.375 1.00 62.68

ATOM 1482 O GLU A 97 4.822 7.486 86.341 1.00 61.59

ATOM 1483 CB GLU A 97 2.525 5.739 84.871 1.00 66.33

ATOM 1484 CG GLU A 97 1.207 5.010 85.166 1.00 70.87

ATOM 1485 CD GLU A 97 0.180 5.095 83.989 1.00 77.13

ATOM 1486 OE1 GLU A 97 -0.894 4.423 84.116 1.00 81.16

ATOM 1487 OE2 GLU A 97 0.448 5.783 82.959 1.00 72.08

ATOM 1488 H GLU A 97 1.112 7.656 83.984 1.00 0.00

ATOM 1489 H GLU A 97 2.294 7.160 86.478 1.00 0.00

ATOM 1490 H GLU A 97 3.319 5.165 85.349 1.00 0.00

ATOM 1491 H GLU A 97 2.614 5.826 83.788 1.00 0.00

ATOM 1492 H GLU A 97 0.760 5.501 86.030 1.00 0.00

ATOM 1493 H GLU A 97 1.442 3.961 85.344 1.00 0.00

ATOM 1494 N ARG A 98 4.476 8.168 84.249 1.00 62.36

ATOM 1495 CA ARG A 98 5.831 8.624 84.154 1.00 61.34

ATOM 1496 C ARG A 98 6.083 9.796 85.104 1.00 60.09

ATOM 1497 O ARG A 98 7.109 9.854 85.750 1.00 61.41

ATOM 1498 CB ARG A 98 6.137 8.964 82.724 1.00 62.28

ATOM 1499 CG ARG A 98 7.556 9.384 82.488 1.00 62.14

ATOM 1500 CD ARG A 98 7.749 9.677 81.027 1.00 57.70

ATOM 1501 NE ARG A 98 7.148 10.944 80.743 1.00 60.42

ATOM 1502 CZ ARG A 98 7.401 11.666 79.657 1.00 63.16

ATOM 1503 NH1 ARG A 98 8.296 11.208 78.768 1.00 59.18

ATOM 1504 NH2 ARG A 98 6.778 12.858 79.500 1.00 59.78

ATOM 1505 H ARG A 98 3.828 8.279 83.456 1.00 0.00

ATOM 1506 H ARG A 98 6.510 7.830 84.466 1.00 0.00

ATOM 1507 H ARG A 98 5.501 9.804 82.446 1.00 0.00

ATOM 1508 H ARG A 98 5.965 8.064 82.134 1.00 0.00

ATOM 1509 H ARG A 98 8.228 8.581 82.792 1.00 0.00

ATOM 1510 H ARG A 98 7.775 10.277 83.074 1.00 0.00

ATOM 1511 H ARG A 98 7.313 8.906 80.393 1.00 0.00

ATOM 1512 H ARG A 98 8.812 9.745 80.797 1.00 0.00

ATOM 1513 H ARG A 98 6.475 11.321 81.425 1.00 0.00

ATOM 1514 H ARG A 98 8.505 11.754 77.920 1.00 0.00

ATOM 1515 H ARG A 98 8.774 10.311 78.934 1.00 0.00

ATOM 1516 H ARG A 98 6.963 13.430 78.664 1.00 0.00

ATOM 1517 H ARG A 98 6.119 13.193 80.217 1.00 0.00

ATOM 1518 N ASN A 99 5.134 10.697 85.251 1.00 58.29

ATOM 1519 CA ASN A 99 5.300 11.749 86.212 1.00 57.45

ATOM 1520 C ASN A 99 5.278 11.303 87.683 1.00 58.29

ATOM 1521 O ASN A 99 6.125 11.745 88.486 1.00 58.82

ATOM 1522 CB ASN A 99 4.324 12.921 85.921 1.00 57.62

ATOM 1523 CG ASN A 99 4.682 14.185 86.697 1.00 57.78

ATOM 1524 OD1 ASN A 99 5.805 14.693 86.602 1.00 58.71

ATOM 1525 ND2 ASN A 99 3.750 14.643 87.554 1.00 60.39

ATOM 1526 H ASN A 99 4.278 10.647 84.680 1.00 0.00

ATOM 1527 H ASN A 99 6.320 12.110 86.078 1.00 0.00

ATOM 1528 H ASN A 99 3.318 12.620 86.214 1.00 0.00

ATOM 1529 H ASN A 99 4.394 13.162 84.860 1.00 0.00

ATOM 1530 H ASN A 99 3.932 15.493 88.107 1.00 0.00

ATOM 1531 H ASN A 99 2.855 14.144 87.656 1.00 0.00

ATOM 1532 N GLU A 100 4.373 10.397 88.054 1.00 59.76

ATOM 1533 CA GLU A 100 4.370 9.836 89.423 1.00 60.56

ATOM 1534 C GLU A 100 5.710 9.296 89.840 1.00 59.92

ATOM 1535 O GLU A 100 6.108 9.373 90.999 1.00 59.78

ATOM 1536 CB GLU A 100 3.377 8.679 89.571 1.00 62.45

ATOM 1537 CG GLU A 100 1.971 9.045 89.216 1.00 67.19

ATOM 1538 CD GLU A 100 1.385 10.123 90.128 1.00 74.27

ATOM 1539 OE1 GLU A 100 2.107 10.663 91.024 1.00 78.09

ATOM 1540 OE2 GLU A 100 0.184 10.442 89.933 1.00 76.78

ATOM 1541 H GLU A 100 3.664 10.082 87.376 1.00 0.00

ATOM 1542 H GLU A 100 4.089 10.679 90.055 1.00 0.00

ATOM 1543 H GLU A 100 3.377 8.356 90.612 1.00 0.00

ATOM 1544 H GLU A 100 3.678 7.900 88.871 1.00 0.00

ATOM 1545 H GLU A 100 1.370 8.144 89.339 1.00 0.00

ATOM 1546 H GLU A 100 1.974 9.419 88.192 1.00 0.00

ATOM 1547 N CYS A 101 6.367 8.722 88.869 1.00 60.01

ATOM 1548 CA CYS A 101 7.586 7.976 89.057 1.00 61.84

ATOM 1549 C CYS A 101 8.731 8.932 89.346 1.00 59.95

ATOM 1550 O CYS A 101 9.421 8.728 90.271 1.00 60.80

ATOM 1551 CB CYS A 101 7.798 7.174 87.767 1.00 63.80

ATOM 1552 SG CYS A 101 9.234 6.210 87.615 1.00 70.85

ATOM 1553 H CYS A 101 5.993 8.807 87.913 1.00 0.00

ATOM 1554 H CYS A 101 7.536 7.297 89.908 1.00 0.00

ATOM 1555 H CYS A 101 7.940 7.988 87.057 1.00 0.00

ATOM 1556 H CYS A 101 6.904 6.556 87.680 1.00 0.00

ATOM 1557 N PHE A 102 8.874 10.007 88.571 1.00 59.15

ATOM 1558 CA PHE A 102 9.807 11.112 88.848 1.00 58.03

ATOM 1559 C PHE A 102 9.698 11.733 90.253 1.00 57.81

ATOM 1560 O PHE A 102 10.694 11.734 91.002 1.00 58.60

ATOM 1561 CB PHE A 102 9.671 12.226 87.816 1.00 55.75

ATOM 1562 CG PHE A 102 10.083 11.845 86.432 1.00 56.87

ATOM 1563 CD1 PHE A 102 11.340 11.361 86.167 1.00 55.75

ATOM 1564 CD2 PHE A 102 9.217 12.038 85.371 1.00 60.08

ATOM 1565 CE1 PHE A 102 11.713 10.997 84.906 1.00 59.11

ATOM 1566 CE2 PHE A 102 9.591 11.683 84.105 1.00 60.56

ATOM 1567 CZ PHE A 102 10.868 11.151 83.883 1.00 62.75

ATOM 1568 H PHE A 102 8.294 10.069 87.722 1.00 0.00

ATOM 1569 H PHE A 102 10.787 10.638 88.790 1.00 0.00

ATOM 1570 H PHE A 102 10.316 13.048 88.125 1.00 0.00

ATOM 1571 H PHE A 102 8.612 12.478 87.758 1.00 0.00

ATOM 1572 H PHE A 102 12.057 11.265 86.983 1.00 0.00

ATOM 1573 H PHE A 102 8.233 12.474 85.545 1.00 0.00

ATOM 1574 H PHE A 102 12.705 10.579 84.733 1.00 0.00

ATOM 1575 H PHE A 102 8.899 11.813 83.273 1.00 0.00

ATOM 1576 H PHE A 102 11.172 10.863 82.877 1.00 0.00

ATOM 1577 N LEU A 103 8.519 12.273 90.600 1.00 56.62

ATOM 1578 CA LEU A 103 8.181 12.613 92.050 1.00 56.10

ATOM 1579 C LEU A 103 8.678 11.655 93.063 1.00 56.89

ATOM 1580 O LEU A 103 9.449 12.030 93.953 1.00 59.10

ATOM 1581 CB LEU A 103 6.689 12.685 92.342 1.00 54.01

ATOM 1582 CG LEU A 103 5.949 13.629 91.455 1.00 54.42

ATOM 1583 CD1 LEU A 103 4.522 13.895 91.990 1.00 55.12

ATOM 1584 CD2 LEU A 103 6.816 14.895 91.320 1.00 55.75

ATOM 1585 H LEU A 103 7.822 12.464 89.866 1.00 0.00

ATOM 1586 H LEU A 103 8.678 13.579 92.136 1.00 0.00

ATOM 1587 H LEU A 103 6.558 13.031 93.367 1.00 0.00

ATOM 1588 H LEU A 103 6.273 11.695 92.157 1.00 0.00

ATOM 1589 H LEU A 103 5.790 13.209 90.462 1.00 0.00

ATOM 1590 H LEU A 103 4.046 14.588 91.296 1.00 0.00

ATOM 1591 H LEU A 103 4.009 12.934 92.020 1.00 0.00

ATOM 1592 H LEU A 103 4.630 14.328 92.985 1.00 0.00

ATOM 1593 H LEU A 103 6.275 15.584 90.672 1.00 0.00

ATOM 1594 H LEU A 103 6.947 15.300 92.323 1.00 0.00

ATOM 1595 H LEU A 103 7.765 14.586 90.881 1.00 0.00

ATOM 1596 N SER A 104 8.250 10.415 92.968 1.00 58.11

ATOM 1597 CA SER A 104 8.665 9.447 94.022 1.00 60.99

ATOM 1598 C SER A 104 10.161 9.287 94.209 1.00 60.83

ATOM 1599 O SER A 104 10.540 8.710 95.162 1.00 62.98

ATOM 1600 CB SER A 104 8.062 8.087 93.841 1.00 60.03

ATOM 1601 OG SER A 104 8.640 7.626 92.676 1.00 63.55

ATOM 1602 H SER A 104 7.645 10.122 92.188 1.00 0.00

ATOM 1603 H SER A 104 8.273 9.913 94.926 1.00 0.00

ATOM 1604 H SER A 104 6.973 8.054 93.803 1.00 0.00

ATOM 1605 H SER A 104 8.312 7.412 94.660 1.00 0.00

ATOM 1606 H SER A 104 8.258 6.686 92.502 1.00 0.00

ATOM 1607 N HIS A 105 10.984 9.808 93.312 1.00 61.07

ATOM 1608 CA HIS A 105 12.457 9.861 93.525 1.00 62.03

ATOM 1609 C HIS A 105 12.984 11.213 94.018 1.00 60.88

ATOM 1610 O HIS A 105 14.209 11.398 94.049 1.00 61.14

ATOM 1611 CB HIS A 105 13.243 9.516 92.253 1.00 62.26

ATOM 1612 CG HIS A 105 13.109 8.101 91.849 1.00 63.82

ATOM 1613 ND1 HIS A 105 12.278 7.699 90.824 1.00 64.69

ATOM 1614 CD2 HIS A 105 13.674 6.980 92.340 1.00 66.75

ATOM 1615 CE1 HIS A 105 12.348 6.395 90.685 1.00 67.00

ATOM 1616 NE2 HIS A 105 13.194 5.930 91.590 1.00 69.64

ATOM 1617 H HIS A 105 10.593 10.189 92.439 1.00 0.00

ATOM 1618 H HIS A 105 12.616 9.116 94.304 1.00 0.00

ATOM 1619 H HIS A 105 14.302 9.711 92.422 1.00 0.00

ATOM 1620 H HIS A 105 12.822 10.103 91.437 1.00 0.00

ATOM 1621 H HIS A 105 11.692 8.326 90.254 1.00 0.00

ATOM 1622 H HIS A 105 14.376 6.917 93.171 1.00 0.00

ATOM 1623 H HIS A 105 11.804 5.799 89.952 1.00 0.00

ATOM 1624 N LYS A 106 12.087 12.139 94.364 1.00 58.69

ATOM 1625 CA LYS A 106 12.514 13.334 95.092 1.00 59.65

ATOM 1626 C LYS A 106 13.275 12.894 96.376 1.00 61.69

ATOM 1627 O LYS A 106 12.821 11.997 97.071 1.00 62.39

ATOM 1628 CB LYS A 106 11.330 14.254 95.420 1.00 56.99

ATOM 1629 CG LYS A 106 10.922 15.192 94.278 1.00 56.23

ATOM 1630 CD LYS A 106 9.869 16.244 94.757 1.00 55.66

ATOM 1631 CE LYS A 106 9.292 17.062 93.576 1.00 55.08

ATOM 1632 NZ LYS A 106 8.252 18.116 94.043 1.00 56.41

ATOM 1633 H LYS A 106 11.095 12.012 94.119 1.00 0.00

ATOM 1634 H LYS A 106 13.184 13.918 94.461 1.00 0.00

ATOM 1635 H LYS A 106 11.634 14.882 96.257 1.00 0.00

ATOM 1636 H LYS A 106 10.474 13.612 95.627 1.00 0.00

ATOM 1637 H LYS A 106 10.473 14.584 93.492 1.00 0.00

ATOM 1638 H LYS A 106 11.812 15.708 93.918 1.00 0.00

ATOM 1639 H LYS A 106 10.354 16.935 95.447 1.00 0.00

ATOM 1640 H LYS A 106 9.044 15.711 95.229 1.00 0.00

ATOM 1641 H LYS A 106 8.791 16.369 92.900 1.00 0.00

ATOM 1642 H LYS A 106 10.116 17.587 93.093 1.00 0.00

ATOM 1643 H LYS A 106 7.924 18.609 93.200 1.00 0.00

ATOM 1644 H LYS A 106 7.490 17.602 94.507 1.00 0.00

ATOM 1645 H LYS A 106 8.737 18.750 94.693 1.00 0.00

ATOM 1646 N ASP A 107 14.438 13.483 96.665 1.00 57.38

ATOM 1647 CA ASP A 107 15.256 12.993 97.781 1.00 58.52

ATOM 1648 C ASP A 107 15.141 14.010 98.896 1.00 56.54

ATOM 1649 O ASP A 107 15.577 15.133 98.750 1.00 55.09

ATOM 1650 CB ASP A 107 16.716 12.810 97.364 1.00 60.22

ATOM 1651 CG ASP A 107 17.558 12.056 98.406 1.00 65.76

ATOM 1652 OD1 ASP A 107 17.316 12.127 99.660 1.00 65.80

ATOM 1653 OD2 ASP A 107 18.517 11.374 97.945 1.00 71.77

ATOM 1654 H ASP A 107 14.762 14.283 96.103 1.00 0.00

ATOM 1655 H ASP A 107 14.901 12.015 98.108 1.00 0.00

ATOM 1656 H ASP A 107 17.148 13.804 97.249 1.00 0.00

ATOM 1657 H ASP A 107 16.724 12.226 96.444 1.00 0.00

ATOM 1658 N ASP A 108 14.541 13.607 100.013 1.00 56.21

ATOM 1659 CA ASP A 108 14.361 14.505 101.092 1.00 55.65

ATOM 1660 C ASP A 108 15.640 14.754 101.916 1.00 56.45

ATOM 1661 O ASP A 108 15.681 15.721 102.687 1.00 55.71

ATOM 1662 CB ASP A 108 13.241 14.027 102.000 1.00 56.70

ATOM 1663 CG ASP A 108 11.899 14.393 101.488 1.00 57.18

ATOM 1664 OD1 ASP A 108 11.610 15.601 101.457 1.00 56.60

ATOM 1665 OD2 ASP A 108 11.109 13.473 101.152 1.00 60.08

ATOM 1666 H ASP A 108 14.206 12.636 100.093 1.00 0.00

ATOM 1667 H ASP A 108 14.094 15.462 100.643 1.00 0.00

ATOM 1668 H ASP A 108 13.362 14.484 102.982 1.00 0.00

ATOM 1669 H ASP A 108 13.281 12.938 102.038 1.00 0.00

ATOM 1670 N SER A 109 16.666 13.920 101.737 1.00 58.17

ATOM 1671 CA SER A 109 17.975 14.101 102.399 1.00 59.57

ATOM 1672 C SER A 109 19.095 13.961 101.388 1.00 61.81

ATOM 1673 O SER A 109 19.804 12.988 101.414 1.00 65.38

ATOM 1674 CB SER A 109 18.158 13.023 103.437 1.00 61.46

ATOM 1675 OG SER A 109 18.972 13.478 104.486 1.00 63.21

ATOM 1676 H SER A 109 16.540 13.113 101.109 1.00 0.00

ATOM 1677 H SER A 109 18.002 15.091 102.853 1.00 0.00

ATOM 1678 H SER A 109 18.604 12.123 103.014 1.00 0.00

ATOM 1679 H SER A 109 17.189 12.799 103.883 1.00 0.00

ATOM 1680 H SER A 109 19.059 12.702 105.158 1.00 0.00

ATOM 1681 N PRO A 110 19.269 14.941 100.490 1.00 61.49

ATOM 1682 CA PRO A 110 20.139 14.814 99.288 1.00 63.22

ATOM 1683 C PRO A 110 21.659 14.696 99.394 1.00 67.89

ATOM 1684 O PRO A 110 22.337 14.541 98.354 1.00 69.91

ATOM 1685 CB PRO A 110 19.862 16.089 98.508 1.00 60.68

ATOM 1686 CG PRO A 110 18.650 16.729 99.159 1.00 58.01

ATOM 1687 CD PRO A 110 18.470 16.188 100.503 1.00 57.19

ATOM 1688 H PRO A 110 19.877 13.842 98.871 1.00 0.00

ATOM 1689 H PRO A 110 19.675 15.875 97.456 1.00 0.00

ATOM 1690 H PRO A 110 20.721 16.760 98.534 1.00 0.00

ATOM 1691 H PRO A 110 17.750 16.544 98.573 1.00 0.00

ATOM 1692 H PRO A 110 18.815 17.802 99.251 1.00 0.00

ATOM 1693 H PRO A 110 18.785 16.887 101.278 1.00 0.00

ATOM 1694 H PRO A 110 17.420 16.006 100.730 1.00 0.00

ATOM 1695 N ASP A 111 22.242 14.835 100.572 1.00 70.79

ATOM 1696 CA ASP A 111 23.750 14.686 100.658 1.00 76.10

ATOM 1697 C ASP A 111 24.604 15.942 100.319 1.00 76.19

ATOM 1698 O ASP A 111 25.838 15.931 100.392 1.00 80.48

ATOM 1699 CB ASP A 111 24.203 13.362 99.944 1.00 79.50

ATOM 1700 CG ASP A 111 25.484 13.527 99.027 1.00 87.00

ATOM 1701 OD1 ASP A 111 26.456 12.739 99.222 1.00 88.77

ATOM 1702 OD2 ASP A 111 25.517 14.430 98.108 1.00 87.90

ATOM 1703 H ASP A 111 21.685 15.040 101.414 1.00 0.00

ATOM 1704 H ASP A 111 23.978 14.595 101.720 1.00 0.00

ATOM 1705 H ASP A 111 23.381 13.055 99.297 1.00 0.00

ATOM 1706 H ASP A 111 24.448 12.642 100.725 1.00 0.00

ATOM 1707 N LEU A 112 23.927 17.059 100.078 1.00 72.40

ATOM 1708 CA LEU A 112 24.530 18.224 99.465 1.00 71.39

ATOM 1709 C LEU A 112 25.536 18.948 100.312 1.00 74.63

ATOM 1710 O LEU A 112 25.359 19.023 101.516 1.00 73.99

ATOM 1711 CB LEU A 112 23.443 19.096 98.828 1.00 66.82

ATOM 1712 CG LEU A 112 22.979 18.352 97.561 1.00 64.64

ATOM 1713 CD1 LEU A 112 21.881 19.178 96.895 1.00 60.13

ATOM 1714 CD2 LEU A 112 24.183 18.014 96.594 1.00 61.12

ATOM 1715 H LEU A 112 22.931 17.098 100.338 1.00 0.00

ATOM 1716 H LEU A 112 25.174 17.875 98.657 1.00 0.00

ATOM 1717 H LEU A 112 23.833 20.084 98.582 1.00 0.00

ATOM 1718 H LEU A 112 22.615 19.257 99.518 1.00 0.00

ATOM 1719 H LEU A 112 22.567 17.380 97.832 1.00 0.00

ATOM 1720 H LEU A 112 21.569 18.634 96.004 1.00 0.00

ATOM 1721 H LEU A 112 21.070 19.271 97.617 1.00 0.00

ATOM 1722 H LEU A 112 22.316 20.146 96.647 1.00 0.00

ATOM 1723 H LEU A 112 23.764 17.493 95.733 1.00 0.00

ATOM 1724 H LEU A 112 24.640 18.962 96.311 1.00 0.00

ATOM 1725 H LEU A 112 24.873 17.382 97.153 1.00 0.00

ATOM 1726 N PRO A 113 26.649 19.404 99.664 1.00 78.78

ATOM 1727 CA PRO A 113 27.736 20.222 100.205 1.00 83.04

ATOM 1728 C PRO A 113 27.193 21.446 100.861 1.00 81.44

ATOM 1729 O PRO A 113 26.264 22.066 100.328 1.00 78.08

ATOM 1730 CB PRO A 113 28.514 20.630 98.947 1.00 85.00

ATOM 1731 CG PRO A 113 28.400 19.440 98.054 1.00 85.54

ATOM 1732 CD PRO A 113 27.092 18.722 98.426 1.00 80.43

ATOM 1733 H PRO A 113 28.333 19.697 100.951 1.00 0.00

ATOM 1734 H PRO A 113 29.551 20.899 99.147 1.00 0.00

ATOM 1735 H PRO A 113 28.099 21.520 98.474 1.00 0.00

ATOM 1736 H PRO A 113 29.257 18.784 98.209 1.00 0.00

ATOM 1737 H PRO A 113 28.415 19.742 97.007 1.00 0.00

ATOM 1738 H PRO A 113 26.337 18.808 97.644 1.00 0.00

ATOM 1739 H PRO A 113 27.206 17.647 98.565 1.00 0.00

ATOM 1740 N LYS A 114 27.762 21.776 102.009 1.00 84.75

ATOM 1741 CA LYS A 114 27.345 22.934 102.771 1.00 84.64

ATOM 1742 C LYS A 114 27.965 24.248 102.258 1.00 86.22

ATOM 1743 O LYS A 114 29.212 24.458 102.309 1.00 91.01

ATOM 1744 CB LYS A 114 27.687 22.727 104.252 1.00 88.95

ATOM 1745 CG LYS A 114 26.541 23.063 105.177 1.00 86.67

ATOM 1746 CD LYS A 114 25.363 22.107 104.921 1.00 82.93

ATOM 1747 CE LYS A 114 24.265 22.283 105.979 1.00 82.54

ATOM 1748 NZ LYS A 114 24.779 22.312 107.392 1.00 83.46

ATOM 1749 H LYS A 114 28.527 21.190 102.372 1.00 0.00

ATOM 1750 H LYS A 114 26.267 23.031 102.646 1.00 0.00

ATOM 1751 H LYS A 114 28.521 23.381 104.506 1.00 0.00

ATOM 1752 H LYS A 114 27.914 21.671 104.395 1.00 0.00

ATOM 1753 H LYS A 114 26.223 24.086 104.977 1.00 0.00

ATOM 1754 H LYS A 114 26.876 22.974 106.211 1.00 0.00

ATOM 1755 H LYS A 114 25.729 21.081 104.967 1.00 0.00

ATOM 1756 H LYS A 114 24.939 22.331 103.942 1.00 0.00

ATOM 1757 H LYS A 114 23.580 21.439 105.901 1.00 0.00

ATOM 1758 H LYS A 114 23.796 23.251 105.801 1.00 0.00

ATOM 1759 H LYS A 114 23.962 22.433 108.007 1.00 0.00

ATOM 1760 H LYS A 114 25.254 21.413 107.558 1.00 0.00

ATOM 1761 H LYS A 114 25.430 23.107 107.459 1.00 0.00

ATOM 1762 N LEU A 115 27.079 25.113 101.761 1.00 82.21

ATOM 1763 CA LEU A 115 27.432 26.450 101.311 1.00 83.29

ATOM 1764 C LEU A 115 27.986 27.262 102.476 1.00 87.07

ATOM 1765 O LEU A 115 27.259 27.583 103.426 1.00 86.13

ATOM 1766 CB LEU A 115 26.204 27.165 100.734 1.00 78.29

ATOM 1767 CG LEU A 115 25.581 26.669 99.432 1.00 75.04

ATOM 1768 CD1 LEU A 115 24.255 27.397 99.198 1.00 70.38

ATOM 1769 CD2 LEU A 115 26.535 26.817 98.240 1.00 77.05

ATOM 1770 H LEU A 115 26.094 24.819 101.693 1.00 0.00

ATOM 1771 H LEU A 115 28.190 26.361 100.533 1.00 0.00

ATOM 1772 H LEU A 115 26.550 28.173 100.506 1.00 0.00

ATOM 1773 H LEU A 115 25.424 27.017 101.481 1.00 0.00

ATOM 1774 H LEU A 115 25.386 25.601 99.524 1.00 0.00

ATOM 1775 H LEU A 115 23.843 27.017 98.263 1.00 0.00

ATOM 1776 H LEU A 115 23.611 27.168 100.047 1.00 0.00

ATOM 1777 H LEU A 115 24.482 28.461 99.138 1.00 0.00

ATOM 1778 H LEU A 115 26.009 26.441 97.362 1.00 0.00

ATOM 1779 H LEU A 115 26.769 27.878 98.149 1.00 0.00

ATOM 1780 H LEU A 115 27.422 26.225 98.466 1.00 0.00

ATOM 1781 N LYS A 116 29.280 27.544 102.406 1.00 91.82

ATOM 1782 CA LYS A 116 29.941 28.420 103.349 1.00 96.27

ATOM 1783 C LYS A 116 30.090 29.753 102.621 1.00 96.51

ATOM 1784 O LYS A 116 31.118 30.007 101.965 1.00100.26

ATOM 1785 CB LYS A 116 31.308 27.856 103.730 1.00102.67

ATOM 1786 CG LYS A 116 32.079 28.715 104.729 1.00107.71

ATOM 1787 CD LYS A 116 33.587 28.547 104.552 1.00114.06

ATOM 1788 CE LYS A 116 34.020 28.865 103.129 1.00112.96

ATOM 1789 NZ LYS A 116 35.488 28.952 103.023 1.00118.48

ATOM 1790 H LYS A 116 29.837 27.122 101.649 1.00 0.00

ATOM 1791 H LYS A 116 29.376 28.527 104.275 1.00 0.00

ATOM 1792 H LYS A 116 31.903 27.808 102.818 1.00 0.00

ATOM 1793 H LYS A 116 31.138 26.888 104.201 1.00 0.00

ATOM 1794 H LYS A 116 31.810 28.402 105.738 1.00 0.00

ATOM 1795 H LYS A 116 31.822 29.761 104.560 1.00 0.00

ATOM 1796 H LYS A 116 33.850 27.512 104.770 1.00 0.00

ATOM 1797 H LYS A 116 34.093 29.238 105.226 1.00 0.00

ATOM 1798 H LYS A 116 33.593 29.818 102.816 1.00 0.00

ATOM 1799 H LYS A 116 33.696 28.053 102.478 1.00 0.00

ATOM 1800 H LYS A 116 35.707 29.168 102.040 1.00 0.00

ATOM 1801 H LYS A 116 35.792 29.705 103.657 1.00 0.00

ATOM 1802 H LYS A 116 35.865 28.037 103.307 1.00 0.00

ATOM 1803 N PRO A 117 29.058 30.607 102.709 1.00 92.97

ATOM 1804 CA PRO A 117 29.088 31.768 101.835 1.00 92.50

ATOM 1805 C PRO A 117 30.124 32.809 102.302 1.00 98.30

ATOM 1806 O PRO A 117 30.157 33.205 103.475 1.00100.89

ATOM 1807 CB PRO A 117 27.633 32.297 101.890 1.00 87.46

ATOM 1808 CG PRO A 117 26.813 31.216 102.590 1.00 84.34

ATOM 1809 CD PRO A 117 27.824 30.573 103.515 1.00 90.03

ATOM 1810 H PRO A 117 29.399 31.532 100.817 1.00 0.00

ATOM 1811 H PRO A 117 27.241 32.504 100.894 1.00 0.00

ATOM 1812 H PRO A 117 27.577 33.236 102.440 1.00 0.00

ATOM 1813 H PRO A 117 26.359 30.506 101.899 1.00 0.00

ATOM 1814 H PRO A 117 25.952 31.617 103.125 1.00 0.00

ATOM 1815 H PRO A 117 27.937 31.104 104.460 1.00 0.00

ATOM 1816 H PRO A 117 27.554 29.569 103.841 1.00 0.00

ATOM 1817 N ASP A 118 30.994 33.195 101.377 1.00100.52

ATOM 1818 CA ASP A 118 31.998 34.211 101.628 1.00106.23

ATOM 1819 C ASP A 118 31.621 35.498 100.882 1.00104.17

ATOM 1820 O ASP A 118 31.430 35.479 99.661 1.00101.70

ATOM 1821 CB ASP A 118 33.392 33.698 101.247 1.00111.81

ATOM 1822 CG ASP A 118 34.241 34.756 100.631 1.00116.75

ATOM 1823 OD1 ASP A 118 34.084 34.973 99.413 1.00116.36

ATOM 1824 OD2 ASP A 118 35.052 35.374 101.352 1.00123.71

ATOM 1825 H ASP A 118 30.955 32.756 100.446 1.00 0.00

ATOM 1826 H ASP A 118 32.032 34.441 102.693 1.00 0.00

ATOM 1827 H ASP A 118 33.290 32.887 100.526 1.00 0.00

ATOM 1828 H ASP A 118 33.895 33.383 102.161 1.00 0.00

ATOM 1829 N PRO A 119 31.537 36.624 101.619 1.00105.89

ATOM 1830 CA PRO A 119 30.876 37.844 101.133 1.00103.33

ATOM 1831 C PRO A 119 31.567 38.535 99.934 1.00105.01

ATOM 1832 O PRO A 119 30.857 39.078 99.071 1.00100.74

ATOM 1833 CB PRO A 119 30.837 38.752 102.376 1.00106.91

ATOM 1834 CG PRO A 119 31.383 37.919 103.525 1.00110.91

ATOM 1835 CD PRO A 119 32.215 36.848 102.909 1.00111.44

ATOM 1836 H PRO A 119 29.893 37.611 100.724 1.00 0.00

ATOM 1837 H PRO A 119 29.826 39.100 102.588 1.00 0.00

ATOM 1838 H PRO A 119 31.441 39.647 102.229 1.00 0.00

ATOM 1839 H PRO A 119 30.585 37.493 104.133 1.00 0.00

ATOM 1840 H PRO A 119 31.989 38.528 104.196 1.00 0.00

ATOM 1841 H PRO A 119 33.261 37.132 102.792 1.00 0.00

ATOM 1842 H PRO A 119 32.285 35.947 103.518 1.00 0.00

ATOM 1843 N ASN A 120 32.914 38.503 99.890 1.00110.61

ATOM 1844 CA ASN A 120 33.710 39.062 98.770 1.00113.44

ATOM 1845 C ASN A 120 33.518 38.383 97.403 1.00110.79

ATOM 1846 O ASN A 120 33.016 39.030 96.461 1.00109.26

ATOM 1847 CB ASN A 120 35.210 39.045 99.082 1.00121.10

ATOM 1848 CG ASN A 120 35.605 40.053 100.158 1.00125.00

ATOM 1849 OD1 ASN A 120 35.133 41.191 100.184 1.00121.50

ATOM 1850 ND2 ASN A 120 36.492 39.633 101.045 1.00130.45

ATOM 1851 H ASN A 120 33.417 38.067 100.676 1.00 0.00

ATOM 1852 H ASN A 120 33.321 40.077 98.684 1.00 0.00

ATOM 1853 H ASN A 120 35.746 39.306 98.169 1.00 0.00

ATOM 1854 H ASN A 120 35.461 38.051 99.453 1.00 0.00

ATOM 1855 H ASN A 120 36.804 40.262 101.799 1.00 0.00

ATOM 1856 H ASN A 120 36.871 38.677 100.982 1.00 0.00

ATOM 1857 N THR A 121 33.858 37.091 97.290 1.00110.37

ATOM 1858 CA THR A 121 33.511 36.324 96.081 1.00106.84

ATOM 1859 C THR A 121 31.986 36.343 95.871 1.00 99.32

ATOM 1860 O THR A 121 31.517 36.262 94.742 1.00 96.75

ATOM 1861 CB THR A 121 33.951 34.846 96.179 1.00108.13

ATOM 1862 OG1 THR A 121 34.374 34.398 94.886 1.00109.66

ATOM 1863 CG2 THR A 121 32.786 33.956 96.650 1.00 99.76

ATOM 1864 H THR A 121 34.368 36.630 98.057 1.00 0.00

ATOM 1865 H THR A 121 34.034 36.795 95.249 1.00 0.00

ATOM 1866 H THR A 121 34.765 34.774 96.900 1.00 0.00

ATOM 1867 H THR A 121 35.151 35.008 94.595 1.00 0.00

ATOM 1868 H THR A 121 33.164 32.935 96.696 1.00 0.00

ATOM 1869 H THR A 121 32.483 34.321 97.631 1.00 0.00

ATOM 1870 H THR A 121 31.988 34.060 95.915 1.00 0.00

ATOM 1871 N LEU A 122 31.221 36.462 96.949 1.00 96.14

ATOM 1872 CA LEU A 122 29.752 36.450 96.812 1.00 90.26

ATOM 1873 C LEU A 122 29.194 37.788 96.345 1.00 88.79

ATOM 1874 O LEU A 122 28.339 37.805 95.479 1.00 84.57

ATOM 1875 CB LEU A 122 29.066 35.979 98.097 1.00 88.10

ATOM 1876 CG LEU A 122 27.593 35.579 98.113 1.00 84.90

ATOM 1877 CD1 LEU A 122 27.195 34.532 97.021 1.00 85.05

ATOM 1878 CD2 LEU A 122 27.265 35.006 99.496 1.00 86.67

ATOM 1879 H LEU A 122 31.652 36.563 97.879 1.00 0.00

ATOM 1880 H LEU A 122 29.525 35.726 96.029 1.00 0.00

ATOM 1881 H LEU A 122 29.107 36.850 98.751 1.00 0.00

ATOM 1882 H LEU A 122 29.587 35.055 98.347 1.00 0.00

ATOM 1883 H LEU A 122 27.021 36.479 97.890 1.00 0.00

ATOM 1884 H LEU A 122 26.130 34.336 97.141 1.00 0.00

ATOM 1885 H LEU A 122 27.418 34.981 96.053 1.00 0.00

ATOM 1886 H LEU A 122 27.794 33.640 97.202 1.00 0.00

ATOM 1887 H LEU A 122 26.211 34.728 99.484 1.00 0.00

ATOM 1888 H LEU A 122 27.911 34.140 99.644 1.00 0.00

ATOM 1889 H LEU A 122 27.469 35.792 100.223 1.00 0.00

ATOM 1890 N CYS A 123 29.680 38.901 96.910 1.00 92.43

ATOM 1891 CA CYS A 123 29.320 40.233 96.409 1.00 92.96

ATOM 1892 C CYS A 123 29.883 40.443 95.002 1.00 94.97

ATOM 1893 O CYS A 123 29.327 41.237 94.232 1.00 94.08

ATOM 1894 CB CYS A 123 29.841 41.351 97.312 1.00 97.41

ATOM 1895 SG CYS A 123 28.704 42.103 98.525 1.00 97.38

ATOM 1896 H CYS A 123 30.320 38.821 97.713 1.00 0.00

ATOM 1897 H CYS A 123 28.231 40.277 96.395 1.00 0.00

ATOM 1898 H CYS A 123 29.955 42.123 96.551 1.00 0.00

ATOM 1899 H CYS A 123 30.736 40.923 97.764 1.00 0.00

ATOM 1900 N ASP A 124 30.995 39.750 94.697 1.00 97.84

ATOM 1901 CA ASP A 124 31.565 39.680 93.344 1.00 99.79

ATOM 1902 C ASP A 124 30.583 39.174 92.303 1.00 95.35

ATOM 1903 O ASP A 124 30.477 39.743 91.219 1.00 96.18

ATOM 1904 CB ASP A 124 32.881 38.863 93.306 1.00104.11

ATOM 1905 CG ASP A 124 34.096 39.714 93.661 1.00110.51

ATOM 1906 OD1 ASP A 124 33.990 40.945 93.504 1.00109.34

ATOM 1907 OD2 ASP A 124 35.150 39.179 94.095 1.00116.68

ATOM 1908 H ASP A 124 31.473 39.240 95.453 1.00 0.00

ATOM 1909 H ASP A 124 31.800 40.710 93.078 1.00 0.00

ATOM 1910 H ASP A 124 33.023 38.472 92.299 1.00 0.00

ATOM 1911 H ASP A 124 32.809 38.068 94.049 1.00 0.00

ATOM 1912 N GLU A 125 29.851 38.121 92.645 1.00 91.34

ATOM 1913 CA GLU A 125 28.974 37.464 91.684 1.00 88.61

ATOM 1914 C GLU A 125 27.634 38.106 91.427 1.00 84.54

ATOM 1915 O GLU A 125 27.019 37.804 90.397 1.00 84.07

ATOM 1916 CB GLU A 125 28.740 36.010 92.052 1.00 86.84

ATOM 1917 CG GLU A 125 29.988 35.160 91.988 1.00 93.46

ATOM 1918 CD GLU A 125 29.996 34.182 93.126 1.00 95.78

ATOM 1919 OE1 GLU A 125 31.090 33.957 93.715 1.00 99.97

ATOM 1920 OE2 GLU A 125 28.882 33.680 93.445 1.00 92.65

ATOM 1921 H GLU A 125 29.904 37.761 93.609 1.00 0.00

ATOM 1922 H GLU A 125 29.534 37.567 90.755 1.00 0.00

ATOM 1923 H GLU A 125 28.028 35.599 91.336 1.00 0.00

ATOM 1924 H GLU A 125 28.386 35.987 93.083 1.00 0.00

ATOM 1925 H GLU A 125 30.869 35.798 92.055 1.00 0.00

ATOM 1926 H GLU A 125 30.001 34.610 91.047 1.00 0.00

ATOM 1927 N PHE A 126 27.155 38.942 92.348 1.00 82.85

ATOM 1928 CA PHE A 126 25.891 39.688 92.152 1.00 79.71

ATOM 1929 C PHE A 126 26.107 40.945 91.276 1.00 84.06

ATOM 1930 O PHE A 126 25.279 41.283 90.417 1.00 83.73

ATOM 1931 CB PHE A 126 25.241 39.958 93.513 1.00 77.79

ATOM 1932 CG PHE A 126 24.440 41.223 93.602 1.00 74.40

ATOM 1933 CD1 PHE A 126 23.089 41.208 93.368 1.00 68.33

ATOM 1934 CD2 PHE A 126 25.043 42.416 93.984 1.00 75.53

ATOM 1935 CE1 PHE A 126 22.360 42.352 93.468 1.00 69.44

ATOM 1936 CE2 PHE A 126 24.310 43.575 94.090 1.00 74.79

ATOM 1937 CZ PHE A 126 22.970 43.546 93.824 1.00 72.62

ATOM 1938 H PHE A 126 27.681 39.073 93.224 1.00 0.00

ATOM 1939 H PHE A 126 25.182 39.084 91.586 1.00 0.00

ATOM 1940 H PHE A 126 26.041 40.044 94.248 1.00 0.00

ATOM 1941 H PHE A 126 24.533 39.147 93.686 1.00 0.00

ATOM 1942 H PHE A 126 22.596 40.273 93.100 1.00 0.00

ATOM 1943 H PHE A 126 26.111 42.433 94.202 1.00 0.00

ATOM 1944 H PHE A 126 21.289 42.330 93.267 1.00 0.00

ATOM 1945 H PHE A 126 24.793 44.507 94.383 1.00 0.00

ATOM 1946 H PHE A 126 22.381 44.460 93.891 1.00 0.00

ATOM 1947 N LYS A 127 27.236 41.618 91.442 1.00 87.99

ATOM 1948 CA LYS A 127 27.553 42.653 90.483 1.00 92.13

ATOM 1949 C LYS A 127 27.713 42.100 89.047 1.00 93.46

ATOM 1950 O LYS A 127 27.055 42.595 88.114 1.00 94.06

ATOM 1951 CB LYS A 127 28.744 43.531 90.926 1.00 96.62

ATOM 1952 CG LYS A 127 28.332 44.985 91.175 1.00 98.19

ATOM 1953 CD LYS A 127 28.182 45.729 89.828 1.00101.96

ATOM 1954 CE LYS A 127 27.817 47.221 89.967 1.00102.54

ATOM 1955 NZ LYS A 127 28.732 47.989 90.839 1.00104.01

ATOM 1956 H LYS A 127 27.868 41.411 92.229 1.00 0.00

ATOM 1957 H LYS A 127 26.690 43.318 90.454 1.00 0.00

ATOM 1958 H LYS A 127 29.492 43.522 90.134 1.00 0.00

ATOM 1959 H LYS A 127 29.130 43.130 91.863 1.00 0.00

ATOM 1960 H LYS A 127 29.104 45.473 91.770 1.00 0.00

ATOM 1961 H LYS A 127 27.383 45.002 91.710 1.00 0.00

ATOM 1962 H LYS A 127 27.370 45.249 89.282 1.00 0.00

ATOM 1963 H LYS A 127 29.148 45.682 89.325 1.00 0.00

ATOM 1964 H LYS A 127 26.819 47.303 90.397 1.00 0.00

ATOM 1965 H LYS A 127 27.914 47.669 88.978 1.00 0.00

ATOM 1966 H LYS A 127 28.390 48.960 90.853 1.00 0.00

ATOM 1967 H LYS A 127 28.693 47.555 91.772 1.00 0.00

ATOM 1968 H LYS A 127 29.672 47.920 90.424 1.00 0.00

ATOM 1969 N ALA A 128 28.557 41.079 88.873 1.00 94.46

ATOM 1970 CA ALA A 128 28.774 40.465 87.560 1.00 95.52

ATOM 1971 C ALA A 128 27.461 40.228 86.785 1.00 92.61

ATOM 1972 O ALA A 128 27.353 40.646 85.638 1.00 94.84

ATOM 1973 CB ALA A 128 29.584 39.176 87.691 1.00 96.47

ATOM 1974 H ALA A 128 29.071 40.712 89.687 1.00 0.00

ATOM 1975 H ALA A 128 29.351 41.177 86.970 1.00 0.00

ATOM 1976 H ALA A 128 29.708 38.774 86.685 1.00 0.00

ATOM 1977 H ALA A 128 30.540 39.445 88.141 1.00 0.00

ATOM 1978 H ALA A 128 29.011 38.503 88.329 1.00 0.00

ATOM 1979 N ASP A 129 26.461 39.603 87.429 1.00 88.21

ATOM 1980 CA ASP A 129 25.163 39.293 86.795 1.00 85.71

ATOM 1981 C ASP A 129 24.047 38.981 87.839 1.00 80.57

ATOM 1982 O ASP A 129 23.885 37.862 88.323 1.00 76.92

ATOM 1983 CB ASP A 129 25.331 38.179 85.745 1.00 87.14

ATOM 1984 CG ASP A 129 23.995 37.672 85.141 1.00 88.27

ATOM 1985 OD1 ASP A 129 22.860 37.804 85.721 1.00 86.89

ATOM 1986 OD2 ASP A 129 24.105 37.068 84.051 1.00 92.15

ATOM 1987 H ASP A 129 26.606 39.327 88.411 1.00 0.00

ATOM 1988 H ASP A 129 24.822 40.187 86.273 1.00 0.00

ATOM 1989 H ASP A 129 25.798 37.334 86.251 1.00 0.00

ATOM 1990 H ASP A 129 25.925 38.591 84.929 1.00 0.00

ATOM 1991 N GLU A 130 23.259 40.007 88.119 1.00 80.06

ATOM 1992 CA GLU A 130 22.184 39.948 89.083 1.00 76.85

ATOM 1993 C GLU A 130 21.117 38.894 88.756 1.00 73.73

ATOM 1994 O GLU A 130 20.518 38.320 89.673 1.00 70.41

ATOM 1995 CB GLU A 130 21.585 41.368 89.293 1.00 77.97

ATOM 1996 CG GLU A 130 22.614 42.313 90.038 1.00 84.31

ATOM 1997 CD GLU A 130 22.508 43.854 89.805 1.00 89.38

ATOM 1998 OE1 GLU A 130 23.579 44.514 89.764 1.00 91.38

ATOM 1999 OE2 GLU A 130 21.382 44.412 89.694 1.00 90.85

ATOM 2000 H GLU A 130 23.421 40.895 87.623 1.00 0.00

ATOM 2001 H GLU A 130 22.610 39.609 90.027 1.00 0.00

ATOM 2002 H GLU A 130 20.692 41.269 89.910 1.00 0.00

ATOM 2003 H GLU A 130 21.342 41.792 88.319 1.00 0.00

ATOM 2004 H GLU A 130 23.599 42.034 89.663 1.00 0.00

ATOM 2005 H GLU A 130 22.408 42.180 91.100 1.00 0.00

ATOM 2006 N LYS A 131 20.880 38.607 87.484 1.00 75.25

ATOM 2007 CA LYS A 131 19.755 37.707 87.178 1.00 74.02

ATOM 2008 C LYS A 131 20.148 36.226 87.301 1.00 72.27

ATOM 2009 O LYS A 131 19.327 35.378 87.658 1.00 70.09

ATOM 2010 CB LYS A 131 19.033 38.061 85.854 1.00 77.18

ATOM 2011 CG LYS A 131 17.489 37.850 85.904 1.00 77.75

ATOM 2012 CD LYS A 131 16.896 38.047 87.352 1.00 75.38

ATOM 2013 CE LYS A 131 15.378 38.159 87.378 1.00 74.83

ATOM 2014 NZ LYS A 131 14.926 38.605 88.733 1.00 70.88

ATOM 2015 H LYS A 131 21.464 39.001 86.733 1.00 0.00

ATOM 2016 H LYS A 131 19.003 37.875 87.949 1.00 0.00

ATOM 2017 H LYS A 131 19.427 37.395 85.086 1.00 0.00

ATOM 2018 H LYS A 131 19.216 39.117 85.656 1.00 0.00

ATOM 2019 H LYS A 131 17.290 36.823 85.597 1.00 0.00

ATOM 2020 H LYS A 131 17.029 38.583 85.241 1.00 0.00

ATOM 2021 H LYS A 131 17.298 38.966 87.778 1.00 0.00

ATOM 2022 H LYS A 131 17.141 37.156 87.929 1.00 0.00

ATOM 2023 H LYS A 131 14.929 37.193 87.148 1.00 0.00

ATOM 2024 H LYS A 131 15.059 38.899 86.644 1.00 0.00

ATOM 2025 H LYS A 131 13.898 38.666 88.703 1.00 0.00

ATOM 2026 H LYS A 131 15.248 37.895 89.407 1.00 0.00

ATOM 2027 H LYS A 131 15.359 39.523 88.908 1.00 0.00

ATOM 2028 N LYS A 132 21.431 35.953 87.097 1.00 73.41

ATOM 2029 CA LYS A 132 21.975 34.625 87.319 1.00 72.41

ATOM 2030 C LYS A 132 22.294 34.347 88.801 1.00 68.93

ATOM 2031 O LYS A 132 22.139 33.241 89.300 1.00 66.51

ATOM 2032 CB LYS A 132 23.215 34.440 86.466 1.00 75.90

ATOM 2033 CG LYS A 132 24.198 33.478 87.038 1.00 77.90

ATOM 2034 CD LYS A 132 25.092 32.991 85.928 1.00 87.15

ATOM 2035 CE LYS A 132 26.534 33.016 86.395 1.00 91.97

ATOM 2036 NZ LYS A 132 27.425 32.526 85.322 1.00 98.90

ATOM 2037 H LYS A 132 22.058 36.703 86.772 1.00 0.00

ATOM 2038 H LYS A 132 21.210 33.905 87.029 1.00 0.00

ATOM 2039 H LYS A 132 23.715 35.405 86.381 1.00 0.00

ATOM 2040 H LYS A 132 22.894 34.027 85.510 1.00 0.00

ATOM 2041 H LYS A 132 23.667 32.637 87.484 1.00 0.00

ATOM 2042 H LYS A 132 24.787 33.966 87.814 1.00 0.00

ATOM 2043 H LYS A 132 24.978 33.644 85.063 1.00 0.00

ATOM 2044 H LYS A 132 24.811 31.974 85.654 1.00 0.00

ATOM 2045 H LYS A 132 26.655 32.385 87.275 1.00 0.00

ATOM 2046 H LYS A 132 26.816 34.043 86.628 1.00 0.00

ATOM 2047 H LYS A 132 28.387 32.563 85.689 1.00 0.00

ATOM 2048 H LYS A 132 27.134 31.562 85.106 1.00 0.00

ATOM 2049 H LYS A 132 27.298 33.154 84.515 1.00 0.00

ATOM 2050 N PHE A 133 22.767 35.353 89.499 1.00 68.81

ATOM 2051 CA PHE A 133 22.947 35.233 90.928 1.00 67.54

ATOM 2052 C PHE A 133 21.583 34.794 91.539 1.00 63.98

ATOM 2053 O PHE A 133 21.504 33.962 92.424 1.00 61.14

ATOM 2054 CB PHE A 133 23.438 36.582 91.449 1.00 68.94

ATOM 2055 CG PHE A 133 23.647 36.655 92.939 1.00 67.70

ATOM 2056 CD1 PHE A 133 24.911 36.450 93.480 1.00 67.74

ATOM 2057 CD2 PHE A 133 22.580 36.999 93.788 1.00 64.28

ATOM 2058 CE1 PHE A 133 25.128 36.586 94.843 1.00 71.57

ATOM 2059 CE2 PHE A 133 22.750 37.085 95.146 1.00 64.11

ATOM 2060 CZ PHE A 133 24.023 36.900 95.698 1.00 69.95

ATOM 2061 H PHE A 133 23.012 36.234 89.025 1.00 0.00

ATOM 2062 H PHE A 133 23.689 34.485 91.207 1.00 0.00

ATOM 2063 H PHE A 133 22.676 37.321 91.203 1.00 0.00

ATOM 2064 H PHE A 133 24.415 36.756 90.997 1.00 0.00

ATOM 2065 H PHE A 133 25.740 36.180 92.826 1.00 0.00

ATOM 2066 H PHE A 133 21.599 37.201 93.358 1.00 0.00

ATOM 2067 H PHE A 133 26.127 36.455 95.258 1.00 0.00

ATOM 2068 H PHE A 133 21.898 37.296 95.792 1.00 0.00

ATOM 2069 H PHE A 133 24.172 36.995 96.774 1.00 0.00

ATOM 2070 N TRP A 134 20.525 35.319 90.941 1.00 64.46

ATOM 2071 CA TRP A 134 19.161 35.118 91.333 1.00 61.87

ATOM 2072 C TRP A 134 18.771 33.664 91.084 1.00 61.31

ATOM 2073 O TRP A 134 18.221 33.026 91.957 1.00 60.39

ATOM 2074 CB TRP A 134 18.311 36.092 90.518 1.00 62.39

ATOM 2075 CG TRP A 134 16.888 36.100 90.894 1.00 60.38

ATOM 2076 CD1 TRP A 134 16.334 36.762 91.920 1.00 60.35

ATOM 2077 CD2 TRP A 134 15.816 35.450 90.200 1.00 58.91

ATOM 2078 NE1 TRP A 134 14.980 36.547 91.952 1.00 58.77

ATOM 2079 CE2 TRP A 134 14.633 35.740 90.904 1.00 58.84

ATOM 2080 CE3 TRP A 134 15.743 34.665 89.034 1.00 59.93

ATOM 2081 CZ2 TRP A 134 13.370 35.250 90.510 1.00 59.50

ATOM 2082 CZ3 TRP A 134 14.475 34.159 88.630 1.00 57.83

ATOM 2083 CH2 TRP A 134 13.316 34.467 89.370 1.00 61.10

ATOM 2084 H TRP A 134 20.697 35.923 90.125 1.00 0.00

ATOM 2085 H TRP A 134 19.008 35.310 92.395 1.00 0.00

ATOM 2086 H TRP A 134 18.366 35.814 89.466 1.00 0.00

ATOM 2087 H TRP A 134 18.678 37.096 90.729 1.00 0.00

ATOM 2088 H TRP A 134 16.882 37.383 92.628 1.00 0.00

ATOM 2089 H TRP A 134 14.330 36.931 92.652 1.00 0.00

ATOM 2090 H TRP A 134 16.639 34.449 88.452 1.00 0.00

ATOM 2091 H TRP A 134 12.471 35.479 91.082 1.00 0.00

ATOM 2092 H TRP A 134 14.400 33.530 87.743 1.00 0.00

ATOM 2093 H TRP A 134 12.353 34.079 89.036 1.00 0.00

ATOM 2094 N GLY A 135 19.042 33.152 89.884 1.00 63.39

ATOM 2095 CA GLY A 135 18.772 31.759 89.530 1.00 62.22

ATOM 2096 C GLY A 135 19.554 30.767 90.358 1.00 61.84

ATOM 2097 O GLY A 135 18.993 29.796 90.893 1.00 59.00

ATOM 2098 H GLY A 135 19.462 33.767 89.172 1.00 0.00

ATOM 2099 H GLY A 135 19.088 31.642 88.493 1.00 0.00

ATOM 2100 H GLY A 135 17.707 31.583 89.681 1.00 0.00

ATOM 2101 N LYS A 136 20.860 31.000 90.488 1.00 64.25

ATOM 2102 CA LYS A 136 21.677 30.131 91.342 1.00 64.49

ATOM 2103 C LYS A 136 21.077 29.969 92.746 1.00 60.65

ATOM 2104 O LYS A 136 21.207 28.924 93.350 1.00 60.88

ATOM 2105 CB LYS A 136 23.112 30.618 91.458 1.00 67.28

ATOM 2106 CG LYS A 136 24.062 29.454 91.666 1.00 72.29

ATOM 2107 CD LYS A 136 25.018 29.299 90.446 1.00 80.80

ATOM 2108 CE LYS A 136 25.783 27.950 90.447 1.00 85.88

ATOM 2109 NZ LYS A 136 26.968 27.919 89.459 1.00 93.69

ATOM 2110 H LYS A 136 21.295 31.789 89.989 1.00 0.00

ATOM 2111 H LYS A 136 21.681 29.158 90.850 1.00 0.00

ATOM 2112 H LYS A 136 23.188 31.290 92.313 1.00 0.00

ATOM 2113 H LYS A 136 23.384 31.131 90.536 1.00 0.00

ATOM 2114 H LYS A 136 23.463 28.548 91.761 1.00 0.00

ATOM 2115 H LYS A 136 24.641 29.630 92.573 1.00 0.00

ATOM 2116 H LYS A 136 25.756 30.100 90.493 1.00 0.00

ATOM 2117 H LYS A 136 24.411 29.333 89.541 1.00 0.00

ATOM 2118 H LYS A 136 25.083 27.170 90.146 1.00 0.00

ATOM 2119 H LYS A 136 26.190 27.803 91.448 1.00 0.00

ATOM 2120 H LYS A 136 27.402 26.988 89.539 1.00 0.00

ATOM 2121 H LYS A 136 26.580 28.081 88.519 1.00 0.00

ATOM 2122 H LYS A 136 27.615 28.669 89.741 1.00 0.00

ATOM 2123 N TYR A 137 20.444 31.002 93.274 1.00 58.27

ATOM 2124 CA TYR A 137 19.892 30.878 94.563 1.00 55.75

ATOM 2125 C TYR A 137 18.631 30.007 94.506 1.00 55.31

ATOM 2126 O TYR A 137 18.479 29.078 95.313 1.00 55.33

ATOM 2127 CB TYR A 137 19.559 32.240 95.115 1.00 56.42

ATOM 2128 CG TYR A 137 18.809 32.177 96.432 1.00 54.40

ATOM 2129 CD1 TYR A 137 19.493 32.120 97.646 1.00 55.31

ATOM 2130 CD2 TYR A 137 17.424 32.114 96.454 1.00 51.68

ATOM 2131 CE1 TYR A 137 18.816 32.037 98.869 1.00 55.88

ATOM 2132 CE2 TYR A 137 16.734 32.053 97.664 1.00 54.22

ATOM 2133 CZ TYR A 137 17.427 32.015 98.859 1.00 55.02

ATOM 2134 OH TYR A 137 16.707 31.925 100.015 1.00 58.32

ATOM 2135 H TYR A 137 20.356 31.885 92.750 1.00 0.00

ATOM 2136 H TYR A 137 20.621 30.403 95.220 1.00 0.00

ATOM 2137 H TYR A 137 18.916 32.739 94.390 1.00 0.00

ATOM 2138 H TYR A 137 20.497 32.768 95.286 1.00 0.00

ATOM 2139 H TYR A 137 20.583 32.141 97.643 1.00 0.00

ATOM 2140 H TYR A 137 16.869 32.112 95.516 1.00 0.00

ATOM 2141 H TYR A 137 19.367 31.991 99.808 1.00 0.00

ATOM 2142 H TYR A 137 15.644 32.035 97.667 1.00 0.00

ATOM 2143 H TYR A 137 17.385 31.884 100.789 1.00 0.00

ATOM 2144 N LEU A 138 17.696 30.327 93.595 1.00 54.84

ATOM 2145 CA LEU A 138 16.589 29.447 93.341 1.00 52.97

ATOM 2146 C LEU A 138 17.055 27.975 93.324 1.00 53.44

ATOM 2147 O LEU A 138 16.559 27.133 94.112 1.00 53.04

ATOM 2148 CB LEU A 138 15.815 29.816 92.082 1.00 53.27

ATOM 2149 CG LEU A 138 15.013 31.093 92.099 1.00 54.65

ATOM 2150 CD1 LEU A 138 14.414 31.314 90.757 1.00 54.82

ATOM 2151 CD2 LEU A 138 13.904 31.163 93.191 1.00 53.29

ATOM 2152 H LEU A 138 17.774 31.212 93.074 1.00 0.00

ATOM 2153 H LEU A 138 15.886 29.570 94.165 1.00 0.00

ATOM 2154 H LEU A 138 15.088 29.018 91.933 1.00 0.00

ATOM 2155 H LEU A 138 16.570 29.966 91.310 1.00 0.00

ATOM 2156 H LEU A 138 15.718 31.883 92.357 1.00 0.00

ATOM 2157 H LEU A 138 13.845 32.242 90.811 1.00 0.00

ATOM 2158 H LEU A 138 15.239 31.385 90.048 1.00 0.00

ATOM 2159 H LEU A 138 13.773 30.457 90.549 1.00 0.00

ATOM 2160 H LEU A 138 13.413 32.130 93.085 1.00 0.00

ATOM 2161 H LEU A 138 13.218 30.337 93.002 1.00 0.00

ATOM 2162 H LEU A 138 14.401 31.065 94.156 1.00 0.00

ATOM 2163 N TYR A 139 18.021 27.665 92.464 1.00 54.96

ATOM 2164 CA TYR A 139 18.452 26.277 92.281 1.00 54.31

ATOM 2165 C TYR A 139 19.085 25.698 93.507 1.00 53.47

ATOM 2166 O TYR A 139 19.029 24.510 93.690 1.00 54.86

ATOM 2167 CB TYR A 139 19.418 26.193 91.131 1.00 55.64

ATOM 2168 CG TYR A 139 20.461 25.105 91.099 1.00 57.06

ATOM 2169 CD1 TYR A 139 20.269 23.954 90.330 1.00 57.30

ATOM 2170 CD2 TYR A 139 21.713 25.268 91.723 1.00 57.25

ATOM 2171 CE1 TYR A 139 21.272 22.977 90.199 1.00 55.63

ATOM 2172 CE2 TYR A 139 22.720 24.273 91.599 1.00 56.15

ATOM 2173 CZ TYR A 139 22.470 23.121 90.843 1.00 57.62

ATOM 2174 OH TYR A 139 23.457 22.121 90.692 1.00 62.81

ATOM 2175 H TYR A 139 18.473 28.412 91.918 1.00 0.00

ATOM 2176 H TYR A 139 17.557 25.691 92.072 1.00 0.00

ATOM 2177 H TYR A 139 19.992 27.118 91.192 1.00 0.00

ATOM 2178 H TYR A 139 18.790 25.996 90.262 1.00 0.00

ATOM 2179 H TYR A 139 19.317 23.810 89.819 1.00 0.00

ATOM 2180 H TYR A 139 21.912 26.167 92.307 1.00 0.00

ATOM 2181 H TYR A 139 21.093 22.098 89.579 1.00 0.00

ATOM 2182 H TYR A 139 23.684 24.406 92.091 1.00 0.00

ATOM 2183 H TYR A 139 23.063 21.401 90.070 1.00 0.00

ATOM 2184 N GLU A 140 19.695 26.492 94.346 1.00 53.15

ATOM 2185 CA GLU A 140 20.353 25.873 95.449 1.00 55.88

ATOM 2186 C GLU A 140 19.360 25.561 96.572 1.00 54.94

ATOM 2187 O GLU A 140 19.488 24.527 97.199 1.00 56.09

ATOM 2188 CB GLU A 140 21.506 26.720 95.931 1.00 59.16

ATOM 2189 CG GLU A 140 22.792 25.864 96.294 1.00 67.14

ATOM 2190 CD GLU A 140 23.691 25.477 95.098 1.00 67.33

ATOM 2191 OE1 GLU A 140 24.212 26.408 94.494 1.00 73.89

ATOM 2192 OE2 GLU A 140 23.897 24.277 94.795 1.00 64.28

ATOM 2193 H GLU A 140 19.700 27.514 94.217 1.00 0.00

ATOM 2194 H GLU A 140 20.769 24.923 95.114 1.00 0.00

ATOM 2195 H GLU A 140 21.158 27.200 96.846 1.00 0.00

ATOM 2196 H GLU A 140 21.745 27.433 95.142 1.00 0.00

ATOM 2197 H GLU A 140 22.453 24.930 96.743 1.00 0.00

ATOM 2198 H GLU A 140 23.413 26.492 96.933 1.00 0.00

ATOM 2199 N ILE A 141 18.336 26.391 96.786 1.00 53.37

ATOM 2200 CA ILE A 141 17.404 26.102 97.851 1.00 52.45

ATOM 2201 C ILE A 141 16.510 24.909 97.433 1.00 52.04

ATOM 2202 O ILE A 141 16.212 23.971 98.229 1.00 50.12

ATOM 2203 CB ILE A 141 16.482 27.295 98.133 1.00 51.95

ATOM 2204 CG1 ILE A 141 17.298 28.515 98.585 1.00 51.83

ATOM 2205 CG2 ILE A 141 15.471 26.914 99.184 1.00 49.34

ATOM 2206 CD1 ILE A 141 18.116 28.261 99.717 1.00 45.83

ATOM 2207 H ILE A 141 18.212 27.228 96.198 1.00 0.00

ATOM 2208 H ILE A 141 17.984 25.877 98.746 1.00 0.00

ATOM 2209 H ILE A 141 15.958 27.563 97.216 1.00 0.00

ATOM 2210 H ILE A 141 16.643 29.348 98.839 1.00 0.00

ATOM 2211 H ILE A 141 18.005 28.745 97.788 1.00 0.00

ATOM 2212 H ILE A 141 14.841 27.787 99.351 1.00 0.00

ATOM 2213 H ILE A 141 14.901 26.073 98.788 1.00 0.00

ATOM 2214 H ILE A 141 16.030 26.640 100.079 1.00 0.00

ATOM 2215 H ILE A 141 18.642 29.188 99.944 1.00 0.00

ATOM 2216 H ILE A 141 17.453 27.962 100.529 1.00 0.00

ATOM 2217 H ILE A 141 18.804 27.462 99.440 1.00 0.00

ATOM 2218 N ALA A 142 16.119 24.949 96.169 1.00 50.88

ATOM 2219 CA ALA A 142 15.106 24.041 95.684 1.00 49.97

ATOM 2220 C ALA A 142 15.678 22.599 95.527 1.00 51.60

ATOM 2221 O ALA A 142 14.975 21.618 95.744 1.00 51.05

ATOM 2222 CB ALA A 142 14.537 24.565 94.387 1.00 48.89

ATOM 2223 H ALA A 142 16.541 25.634 95.526 1.00 0.00

ATOM 2224 H ALA A 142 14.298 23.982 96.413 1.00 0.00

ATOM 2225 H ALA A 142 13.781 23.852 94.060 1.00 0.00

ATOM 2226 H ALA A 142 14.106 25.543 94.600 1.00 0.00

ATOM 2227 H ALA A 142 15.364 24.632 93.680 1.00 0.00

ATOM 2228 N ARG A 143 16.969 22.473 95.175 1.00 53.39

ATOM 2229 CA ARG A 143 17.553 21.143 95.052 1.00 53.73

ATOM 2230 C ARG A 143 17.823 20.520 96.411 1.00 53.82

ATOM 2231 O ARG A 143 17.884 19.297 96.509 1.00 56.31

ATOM 2232 CB ARG A 143 18.813 21.152 94.177 1.00 55.87

ATOM 2233 CG ARG A 143 20.027 21.802 94.850 1.00 56.71

ATOM 2234 CD ARG A 143 20.968 22.227 93.820 1.00 55.19

ATOM 2235 NE ARG A 143 21.686 21.092 93.301 1.00 56.59

ATOM 2236 CZ ARG A 143 22.979 20.880 93.499 1.00 61.57

ATOM 2237 NH1 ARG A 143 23.682 21.762 94.196 1.00 58.99

ATOM 2238 NH2 ARG A 143 23.576 19.792 92.983 1.00 63.36

ATOM 2239 H ARG A 143 17.541 23.310 94.994 1.00 0.00

ATOM 2240 H ARG A 143 16.816 20.517 94.549 1.00 0.00

ATOM 2241 H ARG A 143 18.586 21.734 93.284 1.00 0.00

ATOM 2242 H ARG A 143 19.073 20.114 93.970 1.00 0.00

ATOM 2243 H ARG A 143 20.522 21.118 95.539 1.00 0.00

ATOM 2244 H ARG A 143 19.711 22.689 95.399 1.00 0.00

ATOM 2245 H ARG A 143 21.680 22.935 94.245 1.00 0.00

ATOM 2246 H ARG A 143 20.416 22.691 93.003 1.00 0.00

ATOM 2247 H ARG A 143 21.163 20.403 92.742 1.00 0.00

ATOM 2248 H ARG A 143 24.688 21.608 94.356 1.00 0.00

ATOM 2249 H ARG A 143 23.222 22.601 94.577 1.00 0.00

ATOM 2250 H ARG A 143 24.581 19.633 93.140 1.00 0.00

ATOM 2251 H ARG A 143 23.028 19.117 92.430 1.00 0.00

ATOM 2252 N ARG A 144 18.012 21.319 97.463 1.00 52.62

ATOM 2253 CA ARG A 144 18.113 20.753 98.798 1.00 51.34

ATOM 2254 C ARG A 144 16.770 20.670 99.464 1.00 50.41

ATOM 2255 O ARG A 144 16.626 19.993 100.497 1.00 50.66

ATOM 2256 CB ARG A 144 19.020 21.595 99.666 1.00 52.33

ATOM 2257 CG ARG A 144 20.416 21.604 99.191 1.00 53.37

ATOM 2258 CD ARG A 144 21.169 22.734 99.855 1.00 53.21

ATOM 2259 NE ARG A 144 22.579 22.624 99.573 1.00 53.55

ATOM 2260 CZ ARG A 144 23.123 23.057 98.453 1.00 54.80

ATOM 2261 NH1 ARG A 144 22.352 23.623 97.549 1.00 54.94

ATOM 2262 NH2 ARG A 144 24.410 22.932 98.239 1.00 55.88

ATOM 2263 H ARG A 144 18.086 22.338 97.329 1.00 0.00

ATOM 2264 H ARG A 144 18.523 19.749 98.686 1.00 0.00

ATOM 2265 H ARG A 144 19.014 21.191 100.679 1.00 0.00

ATOM 2266 H ARG A 144 18.664 22.624 99.621 1.00 0.00

ATOM 2267 H ARG A 144 20.412 21.756 98.112 1.00 0.00

ATOM 2268 H ARG A 144 20.884 20.647 99.423 1.00 0.00

ATOM 2269 H ARG A 144 21.020 22.724 100.935 1.00 0.00

ATOM 2270 H ARG A 144 20.829 23.682 99.438 1.00 0.00

ATOM 2271 H ARG A 144 23.189 22.187 100.279 1.00 0.00

ATOM 2272 H ARG A 144 22.759 23.967 96.668 1.00 0.00

ATOM 2273 H ARG A 144 21.342 23.722 97.723 1.00 0.00

ATOM 2274 H ARG A 144 24.821 23.275 97.359 1.00 0.00

ATOM 2275 H ARG A 144 25.010 22.491 98.951 1.00 0.00

ATOM 2276 N HIS A 145 15.768 21.380 98.937 1.00 49.63

ATOM 2277 CA HIS A 145 14.393 21.221 99.475 1.00 47.44

ATOM 2278 C HIS A 145 13.415 20.893 98.385 1.00 48.17

ATOM 2279 O HIS A 145 12.743 21.738 97.839 1.00 49.35

ATOM 2280 CB HIS A 145 13.969 22.419 100.303 1.00 47.19

ATOM 2281 CG HIS A 145 14.997 22.845 101.324 1.00 50.73

ATOM 2282 ND1 HIS A 145 14.864 22.606 102.683 1.00 48.39

ATOM 2283 CD2 HIS A 145 16.175 23.507 101.169 1.00 50.22

ATOM 2284 CE1 HIS A 145 15.928 23.080 103.309 1.00 50.44

ATOM 2285 NE2 HIS A 145 16.731 23.645 102.408 1.00 52.30

ATOM 2286 H HIS A 145 15.948 22.034 98.162 1.00 0.00

ATOM 2287 H HIS A 145 14.400 20.370 100.156 1.00 0.00

ATOM 2288 H HIS A 145 13.066 22.141 100.846 1.00 0.00

ATOM 2289 H HIS A 145 13.828 23.255 99.618 1.00 0.00

ATOM 2290 H HIS A 145 14.066 22.135 103.132 1.00 0.00

ATOM 2291 H HIS A 145 16.597 23.862 100.229 1.00 0.00

ATOM 2292 H HIS A 145 16.116 23.019 104.381 1.00 0.00

ATOM 2293 N PRO A 146 13.273 19.623 98.061 1.00 49.62

ATOM 2294 CA PRO A 146 12.540 19.373 96.833 1.00 50.01

ATOM 2295 C PRO A 146 11.010 19.481 96.906 1.00 50.94

ATOM 2296 O PRO A 146 10.323 19.231 95.915 1.00 52.85

ATOM 2297 CB PRO A 146 13.016 17.983 96.439 1.00 52.28

ATOM 2298 CG PRO A 146 13.264 17.301 97.770 1.00 51.54

ATOM 2299 CD PRO A 146 13.653 18.390 98.768 1.00 50.55

ATOM 2300 H PRO A 146 12.747 20.151 96.098 1.00 0.00

ATOM 2301 H PRO A 146 13.906 18.007 95.811 1.00 0.00

ATOM 2302 H PRO A 146 12.287 17.450 95.829 1.00 0.00

ATOM 2303 H PRO A 146 14.061 16.564 97.675 1.00 0.00

ATOM 2304 H PRO A 146 12.372 16.769 98.101 1.00 0.00

ATOM 2305 H PRO A 146 13.134 18.298 99.722 1.00 0.00

ATOM 2306 H PRO A 146 14.701 18.376 99.066 1.00 0.00

ATOM 2307 N TYR A 147 10.449 19.905 98.014 1.00 49.60

ATOM 2308 CA TYR A 147 9.018 20.119 98.024 1.00 49.83

ATOM 2309 C TYR A 147 8.796 21.562 98.501 1.00 50.32

ATOM 2310 O TYR A 147 7.707 21.938 98.953 1.00 50.43

ATOM 2311 CB TYR A 147 8.324 19.166 98.996 1.00 50.17

ATOM 2312 CG TYR A 147 8.289 17.682 98.678 1.00 51.03

ATOM 2313 CD1 TYR A 147 9.349 16.867 99.003 1.00 55.00

ATOM 2314 CD2 TYR A 147 7.143 17.077 98.114 1.00 54.88

ATOM 2315 CE1 TYR A 147 9.302 15.493 98.769 1.00 56.92

ATOM 2316 CE2 TYR A 147 7.070 15.734 97.889 1.00 54.32

ATOM 2317 CZ TYR A 147 8.169 14.930 98.225 1.00 57.33

ATOM 2318 OH TYR A 147 8.165 13.547 98.021 1.00 59.45

ATOM 2319 H TYR A 147 11.014 20.081 98.857 1.00 0.00

ATOM 2320 H TYR A 147 8.606 19.942 97.031 1.00 0.00

ATOM 2321 H TYR A 147 7.279 19.476 99.001 1.00 0.00

ATOM 2322 H TYR A 147 8.903 19.236 99.917 1.00 0.00

ATOM 2323 H TYR A 147 10.242 17.303 99.451 1.00 0.00

ATOM 2324 H TYR A 147 6.291 17.704 97.851 1.00 0.00

ATOM 2325 H TYR A 147 10.159 14.867 99.016 1.00 0.00

ATOM 2326 H TYR A 147 6.173 15.293 97.455 1.00 0.00

ATOM 2327 H TYR A 147 9.078 13.193 98.341 1.00 0.00

ATOM 2328 N PHE A 148 9.830 22.392 98.412 1.00 54.03

ATOM 2329 CA PHE A 148 9.622 23.804 98.735 1.00 55.32

ATOM 2330 C PHE A 148 8.464 24.489 97.994 1.00 54.81

ATOM 2331 O PHE A 148 8.287 24.318 96.823 1.00 55.19

ATOM 2332 CB PHE A 148 10.854 24.580 98.414 1.00 57.15

ATOM 2333 CG PHE A 148 11.043 25.760 99.280 1.00 60.31

ATOM 2334 CD1 PHE A 148 10.368 26.941 99.028 1.00 61.43

ATOM 2335 CD2 PHE A 148 11.902 25.698 100.354 1.00 58.92

ATOM 2336 CE1 PHE A 148 10.562 28.029 99.832 1.00 56.81

ATOM 2337 CE2 PHE A 148 12.106 26.791 101.128 1.00 55.58

ATOM 2338 CZ PHE A 148 11.437 27.943 100.864 1.00 57.48

ATOM 2339 H PHE A 148 10.757 22.049 98.122 1.00 0.00

ATOM 2340 H PHE A 148 9.374 23.802 99.796 1.00 0.00

ATOM 2341 H PHE A 148 10.771 24.935 97.387 1.00 0.00

ATOM 2342 H PHE A 148 11.708 23.923 98.575 1.00 0.00

ATOM 2343 H PHE A 148 9.679 27.004 98.186 1.00 0.00

ATOM 2344 H PHE A 148 12.420 24.766 100.582 1.00 0.00

ATOM 2345 H PHE A 148 10.019 28.956 99.646 1.00 0.00

ATOM 2346 H PHE A 148 12.806 26.746 101.962 1.00 0.00

ATOM 2347 H PHE A 148 11.607 28.816 101.494 1.00 0.00

ATOM 2348 N TYR A 149 7.669 25.254 98.730 1.00 55.69

ATOM 2349 CA TYR A 149 6.568 26.077 98.205 1.00 55.09

ATOM 2350 C TYR A 149 7.178 27.080 97.249 1.00 55.36

ATOM 2351 O TYR A 149 8.015 27.902 97.643 1.00 56.91

ATOM 2352 CB TYR A 149 5.907 26.738 99.401 1.00 54.44

ATOM 2353 CG TYR A 149 4.602 27.405 99.121 1.00 56.28

ATOM 2354 CD1 TYR A 149 3.831 27.028 98.031 1.00 55.87

ATOM 2355 CD2 TYR A 149 4.087 28.343 100.006 1.00 56.46

ATOM 2356 CE1 TYR A 149 2.649 27.606 97.790 1.00 59.72

ATOM 2357 CE2 TYR A 149 2.864 28.934 99.787 1.00 58.39

ATOM 2358 CZ TYR A 149 2.162 28.579 98.641 1.00 60.14

ATOM 2359 OH TYR A 149 0.955 29.131 98.361 1.00 56.97

ATOM 2360 H TYR A 149 7.835 25.274 99.746 1.00 0.00

ATOM 2361 H TYR A 149 5.812 25.508 97.663 1.00 0.00

ATOM 2362 H TYR A 149 6.583 27.514 99.759 1.00 0.00

ATOM 2363 H TYR A 149 5.689 25.944 100.115 1.00 0.00

ATOM 2364 H TYR A 149 4.194 26.249 97.361 1.00 0.00

ATOM 2365 H TYR A 149 4.662 28.616 100.891 1.00 0.00

ATOM 2366 H TYR A 149 2.067 27.308 96.918 1.00 0.00

ATOM 2367 H TYR A 149 2.457 29.659 100.492 1.00 0.00

ATOM 2368 H TYR A 149 0.610 28.681 97.501 1.00 0.00

ATOM 2369 N ALA A 150 6.789 27.000 95.990 1.00 55.58

ATOM 2370 CA ALA A 150 7.597 27.548 94.885 1.00 56.29

ATOM 2371 C ALA A 150 7.424 29.072 94.608 1.00 57.93

ATOM 2372 O ALA A 150 8.408 29.760 94.219 1.00 57.96

ATOM 2373 CB ALA A 150 7.356 26.810 93.668 1.00 57.02

ATOM 2374 H ALA A 150 5.893 26.540 95.773 1.00 0.00

ATOM 2375 H ALA A 150 8.626 27.430 95.223 1.00 0.00

ATOM 2376 H ALA A 150 7.978 27.260 92.894 1.00 0.00

ATOM 2377 H ALA A 150 7.637 25.775 93.862 1.00 0.00

ATOM 2378 H ALA A 150 6.294 26.906 93.442 1.00 0.00

ATOM 2379 N PRO A 151 6.174 29.592 94.752 1.00 58.31

ATOM 2380 CA PRO A 151 5.957 31.036 94.701 1.00 59.55

ATOM 2381 C PRO A 151 6.579 31.750 95.864 1.00 58.11

ATOM 2382 O PRO A 151 6.832 32.942 95.741 1.00 58.13

ATOM 2383 CB PRO A 151 4.438 31.174 94.757 1.00 60.22

ATOM 2384 CG PRO A 151 3.961 29.951 94.267 1.00 59.25

ATOM 2385 CD PRO A 151 4.894 28.886 94.680 1.00 58.30

ATOM 2386 H PRO A 151 6.410 31.478 93.813 1.00 0.00

ATOM 2387 H PRO A 151 4.023 32.021 94.210 1.00 0.00

ATOM 2388 H PRO A 151 4.079 31.310 95.777 1.00 0.00

ATOM 2389 H PRO A 151 3.929 30.014 93.179 1.00 0.00

ATOM 2390 H PRO A 151 2.950 29.774 94.633 1.00 0.00

ATOM 2391 H PRO A 151 4.617 28.420 95.626 1.00 0.00

ATOM 2392 H PRO A 151 4.913 28.039 93.994 1.00 0.00

ATOM 2393 N GLU A 152 6.829 31.042 96.964 1.00 55.87

ATOM 2394 CA GLU A 152 7.583 31.677 98.061 1.00 57.91

ATOM 2395 C GLU A 152 9.093 31.694 97.814 1.00 57.42

ATOM 2396 O GLU A 152 9.815 32.572 98.262 1.00 58.26

ATOM 2397 CB GLU A 152 7.226 31.128 99.453 1.00 57.65

ATOM 2398 CG GLU A 152 5.830 31.597 99.960 1.00 60.56

ATOM 2399 CD GLU A 152 5.930 32.857 100.850 1.00 68.94

ATOM 2400 OE1 GLU A 152 6.623 32.776 101.884 1.00 70.72

ATOM 2401 OE2 GLU A 152 5.328 33.940 100.536 1.00 72.63

ATOM 2402 H GLU A 152 6.502 30.069 97.047 1.00 0.00

ATOM 2403 H GLU A 152 7.257 32.717 98.061 1.00 0.00

ATOM 2404 H GLU A 152 7.974 31.508 100.149 1.00 0.00

ATOM 2405 H GLU A 152 7.214 30.040 99.385 1.00 0.00

ATOM 2406 H GLU A 152 5.390 30.795 100.553 1.00 0.00

ATOM 2407 H GLU A 152 5.219 31.847 99.093 1.00 0.00

ATOM 2408 N LEU A 153 9.549 30.733 97.058 1.00 56.91

ATOM 2409 CA LEU A 153 10.931 30.675 96.703 1.00 56.77

ATOM 2410 C LEU A 153 11.327 31.873 95.889 1.00 57.96

ATOM 2411 O LEU A 153 12.403 32.376 96.022 1.00 59.20

ATOM 2412 CB LEU A 153 11.200 29.427 95.919 1.00 56.80

ATOM 2413 CG LEU A 153 12.642 29.051 95.989 1.00 57.63

ATOM 2414 CD1 LEU A 153 13.224 29.761 97.129 1.00 57.93

ATOM 2415 CD2 LEU A 153 12.742 27.618 96.180 1.00 58.66

ATOM 2416 H LEU A 153 8.904 30.008 96.713 1.00 0.00

ATOM 2417 H LEU A 153 11.520 30.669 97.620 1.00 0.00

ATOM 2418 H LEU A 153 10.935 29.602 94.876 1.00 0.00

ATOM 2419 H LEU A 153 10.609 28.614 96.342 1.00 0.00

ATOM 2420 H LEU A 153 13.171 29.318 95.074 1.00 0.00

ATOM 2421 H LEU A 153 14.277 29.482 97.174 1.00 0.00

ATOM 2422 H LEU A 153 13.095 30.827 96.940 1.00 0.00

ATOM 2423 H LEU A 153 12.681 29.438 98.017 1.00 0.00

ATOM 2424 H LEU A 153 13.804 27.376 96.227 1.00 0.00

ATOM 2425 H LEU A 153 12.232 27.385 97.114 1.00 0.00

ATOM 2426 H LEU A 153 12.257 27.148 95.325 1.00 0.00

ATOM 2427 N LEU A 154 10.459 32.304 95.007 1.00 58.83

ATOM 2428 CA LEU A 154 10.738 33.522 94.217 1.00 58.87

ATOM 2429 C LEU A 154 10.758 34.766 95.141 1.00 58.93

ATOM 2430 O LEU A 154 11.615 35.629 95.002 1.00 59.04

ATOM 2431 CB LEU A 154 9.707 33.690 93.103 1.00 59.59

ATOM 2432 CG LEU A 154 9.407 32.544 92.115 1.00 59.80

ATOM 2433 CD1 LEU A 154 8.055 32.779 91.430 1.00 59.08

ATOM 2434 CD2 LEU A 154 10.477 32.439 91.073 1.00 57.74

ATOM 2435 H LEU A 154 9.577 31.791 94.863 1.00 0.00

ATOM 2436 H LEU A 154 11.720 33.420 93.754 1.00 0.00

ATOM 2437 H LEU A 154 10.134 34.473 92.476 1.00 0.00

ATOM 2438 H LEU A 154 8.768 33.843 93.634 1.00 0.00

ATOM 2439 H LEU A 154 9.376 31.613 92.681 1.00 0.00

ATOM 2440 H LEU A 154 7.894 31.945 90.747 1.00 0.00

ATOM 2441 H LEU A 154 7.301 32.810 92.216 1.00 0.00

ATOM 2442 H LEU A 154 8.127 33.729 90.900 1.00 0.00

ATOM 2443 H LEU A 154 10.201 31.615 90.415 1.00 0.00

ATOM 2444 H LEU A 154 10.500 33.391 90.543 1.00 0.00

ATOM 2445 H LEU A 154 11.413 32.244 91.596 1.00 0.00

ATOM 2446 N TYR A 155 9.781 34.843 96.054 1.00 58.63

ATOM 2447 CA TYR A 155 9.712 35.863 97.105 1.00 59.34

ATOM 2448 C TYR A 155 11.048 36.001 97.836 1.00 59.33

ATOM 2449 O TYR A 155 11.632 37.092 97.879 1.00 60.92

ATOM 2450 CB TYR A 155 8.580 35.528 98.124 1.00 59.59

ATOM 2451 CG TYR A 155 8.657 36.402 99.339 1.00 60.24

ATOM 2452 CD1 TYR A 155 8.436 37.783 99.245 1.00 64.42

ATOM 2453 CD2 TYR A 155 9.025 35.871 100.573 1.00 60.36

ATOM 2454 CE1 TYR A 155 8.598 38.601 100.369 1.00 66.66

ATOM 2455 CE2 TYR A 155 9.205 36.671 101.677 1.00 61.33

ATOM 2456 CZ TYR A 155 8.974 38.015 101.579 1.00 64.23

ATOM 2457 OH TYR A 155 9.122 38.765 102.698 1.00 66.47

ATOM 2458 H TYR A 155 9.029 34.140 96.015 1.00 0.00

ATOM 2459 H TYR A 155 9.486 36.815 96.624 1.00 0.00

ATOM 2460 H TYR A 155 8.658 34.489 98.443 1.00 0.00

ATOM 2461 H TYR A 155 7.620 35.730 97.650 1.00 0.00

ATOM 2462 H TYR A 155 8.137 38.221 98.293 1.00 0.00

ATOM 2463 H TYR A 155 9.174 34.795 100.666 1.00 0.00

ATOM 2464 H TYR A 155 8.434 39.676 100.301 1.00 0.00

ATOM 2465 H TYR A 155 9.529 36.237 102.623 1.00 0.00

ATOM 2466 H TYR A 155 8.910 39.739 102.438 1.00 0.00

ATOM 2467 N TYR A 156 11.518 34.899 98.434 1.00 58.75

ATOM 2468 CA TYR A 156 12.770 34.911 99.185 1.00 59.04

ATOM 2469 C TYR A 156 13.894 35.185 98.283 1.00 59.49

ATOM 2470 O TYR A 156 14.823 35.843 98.694 1.00 60.87

ATOM 2471 CB TYR A 156 13.080 33.596 99.848 1.00 58.59

ATOM 2472 CG TYR A 156 12.191 33.298 101.002 1.00 60.02

ATOM 2473 CD1 TYR A 156 12.202 34.094 102.126 1.00 60.59

ATOM 2474 CD2 TYR A 156 11.357 32.214 100.967 1.00 57.08

ATOM 2475 CE1 TYR A 156 11.386 33.827 103.161 1.00 61.46

ATOM 2476 CE2 TYR A 156 10.547 31.941 101.995 1.00 57.92

ATOM 2477 CZ TYR A 156 10.553 32.748 103.089 1.00 60.11

ATOM 2478 OH TYR A 156 9.730 32.442 104.143 1.00 59.65

ATOM 2479 H TYR A 156 10.983 34.022 98.362 1.00 0.00

ATOM 2480 H TYR A 156 12.643 35.680 99.947 1.00 0.00

ATOM 2481 H TYR A 156 14.103 33.637 100.221 1.00 0.00

ATOM 2482 H TYR A 156 12.923 32.809 99.110 1.00 0.00

ATOM 2483 H TYR A 156 12.878 34.948 102.177 1.00 0.00

ATOM 2484 H TYR A 156 11.353 31.565 100.091 1.00 0.00

ATOM 2485 H TYR A 156 11.390 34.464 104.046 1.00 0.00

ATOM 2486 H TYR A 156 9.885 31.076 101.956 1.00 0.00

ATOM 2487 H TYR A 156 9.901 33.148 104.874 1.00 0.00

ATOM 2488 N ALA A 157 13.857 34.666 97.067 1.00 59.60

ATOM 2489 CA ALA A 157 15.018 34.877 96.226 1.00 60.95

ATOM 2490 C ALA A 157 15.079 36.364 95.857 1.00 62.88

ATOM 2491 O ALA A 157 16.155 36.969 95.825 1.00 65.20

ATOM 2492 CB ALA A 157 15.009 33.967 95.038 1.00 60.80

ATOM 2493 H ALA A 157 13.040 34.136 96.733 1.00 0.00

ATOM 2494 H ALA A 157 15.930 34.620 96.765 1.00 0.00

ATOM 2495 H ALA A 157 15.908 34.187 94.461 1.00 0.00

ATOM 2496 H ALA A 157 15.012 32.946 95.419 1.00 0.00

ATOM 2497 H ALA A 157 14.102 34.187 94.475 1.00 0.00

ATOM 2498 N ASN A 158 13.937 37.006 95.674 1.00 63.29

ATOM 2499 CA ASN A 158 13.997 38.460 95.578 1.00 66.03

ATOM 2500 C ASN A 158 14.436 39.188 96.851 1.00 66.00

ATOM 2501 O ASN A 158 15.043 40.262 96.776 1.00 67.29

ATOM 2502 CB ASN A 158 12.683 39.049 95.068 1.00 67.85

ATOM 2503 CG ASN A 158 12.380 38.653 93.626 1.00 71.14

ATOM 2504 OD1 ASN A 158 13.250 38.656 92.726 1.00 70.43

ATOM 2505 ND2 ASN A 158 11.131 38.256 93.410 1.00 75.60

ATOM 2506 H ASN A 158 13.043 36.500 95.602 1.00 0.00

ATOM 2507 H ASN A 158 14.790 38.635 94.851 1.00 0.00

ATOM 2508 H ASN A 158 12.765 40.135 95.106 1.00 0.00

ATOM 2509 H ASN A 158 11.879 38.665 95.695 1.00 0.00

ATOM 2510 H ASN A 158 10.838 37.970 92.465 1.00 0.00

ATOM 2511 H ASN A 158 10.456 38.234 94.188 1.00 0.00

ATOM 2512 N LYS A 159 14.096 38.647 98.021 1.00 65.05

ATOM 2513 CA LYS A 159 14.467 39.316 99.260 1.00 65.54

ATOM 2514 C LYS A 159 15.962 39.351 99.346 1.00 65.03

ATOM 2515 O LYS A 159 16.574 40.250 99.973 1.00 67.16

ATOM 2516 CB LYS A 159 13.888 38.608 100.469 1.00 64.87

ATOM 2517 CG LYS A 159 12.450 39.001 100.741 1.00 68.80

ATOM 2518 CD LYS A 159 12.323 40.511 100.978 1.00 72.96

ATOM 2519 CE LYS A 159 10.905 41.006 100.633 1.00 78.78

ATOM 2520 NZ LYS A 159 10.387 42.074 101.550 1.00 79.46

ATOM 2521 H LYS A 159 13.573 37.760 98.048 1.00 0.00

ATOM 2522 H LYS A 159 14.062 40.328 99.255 1.00 0.00

ATOM 2523 H LYS A 159 14.483 38.888 101.339 1.00 0.00

ATOM 2524 H LYS A 159 13.911 37.536 100.273 1.00 0.00

ATOM 2525 H LYS A 159 12.120 38.479 101.639 1.00 0.00

ATOM 2526 H LYS A 159 11.844 38.723 99.878 1.00 0.00

ATOM 2527 H LYS A 159 13.039 41.024 100.336 1.00 0.00

ATOM 2528 H LYS A 159 12.524 40.718 102.029 1.00 0.00

ATOM 2529 H LYS A 159 10.220 40.161 100.705 1.00 0.00

ATOM 2530 H LYS A 159 10.955 41.458 99.642 1.00 0.00

ATOM 2531 H LYS A 159 9.442 42.320 101.222 1.00 0.00

ATOM 2532 H LYS A 159 10.371 41.674 102.499 1.00 0.00

ATOM 2533 H LYS A 159 11.036 42.871 101.481 1.00 0.00

ATOM 2534 N TYR A 160 16.525 38.370 98.674 1.00 63.01

ATOM 2535 CA TYR A 160 17.912 37.995 98.744 1.00 63.10

ATOM 2536 C TYR A 160 18.766 38.906 97.890 1.00 64.13

ATOM 2537 O TYR A 160 19.720 39.537 98.367 1.00 64.76

ATOM 2538 CB TYR A 160 18.018 36.589 98.160 1.00 62.57

ATOM 2539 CG TYR A 160 19.344 35.964 98.323 1.00 63.08

ATOM 2540 CD1 TYR A 160 19.782 35.569 99.590 1.00 63.23

ATOM 2541 CD2 TYR A 160 20.193 35.792 97.223 1.00 63.41

ATOM 2542 CE1 TYR A 160 21.018 34.977 99.743 1.00 67.41

ATOM 2543 CE2 TYR A 160 21.430 35.188 97.371 1.00 64.74

ATOM 2544 CZ TYR A 160 21.833 34.798 98.619 1.00 65.25

ATOM 2545 OH TYR A 160 23.042 34.227 98.787 1.00 69.88

ATOM 2546 H TYR A 160 15.921 37.824 98.044 1.00 0.00

ATOM 2547 H TYR A 160 18.255 38.056 99.777 1.00 0.00

ATOM 2548 H TYR A 160 17.817 36.641 97.090 1.00 0.00

ATOM 2549 H TYR A 160 17.324 35.958 98.715 1.00 0.00

ATOM 2550 H TYR A 160 19.145 35.729 100.460 1.00 0.00

ATOM 2551 H TYR A 160 19.876 36.138 96.239 1.00 0.00

ATOM 2552 H TYR A 160 21.356 34.652 100.727 1.00 0.00

ATOM 2553 H TYR A 160 22.073 35.026 96.506 1.00 0.00

ATOM 2554 H TYR A 160 23.139 34.016 99.791 1.00 0.00

ATOM 2555 N ASN A 161 18.459 38.958 96.607 1.00 64.34

ATOM 2556 CA ASN A 161 19.059 40.058 95.801 1.00 66.21

ATOM 2557 C ASN A 161 18.889 41.407 96.509 1.00 65.74

ATOM 2558 O ASN A 161 19.734 42.234 96.396 1.00 66.04

ATOM 2559 CB ASN A 161 18.547 40.086 94.346 1.00 66.78

ATOM 2560 CG ASN A 161 19.185 38.996 93.467 1.00 69.07

ATOM 2561 OD1 ASN A 161 19.578 37.931 93.943 1.00 68.23

ATOM 2562 ND2 ASN A 161 19.279 39.270 92.160 1.00 72.21

ATOM 2563 H ASN A 161 17.829 38.265 96.180 1.00 0.00

ATOM 2564 H ASN A 161 20.127 39.856 95.726 1.00 0.00

ATOM 2565 H ASN A 161 18.807 41.054 93.918 1.00 0.00

ATOM 2566 H ASN A 161 17.472 39.907 94.367 1.00 0.00

ATOM 2567 H ASN A 161 19.697 38.581 91.518 1.00 0.00

ATOM 2568 H ASN A 161 18.934 40.170 91.796 1.00 0.00

ATOM 2569 N GLY A 162 17.804 41.575 97.266 1.00 66.50

ATOM 2570 CA GLY A 162 17.509 42.803 98.081 1.00 68.35

ATOM 2571 C GLY A 162 18.580 43.153 99.122 1.00 69.77

ATOM 2572 O GLY A 162 19.034 44.321 99.250 1.00 72.03

ATOM 2573 H GLY A 162 17.118 40.807 97.294 1.00 0.00

ATOM 2574 H GLY A 162 16.592 42.588 98.629 1.00 0.00

ATOM 2575 H GLY A 162 17.437 43.637 97.383 1.00 0.00

ATOM 2576 N VAL A 163 19.024 42.138 99.852 1.00 68.43

ATOM 2577 CA VAL A 163 20.113 42.320 100.800 1.00 69.13

ATOM 2578 C VAL A 163 21.381 42.798 100.103 1.00 69.91

ATOM 2579 O VAL A 163 22.019 43.768 100.563 1.00 72.06

ATOM 2580 CB VAL A 163 20.418 41.015 101.541 1.00 68.34

ATOM 2581 CG1 VAL A 163 21.477 41.280 102.541 1.00 69.48

ATOM 2582 CG2 VAL A 163 19.155 40.445 102.217 1.00 66.88

ATOM 2583 H VAL A 163 18.592 41.209 99.746 1.00 0.00

ATOM 2584 H VAL A 163 19.790 43.078 101.514 1.00 0.00

ATOM 2585 H VAL A 163 20.761 40.266 100.827 1.00 0.00

ATOM 2586 H VAL A 163 21.675 40.338 103.053 1.00 0.00

ATOM 2587 H VAL A 163 22.350 41.635 101.994 1.00 0.00

ATOM 2588 H VAL A 163 21.090 42.038 103.222 1.00 0.00

ATOM 2589 H VAL A 163 19.451 39.524 102.720 1.00 0.00

ATOM 2590 H VAL A 163 18.804 41.198 102.923 1.00 0.00

ATOM 2591 H VAL A 163 18.427 40.260 101.427 1.00 0.00

ATOM 2592 N PHE A 164 21.753 42.125 99.009 1.00 68.50

ATOM 2593 CA PHE A 164 22.976 42.484 98.236 1.00 70.16

ATOM 2594 C PHE A 164 22.880 43.823 97.564 1.00 72.38

ATOM 2595 O PHE A 164 23.863 44.556 97.501 1.00 74.33

ATOM 2596 CB PHE A 164 23.374 41.411 97.224 1.00 68.52

ATOM 2597 CG PHE A 164 23.878 40.190 97.879 1.00 67.95

ATOM 2598 CD1 PHE A 164 25.208 40.088 98.229 1.00 67.56

ATOM 2599 CD2 PHE A 164 23.002 39.165 98.233 1.00 65.73

ATOM 2600 CE1 PHE A 164 25.666 38.980 98.882 1.00 66.80

ATOM 2601 CE2 PHE A 164 23.458 38.056 98.915 1.00 64.23

ATOM 2602 CZ PHE A 164 24.786 37.963 99.238 1.00 63.91

ATOM 2603 H PHE A 164 21.176 41.333 98.690 1.00 0.00

ATOM 2604 H PHE A 164 23.766 42.549 98.985 1.00 0.00

ATOM 2605 H PHE A 164 24.159 41.795 96.572 1.00 0.00

ATOM 2606 H PHE A 164 22.482 41.123 96.667 1.00 0.00

ATOM 2607 H PHE A 164 25.897 40.896 97.983 1.00 0.00

ATOM 2608 H PHE A 164 21.947 39.241 97.969 1.00 0.00

ATOM 2609 H PHE A 164 26.725 38.892 99.125 1.00 0.00

ATOM 2610 H PHE A 164 22.767 37.260 99.195 1.00 0.00

ATOM 2611 H PHE A 164 25.154 37.089 99.776 1.00 0.00

ATOM 2612 N GLN A 165 21.691 44.139 97.069 1.00 73.13

ATOM 2613 CA GLN A 165 21.430 45.452 96.555 1.00 76.36

ATOM 2614 C GLN A 165 21.758 46.523 97.628 1.00 78.58

ATOM 2615 O GLN A 165 22.489 47.421 97.299 1.00 79.63

ATOM 2616 CB GLN A 165 20.018 45.556 95.971 1.00 75.87

ATOM 2617 CG GLN A 165 19.739 46.774 95.083 1.00 81.21

ATOM 2618 CD GLN A 165 20.934 47.223 94.190 1.00 84.93

ATOM 2619 OE1 GLN A 165 20.947 46.964 92.991 1.00 87.64

ATOM 2620 NE2 GLN A 165 21.929 47.880 94.785 1.00 85.79

ATOM 2621 H GLN A 165 20.944 43.430 97.053 1.00 0.00

ATOM 2622 H GLN A 165 22.095 45.650 95.714 1.00 0.00

ATOM 2623 H GLN A 165 19.343 45.640 96.823 1.00 0.00

ATOM 2624 H GLN A 165 19.887 44.680 95.336 1.00 0.00

ATOM 2625 H GLN A 165 19.524 47.600 95.761 1.00 0.00

ATOM 2626 H GLN A 165 18.919 46.499 94.420 1.00 0.00

ATOM 2627 H GLN A 165 22.740 48.196 94.234 1.00 0.00

ATOM 2628 H GLN A 165 21.887 48.072 95.796 1.00 0.00

ATOM 2629 N GLU A 166 21.244 46.391 98.872 1.00 79.46

ATOM 2630 CA GLU A 166 21.557 47.276 100.031 1.00 82.26

ATOM 2631 C GLU A 166 22.929 47.046 100.639 1.00 83.88

ATOM 2632 O GLU A 166 23.599 48.005 100.991 1.00 87.47

ATOM 2633 CB GLU A 166 20.471 47.240 101.161 1.00 82.35

ATOM 2634 CG GLU A 166 20.514 45.974 102.133 1.00 81.08

ATOM 2635 CD GLU A 166 19.247 45.730 103.013 1.00 81.19

ATOM 2636 OE1 GLU A 166 18.600 44.636 102.877 1.00 78.68

ATOM 2637 OE2 GLU A 166 18.920 46.603 103.866 1.00 82.36

ATOM 2638 H GLU A 166 20.583 45.617 99.032 1.00 0.00

ATOM 2639 H GLU A 166 21.556 48.271 99.585 1.00 0.00

ATOM 2640 H GLU A 166 19.513 47.196 100.643 1.00 0.00

ATOM 2641 H GLU A 166 20.641 48.123 101.777 1.00 0.00

ATOM 2642 H GLU A 166 21.339 46.132 102.828 1.00 0.00

ATOM 2643 H GLU A 166 20.585 45.099 101.487 1.00 0.00

ATOM 2644 N CYS A 167 23.385 45.803 100.779 1.00 83.62

ATOM 2645 CA CYS A 167 24.697 45.620 101.448 1.00 85.26

ATOM 2646 C CYS A 167 26.003 45.699 100.651 1.00 85.91

ATOM 2647 O CYS A 167 27.005 46.043 101.248 1.00 87.83

ATOM 2648 CB CYS A 167 24.730 44.352 102.327 1.00 83.62

ATOM 2649 SG CYS A 167 23.619 44.570 103.751 1.00 86.73

ATOM 2650 H CYS A 167 22.846 44.996 100.435 1.00 0.00

ATOM 2651 H CYS A 167 24.712 46.546 102.023 1.00 0.00

ATOM 2652 H CYS A 167 25.758 44.325 102.688 1.00 0.00

ATOM 2653 H CYS A 167 24.543 43.484 101.694 1.00 0.00

ATOM 2654 N CYS A 168 26.032 45.397 99.352 1.00 85.21

ATOM 2655 CA CYS A 168 27.330 45.435 98.641 1.00 87.12

ATOM 2656 C CYS A 168 27.857 46.828 98.366 1.00 89.45

ATOM 2657 O CYS A 168 28.884 46.946 97.704 1.00 91.82

ATOM 2658 CB CYS A 168 27.329 44.673 97.316 1.00 86.56

ATOM 2659 SG CYS A 168 27.112 42.883 97.437 1.00 86.44

ATOM 2660 H CYS A 168 25.168 45.142 98.853 1.00 0.00

ATOM 2661 H CYS A 168 27.992 44.940 99.352 1.00 0.00

ATOM 2662 H CYS A 168 28.367 44.818 97.016 1.00 0.00

ATOM 2663 H CYS A 168 26.582 45.170 96.698 1.00 0.00

ATOM 2664 N GLN A 169 27.146 47.858 98.845 1.00 90.05

ATOM 2665 CA GLN A 169 27.552 49.274 98.731 1.00 92.00

ATOM 2666 C GLN A 169 28.201 49.823 100.030 1.00 92.95

ATOM 2667 O GLN A 169 28.762 50.912 100.025 1.00 95.16

ATOM 2668 CB GLN A 169 26.362 50.172 98.273 1.00 92.85

ATOM 2669 CG GLN A 169 26.774 51.553 97.577 1.00 95.88

ATOM 2670 CD GLN A 169 25.590 52.484 97.177 1.00 95.84

ATOM 2671 OE1 GLN A 169 24.417 52.139 97.304 1.00 96.55

ATOM 2672 NE2 GLN A 169 25.921 53.656 96.691 1.00 95.31

ATOM 2673 H GLN A 169 26.258 47.649 99.324 1.00 0.00

ATOM 2674 H GLN A 169 28.322 49.310 97.960 1.00 0.00

ATOM 2675 H GLN A 169 25.824 50.431 99.185 1.00 0.00

ATOM 2676 H GLN A 169 25.795 49.590 97.546 1.00 0.00

ATOM 2677 H GLN A 169 27.304 51.314 96.655 1.00 0.00

ATOM 2678 H GLN A 169 27.346 52.114 98.317 1.00 0.00

ATOM 2679 H GLN A 169 25.188 54.321 96.406 1.00 0.00

ATOM 2680 H GLN A 169 26.914 53.911 96.594 1.00 0.00

ATOM 2681 N ALA A 170 28.133 49.057 101.117 1.00 91.54

ATOM 2682 CA ALA A 170 28.634 49.484 102.431 1.00 93.40

ATOM 2683 C ALA A 170 30.124 49.203 102.549 1.00 94.88

ATOM 2684 O ALA A 170 30.671 48.456 101.726 1.00 93.95

ATOM 2685 CB ALA A 170 27.854 48.801 103.565 1.00 91.82

ATOM 2686 H ALA A 170 27.710 48.122 101.033 1.00 0.00

ATOM 2687 H ALA A 170 28.481 50.559 102.523 1.00 0.00

ATOM 2688 H ALA A 170 28.275 49.161 104.504 1.00 0.00

ATOM 2689 H ALA A 170 26.809 49.090 103.452 1.00 0.00

ATOM 2690 H ALA A 170 27.992 47.727 103.445 1.00 0.00

ATOM 2691 N GLU A 171 30.778 49.798 103.554 1.00 97.42

ATOM 2692 CA GLU A 171 32.250 49.682 103.709 1.00100.12

ATOM 2693 C GLU A 171 32.717 48.408 104.440 1.00 98.88

ATOM 2694 O GLU A 171 33.919 48.111 104.466 1.00101.53

ATOM 2695 CB GLU A 171 32.876 50.908 104.410 1.00103.81

ATOM 2696 CG GLU A 171 32.284 52.285 104.047 1.00107.73

ATOM 2697 CD GLU A 171 30.955 52.606 104.779 1.00109.37

ATOM 2698 OE1 GLU A 171 30.857 53.742 105.329 1.00112.70

ATOM 2699 OE2 GLU A 171 30.025 51.741 104.780 1.00105.51

ATOM 2700 H GLU A 171 30.245 50.352 104.239 1.00 0.00

ATOM 2701 H GLU A 171 32.604 49.626 102.680 1.00 0.00

ATOM 2702 H GLU A 171 33.920 50.936 104.097 1.00 0.00

ATOM 2703 H GLU A 171 32.691 50.773 105.476 1.00 0.00

ATOM 2704 H GLU A 171 32.072 52.272 102.978 1.00 0.00

ATOM 2705 H GLU A 171 33.013 53.040 104.340 1.00 0.00

ATOM 2706 N ASP A 172 31.788 47.714 105.079 1.00 95.33

ATOM 2707 CA ASP A 172 32.034 46.388 105.607 1.00 93.34

ATOM 2708 C ASP A 172 30.887 45.517 105.116 1.00 89.07

ATOM 2709 O ASP A 172 29.922 45.345 105.859 1.00 87.78

ATOM 2710 CB ASP A 172 32.046 46.409 107.149 1.00 95.36

ATOM 2711 CG ASP A 172 32.705 45.158 107.769 1.00 95.57

ATOM 2712 OD1 ASP A 172 32.438 44.014 107.303 1.00 89.51

ATOM 2713 OD2 ASP A 172 33.514 45.342 108.726 1.00 99.11

ATOM 2714 H ASP A 172 30.855 48.133 105.205 1.00 0.00

ATOM 2715 H ASP A 172 33.002 46.011 105.277 1.00 0.00

ATOM 2716 H ASP A 172 31.010 46.438 107.486 1.00 0.00

ATOM 2717 H ASP A 172 32.624 47.278 107.463 1.00 0.00

ATOM 2718 N LYS A 173 30.978 45.024 103.866 1.00 86.36

ATOM 2719 CA LYS A 173 30.059 44.024 103.325 1.00 83.27

ATOM 2720 C LYS A 173 29.771 42.947 104.376 1.00 82.47

ATOM 2721 O LYS A 173 28.600 42.718 104.723 1.00 81.04

ATOM 2722 CB LYS A 173 30.571 43.363 102.020 1.00 82.43

ATOM 2723 CG LYS A 173 31.020 44.374 100.918 1.00 85.60

ATOM 2724 CD LYS A 173 31.399 43.767 99.530 1.00 83.60

ATOM 2725 CE LYS A 173 32.767 43.071 99.480 1.00 86.01

ATOM 2726 NZ LYS A 173 33.959 43.993 99.730 1.00 88.73

ATOM 2727 H LYS A 173 31.735 45.370 103.259 1.00 0.00

ATOM 2728 H LYS A 173 29.141 44.554 103.070 1.00 0.00

ATOM 2729 H LYS A 173 29.738 42.792 101.610 1.00 0.00

ATOM 2730 H LYS A 173 31.441 42.763 102.286 1.00 0.00

ATOM 2731 H LYS A 173 31.924 44.851 101.296 1.00 0.00

ATOM 2732 H LYS A 173 30.165 45.026 100.740 1.00 0.00

ATOM 2733 H LYS A 173 31.436 44.586 98.812 1.00 0.00

ATOM 2734 H LYS A 173 30.660 42.997 99.310 1.00 0.00

ATOM 2735 H LYS A 173 32.885 42.664 98.476 1.00 0.00

ATOM 2736 H LYS A 173 32.774 42.326 100.275 1.00 0.00

ATOM 2737 H LYS A 173 34.807 43.412 99.670 1.00 0.00

ATOM 2738 H LYS A 173 33.939 44.717 98.998 1.00 0.00

ATOM 2739 H LYS A 173 33.836 44.396 100.670 1.00 0.00

ATOM 2740 N GLY A 174 30.825 42.321 104.908 1.00 83.65

ATOM 2741 CA GLY A 174 30.677 41.200 105.857 1.00 83.11

ATOM 2742 C GLY A 174 29.708 41.500 106.983 1.00 82.52

ATOM 2743 O GLY A 174 28.670 40.866 107.117 1.00 80.35

ATOM 2744 H GLY A 174 31.772 42.631 104.647 1.00 0.00

ATOM 2745 H GLY A 174 31.659 41.035 106.300 1.00 0.00

ATOM 2746 H GLY A 174 30.311 40.339 105.298 1.00 0.00

ATOM 2747 N ALA A 175 30.046 42.512 107.761 1.00 85.38

ATOM 2748 CA ALA A 175 29.222 42.975 108.856 1.00 86.76

ATOM 2749 C ALA A 175 27.785 43.325 108.456 1.00 85.11

ATOM 2750 O ALA A 175 26.894 43.309 109.299 1.00 84.86

ATOM 2751 CB ALA A 175 29.884 44.163 109.526 1.00 90.33

ATOM 2752 H ALA A 175 30.938 42.994 107.580 1.00 0.00

ATOM 2753 H ALA A 175 29.139 42.140 109.551 1.00 0.00

ATOM 2754 H ALA A 175 29.230 44.475 110.340 1.00 0.00

ATOM 2755 H ALA A 175 30.853 43.823 109.892 1.00 0.00

ATOM 2756 H ALA A 175 29.987 44.940 108.768 1.00 0.00

ATOM 2757 N CYS A 176 27.556 43.660 107.192 1.00 84.49

ATOM 2758 CA CYS A 176 26.212 44.013 106.763 1.00 84.39

ATOM 2759 C CYS A 176 25.435 42.771 106.346 1.00 81.06

ATOM 2760 O CYS A 176 24.234 42.671 106.647 1.00 80.47

ATOM 2761 CB CYS A 176 26.209 45.068 105.638 1.00 85.44

ATOM 2762 SG CYS A 176 24.504 45.770 105.162 1.00 89.48

ATOM 2763 H CYS A 176 28.331 43.670 106.514 1.00 0.00

ATOM 2764 H CYS A 176 25.713 44.466 107.620 1.00 0.00

ATOM 2765 H CYS A 176 26.483 44.417 104.807 1.00 0.00

ATOM 2766 H CYS A 176 26.961 45.797 105.939 1.00 0.00

ATOM 2767 N LEU A 177 26.129 41.813 105.715 1.00 80.05

ATOM 2768 CA LEU A 177 25.476 40.684 105.006 1.00 77.36

ATOM 2769 C LEU A 177 25.035 39.525 105.843 1.00 75.65

ATOM 2770 O LEU A 177 23.894 39.094 105.786 1.00 74.73

ATOM 2771 CB LEU A 177 26.384 40.130 103.897 1.00 77.68

ATOM 2772 CG LEU A 177 26.356 40.942 102.588 1.00 79.75

ATOM 2773 CD1 LEU A 177 27.522 40.579 101.685 1.00 79.90

ATOM 2774 CD2 LEU A 177 25.007 40.744 101.857 1.00 77.76

ATOM 2775 H LEU A 177 27.158 41.862 105.722 1.00 0.00

ATOM 2776 H LEU A 177 24.569 41.146 104.617 1.00 0.00

ATOM 2777 H LEU A 177 26.023 39.129 103.661 1.00 0.00

ATOM 2778 H LEU A 177 27.406 40.162 104.274 1.00 0.00

ATOM 2779 H LEU A 177 26.458 41.997 102.844 1.00 0.00

ATOM 2780 H LEU A 177 27.432 41.191 100.787 1.00 0.00

ATOM 2781 H LEU A 177 28.435 40.802 102.238 1.00 0.00

ATOM 2782 H LEU A 177 27.433 39.516 101.461 1.00 0.00

ATOM 2783 H LEU A 177 25.048 41.339 100.945 1.00 0.00

ATOM 2784 H LEU A 177 24.914 39.679 101.642 1.00 0.00

ATOM 2785 H LEU A 177 24.225 41.091 102.532 1.00 0.00

ATOM 2786 N LEU A 178 25.962 38.999 106.613 1.00 76.54

ATOM 2787 CA LEU A 178 25.789 37.685 107.202 1.00 74.65

ATOM 2788 C LEU A 178 24.649 37.647 108.218 1.00 73.57

ATOM 2789 O LEU A 178 23.999 36.610 108.341 1.00 71.82

ATOM 2790 CB LEU A 178 27.123 37.214 107.820 1.00 77.11

ATOM 2791 CG LEU A 178 28.332 37.445 106.896 1.00 76.86

ATOM 2792 CD1 LEU A 178 29.622 37.586 107.696 1.00 81.68

ATOM 2793 CD2 LEU A 178 28.448 36.350 105.827 1.00 75.42

ATOM 2794 H LEU A 178 26.825 39.529 106.801 1.00 0.00

ATOM 2795 H LEU A 178 25.504 36.993 106.410 1.00 0.00

ATOM 2796 H LEU A 178 27.046 36.142 108.002 1.00 0.00

ATOM 2797 H LEU A 178 27.289 37.792 108.729 1.00 0.00

ATOM 2798 H LEU A 178 28.166 38.387 106.373 1.00 0.00

ATOM 2799 H LEU A 178 30.426 37.746 106.978 1.00 0.00

ATOM 2800 H LEU A 178 29.494 38.442 108.359 1.00 0.00

ATOM 2801 H LEU A 178 29.754 36.659 108.253 1.00 0.00

ATOM 2802 H LEU A 178 29.322 36.588 105.221 1.00 0.00

ATOM 2803 H LEU A 178 28.565 35.402 106.353 1.00 0.00

ATOM 2804 H LEU A 178 27.529 36.378 105.241 1.00 0.00

ATOM 2805 N PRO A 179 24.406 38.765 108.945 1.00 75.11

ATOM 2806 CA PRO A 179 23.275 38.770 109.903 1.00 75.21

ATOM 2807 C PRO A 179 21.943 38.717 109.158 1.00 72.71

ATOM 2808 O PRO A 179 21.023 37.994 109.582 1.00 70.68

ATOM 2809 CB PRO A 179 23.422 40.094 110.641 1.00 78.02

ATOM 2810 CG PRO A 179 24.751 40.675 110.205 1.00 79.04

ATOM 2811 CD PRO A 179 25.081 40.079 108.888 1.00 76.87

ATOM 2812 H PRO A 179 23.288 37.911 110.574 1.00 0.00

ATOM 2813 H PRO A 179 23.391 39.952 111.721 1.00 0.00

ATOM 2814 H PRO A 179 22.603 40.772 110.400 1.00 0.00

ATOM 2815 H PRO A 179 25.536 40.461 110.930 1.00 0.00

ATOM 2816 H PRO A 179 24.683 41.759 110.112 1.00 0.00

ATOM 2817 H PRO A 179 24.746 40.690 108.050 1.00 0.00

ATOM 2818 H PRO A 179 26.154 39.998 108.714 1.00 0.00

ATOM 2819 N LYS A 180 21.886 39.405 108.007 1.00 71.60

ATOM 2820 CA LYS A 180 20.680 39.372 107.183 1.00 69.72

ATOM 2821 C LYS A 180 20.452 38.030 106.456 1.00 68.31

ATOM 2822 O LYS A 180 19.292 37.546 106.407 1.00 67.04

ATOM 2823 CB LYS A 180 20.558 40.572 106.246 1.00 69.17

ATOM 2824 CG LYS A 180 20.302 41.906 106.960 1.00 71.17

ATOM 2825 CD LYS A 180 20.170 43.039 105.961 1.00 71.42

ATOM 2826 CE LYS A 180 20.295 44.421 106.582 1.00 79.41

ATOM 2827 NZ LYS A 180 19.244 44.784 107.599 1.00 80.66

ATOM 2828 H LYS A 180 22.698 39.960 107.702 1.00 0.00

ATOM 2829 H LYS A 180 19.860 39.456 107.897 1.00 0.00

ATOM 2830 H LYS A 180 19.701 40.384 105.600 1.00 0.00

ATOM 2831 H LYS A 180 21.506 40.664 105.717 1.00 0.00

ATOM 2832 H LYS A 180 21.137 42.122 107.627 1.00 0.00

ATOM 2833 H LYS A 180 19.368 41.831 107.517 1.00 0.00

ATOM 2834 H LYS A 180 19.175 42.968 105.522 1.00 0.00

ATOM 2835 H LYS A 180 20.978 42.931 105.238 1.00 0.00

ATOM 2836 H LYS A 180 20.196 45.140 105.769 1.00 0.00

ATOM 2837 H LYS A 180 21.245 44.432 107.116 1.00 0.00

ATOM 2838 H LYS A 180 19.458 45.737 107.926 1.00 0.00

ATOM 2839 H LYS A 180 18.333 44.739 107.120 1.00 0.00

ATOM 2840 H LYS A 180 19.311 44.094 108.360 1.00 0.00

ATOM 2841 N ILE A 181 21.504 37.403 105.914 1.00 68.53

ATOM 2842 CA ILE A 181 21.236 36.072 105.328 1.00 67.42

ATOM 2843 C ILE A 181 20.883 35.085 106.389 1.00 67.53

ATOM 2844 O ILE A 181 19.945 34.320 106.196 1.00 66.55

ATOM 2845 CB ILE A 181 22.231 35.537 104.256 1.00 66.03

ATOM 2846 CG1 ILE A 181 23.596 35.216 104.821 1.00 69.87

ATOM 2847 CG2 ILE A 181 22.338 36.528 103.155 1.00 67.13

ATOM 2848 CD1 ILE A 181 24.760 35.639 103.919 1.00 69.50

ATOM 2849 H ILE A 181 22.444 37.823 105.906 1.00 0.00

ATOM 2850 H ILE A 181 20.360 36.230 104.699 1.00 0.00

ATOM 2851 H ILE A 181 21.834 34.595 103.878 1.00 0.00

ATOM 2852 H ILE A 181 23.645 34.132 104.924 1.00 0.00

ATOM 2853 H ILE A 181 23.691 35.756 105.763 1.00 0.00

ATOM 2854 H ILE A 181 23.040 36.121 102.427 1.00 0.00

ATOM 2855 H ILE A 181 21.340 36.645 102.734 1.00 0.00

ATOM 2856 H ILE A 181 22.704 37.456 103.596 1.00 0.00

ATOM 2857 H ILE A 181 25.679 35.356 104.432 1.00 0.00

ATOM 2858 H ILE A 181 24.642 35.106 102.975 1.00 0.00

ATOM 2859 H ILE A 181 24.685 36.718 103.787 1.00 0.00

ATOM 2860 N GLU A 182 21.557 35.158 107.535 1.00 70.25

ATOM 2861 CA GLU A 182 21.300 34.248 108.649 1.00 71.82

ATOM 2862 C GLU A 182 19.879 34.360 109.208 1.00 70.98

ATOM 2863 O GLU A 182 19.247 33.342 109.491 1.00 71.61

ATOM 2864 CB GLU A 182 22.320 34.475 109.768 1.00 75.95

ATOM 2865 CG GLU A 182 23.677 33.840 109.509 1.00 82.02

ATOM 2866 CD GLU A 182 23.567 32.485 108.839 1.00 84.34

ATOM 2867 OE1 GLU A 182 22.821 31.625 109.352 1.00 83.82

ATOM 2868 OE2 GLU A 182 24.227 32.280 107.798 1.00 86.81

ATOM 2869 H GLU A 182 22.284 35.880 107.640 1.00 0.00

ATOM 2870 H GLU A 182 21.403 33.239 108.250 1.00 0.00

ATOM 2871 H GLU A 182 21.919 34.021 110.674 1.00 0.00

ATOM 2872 H GLU A 182 22.482 35.550 109.843 1.00 0.00

ATOM 2873 H GLU A 182 24.174 33.700 110.469 1.00 0.00

ATOM 2874 H GLU A 182 24.234 34.498 108.841 1.00 0.00

ATOM 2875 N THR A 183 19.366 35.580 109.355 1.00 71.71

ATOM 2876 CA THR A 183 17.956 35.747 109.736 1.00 71.46

ATOM 2877 C THR A 183 17.062 35.243 108.587 1.00 69.55

ATOM 2878 O THR A 183 15.930 34.703 108.801 1.00 68.83

ATOM 2879 CB THR A 183 17.569 37.202 109.955 1.00 73.52

ATOM 2880 OG1 THR A 183 18.551 37.853 110.763 1.00 77.35

ATOM 2881 CG2 THR A 183 16.181 37.312 110.632 1.00 73.00

ATOM 2882 H THR A 183 19.958 36.408 109.201 1.00 0.00

ATOM 2883 H THR A 183 17.822 35.191 110.664 1.00 0.00

ATOM 2884 H THR A 183 17.518 37.689 108.981 1.00 0.00

ATOM 2885 H THR A 183 19.454 37.755 110.277 1.00 0.00

ATOM 2886 H THR A 183 15.971 38.374 110.757 1.00 0.00

ATOM 2887 H THR A 183 15.463 36.832 109.967 1.00 0.00

ATOM 2888 H THR A 183 16.252 36.798 111.590 1.00 0.00

ATOM 2889 N MET A 184 17.561 35.395 107.368 1.00 68.10

ATOM 2890 CA MET A 184 16.780 34.970 106.256 1.00 67.50

ATOM 2891 C MET A 184 16.732 33.420 106.238 1.00 66.83

ATOM 2892 O MET A 184 15.631 32.833 106.227 1.00 66.31

ATOM 2893 CB MET A 184 17.315 35.565 104.948 1.00 66.96

ATOM 2894 CG MET A 184 16.470 35.221 103.742 1.00 67.01

ATOM 2895 SD MET A 184 17.050 35.851 102.132 1.00 69.09

ATOM 2896 CE MET A 184 16.276 34.685 101.088 1.00 60.85

ATOM 2897 H MET A 184 18.493 35.811 107.229 1.00 0.00

ATOM 2898 H MET A 184 15.759 35.339 106.355 1.00 0.00

ATOM 2899 H MET A 184 18.313 35.162 104.775 1.00 0.00

ATOM 2900 H MET A 184 17.298 36.650 105.049 1.00 0.00

ATOM 2901 H MET A 184 15.592 35.824 103.975 1.00 0.00

ATOM 2902 H MET A 184 16.346 34.139 103.790 1.00 0.00

ATOM 2903 H MET A 184 16.543 34.947 100.064 1.00 0.00

ATOM 2904 H MET A 184 16.658 33.703 101.368 1.00 0.00

ATOM 2905 H MET A 184 15.203 34.766 101.260 1.00 0.00

ATOM 2906 N ARG A 185 17.911 32.789 106.293 1.00 67.29

ATOM 2907 CA ARG A 185 18.072 31.324 106.199 1.00 66.61

ATOM 2908 C ARG A 185 17.205 30.571 107.219 1.00 66.30

ATOM 2909 O ARG A 185 16.610 29.529 106.933 1.00 66.41

ATOM 2910 CB ARG A 185 19.555 30.954 106.349 1.00 67.42

ATOM 2911 CG ARG A 185 19.850 29.477 106.485 1.00 66.27

ATOM 2912 CD ARG A 185 20.956 29.204 107.521 1.00 67.52

ATOM 2913 NE ARG A 185 21.323 27.788 107.531 1.00 69.56

ATOM 2914 CZ ARG A 185 21.640 27.090 108.620 1.00 70.12

ATOM 2915 NH1 ARG A 185 21.640 27.677 109.804 1.00 73.05

ATOM 2916 NH2 ARG A 185 21.959 25.805 108.524 1.00 67.35

ATOM 2917 H ARG A 185 18.760 33.361 106.408 1.00 0.00

ATOM 2918 H ARG A 185 17.723 31.013 105.214 1.00 0.00

ATOM 2919 H ARG A 185 19.913 31.426 107.264 1.00 0.00

ATOM 2920 H ARG A 185 20.048 31.272 105.430 1.00 0.00

ATOM 2921 H ARG A 185 20.196 29.121 105.515 1.00 0.00

ATOM 2922 H ARG A 185 18.936 28.974 106.801 1.00 0.00

ATOM 2923 H ARG A 185 20.630 29.487 108.522 1.00 0.00

ATOM 2924 H ARG A 185 21.850 29.757 107.232 1.00 0.00

ATOM 2925 H ARG A 185 21.337 27.293 106.628 1.00 0.00

ATOM 2926 H ARG A 185 21.885 27.137 110.646 1.00 0.00

ATOM 2927 H ARG A 185 21.395 28.674 109.884 1.00 0.00

ATOM 2928 H ARG A 185 22.203 25.270 109.370 1.00 0.00

ATOM 2929 H ARG A 185 21.962 25.343 107.604 1.00 0.00

ATOM 2930 N GLU A 186 17.098 31.100 108.409 1.00 67.90

ATOM 2931 CA GLU A 186 16.157 30.497 109.342 1.00 68.40

ATOM 2932 C GLU A 186 14.653 30.537 108.870 1.00 66.30

ATOM 2933 O GLU A 186 13.893 29.588 109.080 1.00 65.07

ATOM 2934 CB GLU A 186 16.398 31.041 110.763 1.00 71.14

ATOM 2935 CG GLU A 186 17.720 30.457 111.428 1.00 75.61

ATOM 2936 CD GLU A 186 17.478 29.496 112.665 1.00 83.44

ATOM 2937 OE1 GLU A 186 16.403 28.808 112.766 1.00 83.93

ATOM 2938 OE2 GLU A 186 18.382 29.431 113.552 1.00 84.84

ATOM 2939 H GLU A 186 17.662 31.919 108.679 1.00 0.00

ATOM 2940 H GLU A 186 16.361 29.427 109.366 1.00 0.00

ATOM 2941 H GLU A 186 15.550 30.719 111.367 1.00 0.00

ATOM 2942 H GLU A 186 16.479 32.126 110.696 1.00 0.00

ATOM 2943 H GLU A 186 18.293 31.308 111.797 1.00 0.00

ATOM 2944 H GLU A 186 18.221 29.862 110.664 1.00 0.00

ATOM 2945 N LYS A 187 14.235 31.617 108.217 1.00 65.40

ATOM 2946 CA LYS A 187 12.835 31.729 107.792 1.00 63.34

ATOM 2947 C LYS A 187 12.529 30.761 106.646 1.00 60.84

ATOM 2948 O LYS A 187 11.391 30.334 106.498 1.00 59.84

ATOM 2949 CB LYS A 187 12.499 33.159 107.362 1.00 63.86

ATOM 2950 CG LYS A 187 12.254 34.102 108.482 1.00 64.24

ATOM 2951 CD LYS A 187 12.362 35.504 107.978 1.00 67.00

ATOM 2952 CE LYS A 187 12.156 36.496 109.112 1.00 71.36

ATOM 2953 NZ LYS A 187 12.696 37.847 108.719 1.00 74.56

ATOM 2954 H LYS A 187 14.897 32.378 108.009 1.00 0.00

ATOM 2955 H LYS A 187 12.215 31.467 108.650 1.00 0.00

ATOM 2956 H LYS A 187 11.587 33.131 106.766 1.00 0.00

ATOM 2957 H LYS A 187 13.373 33.546 106.838 1.00 0.00

ATOM 2958 H LYS A 187 12.997 33.936 109.262 1.00 0.00

ATOM 2959 H LYS A 187 11.258 33.932 108.892 1.00 0.00

ATOM 2960 H LYS A 187 11.586 35.657 107.229 1.00 0.00

ATOM 2961 H LYS A 187 13.350 35.647 107.540 1.00 0.00

ATOM 2962 H LYS A 187 12.678 36.146 110.003 1.00 0.00

ATOM 2963 H LYS A 187 11.090 36.589 109.318 1.00 0.00

ATOM 2964 H LYS A 187 12.534 38.480 109.515 1.00 0.00

ATOM 2965 H LYS A 187 13.700 37.725 108.525 1.00 0.00

ATOM 2966 H LYS A 187 12.178 38.146 107.881 1.00 0.00

ATOM 2967 N VAL A 188 13.556 30.426 105.865 1.00 58.10

ATOM 2968 CA VAL A 188 13.426 29.625 104.664 1.00 55.26

ATOM 2969 C VAL A 188 13.318 28.186 105.131 1.00 54.74

ATOM 2970 O VAL A 188 12.454 27.471 104.722 1.00 52.97

ATOM 2971 CB VAL A 188 14.695 29.832 103.789 1.00 55.43

ATOM 2972 CG1 VAL A 188 15.090 28.595 102.931 1.00 52.70

ATOM 2973 CG2 VAL A 188 14.579 31.081 102.957 1.00 56.54

ATOM 2974 H VAL A 188 14.495 30.755 106.130 1.00 0.00

ATOM 2975 H VAL A 188 12.557 29.899 104.066 1.00 0.00

ATOM 2976 H VAL A 188 15.521 29.959 104.489 1.00 0.00

ATOM 2977 H VAL A 188 15.985 28.868 102.371 1.00 0.00

ATOM 2978 H VAL A 188 15.280 27.775 103.623 1.00 0.00

ATOM 2979 H VAL A 188 14.248 28.383 102.272 1.00 0.00

ATOM 2980 H VAL A 188 15.496 31.162 102.374 1.00 0.00

ATOM 2981 H VAL A 188 13.703 30.959 102.320 1.00 0.00

ATOM 2982 H VAL A 188 14.464 31.915 103.649 1.00 0.00

ATOM 2983 N LEU A 189 14.176 27.773 106.068 1.00 56.47

ATOM 2984 CA LEU A 189 14.070 26.403 106.546 1.00 54.82

ATOM 2985 C LEU A 189 12.735 26.253 107.226 1.00 54.11

ATOM 2986 O LEU A 189 12.068 25.273 107.055 1.00 54.31

ATOM 2987 CB LEU A 189 15.209 26.057 107.488 1.00 55.51

ATOM 2988 CG LEU A 189 16.647 26.129 106.990 1.00 55.97

ATOM 2989 CD1 LEU A 189 17.551 25.865 108.187 1.00 56.86

ATOM 2990 CD2 LEU A 189 17.028 25.212 105.764 1.00 48.58

ATOM 2991 H LEU A 189 14.895 28.409 106.443 1.00 0.00

ATOM 2992 H LEU A 189 14.142 25.710 105.707 1.00 0.00

ATOM 2993 H LEU A 189 15.053 25.005 107.729 1.00 0.00

ATOM 2994 H LEU A 189 15.150 26.806 108.278 1.00 0.00

ATOM 2995 H LEU A 189 16.782 27.127 106.572 1.00 0.00

ATOM 2996 H LEU A 189 18.579 25.917 107.828 1.00 0.00

ATOM 2997 H LEU A 189 17.339 26.640 108.923 1.00 0.00

ATOM 2998 H LEU A 189 17.302 24.872 108.561 1.00 0.00

ATOM 2999 H LEU A 189 18.081 25.391 105.547 1.00 0.00

ATOM 3000 H LEU A 189 16.847 24.181 106.069 1.00 0.00

ATOM 3001 H LEU A 189 16.387 25.508 104.933 1.00 0.00

ATOM 3002 N THR A 190 12.321 27.223 108.018 1.00 55.29

ATOM 3003 CA THR A 190 11.005 27.085 108.657 1.00 55.81

ATOM 3004 C THR A 190 9.866 26.981 107.598 1.00 55.11

ATOM 3005 O THR A 190 8.886 26.278 107.799 1.00 55.82

ATOM 3006 CB THR A 190 10.778 28.173 109.671 1.00 56.34

ATOM 3007 OG1 THR A 190 11.611 27.910 110.813 1.00 59.60

ATOM 3008 CG2 THR A 190 9.313 28.228 110.119 1.00 60.29

ATOM 3009 H THR A 190 12.905 28.055 108.184 1.00 0.00

ATOM 3010 H THR A 190 10.987 26.145 109.209 1.00 0.00

ATOM 3011 H THR A 190 11.026 29.132 109.216 1.00 0.00

ATOM 3012 H THR A 190 12.584 27.880 110.476 1.00 0.00

ATOM 3013 H THR A 190 9.233 29.035 110.847 1.00 0.00

ATOM 3014 H THR A 190 8.715 28.424 109.229 1.00 0.00

ATOM 3015 H THR A 190 9.078 27.259 110.560 1.00 0.00

ATOM 3016 N SER A 191 10.034 27.633 106.453 1.00 53.52

ATOM 3017 CA SER A 191 8.986 27.682 105.541 1.00 53.32

ATOM 3018 C SER A 191 8.938 26.360 104.747 1.00 52.58

ATOM 3019 O SER A 191 7.870 25.898 104.351 1.00 52.60

ATOM 3020 CB SER A 191 9.157 28.921 104.671 1.00 52.88

ATOM 3021 OG SER A 191 8.669 28.723 103.330 1.00 59.33

ATOM 3022 H SER A 191 10.930 28.097 106.243 1.00 0.00

ATOM 3023 H SER A 191 8.020 27.773 106.038 1.00 0.00

ATOM 3024 H SER A 191 10.209 29.198 104.607 1.00 0.00

ATOM 3025 H SER A 191 8.543 29.715 105.095 1.00 0.00

ATOM 3026 H SER A 191 8.824 29.604 102.820 1.00 0.00

ATOM 3027 N SER A 192 10.099 25.826 104.408 1.00 51.71

ATOM 3028 CA SER A 192 10.190 24.524 103.791 1.00 50.44

ATOM 3029 C SER A 192 9.676 23.391 104.692 1.00 50.18

ATOM 3030 O SER A 192 9.074 22.450 104.213 1.00 47.72

ATOM 3031 CB SER A 192 11.633 24.281 103.439 1.00 50.51

ATOM 3032 OG SER A 192 11.985 22.947 103.572 1.00 51.35

ATOM 3033 H SER A 192 10.964 26.355 104.589 1.00 0.00

ATOM 3034 H SER A 192 9.553 24.521 102.906 1.00 0.00

ATOM 3035 H SER A 192 12.305 24.869 104.064 1.00 0.00

ATOM 3036 H SER A 192 11.762 24.500 102.379 1.00 0.00

ATOM 3037 H SER A 192 12.979 22.871 103.314 1.00 0.00

ATOM 3038 N ALA A 193 9.954 23.466 105.987 1.00 51.18

ATOM 3039 CA ALA A 193 9.444 22.416 106.900 1.00 53.05

ATOM 3040 C ALA A 193 7.912 22.477 106.937 1.00 52.71

ATOM 3041 O ALA A 193 7.263 21.450 106.843 1.00 53.12

ATOM 3042 CB ALA A 193 10.029 22.550 108.309 1.00 53.57

ATOM 3043 H ALA A 193 10.518 24.246 106.354 1.00 0.00

ATOM 3044 H ALA A 193 9.761 21.445 106.519 1.00 0.00

ATOM 3045 H ALA A 193 9.604 21.745 108.908 1.00 0.00

ATOM 3046 H ALA A 193 11.111 22.457 108.215 1.00 0.00

ATOM 3047 H ALA A 193 9.737 23.531 108.683 1.00 0.00

ATOM 3048 N ARG A 194 7.356 23.692 107.039 1.00 53.35

ATOM 3049 CA ARG A 194 5.918 23.930 106.850 1.00 54.08

ATOM 3050 C ARG A 194 5.289 23.328 105.597 1.00 52.21

ATOM 3051 O ARG A 194 4.284 22.617 105.656 1.00 52.79

ATOM 3052 CB ARG A 194 5.627 25.427 106.910 1.00 56.51

ATOM 3053 CG ARG A 194 5.422 25.937 108.355 1.00 58.51

ATOM 3054 CD ARG A 194 4.846 27.339 108.416 1.00 58.00

ATOM 3055 NE ARG A 194 5.401 28.029 109.571 1.00 61.56

ATOM 3056 CZ ARG A 194 5.055 27.807 110.833 1.00 59.75

ATOM 3057 NH1 ARG A 194 4.131 26.905 111.088 1.00 57.22

ATOM 3058 NH2 ARG A 194 5.651 28.461 111.842 1.00 58.48

ATOM 3059 H ARG A 194 7.963 24.494 107.260 1.00 0.00

ATOM 3060 H ARG A 194 5.445 23.395 107.673 1.00 0.00

ATOM 3061 H ARG A 194 4.703 25.601 106.359 1.00 0.00

ATOM 3062 H ARG A 194 6.479 25.951 106.477 1.00 0.00

ATOM 3063 H ARG A 194 6.390 25.955 108.855 1.00 0.00

ATOM 3064 H ARG A 194 4.702 25.278 108.839 1.00 0.00

ATOM 3065 H ARG A 194 3.759 27.320 108.488 1.00 0.00

ATOM 3066 H ARG A 194 5.132 27.892 107.522 1.00 0.00

ATOM 3067 H ARG A 194 6.119 28.746 109.395 1.00 0.00

ATOM 3068 H ARG A 194 3.848 26.718 112.061 1.00 0.00

ATOM 3069 H ARG A 194 3.690 26.386 110.315 1.00 0.00

ATOM 3070 H ARG A 194 5.368 28.275 112.815 1.00 0.00

ATOM 3071 H ARG A 194 6.391 29.149 111.644 1.00 0.00

ATOM 3072 N GLN A 195 5.853 23.625 104.450 1.00 51.11

ATOM 3073 CA GLN A 195 5.347 23.079 103.220 1.00 50.68

ATOM 3074 C GLN A 195 5.436 21.547 103.219 1.00 50.70

ATOM 3075 O GLN A 195 4.463 20.834 102.876 1.00 50.83

ATOM 3076 CB GLN A 195 6.127 23.643 102.050 1.00 50.07

ATOM 3077 CG GLN A 195 5.406 23.409 100.741 1.00 52.23

ATOM 3078 CD GLN A 195 3.936 23.955 100.690 1.00 50.46

ATOM 3079 OE1 GLN A 195 3.505 24.764 101.513 1.00 55.84

ATOM 3080 NE2 GLN A 195 3.211 23.526 99.708 1.00 47.79

ATOM 3081 H GLN A 195 6.667 24.256 104.431 1.00 0.00

ATOM 3082 H GLN A 195 4.298 23.358 103.126 1.00 0.00

ATOM 3083 H GLN A 195 7.095 23.144 102.006 1.00 0.00

ATOM 3084 H GLN A 195 6.239 24.718 102.195 1.00 0.00

ATOM 3085 H GLN A 195 5.343 22.326 100.637 1.00 0.00

ATOM 3086 H GLN A 195 5.987 23.911 99.968 1.00 0.00

ATOM 3087 H GLN A 195 2.236 23.842 99.611 1.00 0.00

ATOM 3088 H GLN A 195 3.609 22.868 99.023 1.00 0.00

ATOM 3089 N ARG A 196 6.591 21.026 103.646 1.00 50.41

ATOM 3090 CA ARG A 196 6.755 19.592 103.667 1.00 50.14

ATOM 3091 C ARG A 196 5.628 18.898 104.475 1.00 49.29

ATOM 3092 O ARG A 196 5.227 17.838 104.153 1.00 48.59

ATOM 3093 CB ARG A 196 8.194 19.180 104.047 1.00 50.29

ATOM 3094 CG ARG A 196 8.468 17.711 103.956 1.00 50.56

ATOM 3095 CD ARG A 196 8.919 17.314 102.606 1.00 53.37

ATOM 3096 NE ARG A 196 8.920 15.864 102.327 1.00 55.81

ATOM 3097 CZ ARG A 196 7.816 15.161 102.144 1.00 54.15

ATOM 3098 NH1 ARG A 196 6.645 15.756 102.292 1.00 56.19

ATOM 3099 NH2 ARG A 196 7.877 13.892 101.847 1.00 49.26

ATOM 3100 H ARG A 196 7.356 21.642 103.957 1.00 0.00

ATOM 3101 H ARG A 196 6.632 19.215 102.652 1.00 0.00

ATOM 3102 H ARG A 196 8.371 19.472 105.082 1.00 0.00

ATOM 3103 H ARG A 196 8.860 19.649 103.322 1.00 0.00

ATOM 3104 H ARG A 196 7.550 17.169 104.181 1.00 0.00

ATOM 3105 H ARG A 196 9.268 17.470 104.656 1.00 0.00

ATOM 3106 H ARG A 196 9.954 17.644 102.518 1.00 0.00

ATOM 3107 H ARG A 196 8.215 17.761 101.904 1.00 0.00

ATOM 3108 H ARG A 196 9.826 15.376 102.272 1.00 0.00

ATOM 3109 H ARG A 196 5.775 15.222 102.153 1.00 0.00

ATOM 3110 H ARG A 196 6.603 16.753 102.546 1.00 0.00

ATOM 3111 H ARG A 196 7.010 13.354 101.707 1.00 0.00

ATOM 3112 H ARG A 196 8.792 13.428 101.753 1.00 0.00

ATOM 3113 N LEU A 197 5.070 19.534 105.473 1.00 49.91

ATOM 3114 CA LEU A 197 4.026 18.892 106.223 1.00 50.57

ATOM 3115 C LEU A 197 2.679 18.986 105.474 1.00 52.77

ATOM 3116 O LEU A 197 1.815 18.059 105.520 1.00 52.58

ATOM 3117 CB LEU A 197 3.976 19.562 107.580 1.00 50.80

ATOM 3118 CG LEU A 197 2.668 19.371 108.334 1.00 51.62

ATOM 3119 CD1 LEU A 197 2.521 17.902 108.754 1.00 48.82

ATOM 3120 CD2 LEU A 197 2.601 20.293 109.531 1.00 47.34

ATOM 3121 H LEU A 197 5.376 20.486 105.719 1.00 0.00

ATOM 3122 H LEU A 197 4.226 17.828 106.347 1.00 0.00

ATOM 3123 H LEU A 197 4.089 20.632 107.406 1.00 0.00

ATOM 3124 H LEU A 197 4.764 19.114 108.185 1.00 0.00

ATOM 3125 H LEU A 197 1.837 19.627 107.676 1.00 0.00

ATOM 3126 H LEU A 197 1.574 17.816 109.288 1.00 0.00

ATOM 3127 H LEU A 197 2.527 17.307 107.841 1.00 0.00

ATOM 3128 H LEU A 197 3.370 17.667 109.396 1.00 0.00

ATOM 3129 H LEU A 197 1.646 20.107 110.023 1.00 0.00

ATOM 3130 H LEU A 197 3.444 20.042 110.174 1.00 0.00

ATOM 3131 H LEU A 197 2.670 21.313 109.152 1.00 0.00

ATOM 3132 N ARG A 198 2.507 20.112 104.765 1.00 53.24

ATOM 3133 CA ARG A 198 1.332 20.288 103.926 1.00 53.38

ATOM 3134 C ARG A 198 1.241 19.166 102.914 1.00 52.86

ATOM 3135 O ARG A 198 0.198 18.500 102.823 1.00 52.91

ATOM 3136 CB ARG A 198 1.254 21.688 103.260 1.00 54.21

ATOM 3137 CG ARG A 198 0.843 22.720 104.246 1.00 51.92

ATOM 3138 CD ARG A 198 0.591 24.001 103.600 1.00 54.24

ATOM 3139 NE ARG A 198 1.778 24.838 103.379 1.00 55.53

ATOM 3140 CZ ARG A 198 2.126 25.877 104.139 1.00 55.08

ATOM 3141 NH1 ARG A 198 1.431 26.179 105.236 1.00 55.64

ATOM 3142 NH2 ARG A 198 3.215 26.562 103.838 1.00 55.29

ATOM 3143 H ARG A 198 3.214 20.859 104.815 1.00 0.00

ATOM 3144 H ARG A 198 0.459 20.238 104.577 1.00 0.00

ATOM 3145 H ARG A 198 0.537 21.683 102.439 1.00 0.00

ATOM 3146 H ARG A 198 2.248 21.963 102.906 1.00 0.00

ATOM 3147 H ARG A 198 1.637 22.853 104.981 1.00 0.00

ATOM 3148 H ARG A 198 -0.085 22.398 104.718 1.00 0.00

ATOM 3149 H ARG A 198 -0.063 24.558 104.271 1.00 0.00

ATOM 3150 H ARG A 198 0.171 23.778 102.619 1.00 0.00

ATOM 3151 H ARG A 198 2.385 24.606 102.580 1.00 0.00

ATOM 3152 H ARG A 198 1.708 26.984 105.816 1.00 0.00

ATOM 3153 H ARG A 198 0.617 25.608 105.503 1.00 0.00

ATOM 3154 H ARG A 198 3.494 27.366 104.418 1.00 0.00

ATOM 3155 H ARG A 198 3.785 26.292 103.024 1.00 0.00

ATOM 3156 N CYS A 199 2.330 18.921 102.188 1.00 53.67

ATOM 3157 CA CYS A 199 2.397 17.766 101.284 1.00 53.50

ATOM 3158 C CYS A 199 2.304 16.425 102.020 1.00 54.03

ATOM 3159 O CYS A 199 1.651 15.529 101.535 1.00 54.89

ATOM 3160 CB CYS A 199 3.674 17.766 100.503 1.00 53.69

ATOM 3161 SG CYS A 199 4.070 19.330 99.800 1.00 62.20

ATOM 3162 H CYS A 199 3.140 19.553 102.262 1.00 0.00

ATOM 3163 H CYS A 199 1.537 17.868 100.622 1.00 0.00

ATOM 3164 H CYS A 199 3.415 17.109 99.673 1.00 0.00

ATOM 3165 H CYS A 199 4.440 17.356 101.161 1.00 0.00

ATOM 3166 N ALA A 200 2.912 16.232 103.173 1.00 53.75

ATOM 3167 CA ALA A 200 2.669 14.917 103.802 1.00 55.79

ATOM 3168 C ALA A 200 1.182 14.705 104.109 1.00 55.75

ATOM 3169 O ALA A 200 0.672 13.632 103.859 1.00 56.65

ATOM 3170 CB ALA A 200 3.487 14.668 105.048 1.00 55.33

ATOM 3171 H ALA A 200 3.514 16.947 103.605 1.00 0.00

ATOM 3172 H ALA A 200 2.997 14.190 103.059 1.00 0.00

ATOM 3173 H ALA A 200 3.219 13.676 105.412 1.00 0.00

ATOM 3174 H ALA A 200 4.536 14.723 104.757 1.00 0.00

ATOM 3175 H ALA A 200 3.223 15.447 105.763 1.00 0.00

ATOM 3176 N SER A 201 0.503 15.717 104.628 1.00 54.56

ATOM 3177 CA SER A 201 -0.917 15.598 104.907 1.00 55.45

ATOM 3178 C SER A 201 -1.666 15.007 103.763 1.00 55.40

ATOM 3179 O SER A 201 -2.307 14.008 103.943 1.00 57.45

ATOM 3180 CB SER A 201 -1.537 16.950 105.242 1.00 55.90

ATOM 3181 OG SER A 201 -0.625 17.646 106.093 1.00 60.35

ATOM 3182 H SER A 201 0.989 16.601 104.837 1.00 0.00

ATOM 3183 H SER A 201 -0.997 14.933 105.767 1.00 0.00

ATOM 3184 H SER A 201 -2.512 16.865 105.722 1.00 0.00

ATOM 3185 H SER A 201 -1.666 17.541 104.335 1.00 0.00

ATOM 3186 H SER A 201 -1.053 18.557 106.313 1.00 0.00

ATOM 3187 N ILE A 202 -1.580 15.600 102.580 1.00 54.81

ATOM 3188 CA ILE A 202 -2.361 15.147 101.453 1.00 54.96

ATOM 3189 C ILE A 202 -1.885 13.809 100.999 1.00 54.49

ATOM 3190 O ILE A 202 -2.721 12.945 100.765 1.00 55.45

ATOM 3191 CB ILE A 202 -2.244 16.027 100.196 1.00 55.83

ATOM 3192 CG1 ILE A 202 -1.993 17.471 100.552 1.00 54.47

ATOM 3193 CG2 ILE A 202 -3.476 15.779 99.196 1.00 56.48

ATOM 3194 CD1 ILE A 202 -2.083 18.401 99.372 1.00 58.31

ATOM 3195 H ILE A 202 -0.943 16.401 102.463 1.00 0.00

ATOM 3196 H ILE A 202 -3.385 15.160 101.826 1.00 0.00

ATOM 3197 H ILE A 202 -1.360 15.725 99.634 1.00 0.00

ATOM 3198 H ILE A 202 -0.981 17.542 100.950 1.00 0.00

ATOM 3199 H ILE A 202 -2.760 17.772 101.265 1.00 0.00

ATOM 3200 H ILE A 202 -3.325 16.432 98.336 1.00 0.00

ATOM 3201 H ILE A 202 -3.451 14.726 98.915 1.00 0.00

ATOM 3202 H ILE A 202 -4.385 16.031 99.742 1.00 0.00

ATOM 3203 H ILE A 202 -1.886 19.407 99.743 1.00 0.00

ATOM 3204 H ILE A 202 -1.329 18.079 98.654 1.00 0.00

ATOM 3205 H ILE A 202 -3.091 18.308 98.969 1.00 0.00

ATOM 3206 N GLN A 203 -0.576 13.644 100.827 1.00 53.60

ATOM 3207 CA GLN A 203 -0.054 12.376 100.271 1.00 56.24

ATOM 3208 C GLN A 203 -0.365 11.142 101.146 1.00 58.10

ATOM 3209 O GLN A 203 -0.328 10.029 100.658 1.00 60.58

ATOM 3210 CB GLN A 203 1.432 12.412 99.989 1.00 55.04

ATOM 3211 CG GLN A 203 1.926 13.588 99.109 1.00 59.57

ATOM 3212 CD GLN A 203 3.462 13.811 99.241 1.00 64.62

ATOM 3213 OE1 GLN A 203 3.985 13.988 100.356 1.00 62.80

ATOM 3214 NE2 GLN A 203 4.181 13.779 98.100 1.00 64.53

ATOM 3215 H GLN A 203 0.075 14.400 101.081 1.00 0.00

ATOM 3216 H GLN A 203 -0.589 12.274 99.327 1.00 0.00

ATOM 3217 H GLN A 203 1.641 11.501 99.428 1.00 0.00

ATOM 3218 H GLN A 203 1.926 12.500 100.957 1.00 0.00

ATOM 3219 H GLN A 203 1.420 14.494 99.443 1.00 0.00

ATOM 3220 H GLN A 203 1.700 13.353 98.069 1.00 0.00

ATOM 3221 H GLN A 203 5.201 13.920 98.130 1.00 0.00

ATOM 3222 H GLN A 203 3.710 13.613 97.199 1.00 0.00

ATOM 3223 N LYS A 204 -0.719 11.342 102.402 1.00 57.71

ATOM 3224 CA LYS A 204 -0.620 10.297 103.374 1.00 60.43

ATOM 3225 C LYS A 204 -1.888 10.294 104.234 1.00 61.31

ATOM 3226 O LYS A 204 -2.207 9.304 104.874 1.00 63.46

ATOM 3227 CB LYS A 204 0.606 10.541 104.260 1.00 60.51

ATOM 3228 CG LYS A 204 1.809 9.628 104.091 1.00 64.61

ATOM 3229 CD LYS A 204 2.271 9.474 102.640 1.00 68.50

ATOM 3230 CE LYS A 204 3.781 9.347 102.551 1.00 69.24

ATOM 3231 NZ LYS A 204 4.366 8.282 103.398 1.00 71.44

ATOM 3232 H LYS A 204 -1.073 12.266 102.687 1.00 0.00

ATOM 3233 H LYS A 204 -0.515 9.334 102.875 1.00 0.00

ATOM 3234 H LYS A 204 0.266 10.381 105.283 1.00 0.00

ATOM 3235 H LYS A 204 0.966 11.533 103.985 1.00 0.00

ATOM 3236 H LYS A 204 1.512 8.640 104.443 1.00 0.00

ATOM 3237 H LYS A 204 2.632 10.068 104.654 1.00 0.00

ATOM 3238 H LYS A 204 1.964 10.355 102.077 1.00 0.00

ATOM 3239 H LYS A 204 1.828 8.566 102.230 1.00 0.00

ATOM 3240 H LYS A 204 4.217 10.289 102.882 1.00 0.00

ATOM 3241 H LYS A 204 4.015 9.071 101.523 1.00 0.00

ATOM 3242 H LYS A 204 5.385 8.302 103.246 1.00 0.00

ATOM 3243 H LYS A 204 4.120 8.504 104.373 1.00 0.00

ATOM 3244 H LYS A 204 3.951 7.391 103.091 1.00 0.00

ATOM 3245 N PHE A 205 -2.592 11.408 104.272 1.00 59.57

ATOM 3246 CA PHE A 205 -3.816 11.454 105.007 1.00 60.64

ATOM 3247 C PHE A 205 -4.988 12.012 104.226 1.00 60.63

ATOM 3248 O PHE A 205 -6.049 12.122 104.790 1.00 64.05

ATOM 3249 CB PHE A 205 -3.622 12.251 106.302 1.00 61.29

ATOM 3250 CG PHE A 205 -2.699 11.575 107.305 1.00 62.51

ATOM 3251 CD1 PHE A 205 -3.198 10.665 108.215 1.00 62.83

ATOM 3252 CD2 PHE A 205 -1.321 11.849 107.319 1.00 61.70

ATOM 3253 CE1 PHE A 205 -2.332 10.001 109.121 1.00 65.48

ATOM 3254 CE2 PHE A 205 -0.485 11.225 108.228 1.00 63.00

ATOM 3255 CZ PHE A 205 -1.003 10.296 109.144 1.00 62.34

ATOM 3256 H PHE A 205 -2.260 12.245 103.772 1.00 0.00

ATOM 3257 H PHE A 205 -4.070 10.418 105.231 1.00 0.00

ATOM 3258 H PHE A 205 -4.598 12.355 106.776 1.00 0.00

ATOM 3259 H PHE A 205 -3.161 13.201 106.033 1.00 0.00

ATOM 3260 H PHE A 205 -4.267 10.455 108.238 1.00 0.00

ATOM 3261 H PHE A 205 -0.906 12.561 106.606 1.00 0.00

ATOM 3262 H PHE A 205 -2.733 9.251 109.803 1.00 0.00

ATOM 3263 H PHE A 205 0.580 11.456 108.233 1.00 0.00

ATOM 3264 H PHE A 205 -0.346 9.814 109.868 1.00 0.00

ATOM 3265 N GLY A 206 -4.807 12.372 102.956 1.00 58.49

ATOM 3266 CA GLY A 206 -5.864 12.864 102.134 1.00 58.04

ATOM 3267 C GLY A 206 -5.957 14.390 102.022 1.00 58.65

ATOM 3268 O GLY A 206 -5.391 15.168 102.857 1.00 56.42

ATOM 3269 H GLY A 206 -3.864 12.292 102.550 1.00 0.00

ATOM 3270 H GLY A 206 -6.765 12.539 102.654 1.00 0.00

ATOM 3271 H GLY A 206 -5.711 12.431 101.145 1.00 0.00

ATOM 3272 N GLU A 207 -6.689 14.809 100.984 1.00 58.56

ATOM 3273 CA GLU A 207 -6.985 16.179 100.742 1.00 59.26

ATOM 3274 C GLU A 207 -7.782 16.659 101.944 1.00 60.29

ATOM 3275 O GLU A 207 -7.487 17.708 102.474 1.00 60.40

ATOM 3276 CB GLU A 207 -7.789 16.331 99.444 1.00 61.16

ATOM 3277 CG GLU A 207 -7.475 17.650 98.711 1.00 63.10

ATOM 3278 CD GLU A 207 -8.236 17.851 97.371 1.00 66.45

ATOM 3279 OE1 GLU A 207 -8.369 16.884 96.567 1.00 65.78

ATOM 3280 OE2 GLU A 207 -8.674 19.004 97.133 1.00 66.72

ATOM 3281 H GLU A 207 -7.056 14.105 100.328 1.00 0.00

ATOM 3282 H GLU A 207 -6.078 16.771 100.618 1.00 0.00

ATOM 3283 H GLU A 207 -8.847 16.333 99.705 1.00 0.00

ATOM 3284 H GLU A 207 -7.526 15.504 98.785 1.00 0.00

ATOM 3285 H GLU A 207 -6.412 17.624 98.470 1.00 0.00

ATOM 3286 H GLU A 207 -7.775 18.460 99.376 1.00 0.00

ATOM 3287 N ARG A 208 -8.768 15.885 102.395 1.00 61.40

ATOM 3288 CA ARG A 208 -9.538 16.207 103.605 1.00 62.23

ATOM 3289 C ARG A 208 -8.692 16.731 104.760 1.00 61.01

ATOM 3290 O ARG A 208 -9.205 17.433 105.624 1.00 62.78

ATOM 3291 CB ARG A 208 -10.248 14.966 104.126 1.00 64.06

ATOM 3292 CG ARG A 208 -11.173 15.285 105.290 1.00 69.51

ATOM 3293 CD ARG A 208 -12.070 14.075 105.692 1.00 71.77

ATOM 3294 NE ARG A 208 -11.513 13.250 106.770 1.00 68.52

ATOM 3295 CZ ARG A 208 -11.957 13.263 108.019 1.00 72.06

ATOM 3296 NH1 ARG A 208 -12.943 14.064 108.340 1.00 74.72

ATOM 3297 NH2 ARG A 208 -11.428 12.472 108.953 1.00 74.51

ATOM 3298 H ARG A 208 -9.000 15.027 101.875 1.00 0.00

ATOM 3299 H ARG A 208 -10.230 16.988 103.292 1.00 0.00

ATOM 3300 H ARG A 208 -9.493 14.262 104.474 1.00 0.00

ATOM 3301 H ARG A 208 -10.850 14.553 103.317 1.00 0.00

ATOM 3302 H ARG A 208 -11.827 16.093 104.962 1.00 0.00

ATOM 3303 H ARG A 208 -10.554 15.566 106.142 1.00 0.00

ATOM 3304 H ARG A 208 -12.218 13.422 104.832 1.00 0.00

ATOM 3305 H ARG A 208 -12.993 14.483 106.104 1.00 0.00

ATOM 3306 H ARG A 208 -10.729 12.622 106.542 1.00 0.00

ATOM 3307 H ARG A 208 -13.297 14.083 109.307 1.00 0.00

ATOM 3308 H ARG A 208 -13.364 14.674 107.625 1.00 0.00

ATOM 3309 H ARG A 208 -11.790 12.500 109.917 1.00 0.00

ATOM 3310 H ARG A 208 -10.657 11.833 108.711 1.00 0.00

ATOM 3311 N ALA A 209 -7.415 16.376 104.825 1.00 59.00

ATOM 3312 CA ALA A 209 -6.665 16.712 106.028 1.00 58.28

ATOM 3313 C ALA A 209 -6.036 18.117 105.878 1.00 57.13

ATOM 3314 O ALA A 209 -5.972 18.931 106.808 1.00 56.00

ATOM 3315 CB ALA A 209 -5.638 15.665 106.296 1.00 57.99

ATOM 3316 H ALA A 209 -6.966 15.876 104.045 1.00 0.00

ATOM 3317 H ALA A 209 -7.336 16.740 106.886 1.00 0.00

ATOM 3318 H ALA A 209 -5.108 15.963 107.201 1.00 0.00

ATOM 3319 H ALA A 209 -6.170 14.723 106.429 1.00 0.00

ATOM 3320 H ALA A 209 -4.977 15.638 105.430 1.00 0.00

ATOM 3321 N LEU A 210 -5.611 18.397 104.658 1.00 56.66

ATOM 3322 CA LEU A 210 -5.035 19.648 104.348 1.00 56.33

ATOM 3323 C LEU A 210 -6.117 20.719 104.420 1.00 58.02

ATOM 3324 O LEU A 210 -5.990 21.661 105.199 1.00 58.59

ATOM 3325 CB LEU A 210 -4.440 19.545 102.961 1.00 54.59

ATOM 3326 CG LEU A 210 -3.890 20.899 102.525 1.00 56.36

ATOM 3327 CD1 LEU A 210 -2.608 21.371 103.336 1.00 54.69

ATOM 3328 CD2 LEU A 210 -3.570 20.737 101.056 1.00 57.82

ATOM 3329 H LEU A 210 -5.702 17.687 103.917 1.00 0.00

ATOM 3330 H LEU A 210 -4.249 19.921 105.053 1.00 0.00

ATOM 3331 H LEU A 210 -5.220 19.235 102.266 1.00 0.00

ATOM 3332 H LEU A 210 -3.634 18.811 102.971 1.00 0.00

ATOM 3333 H LEU A 210 -4.626 21.679 102.721 1.00 0.00

ATOM 3334 H LEU A 210 -2.316 22.341 102.934 1.00 0.00

ATOM 3335 H LEU A 210 -2.901 21.438 104.384 1.00 0.00

ATOM 3336 H LEU A 210 -1.836 20.618 103.178 1.00 0.00

ATOM 3337 H LEU A 210 -3.173 21.691 100.709 1.00 0.00

ATOM 3338 H LEU A 210 -2.833 19.938 100.977 1.00 0.00

ATOM 3339 H LEU A 210 -4.502 20.480 100.553 1.00 0.00

ATOM 3340 N LYS A 211 -7.158 20.554 103.581 1.00 58.87

ATOM 3341 CA LYS A 211 -8.369 21.314 103.627 1.00 59.95

ATOM 3342 C LYS A 211 -8.682 21.624 105.078 1.00 60.94

ATOM 3343 O LYS A 211 -8.835 22.799 105.418 1.00 61.31

ATOM 3344 CB LYS A 211 -9.484 20.495 103.010 1.00 62.10

ATOM 3345 CG LYS A 211 -10.615 21.276 102.475 1.00 63.94

ATOM 3346 CD LYS A 211 -11.663 20.355 101.832 1.00 66.17

ATOM 3347 CE LYS A 211 -13.056 21.068 101.790 1.00 72.84

ATOM 3348 NZ LYS A 211 -14.144 20.366 101.004 1.00 74.73

ATOM 3349 H LYS A 211 -7.076 19.829 102.854 1.00 0.00

ATOM 3350 H LYS A 211 -8.266 22.246 103.071 1.00 0.00

ATOM 3351 H LYS A 211 -9.891 19.847 103.786 1.00 0.00

ATOM 3352 H LYS A 211 -9.054 19.978 102.152 1.00 0.00

ATOM 3353 H LYS A 211 -10.213 21.937 101.707 1.00 0.00

ATOM 3354 H LYS A 211 -11.060 21.849 103.289 1.00 0.00

ATOM 3355 H LYS A 211 -11.743 19.452 102.437 1.00 0.00

ATOM 3356 H LYS A 211 -11.344 20.104 100.820 1.00 0.00

ATOM 3357 H LYS A 211 -12.916 22.039 101.314 1.00 0.00

ATOM 3358 H LYS A 211 -13.420 21.083 102.817 1.00 0.00

ATOM 3359 H LYS A 211 -14.989 20.952 101.070 1.00 0.00

ATOM 3360 H LYS A 211 -13.809 20.285 100.033 1.00 0.00

ATOM 3361 H LYS A 211 -14.283 19.443 101.440 1.00 0.00

ATOM 3362 N ALA A 212 -8.720 20.608 105.950 1.00 60.73

ATOM 3363 CA ALA A 212 -9.145 20.860 107.322 1.00 61.92

ATOM 3364 C ALA A 212 -8.163 21.774 108.034 1.00 61.71

ATOM 3365 O ALA A 212 -8.520 22.598 108.891 1.00 62.51

ATOM 3366 CB ALA A 212 -9.284 19.579 108.071 1.00 63.24

ATOM 3367 H ALA A 212 -8.452 19.658 105.655 1.00 0.00

ATOM 3368 H ALA A 212 -10.116 21.355 107.287 1.00 0.00

ATOM 3369 H ALA A 212 -9.602 19.834 109.082 1.00 0.00

ATOM 3370 H ALA A 212 -10.033 18.984 107.549 1.00 0.00

ATOM 3371 H ALA A 212 -8.306 19.097 108.063 1.00 0.00

ATOM 3372 N TRP A 213 -6.918 21.645 107.651 1.00 60.30

ATOM 3373 CA TRP A 213 -5.935 22.531 108.140 1.00 60.65

ATOM 3374 C TRP A 213 -6.217 23.982 107.806 1.00 60.80

ATOM 3375 O TRP A 213 -6.152 24.872 108.686 1.00 61.65

ATOM 3376 CB TRP A 213 -4.644 22.132 107.537 1.00 59.24

ATOM 3377 CG TRP A 213 -3.615 23.022 107.822 1.00 60.08

ATOM 3378 CD1 TRP A 213 -2.763 22.980 108.884 1.00 61.11

ATOM 3379 CD2 TRP A 213 -3.214 24.128 107.001 1.00 63.50

ATOM 3380 NE1 TRP A 213 -1.833 24.023 108.781 1.00 60.84

ATOM 3381 CE2 TRP A 213 -2.102 24.736 107.630 1.00 62.57

ATOM 3382 CE3 TRP A 213 -3.694 24.669 105.780 1.00 64.39

ATOM 3383 CZ2 TRP A 213 -1.465 25.866 107.083 1.00 63.93

ATOM 3384 CZ3 TRP A 213 -3.080 25.806 105.253 1.00 62.53

ATOM 3385 CH2 TRP A 213 -1.971 26.386 105.905 1.00 63.61

ATOM 3386 H TRP A 213 -6.656 20.897 106.993 1.00 0.00

ATOM 3387 H TRP A 213 -5.923 22.464 109.228 1.00 0.00

ATOM 3388 H TRP A 213 -4.734 22.068 106.453 1.00 0.00

ATOM 3389 H TRP A 213 -4.343 21.190 107.995 1.00 0.00

ATOM 3390 H TRP A 213 -2.799 22.247 109.690 1.00 0.00

ATOM 3391 H TRP A 213 -1.077 24.224 109.451 1.00 0.00

ATOM 3392 H TRP A 213 -4.531 24.203 105.261 1.00 0.00

ATOM 3393 H TRP A 213 -0.601 26.317 107.572 1.00 0.00

ATOM 3394 H TRP A 213 -3.459 26.249 104.332 1.00 0.00

ATOM 3395 H TRP A 213 -1.500 27.267 105.469 1.00 0.00

ATOM 3396 N SER A 214 -6.505 24.247 106.537 1.00 60.64

ATOM 3397 CA SER A 214 -6.817 25.614 106.129 1.00 59.90

ATOM 3398 C SER A 214 -8.074 26.187 106.840 1.00 60.87

ATOM 3399 O SER A 214 -8.097 27.383 107.108 1.00 61.27

ATOM 3400 CB SER A 214 -6.946 25.770 104.627 1.00 58.88

ATOM 3401 OG SER A 214 -5.997 25.014 103.927 1.00 60.20

ATOM 3402 H SER A 214 -6.508 23.488 105.840 1.00 0.00

ATOM 3403 H SER A 214 -5.957 26.201 106.450 1.00 0.00

ATOM 3404 H SER A 214 -6.832 26.811 104.324 1.00 0.00

ATOM 3405 H SER A 214 -7.909 25.353 104.330 1.00 0.00

ATOM 3406 H SER A 214 -6.160 25.181 102.924 1.00 0.00

ATOM 3407 N VAL A 215 -9.105 25.385 107.142 1.00 61.51

ATOM 3408 CA VAL A 215 -10.262 25.940 107.914 1.00 61.69

ATOM 3409 C VAL A 215 -9.724 26.500 109.255 1.00 62.01

ATOM 3410 O VAL A 215 -9.910 27.688 109.618 1.00 61.37

ATOM 3411 CB VAL A 215 -11.371 24.911 108.208 1.00 63.76

ATOM 3412 CG1 VAL A 215 -12.678 25.642 108.722 1.00 64.64

ATOM 3413 CG2 VAL A 215 -11.643 23.969 107.003 1.00 62.28

ATOM 3414 H VAL A 215 -9.101 24.398 106.846 1.00 0.00

ATOM 3415 H VAL A 215 -10.719 26.713 107.296 1.00 0.00

ATOM 3416 H VAL A 215 -11.021 24.259 109.008 1.00 0.00

ATOM 3417 H VAL A 215 -13.422 24.868 108.912 1.00 0.00

ATOM 3418 H VAL A 215 -12.407 26.176 109.633 1.00 0.00

ATOM 3419 H VAL A 215 -12.994 26.323 107.932 1.00 0.00

ATOM 3420 H VAL A 215 -12.435 23.284 107.306 1.00 0.00

ATOM 3421 H VAL A 215 -11.950 24.600 106.168 1.00 0.00

ATOM 3422 H VAL A 215 -10.712 23.443 106.793 1.00 0.00

ATOM 3423 N ALA A 216 -9.022 25.640 109.991 1.00 61.73

ATOM 3424 CA ALA A 216 -8.344 26.086 111.199 1.00 61.68

ATOM 3425 C ALA A 216 -7.515 27.353 110.915 1.00 61.14

ATOM 3426 O ALA A 216 -7.723 28.376 111.534 1.00 61.30

ATOM 3427 CB ALA A 216 -7.481 24.984 111.765 1.00 60.12

ATOM 3428 H ALA A 216 -8.959 24.653 109.703 1.00 0.00

ATOM 3429 H ALA A 216 -9.097 26.336 111.947 1.00 0.00

ATOM 3430 H ALA A 216 -7.005 25.381 112.662 1.00 0.00

ATOM 3431 H ALA A 216 -8.141 24.147 111.993 1.00 0.00

ATOM 3432 H ALA A 216 -6.750 24.726 110.999 1.00 0.00

ATOM 3433 N ARG A 217 -6.604 27.307 109.961 1.00 60.30

ATOM 3434 CA ARG A 217 -5.693 28.439 109.871 1.00 60.18

ATOM 3435 C ARG A 217 -6.480 29.687 109.510 1.00 60.44

ATOM 3436 O ARG A 217 -6.315 30.719 110.141 1.00 60.93

ATOM 3437 CB ARG A 217 -4.501 28.220 108.897 1.00 59.09

ATOM 3438 CG ARG A 217 -3.449 29.336 109.042 1.00 60.28

ATOM 3439 CD ARG A 217 -2.241 29.183 108.136 1.00 61.99

ATOM 3440 NE ARG A 217 -1.226 30.266 108.271 1.00 64.21

ATOM 3441 CZ ARG A 217 -0.549 30.596 109.376 1.00 63.76

ATOM 3442 NH1 ARG A 217 -0.774 30.020 110.539 1.00 64.12

ATOM 3443 NH2 ARG A 217 0.347 31.564 109.324 1.00 67.69

ATOM 3444 H ARG A 217 -6.541 26.510 109.312 1.00 0.00

ATOM 3445 H ARG A 217 -5.232 28.557 110.852 1.00 0.00

ATOM 3446 H ARG A 217 -4.875 28.226 107.873 1.00 0.00

ATOM 3447 H ARG A 217 -4.025 27.270 109.139 1.00 0.00

ATOM 3448 H ARG A 217 -3.083 29.314 110.068 1.00 0.00

ATOM 3449 H ARG A 217 -3.933 30.270 108.755 1.00 0.00

ATOM 3450 H ARG A 217 -2.593 29.203 107.105 1.00 0.00

ATOM 3451 H ARG A 217 -1.741 28.259 108.425 1.00 0.00

ATOM 3452 H ARG A 217 -1.025 30.818 107.425 1.00 0.00

ATOM 3453 H ARG A 217 -0.231 30.303 111.367 1.00 0.00

ATOM 3454 H ARG A 217 -1.493 29.287 110.620 1.00 0.00

ATOM 3455 H ARG A 217 0.873 31.825 110.170 1.00 0.00

ATOM 3456 H ARG A 217 0.520 32.058 108.437 1.00 0.00

ATOM 3457 N LEU A 218 -7.332 29.571 108.497 1.00 60.28

ATOM 3458 CA LEU A 218 -8.090 30.698 107.985 1.00 60.60

ATOM 3459 C LEU A 218 -9.035 31.268 109.040 1.00 61.13

ATOM 3460 O LEU A 218 -8.859 32.433 109.428 1.00 62.50

ATOM 3461 CB LEU A 218 -8.828 30.330 106.701 1.00 60.43

ATOM 3462 CG LEU A 218 -8.022 30.134 105.394 1.00 63.29

ATOM 3463 CD1 LEU A 218 -8.971 29.841 104.212 1.00 63.45

ATOM 3464 CD2 LEU A 218 -7.152 31.337 105.041 1.00 59.76

ATOM 3465 H LEU A 218 -7.459 28.647 108.059 1.00 0.00

ATOM 3466 H LEU A 218 -7.380 31.487 107.737 1.00 0.00

ATOM 3467 H LEU A 218 -9.469 31.189 106.506 1.00 0.00

ATOM 3468 H LEU A 218 -9.283 29.362 106.909 1.00 0.00

ATOM 3469 H LEU A 218 -7.361 29.286 105.572 1.00 0.00

ATOM 3470 H LEU A 218 -8.348 29.714 103.327 1.00 0.00

ATOM 3471 H LEU A 218 -9.515 28.930 104.459 1.00 0.00

ATOM 3472 H LEU A 218 -9.637 30.698 104.118 1.00 0.00

ATOM 3473 H LEU A 218 -6.634 31.094 104.113 1.00 0.00

ATOM 3474 H LEU A 218 -7.821 32.189 104.921 1.00 0.00

ATOM 3475 H LEU A 218 -6.457 31.481 105.868 1.00 0.00

ATOM 3476 N SER A 219 -9.999 30.474 109.513 1.00 60.30

ATOM 3477 CA SER A 219 -10.978 30.930 110.523 1.00 61.20

ATOM 3478 C SER A 219 -10.347 31.742 111.643 1.00 61.58

ATOM 3479 O SER A 219 -10.872 32.819 112.023 1.00 62.68

ATOM 3480 CB SER A 219 -11.745 29.770 111.132 1.00 61.10

ATOM 3481 OG SER A 219 -12.305 29.026 110.087 1.00 61.35

ATOM 3482 H SER A 219 -10.063 29.508 109.161 1.00 0.00

ATOM 3483 H SER A 219 -11.665 31.577 109.977 1.00 0.00

ATOM 3484 H SER A 219 -12.524 30.081 111.828 1.00 0.00

ATOM 3485 H SER A 219 -11.061 29.109 111.665 1.00 0.00

ATOM 3486 H SER A 219 -12.821 28.245 110.516 1.00 0.00

ATOM 3487 N GLN A 220 -9.222 31.230 112.149 1.00 60.52

ATOM 3488 CA GLN A 220 -8.392 31.923 113.153 1.00 60.85

ATOM 3489 C GLN A 220 -7.904 33.308 112.723 1.00 60.20

ATOM 3490 O GLN A 220 -7.973 34.229 113.509 1.00 61.77

ATOM 3491 CB GLN A 220 -7.200 31.057 113.504 1.00 60.14

ATOM 3492 CG GLN A 220 -7.046 30.639 114.969 1.00 62.86

ATOM 3493 CD GLN A 220 -5.626 30.056 115.245 1.00 64.70

ATOM 3494 OE1 GLN A 220 -4.834 29.783 114.308 1.00 63.98

ATOM 3495 NE2 GLN A 220 -5.316 29.860 116.512 1.00 63.84

ATOM 3496 H GLN A 220 -8.918 30.302 111.821 1.00 0.00

ATOM 3497 H GLN A 220 -9.036 32.084 114.018 1.00 0.00

ATOM 3498 H GLN A 220 -6.325 31.666 113.277 1.00 0.00

ATOM 3499 H GLN A 220 -7.326 30.135 112.936 1.00 0.00

ATOM 3500 H GLN A 220 -7.785 29.865 115.174 1.00 0.00

ATOM 3501 H GLN A 220 -7.205 31.515 115.598 1.00 0.00

ATOM 3502 H GLN A 220 -4.393 29.477 116.763 1.00 0.00

ATOM 3503 H GLN A 220 -5.997 30.090 117.250 1.00 0.00

ATOM 3504 N LYS A 221 -7.421 33.477 111.495 1.00 58.79

ATOM 3505 CA LYS A 221 -6.969 34.785 111.064 1.00 59.16

ATOM 3506 C LYS A 221 -8.157 35.683 110.746 1.00 61.15

ATOM 3507 O LYS A 221 -8.068 36.894 110.864 1.00 63.49

ATOM 3508 CB LYS A 221 -6.245 34.719 109.740 1.00 58.21

ATOM 3509 CG LYS A 221 -5.053 33.892 109.585 1.00 58.88

ATOM 3510 CD LYS A 221 -4.468 34.159 108.139 1.00 63.72

ATOM 3511 CE LYS A 221 -2.897 33.980 108.067 1.00 61.45

ATOM 3512 NZ LYS A 221 -2.168 34.853 109.099 1.00 61.89

ATOM 3513 H LYS A 221 -7.368 32.677 110.849 1.00 0.00

ATOM 3514 H LYS A 221 -6.339 35.152 111.874 1.00 0.00

ATOM 3515 H LYS A 221 -5.894 35.732 109.544 1.00 0.00

ATOM 3516 H LYS A 221 -6.958 34.239 109.070 1.00 0.00

ATOM 3517 H LYS A 221 -5.367 32.852 109.678 1.00 0.00

ATOM 3518 H LYS A 221 -4.346 34.097 110.389 1.00 0.00

ATOM 3519 H LYS A 221 -4.698 35.191 107.872 1.00 0.00

ATOM 3520 H LYS A 221 -4.917 33.435 107.459 1.00 0.00

ATOM 3521 H LYS A 221 -2.545 34.262 107.075 1.00 0.00

ATOM 3522 H LYS A 221 -2.662 32.943 108.306 1.00 0.00

ATOM 3523 H LYS A 221 -1.160 34.677 108.984 1.00 0.00

ATOM 3524 H LYS A 221 -2.412 35.832 108.891 1.00 0.00

ATOM 3525 H LYS A 221 -2.504 34.564 110.029 1.00 0.00

ATOM 3526 N PHE A 222 -9.235 35.113 110.219 1.00 60.63

ATOM 3527 CA PHE A 222 -10.271 35.914 109.596 1.00 60.82

ATOM 3528 C PHE A 222 -11.599 35.587 110.248 1.00 62.93

ATOM 3529 O PHE A 222 -12.566 35.224 109.559 1.00 63.26

ATOM 3530 CB PHE A 222 -10.364 35.533 108.144 1.00 60.11

ATOM 3531 CG PHE A 222 -9.127 35.835 107.328 1.00 60.56

ATOM 3532 CD1 PHE A 222 -8.318 36.905 107.608 1.00 60.76

ATOM 3533 CD2 PHE A 222 -8.815 35.058 106.223 1.00 61.99

ATOM 3534 CE1 PHE A 222 -7.221 37.177 106.830 1.00 60.73

ATOM 3535 CE2 PHE A 222 -7.714 35.361 105.421 1.00 59.08

ATOM 3536 CZ PHE A 222 -6.928 36.406 105.744 1.00 60.47

ATOM 3537 H PHE A 222 -9.336 34.089 110.253 1.00 0.00

ATOM 3538 H PHE A 222 -10.039 36.973 109.705 1.00 0.00

ATOM 3539 H PHE A 222 -11.176 36.123 107.718 1.00 0.00

ATOM 3540 H PHE A 222 -10.515 34.454 108.109 1.00 0.00

ATOM 3541 H PHE A 222 -8.548 37.547 108.458 1.00 0.00

ATOM 3542 H PHE A 222 -9.438 34.198 105.977 1.00 0.00

ATOM 3543 H PHE A 222 -6.576 38.019 107.083 1.00 0.00

ATOM 3544 H PHE A 222 -7.492 34.757 104.541 1.00 0.00

ATOM 3545 H PHE A 222 -6.054 36.636 105.135 1.00 0.00

ATOM 3546 N PRO A 223 -11.658 35.686 111.587 1.00 63.62

ATOM 3547 CA PRO A 223 -12.791 35.284 112.439 1.00 65.47

ATOM 3548 C PRO A 223 -14.111 35.965 112.135 1.00 67.64

ATOM 3549 O PRO A 223 -15.135 35.555 112.667 1.00 68.96

ATOM 3550 CB PRO A 223 -12.361 35.735 113.834 1.00 65.36

ATOM 3551 CG PRO A 223 -11.371 36.804 113.580 1.00 66.11

ATOM 3552 CD PRO A 223 -10.618 36.349 112.376 1.00 63.39

ATOM 3553 H PRO A 223 -12.979 34.220 112.298 1.00 0.00

ATOM 3554 H PRO A 223 -11.949 34.930 114.442 1.00 0.00

ATOM 3555 H PRO A 223 -13.199 36.111 114.421 1.00 0.00

ATOM 3556 H PRO A 223 -10.714 36.952 114.437 1.00 0.00

ATOM 3557 H PRO A 223 -11.849 37.771 113.424 1.00 0.00

ATOM 3558 H PRO A 223 -10.143 37.167 111.834 1.00 0.00

ATOM 3559 H PRO A 223 -9.774 35.698 112.605 1.00 0.00

ATOM 3560 N LYS A 224 -14.069 37.009 111.330 1.00 68.53

ATOM 3561 CA LYS A 224 -15.245 37.760 110.983 1.00 72.49

ATOM 3562 C LYS A 224 -15.658 37.441 109.550 1.00 73.26

ATOM 3563 O LYS A 224 -16.736 37.855 109.117 1.00 76.14

ATOM 3564 CB LYS A 224 -15.058 39.264 111.255 1.00 74.17

ATOM 3565 CG LYS A 224 -15.273 40.178 110.058 1.00 78.13

ATOM 3566 CD LYS A 224 -14.983 41.652 110.398 1.00 80.59

ATOM 3567 CE LYS A 224 -14.773 42.418 109.107 1.00 82.11

ATOM 3568 NZ LYS A 224 -13.948 43.659 109.313 1.00 82.88

ATOM 3569 H LYS A 224 -13.162 37.296 110.936 1.00 0.00

ATOM 3570 H LYS A 224 -16.071 37.457 111.627 1.00 0.00

ATOM 3571 H LYS A 224 -14.025 39.406 111.572 1.00 0.00

ATOM 3572 H LYS A 224 -15.816 39.543 111.987 1.00 0.00

ATOM 3573 H LYS A 224 -16.319 40.096 109.762 1.00 0.00

ATOM 3574 H LYS A 224 -14.597 39.863 109.263 1.00 0.00

ATOM 3575 H LYS A 224 -14.094 41.731 111.023 1.00 0.00

ATOM 3576 H LYS A 224 -15.829 42.081 110.935 1.00 0.00

ATOM 3577 H LYS A 224 -15.747 42.718 108.721 1.00 0.00

ATOM 3578 H LYS A 224 -14.237 41.772 108.412 1.00 0.00

ATOM 3579 H LYS A 224 -13.855 44.115 108.394 1.00 0.00

ATOM 3580 H LYS A 224 -14.457 44.251 109.984 1.00 0.00

ATOM 3581 H LYS A 224 -13.036 43.356 109.684 1.00 0.00

ATOM 3582 N ALA A 225 -14.828 36.678 108.841 1.00 71.10

ATOM 3583 CA ALA A 225 -15.262 36.055 107.614 1.00 71.25

ATOM 3584 C ALA A 225 -16.358 35.065 108.018 1.00 72.93

ATOM 3585 O ALA A 225 -16.188 34.289 108.978 1.00 71.16

ATOM 3586 CB ALA A 225 -14.084 35.315 106.941 1.00 67.90

ATOM 3587 H ALA A 225 -13.864 36.531 109.173 1.00 0.00

ATOM 3588 H ALA A 225 -15.629 36.789 106.896 1.00 0.00

ATOM 3589 H ALA A 225 -14.470 34.868 106.025 1.00 0.00

ATOM 3590 H ALA A 225 -13.315 36.060 106.735 1.00 0.00

ATOM 3591 H ALA A 225 -13.738 34.560 107.646 1.00 0.00

ATOM 3592 N GLU A 226 -17.488 35.084 107.313 1.00 75.96

ATOM 3593 CA GLU A 226 -18.551 34.095 107.617 1.00 78.55

ATOM 3594 C GLU A 226 -18.162 32.758 106.988 1.00 76.65

ATOM 3595 O GLU A 226 -17.186 32.688 106.271 1.00 74.61

ATOM 3596 CB GLU A 226 -19.927 34.614 107.171 1.00 81.79

ATOM 3597 CG GLU A 226 -21.191 33.911 107.738 1.00 87.96

ATOM 3598 CD GLU A 226 -21.242 33.696 109.280 1.00 91.10

ATOM 3599 OE1 GLU A 226 -21.714 34.625 110.012 1.00 93.53

ATOM 3600 OE2 GLU A 226 -20.872 32.568 109.726 1.00 85.57

ATOM 3601 H GLU A 226 -17.622 35.778 106.564 1.00 0.00

ATOM 3602 H GLU A 226 -18.641 33.942 108.692 1.00 0.00

ATOM 3603 H GLU A 226 -19.957 34.439 106.095 1.00 0.00

ATOM 3604 H GLU A 226 -19.976 35.644 107.524 1.00 0.00

ATOM 3605 H GLU A 226 -21.201 32.915 107.296 1.00 0.00

ATOM 3606 H GLU A 226 -22.030 34.563 107.496 1.00 0.00

ATOM 3607 N PHE A 227 -18.904 31.695 107.254 1.00 77.92

ATOM 3608 CA PHE A 227 -18.442 30.373 106.859 1.00 76.14

ATOM 3609 C PHE A 227 -18.333 30.150 105.355 1.00 76.43

ATOM 3610 O PHE A 227 -17.462 29.413 104.935 1.00 73.56

ATOM 3611 CB PHE A 227 -19.276 29.233 107.520 1.00 77.90

ATOM 3612 CG PHE A 227 -18.708 27.819 107.263 1.00 73.97

ATOM 3613 CD1 PHE A 227 -17.410 27.472 107.688 1.00 70.67

ATOM 3614 CD2 PHE A 227 -19.458 26.877 106.600 1.00 71.51

ATOM 3615 CE1 PHE A 227 -16.866 26.163 107.434 1.00 68.99

ATOM 3616 CE2 PHE A 227 -18.967 25.615 106.343 1.00 73.45

ATOM 3617 CZ PHE A 227 -17.669 25.236 106.750 1.00 70.55

ATOM 3618 H PHE A 227 -19.806 31.803 107.739 1.00 0.00

ATOM 3619 H PHE A 227 -17.421 30.332 107.240 1.00 0.00

ATOM 3620 H PHE A 227 -20.279 29.264 107.094 1.00 0.00

ATOM 3621 H PHE A 227 -19.253 29.395 108.598 1.00 0.00

ATOM 3622 H PHE A 227 -16.806 28.208 108.219 1.00 0.00

ATOM 3623 H PHE A 227 -20.464 27.134 106.270 1.00 0.00

ATOM 3624 H PHE A 227 -15.861 25.900 107.763 1.00 0.00

ATOM 3625 H PHE A 227 -19.593 24.895 105.815 1.00 0.00

ATOM 3626 H PHE A 227 -17.295 24.235 106.536 1.00 0.00

ATOM 3627 N VAL A 228 -19.240 30.732 104.556 1.00 79.31

ATOM 3628 CA VAL A 228 -19.236 30.472 103.102 1.00 80.15

ATOM 3629 C VAL A 228 -18.021 31.097 102.433 1.00 77.81

ATOM 3630 O VAL A 228 -17.423 30.510 101.518 1.00 77.07

ATOM 3631 CB VAL A 228 -20.520 30.935 102.361 1.00 83.82

ATOM 3632 CG1 VAL A 228 -20.557 30.346 100.940 1.00 84.92

ATOM 3633 CG2 VAL A 228 -21.756 30.475 103.086 1.00 87.75

ATOM 3634 H VAL A 228 -19.945 31.365 104.960 1.00 0.00

ATOM 3635 H VAL A 228 -19.198 29.386 103.019 1.00 0.00

ATOM 3636 H VAL A 228 -20.499 32.024 102.322 1.00 0.00

ATOM 3637 H VAL A 228 -21.474 30.703 100.471 1.00 0.00

ATOM 3638 H VAL A 228 -19.669 30.708 100.422 1.00 0.00

ATOM 3639 H VAL A 228 -20.551 29.261 101.044 1.00 0.00

ATOM 3640 H VAL A 228 -22.612 30.833 102.514 1.00 0.00

ATOM 3641 H VAL A 228 -21.718 29.386 103.121 1.00 0.00

ATOM 3642 H VAL A 228 -21.723 30.914 104.083 1.00 0.00

ATOM 3643 N GLU A 229 -17.661 32.289 102.892 1.00 76.57

ATOM 3644 CA GLU A 229 -16.456 32.925 102.424 1.00 74.39

ATOM 3645 C GLU A 229 -15.260 32.085 102.839 1.00 71.26

ATOM 3646 O GLU A 229 -14.375 31.793 102.028 1.00 69.47

ATOM 3647 CB GLU A 229 -16.379 34.321 103.014 1.00 76.25

ATOM 3648 CG GLU A 229 -15.165 35.144 102.626 1.00 76.28

ATOM 3649 CD GLU A 229 -15.336 35.801 101.294 1.00 81.46

ATOM 3650 OE1 GLU A 229 -16.466 36.234 100.937 1.00 85.65

ATOM 3651 OE2 GLU A 229 -14.317 35.889 100.596 1.00 84.08

ATOM 3652 H GLU A 229 -18.250 32.765 103.591 1.00 0.00

ATOM 3653 H GLU A 229 -16.458 33.007 101.337 1.00 0.00

ATOM 3654 H GLU A 229 -16.328 34.193 104.095 1.00 0.00

ATOM 3655 H GLU A 229 -17.247 34.861 102.635 1.00 0.00

ATOM 3656 H GLU A 229 -14.297 34.487 102.573 1.00 0.00

ATOM 3657 H GLU A 229 -15.036 35.932 103.368 1.00 0.00

ATOM 3658 N VAL A 230 -15.230 31.668 104.099 1.00 70.38

ATOM 3659 CA VAL A 230 -14.115 30.836 104.541 1.00 68.14

ATOM 3660 C VAL A 230 -14.017 29.592 103.664 1.00 67.49

ATOM 3661 O VAL A 230 -12.947 29.301 103.121 1.00 66.82

ATOM 3662 CB VAL A 230 -14.157 30.452 106.048 1.00 68.09

ATOM 3663 CG1 VAL A 230 -12.966 29.507 106.391 1.00 65.32

ATOM 3664 CG2 VAL A 230 -14.099 31.710 106.920 1.00 66.93

ATOM 3665 H VAL A 230 -15.981 31.927 104.754 1.00 0.00

ATOM 3666 H VAL A 230 -13.218 31.444 104.429 1.00 0.00

ATOM 3667 H VAL A 230 -15.093 29.931 106.250 1.00 0.00

ATOM 3668 H VAL A 230 -13.043 29.270 107.452 1.00 0.00

ATOM 3669 H VAL A 230 -13.075 28.619 105.769 1.00 0.00

ATOM 3670 H VAL A 230 -12.050 30.052 106.163 1.00 0.00

ATOM 3671 H VAL A 230 -14.132 31.380 107.958 1.00 0.00

ATOM 3672 H VAL A 230 -13.163 32.218 106.685 1.00 0.00

ATOM 3673 H VAL A 230 -14.966 32.318 106.661 1.00 0.00

ATOM 3674 N THR A 231 -15.145 28.903 103.503 1.00 68.64

ATOM 3675 CA THR A 231 -15.270 27.725 102.666 1.00 67.79

ATOM 3676 C THR A 231 -14.648 27.914 101.293 1.00 66.19

ATOM 3677 O THR A 231 -13.929 27.059 100.819 1.00 65.03

ATOM 3678 CB THR A 231 -16.738 27.328 102.589 1.00 70.99

ATOM 3679 OG1 THR A 231 -17.503 28.489 102.863 1.00 74.04

ATOM 3680 CG2 THR A 231 -17.123 26.285 103.604 1.00 72.05

ATOM 3681 H THR A 231 -15.983 29.228 104.006 1.00 0.00

ATOM 3682 H THR A 231 -14.706 26.911 103.121 1.00 0.00

ATOM 3683 H THR A 231 -16.920 26.910 101.599 1.00 0.00

ATOM 3684 H THR A 231 -17.235 29.198 102.166 1.00 0.00

ATOM 3685 H THR A 231 -18.183 26.077 103.461 1.00 0.00

ATOM 3686 H THR A 231 -16.508 25.407 103.407 1.00 0.00

ATOM 3687 H THR A 231 -16.922 26.708 104.588 1.00 0.00

ATOM 3688 N LYS A 232 -14.936 29.055 100.689 1.00 66.56

ATOM 3689 CA LYS A 232 -14.454 29.465 99.386 1.00 65.75

ATOM 3690 C LYS A 232 -12.900 29.648 99.366 1.00 63.53

ATOM 3691 O LYS A 232 -12.197 28.998 98.581 1.00 62.69

ATOM 3692 CB LYS A 232 -15.193 30.770 99.062 1.00 66.70

ATOM 3693 CG LYS A 232 -15.238 31.162 97.653 1.00 69.35

ATOM 3694 CD LYS A 232 -14.023 31.973 97.264 1.00 72.60

ATOM 3695 CE LYS A 232 -14.082 32.345 95.782 1.00 76.64

ATOM 3696 NZ LYS A 232 -13.485 31.274 94.935 1.00 78.00

ATOM 3697 H LYS A 232 -15.556 29.708 101.188 1.00 0.00

ATOM 3698 H LYS A 232 -14.653 28.702 98.633 1.00 0.00

ATOM 3699 H LYS A 232 -14.690 31.584 99.584 1.00 0.00

ATOM 3700 H LYS A 232 -16.237 30.597 99.323 1.00 0.00

ATOM 3701 H LYS A 232 -16.124 31.783 97.521 1.00 0.00

ATOM 3702 H LYS A 232 -15.289 30.258 97.046 1.00 0.00

ATOM 3703 H LYS A 232 -13.132 31.371 97.443 1.00 0.00

ATOM 3704 H LYS A 232 -13.995 32.881 97.866 1.00 0.00

ATOM 3705 H LYS A 232 -13.531 33.270 95.613 1.00 0.00

ATOM 3706 H LYS A 232 -15.127 32.451 95.491 1.00 0.00

ATOM 3707 H LYS A 232 -13.558 31.588 93.957 1.00 0.00

ATOM 3708 H LYS A 232 -12.504 31.167 95.231 1.00 0.00

ATOM 3709 H LYS A 232 -14.031 30.417 95.104 1.00 0.00

ATOM 3710 N LEU A 233 -12.362 30.532 100.210 1.00 62.13

ATOM 3711 CA LEU A 233 -10.899 30.645 100.356 1.00 60.11

ATOM 3712 C LEU A 233 -10.230 29.283 100.610 1.00 58.66

ATOM 3713 O LEU A 233 -9.124 29.031 100.175 1.00 56.78

ATOM 3714 CB LEU A 233 -10.513 31.622 101.484 1.00 59.51

ATOM 3715 CG LEU A 233 -11.147 33.002 101.649 1.00 61.36

ATOM 3716 CD1 LEU A 233 -10.531 33.523 102.868 1.00 63.46

ATOM 3717 CD2 LEU A 233 -10.844 33.939 100.501 1.00 62.93

ATOM 3718 H LEU A 233 -12.977 31.143 100.766 1.00 0.00

ATOM 3719 H LEU A 233 -10.534 31.036 99.406 1.00 0.00

ATOM 3720 H LEU A 233 -9.469 31.854 101.273 1.00 0.00

ATOM 3721 H LEU A 233 -10.839 31.100 102.384 1.00 0.00

ATOM 3722 H LEU A 233 -12.234 32.926 101.684 1.00 0.00

ATOM 3723 H LEU A 233 -10.949 34.515 103.037 1.00 0.00

ATOM 3724 H LEU A 233 -10.785 32.833 103.673 1.00 0.00

ATOM 3725 H LEU A 233 -9.456 33.561 102.691 1.00 0.00

ATOM 3726 H LEU A 233 -11.341 34.884 100.721 1.00 0.00

ATOM 3727 H LEU A 233 -9.761 34.051 100.458 1.00 0.00

ATOM 3728 H LEU A 233 -11.239 33.474 99.598 1.00 0.00

ATOM 3729 N VAL A 234 -10.911 28.398 101.314 1.00 60.35

ATOM 3730 CA VAL A 234 -10.384 27.049 101.542 1.00 61.05

ATOM 3731 C VAL A 234 -10.264 26.166 100.280 1.00 61.54

ATOM 3732 O VAL A 234 -9.165 25.640 99.966 1.00 60.35

ATOM 3733 CB VAL A 234 -11.101 26.360 102.683 1.00 61.86

ATOM 3734 CG1 VAL A 234 -10.846 24.871 102.647 1.00 62.43

ATOM 3735 CG2 VAL A 234 -10.576 26.941 103.985 1.00 63.38

ATOM 3736 H VAL A 234 -11.826 28.659 101.709 1.00 0.00

ATOM 3737 H VAL A 234 -9.346 27.196 101.842 1.00 0.00

ATOM 3738 H VAL A 234 -12.176 26.521 102.597 1.00 0.00

ATOM 3739 H VAL A 234 -11.385 24.435 103.488 1.00 0.00

ATOM 3740 H VAL A 234 -11.222 24.506 101.691 1.00 0.00

ATOM 3741 H VAL A 234 -9.769 24.730 102.739 1.00 0.00

ATOM 3742 H VAL A 234 -11.102 26.435 104.795 1.00 0.00

ATOM 3743 H VAL A 234 -9.504 26.744 104.012 1.00 0.00

ATOM 3744 H VAL A 234 -10.792 28.009 103.967 1.00 0.00

ATOM 3745 N THR A 235 -11.363 26.055 99.547 1.00 63.06

ATOM 3746 CA THR A 235 -11.365 25.440 98.224 1.00 64.20

ATOM 3747 C THR A 235 -10.167 25.908 97.335 1.00 63.39

ATOM 3748 O THR A 235 -9.386 25.084 96.786 1.00 62.74

ATOM 3749 CB THR A 235 -12.717 25.735 97.552 1.00 67.00

ATOM 3750 OG1 THR A 235 -13.777 25.336 98.446 1.00 68.34

ATOM 3751 CG2 THR A 235 -12.854 25.005 96.230 1.00 67.20

ATOM 3752 H THR A 235 -12.250 26.417 99.926 1.00 0.00

ATOM 3753 H THR A 235 -11.234 24.364 98.340 1.00 0.00

ATOM 3754 H THR A 235 -12.778 26.803 97.344 1.00 0.00

ATOM 3755 H THR A 235 -13.641 25.856 99.325 1.00 0.00

ATOM 3756 H THR A 235 -13.831 25.265 95.822 1.00 0.00

ATOM 3757 H THR A 235 -12.042 25.350 95.590 1.00 0.00

ATOM 3758 H THR A 235 -12.775 23.939 96.445 1.00 0.00

ATOM 3759 N ASP A 236 -9.993 27.220 97.232 1.00 63.31

ATOM 3760 CA ASP A 236 -8.948 27.755 96.367 1.00 63.11

ATOM 3761 C ASP A 236 -7.549 27.626 97.023 1.00 60.08

ATOM 3762 O ASP A 236 -6.546 27.417 96.321 1.00 58.89

ATOM 3763 CB ASP A 236 -9.328 29.135 95.724 1.00 64.33

ATOM 3764 CG ASP A 236 -10.360 28.990 94.482 1.00 72.10

ATOM 3765 OD1 ASP A 236 -10.184 28.060 93.608 1.00 75.79

ATOM 3766 OD2 ASP A 236 -11.338 29.803 94.361 1.00 74.68

ATOM 3767 H ASP A 236 -10.598 27.862 97.764 1.00 0.00

ATOM 3768 H ASP A 236 -8.865 27.127 95.480 1.00 0.00

ATOM 3769 H ASP A 236 -8.402 29.561 95.338 1.00 0.00

ATOM 3770 H ASP A 236 -9.786 29.748 96.500 1.00 0.00

ATOM 3771 N LEU A 237 -7.488 27.647 98.355 1.00 58.78

ATOM 3772 CA LEU A 237 -6.189 27.455 99.027 1.00 56.20

ATOM 3773 C LEU A 237 -5.727 25.966 99.053 1.00 54.93

ATOM 3774 O LEU A 237 -4.542 25.707 98.811 1.00 55.08

ATOM 3775 CB LEU A 237 -6.140 28.077 100.435 1.00 55.56

ATOM 3776 CG LEU A 237 -4.759 27.983 101.148 1.00 53.67

ATOM 3777 CD1 LEU A 237 -3.601 28.568 100.334 1.00 53.79

ATOM 3778 CD2 LEU A 237 -4.727 28.676 102.475 1.00 53.25

ATOM 3779 H LEU A 237 -8.342 27.796 98.911 1.00 0.00

ATOM 3780 H LEU A 237 -5.473 28.000 98.411 1.00 0.00

ATOM 3781 H LEU A 237 -6.847 27.514 101.044 1.00 0.00

ATOM 3782 H LEU A 237 -6.373 29.136 100.320 1.00 0.00

ATOM 3783 H LEU A 237 -4.629 26.908 101.271 1.00 0.00

ATOM 3784 H LEU A 237 -2.698 28.445 100.931 1.00 0.00

ATOM 3785 H LEU A 237 -3.550 28.005 99.402 1.00 0.00

ATOM 3786 H LEU A 237 -3.832 29.619 100.161 1.00 0.00

ATOM 3787 H LEU A 237 -3.723 28.546 102.878 1.00 0.00

ATOM 3788 H LEU A 237 -4.958 29.725 102.292 1.00 0.00

ATOM 3789 H LEU A 237 -5.480 28.199 103.102 1.00 0.00

ATOM 3790 N THR A 238 -6.606 25.004 99.329 1.00 53.57

ATOM 3791 CA THR A 238 -6.213 23.585 99.125 1.00 54.42

ATOM 3792 C THR A 238 -5.738 23.254 97.671 1.00 54.44

ATOM 3793 O THR A 238 -4.729 22.569 97.460 1.00 53.06

ATOM 3794 CB THR A 238 -7.346 22.652 99.511 1.00 56.17

ATOM 3795 OG1 THR A 238 -7.733 22.963 100.857 1.00 59.91

ATOM 3796 CG2 THR A 238 -6.949 21.167 99.412 1.00 54.72

ATOM 3797 H THR A 238 -7.545 25.240 99.679 1.00 0.00

ATOM 3798 H THR A 238 -5.354 23.429 99.778 1.00 0.00

ATOM 3799 H THR A 238 -8.171 22.801 98.815 1.00 0.00

ATOM 3800 H THR A 238 -7.988 23.961 100.874 1.00 0.00

ATOM 3801 H THR A 238 -7.821 20.584 99.707 1.00 0.00

ATOM 3802 H THR A 238 -6.668 20.981 98.375 1.00 0.00

ATOM 3803 H THR A 238 -6.111 21.015 100.093 1.00 0.00

ATOM 3804 N LYS A 239 -6.459 23.761 96.682 1.00 55.05

ATOM 3805 CA LYS A 239 -6.023 23.615 95.293 1.00 55.50

ATOM 3806 C LYS A 239 -4.607 24.077 94.982 1.00 54.58

ATOM 3807 O LYS A 239 -3.881 23.386 94.315 1.00 53.77

ATOM 3808 CB LYS A 239 -6.980 24.302 94.334 1.00 55.75

ATOM 3809 CG LYS A 239 -6.505 24.099 92.928 1.00 56.02

ATOM 3810 CD LYS A 239 -7.497 24.712 91.883 1.00 66.34

ATOM 3811 CE LYS A 239 -7.008 24.482 90.446 1.00 65.86

ATOM 3812 NZ LYS A 239 -6.406 23.107 90.482 1.00 65.92

ATOM 3813 H LYS A 239 -7.334 24.262 96.893 1.00 0.00

ATOM 3814 H LYS A 239 -6.026 22.534 95.150 1.00 0.00

ATOM 3815 H LYS A 239 -7.027 25.369 94.552 1.00 0.00

ATOM 3816 H LYS A 239 -7.974 23.868 94.438 1.00 0.00

ATOM 3817 H LYS A 239 -6.464 23.022 92.768 1.00 0.00

ATOM 3818 H LYS A 239 -5.523 24.562 92.830 1.00 0.00

ATOM 3819 H LYS A 239 -7.582 25.785 92.052 1.00 0.00

ATOM 3820 H LYS A 239 -8.462 24.216 91.989 1.00 0.00

ATOM 3821 H LYS A 239 -6.296 25.236 90.111 1.00 0.00

ATOM 3822 H LYS A 239 -7.818 24.543 89.720 1.00 0.00

ATOM 3823 H LYS A 239 -6.061 22.905 89.533 1.00 0.00

ATOM 3824 H LYS A 239 -5.641 23.131 91.171 1.00 0.00

ATOM 3825 H LYS A 239 -7.154 22.458 90.764 1.00 0.00

ATOM 3826 N VAL A 240 -4.258 25.273 95.436 1.00 54.90

ATOM 3827 CA VAL A 240 -2.918 25.850 95.291 1.00 54.40

ATOM 3828 C VAL A 240 -1.839 24.931 95.924 1.00 54.16

ATOM 3829 O VAL A 240 -0.804 24.626 95.306 1.00 54.51

ATOM 3830 CB VAL A 240 -2.910 27.318 95.951 1.00 55.60

ATOM 3831 CG1 VAL A 240 -1.527 27.847 96.252 1.00 52.60

ATOM 3832 CG2 VAL A 240 -3.749 28.301 95.132 1.00 51.81

ATOM 3833 H VAL A 240 -4.974 25.832 95.921 1.00 0.00

ATOM 3834 H VAL A 240 -2.671 25.935 94.233 1.00 0.00

ATOM 3835 H VAL A 240 -3.384 27.213 96.927 1.00 0.00

ATOM 3836 H VAL A 240 -1.653 28.837 96.691 1.00 0.00

ATOM 3837 H VAL A 240 -1.064 27.150 96.950 1.00 0.00

ATOM 3838 H VAL A 240 -0.989 27.889 95.305 1.00 0.00

ATOM 3839 H VAL A 240 -3.695 29.265 95.638 1.00 0.00

ATOM 3840 H VAL A 240 -3.310 28.342 94.135 1.00 0.00

ATOM 3841 H VAL A 240 -4.767 27.911 95.111 1.00 0.00

ATOM 3842 N HIS A 241 -2.105 24.462 97.137 1.00 54.20

ATOM 3843 CA HIS A 241 -1.224 23.497 97.784 1.00 54.25

ATOM 3844 C HIS A 241 -1.130 22.156 97.119 1.00 54.31

ATOM 3845 O HIS A 241 -0.026 21.782 96.789 1.00 55.74

ATOM 3846 CB HIS A 241 -1.468 23.362 99.285 1.00 54.25

ATOM 3847 CG HIS A 241 -1.024 24.557 100.034 1.00 54.20

ATOM 3848 ND1 HIS A 241 -1.821 25.194 100.951 1.00 57.89

ATOM 3849 CD2 HIS A 241 0.091 25.307 99.918 1.00 55.54

ATOM 3850 CE1 HIS A 241 -1.209 26.268 101.404 1.00 53.06

ATOM 3851 NE2 HIS A 241 -0.051 26.368 100.779 1.00 58.77

ATOM 3852 H HIS A 241 -2.949 24.786 97.630 1.00 0.00

ATOM 3853 H HIS A 241 -0.240 23.947 97.649 1.00 0.00

ATOM 3854 H HIS A 241 -0.919 22.501 99.666 1.00 0.00

ATOM 3855 H HIS A 241 -2.542 23.271 99.446 1.00 0.00

ATOM 3856 H HIS A 241 -2.758 24.879 101.242 1.00 0.00

ATOM 3857 H HIS A 241 0.942 25.109 99.266 1.00 0.00

ATOM 3858 H HIS A 241 -1.591 26.953 102.161 1.00 0.00

ATOM 3859 N LYS A 242 -2.245 21.448 96.932 1.00 55.01

ATOM 3860 CA LYS A 242 -2.314 20.229 96.082 1.00 55.16

ATOM 3861 C LYS A 242 -1.437 20.376 94.864 1.00 55.26

ATOM 3862 O LYS A 242 -0.565 19.569 94.616 1.00 56.36

ATOM 3863 CB LYS A 242 -3.771 19.911 95.727 1.00 55.43

ATOM 3864 CG LYS A 242 -4.086 18.737 94.809 1.00 57.28

ATOM 3865 CD LYS A 242 -5.656 18.660 94.417 1.00 61.25

ATOM 3866 CE LYS A 242 -6.218 20.022 93.687 1.00 65.76

ATOM 3867 NZ LYS A 242 -7.692 20.078 93.119 1.00 68.50

ATOM 3868 H LYS A 242 -3.106 21.763 97.402 1.00 0.00

ATOM 3869 H LYS A 242 -1.927 19.378 96.643 1.00 0.00

ATOM 3870 H LYS A 242 -4.124 20.787 95.183 1.00 0.00

ATOM 3871 H LYS A 242 -4.233 19.642 96.677 1.00 0.00

ATOM 3872 H LYS A 242 -3.841 17.844 95.384 1.00 0.00

ATOM 3873 H LYS A 242 -3.472 18.838 93.914 1.00 0.00

ATOM 3874 H LYS A 242 -6.209 18.536 95.348 1.00 0.00

ATOM 3875 H LYS A 242 -5.780 17.828 93.724 1.00 0.00

ATOM 3876 H LYS A 242 -5.588 20.176 92.811 1.00 0.00

ATOM 3877 H LYS A 242 -6.244 20.762 94.487 1.00 0.00

ATOM 3878 H LYS A 242 -7.822 21.015 92.712 1.00 0.00

ATOM 3879 H LYS A 242 -7.769 19.337 92.408 1.00 0.00

ATOM 3880 H LYS A 242 -8.327 19.914 93.913 1.00 0.00

ATOM 3881 N GLU A 243 -1.594 21.439 94.116 1.00 55.46

ATOM 3882 CA GLU A 243 -0.854 21.518 92.875 1.00 55.43

ATOM 3883 C GLU A 243 0.629 21.722 93.181 1.00 55.45

ATOM 3884 O GLU A 243 1.505 21.092 92.572 1.00 54.27

ATOM 3885 CB GLU A 243 -1.388 22.670 92.043 1.00 56.24

ATOM 3886 CG GLU A 243 -2.838 22.614 91.870 1.00 54.86

ATOM 3887 CD GLU A 243 -3.340 23.737 91.014 1.00 54.79

ATOM 3888 OE1 GLU A 243 -3.389 24.907 91.405 1.00 58.10

ATOM 3889 OE2 GLU A 243 -3.725 23.445 89.923 1.00 59.04

ATOM 3890 H GLU A 243 -2.227 22.199 94.403 1.00 0.00

ATOM 3891 H GLU A 243 -0.973 20.592 92.312 1.00 0.00

ATOM 3892 H GLU A 243 -0.932 22.647 91.053 1.00 0.00

ATOM 3893 H GLU A 243 -1.178 23.595 92.580 1.00 0.00

ATOM 3894 H GLU A 243 -3.286 22.711 92.859 1.00 0.00

ATOM 3895 H GLU A 243 -3.090 21.662 91.403 1.00 0.00

ATOM 3896 N CYS A 244 0.905 22.618 94.131 1.00 55.51

ATOM 3897 CA CYS A 244 2.316 22.817 94.552 1.00 56.33

ATOM 3898 C CYS A 244 2.976 21.546 94.964 1.00 54.72

ATOM 3899 O CYS A 244 3.970 21.197 94.368 1.00 55.67

ATOM 3900 CB CYS A 244 2.498 23.977 95.517 1.00 55.55

ATOM 3901 SG CYS A 244 3.057 25.399 94.492 1.00 64.75

ATOM 3902 H CYS A 244 0.149 23.165 94.567 1.00 0.00

ATOM 3903 H CYS A 244 2.863 23.133 93.664 1.00 0.00

ATOM 3904 H CYS A 244 3.297 23.652 96.183 1.00 0.00

ATOM 3905 H CYS A 244 1.615 24.090 96.145 1.00 0.00

ATOM 3906 N CYS A 245 2.299 20.767 95.808 1.00 53.80

ATOM 3907 CA CYS A 245 2.802 19.484 96.330 1.00 54.19

ATOM 3908 C CYS A 245 2.994 18.373 95.299 1.00 54.70

ATOM 3909 O CYS A 245 3.736 17.441 95.534 1.00 54.79

ATOM 3910 CB CYS A 245 1.962 19.021 97.525 1.00 54.16

ATOM 3911 SG CYS A 245 2.300 20.112 99.032 1.00 55.94

ATOM 3912 H CYS A 245 1.367 21.081 96.114 1.00 0.00

ATOM 3913 H CYS A 245 3.819 19.695 96.662 1.00 0.00

ATOM 3914 H CYS A 245 2.396 18.041 97.723 1.00 0.00

ATOM 3915 H CYS A 245 0.931 18.920 97.185 1.00 0.00

ATOM 3916 N HIS A 246 2.343 18.529 94.156 1.00 55.34

ATOM 3917 CA HIS A 246 2.316 17.591 93.050 1.00 55.99

ATOM 3918 C HIS A 246 3.269 18.060 91.893 1.00 57.26

ATOM 3919 O HIS A 246 3.280 17.460 90.827 1.00 57.70

ATOM 3920 CB HIS A 246 0.858 17.483 92.549 1.00 55.97

ATOM 3921 CG HIS A 246 0.629 16.397 91.545 1.00 58.31

ATOM 3922 ND1 HIS A 246 0.158 16.645 90.266 1.00 62.78

ATOM 3923 CD2 HIS A 246 0.803 15.058 91.628 1.00 59.49

ATOM 3924 CE1 HIS A 246 0.078 15.510 89.596 1.00 63.95

ATOM 3925 NE2 HIS A 246 0.461 14.529 90.404 1.00 66.74

ATOM 3926 H HIS A 246 1.805 19.400 94.042 1.00 0.00

ATOM 3927 H HIS A 246 2.670 16.615 93.382 1.00 0.00

ATOM 3928 H HIS A 246 0.587 18.424 92.070 1.00 0.00

ATOM 3929 H HIS A 246 0.243 17.224 93.410 1.00 0.00

ATOM 3930 H HIS A 246 -0.092 17.573 89.895 1.00 0.00

ATOM 3931 H HIS A 246 1.149 14.503 92.500 1.00 0.00

ATOM 3932 H HIS A 246 -0.246 15.398 88.561 1.00 0.00

ATOM 3933 N GLY A 247 4.058 19.121 92.088 1.00 57.31

ATOM 3934 CA GLY A 247 4.985 19.606 91.005 1.00 57.69

ATOM 3935 C GLY A 247 4.365 20.418 89.839 1.00 58.34

ATOM 3936 O GLY A 247 5.048 20.753 88.847 1.00 59.33

ATOM 3937 H GLY A 247 4.032 19.614 92.992 1.00 0.00

ATOM 3938 H GLY A 247 5.371 18.693 90.552 1.00 0.00

ATOM 3939 H GLY A 247 5.698 20.263 91.504 1.00 0.00

ATOM 3940 N ASP A 248 3.065 20.688 89.930 1.00 57.25

ATOM 3941 CA ASP A 248 2.344 21.550 89.013 1.00 57.85

ATOM 3942 C ASP A 248 2.656 23.028 89.477 1.00 55.51

ATOM 3943 O ASP A 248 1.800 23.764 89.911 1.00 52.60

ATOM 3944 CB ASP A 248 0.815 21.219 89.023 1.00 58.54

ATOM 3945 CG ASP A 248 0.504 19.709 88.794 1.00 65.54

ATOM 3946 OD1 ASP A 248 1.327 18.961 88.158 1.00 72.26

ATOM 3947 OD2 ASP A 248 -0.608 19.250 89.205 1.00 69.01

ATOM 3948 H ASP A 248 2.537 20.257 90.702 1.00 0.00

ATOM 3949 H ASP A 248 2.658 21.406 87.979 1.00 0.00

ATOM 3950 H ASP A 248 0.351 21.776 88.209 1.00 0.00

ATOM 3951 H ASP A 248 0.429 21.484 90.007 1.00 0.00

ATOM 3952 N LEU A 249 3.920 23.409 89.374 1.00 55.12

ATOM 3953 CA LEU A 249 4.414 24.667 89.900 1.00 55.05

ATOM 3954 C LEU A 249 3.909 25.913 89.185 1.00 55.74

ATOM 3955 O LEU A 249 3.715 26.923 89.837 1.00 55.89

ATOM 3956 CB LEU A 249 5.908 24.667 89.885 1.00 55.16

ATOM 3957 CG LEU A 249 6.580 23.551 90.626 1.00 57.84

ATOM 3958 CD1 LEU A 249 8.044 23.949 90.781 1.00 63.88

ATOM 3959 CD2 LEU A 249 5.939 23.251 92.016 1.00 55.49

ATOM 3960 H LEU A 249 4.584 22.782 88.898 1.00 0.00

ATOM 3961 H LEU A 249 4.019 24.726 90.914 1.00 0.00

ATOM 3962 H LEU A 249 6.214 25.588 90.381 1.00 0.00

ATOM 3963 H LEU A 249 6.201 24.560 88.840 1.00 0.00

ATOM 3964 H LEU A 249 6.466 22.627 90.060 1.00 0.00

ATOM 3965 H LEU A 249 8.539 23.141 91.320 1.00 0.00

ATOM 3966 H LEU A 249 8.448 24.074 89.777 1.00 0.00

ATOM 3967 H LEU A 249 8.063 24.882 91.344 1.00 0.00

ATOM 3968 H LEU A 249 6.505 22.430 92.457 1.00 0.00

ATOM 3969 H LEU A 249 6.020 24.163 92.608 1.00 0.00

ATOM 3970 H LEU A 249 4.900 22.976 91.835 1.00 0.00

ATOM 3971 N LEU A 250 3.746 25.831 87.860 1.00 56.27

ATOM 3972 CA LEU A 250 3.139 26.884 87.046 1.00 56.25

ATOM 3973 C LEU A 250 1.662 27.133 87.366 1.00 55.30

ATOM 3974 O LEU A 250 1.293 28.303 87.467 1.00 56.01

ATOM 3975 CB LEU A 250 3.361 26.657 85.548 1.00 56.90

ATOM 3976 CG LEU A 250 4.775 26.398 84.981 1.00 58.05

ATOM 3977 CD1 LEU A 250 4.662 26.145 83.473 1.00 63.77

ATOM 3978 CD2 LEU A 250 5.776 27.506 85.136 1.00 56.92

ATOM 3979 H LEU A 250 4.067 24.976 87.385 1.00 0.00

ATOM 3980 H LEU A 250 3.663 27.799 87.322 1.00 0.00

ATOM 3981 H LEU A 250 3.067 27.606 85.099 1.00 0.00

ATOM 3982 H LEU A 250 2.812 25.740 85.336 1.00 0.00

ATOM 3983 H LEU A 250 5.142 25.554 85.566 1.00 0.00

ATOM 3984 H LEU A 250 5.672 25.966 83.103 1.00 0.00

ATOM 3985 H LEU A 250 4.023 25.272 83.343 1.00 0.00

ATOM 3986 H LEU A 250 4.221 27.039 83.032 1.00 0.00

ATOM 3987 H LEU A 250 6.707 27.160 84.687 1.00 0.00

ATOM 3988 H LEU A 250 5.373 28.375 84.615 1.00 0.00

ATOM 3989 H LEU A 250 5.885 27.689 86.205 1.00 0.00

ATOM 3990 N GLU A 251 0.858 26.084 87.585 1.00 59.16

ATOM 3991 CA GLU A 251 -0.582 26.219 87.905 1.00 60.52

ATOM 3992 C GLU A 251 -0.750 26.766 89.287 1.00 59.76

ATOM 3993 O GLU A 251 -1.680 27.543 89.550 1.00 59.34

ATOM 3994 CB GLU A 251 -1.349 24.885 87.922 1.00 61.53

ATOM 3995 CG GLU A 251 -1.554 24.179 86.617 1.00 66.82

ATOM 3996 CD GLU A 251 -0.354 23.355 86.163 1.00 70.84

ATOM 3997 OE1 GLU A 251 0.797 23.874 86.226 1.00 68.69

ATOM 3998 OE2 GLU A 251 -0.581 22.183 85.721 1.00 76.37

ATOM 3999 H GLU A 251 1.261 25.138 87.528 1.00 0.00

ATOM 4000 H GLU A 251 -0.976 26.865 87.121 1.00 0.00

ATOM 4001 H GLU A 251 -2.350 25.105 88.293 1.00 0.00

ATOM 4002 H GLU A 251 -0.729 24.202 88.503 1.00 0.00

ATOM 4003 H GLU A 251 -1.716 24.954 85.868 1.00 0.00

ATOM 4004 H GLU A 251 -2.399 23.502 86.745 1.00 0.00

ATOM 4005 N CYS A 252 0.130 26.310 90.170 1.00 59.99

ATOM 4006 CA CYS A 252 0.156 26.688 91.566 1.00 60.71

ATOM 4007 C CYS A 252 0.372 28.203 91.708 1.00 60.97

ATOM 4008 O CYS A 252 -0.457 28.902 92.333 1.00 60.60

ATOM 4009 CB CYS A 252 1.306 25.949 92.263 1.00 61.51

ATOM 4010 SG CYS A 252 1.444 26.411 93.962 1.00 64.38

ATOM 4011 H CYS A 252 0.842 25.643 89.839 1.00 0.00

ATOM 4012 H CYS A 252 -0.797 26.423 92.023 1.00 0.00

ATOM 4013 H CYS A 252 2.188 26.325 91.744 1.00 0.00

ATOM 4014 H CYS A 252 1.164 24.881 92.095 1.00 0.00

ATOM 4015 N ALA A 253 1.489 28.691 91.131 1.00 61.19

ATOM 4016 CA ALA A 253 1.872 30.115 91.157 1.00 61.41

ATOM 4017 C ALA A 253 0.727 31.012 90.710 1.00 61.95

ATOM 4018 O ALA A 253 0.268 31.906 91.433 1.00 61.73

ATOM 4019 CB ALA A 253 3.031 30.328 90.289 1.00 60.90

ATOM 4020 H ALA A 253 2.111 28.030 90.644 1.00 0.00

ATOM 4021 H ALA A 253 2.122 30.377 92.185 1.00 0.00

ATOM 4022 H ALA A 253 3.277 31.389 90.340 1.00 0.00

ATOM 4023 H ALA A 253 3.838 29.706 90.677 1.00 0.00

ATOM 4024 H ALA A 253 2.736 30.028 89.284 1.00 0.00

ATOM 4025 N ASP A 254 0.249 30.732 89.510 1.00 63.17

ATOM 4026 CA ASP A 254 -0.836 31.488 88.900 1.00 63.85

ATOM 4027 C ASP A 254 -2.132 31.480 89.751 1.00 62.29

ATOM 4028 O ASP A 254 -2.878 32.453 89.760 1.00 63.14

ATOM 4029 CB ASP A 254 -1.057 30.977 87.467 1.00 65.31

ATOM 4030 CG ASP A 254 -1.994 31.859 86.652 1.00 69.80

ATOM 4031 OD1 ASP A 254 -2.364 31.490 85.503 1.00 71.68

ATOM 4032 OD2 ASP A 254 -2.373 32.937 87.155 1.00 74.28

ATOM 4033 H ASP A 254 0.661 29.947 88.985 1.00 0.00

ATOM 4034 H ASP A 254 -0.548 32.538 88.856 1.00 0.00

ATOM 4035 H ASP A 254 -1.507 29.986 87.528 1.00 0.00

ATOM 4036 H ASP A 254 -0.091 30.980 86.963 1.00 0.00

ATOM 4037 N ASP A 255 -2.388 30.392 90.455 1.00 61.06

ATOM 4038 CA ASP A 255 -3.597 30.259 91.287 1.00 61.73

ATOM 4039 C ASP A 255 -3.436 30.952 92.644 1.00 59.40

ATOM 4040 O ASP A 255 -4.385 31.406 93.192 1.00 60.56

ATOM 4041 CB ASP A 255 -4.025 28.775 91.466 1.00 61.75

ATOM 4042 CG ASP A 255 -4.648 28.161 90.193 1.00 68.06

ATOM 4043 OD1 ASP A 255 -5.315 28.874 89.399 1.00 74.17

ATOM 4044 OD2 ASP A 255 -4.517 26.938 89.985 1.00 72.94

ATOM 4045 H ASP A 255 -1.719 29.609 90.421 1.00 0.00

ATOM 4046 H ASP A 255 -4.398 30.766 90.750 1.00 0.00

ATOM 4047 H ASP A 255 -4.778 28.737 92.253 1.00 0.00

ATOM 4048 H ASP A 255 -3.129 28.200 91.699 1.00 0.00

ATOM 4049 N ARG A 256 -2.236 31.002 93.180 1.00 58.01

ATOM 4050 CA ARG A 256 -1.920 31.867 94.304 1.00 57.90

ATOM 4051 C ARG A 256 -2.090 33.343 94.000 1.00 59.48

ATOM 4052 O ARG A 256 -2.585 34.123 94.846 1.00 58.89

ATOM 4053 CB ARG A 256 -0.496 31.673 94.678 1.00 57.90

ATOM 4054 CG ARG A 256 -0.259 31.732 96.133 1.00 59.79

ATOM 4055 CD ARG A 256 1.240 31.915 96.419 1.00 61.03

ATOM 4056 NE ARG A 256 1.405 32.076 97.844 1.00 65.17

ATOM 4057 CZ ARG A 256 2.483 32.580 98.413 1.00 68.16

ATOM 4058 NH1 ARG A 256 3.501 32.965 97.650 1.00 69.81

ATOM 4059 NH2 ARG A 256 2.537 32.680 99.740 1.00 69.22

ATOM 4060 H ARG A 256 -1.493 30.406 92.789 1.00 0.00

ATOM 4061 H ARG A 256 -2.613 31.594 95.100 1.00 0.00

ATOM 4062 H ARG A 256 0.082 32.475 94.218 1.00 0.00

ATOM 4063 H ARG A 256 -0.201 30.679 94.342 1.00 0.00

ATOM 4064 H ARG A 256 -0.589 30.784 96.557 1.00 0.00

ATOM 4065 H ARG A 256 -0.837 32.554 96.555 1.00 0.00

ATOM 4066 H ARG A 256 1.669 32.768 95.893 1.00 0.00

ATOM 4067 H ARG A 256 1.790 31.023 96.119 1.00 0.00

ATOM 4068 H ARG A 256 0.632 31.778 98.456 1.00 0.00

ATOM 4069 H ARG A 256 4.348 33.360 98.083 1.00 0.00

ATOM 4070 H ARG A 256 3.443 32.868 96.626 1.00 0.00

ATOM 4071 H ARG A 256 3.375 33.072 100.193 1.00 0.00

ATOM 4072 H ARG A 256 1.741 32.365 100.313 1.00 0.00

ATOM 4073 N ALA A 257 -1.698 33.756 92.795 1.00 60.40

ATOM 4074 CA ALA A 257 -1.889 35.139 92.468 1.00 62.05

ATOM 4075 C ALA A 257 -3.398 35.410 92.453 1.00 63.18

ATOM 4076 O ALA A 257 -3.863 36.384 93.054 1.00 64.35

ATOM 4077 CB ALA A 257 -1.266 35.463 91.168 1.00 63.65

ATOM 4078 H ALA A 257 -1.272 33.104 92.121 1.00 0.00

ATOM 4079 H ALA A 257 -1.408 35.776 93.210 1.00 0.00

ATOM 4080 H ALA A 257 -1.448 36.522 90.983 1.00 0.00

ATOM 4081 H ALA A 257 -0.202 35.245 91.261 1.00 0.00

ATOM 4082 H ALA A 257 -1.745 34.832 90.419 1.00 0.00

ATOM 4083 N ASP A 258 -4.174 34.519 91.840 1.00 63.45

ATOM 4084 CA ASP A 258 -5.590 34.794 91.602 1.00 63.94

ATOM 4085 C ASP A 258 -6.245 34.864 92.911 1.00 62.61

ATOM 4086 O ASP A 258 -7.144 35.672 93.097 1.00 64.80

ATOM 4087 CB ASP A 258 -6.270 33.724 90.759 1.00 65.48

ATOM 4088 CG ASP A 258 -5.712 33.628 89.335 1.00 68.63

ATOM 4089 OD1 ASP A 258 -5.074 34.582 88.839 1.00 76.32

ATOM 4090 OD2 ASP A 258 -5.931 32.594 88.675 1.00 73.82

ATOM 4091 H ASP A 258 -3.771 33.623 91.529 1.00 0.00

ATOM 4092 H ASP A 258 -5.673 35.728 91.046 1.00 0.00

ATOM 4093 H ASP A 258 -7.324 33.993 90.682 1.00 0.00

ATOM 4094 H ASP A 258 -6.104 32.764 91.248 1.00 0.00

ATOM 4095 N LEU A 259 -5.764 34.068 93.856 1.00 59.81

ATOM 4096 CA LEU A 259 -6.263 34.167 95.228 1.00 59.15

ATOM 4097 C LEU A 259 -5.810 35.456 95.990 1.00 59.59

ATOM 4098 O LEU A 259 -6.646 36.175 96.599 1.00 61.50

ATOM 4099 CB LEU A 259 -5.913 32.889 95.993 1.00 58.25

ATOM 4100 CG LEU A 259 -6.562 32.801 97.363 1.00 57.46

ATOM 4101 CD1 LEU A 259 -8.064 32.535 97.295 1.00 57.65

ATOM 4102 CD2 LEU A 259 -5.822 31.808 98.241 1.00 53.23

ATOM 4103 H LEU A 259 -5.036 33.377 93.623 1.00 0.00

ATOM 4104 H LEU A 259 -7.347 34.267 95.166 1.00 0.00

ATOM 4105 H LEU A 259 -4.833 32.877 96.142 1.00 0.00

ATOM 4106 H LEU A 259 -6.284 32.047 95.408 1.00 0.00

ATOM 4107 H LEU A 259 -6.474 33.781 97.832 1.00 0.00

ATOM 4108 H LEU A 259 -8.427 32.491 98.322 1.00 0.00

ATOM 4109 H LEU A 259 -8.510 33.363 96.743 1.00 0.00

ATOM 4110 H LEU A 259 -8.196 31.585 96.777 1.00 0.00

ATOM 4111 H LEU A 259 -6.333 31.791 99.204 1.00 0.00

ATOM 4112 H LEU A 259 -5.870 30.841 97.739 1.00 0.00

ATOM 4113 H LEU A 259 -4.797 32.166 98.333 1.00 0.00

ATOM 4114 N ALA A 260 -4.515 35.765 95.973 1.00 58.27

ATOM 4115 CA ALA A 260 -4.073 37.050 96.491 1.00 59.50

ATOM 4116 C ALA A 260 -4.969 38.143 95.935 1.00 62.31

ATOM 4117 O ALA A 260 -5.461 38.994 96.661 1.00 63.64

ATOM 4118 CB ALA A 260 -2.624 37.312 96.125 1.00 59.25

ATOM 4119 H ALA A 260 -3.829 35.097 95.594 1.00 0.00

ATOM 4120 H ALA A 260 -4.142 37.040 97.579 1.00 0.00

ATOM 4121 H ALA A 260 -2.361 38.284 96.542 1.00 0.00

ATOM 4122 H ALA A 260 -2.034 36.509 96.566 1.00 0.00

ATOM 4123 H ALA A 260 -2.564 37.308 95.037 1.00 0.00

ATOM 4124 N LYS A 261 -5.199 38.113 94.634 1.00 64.53

ATOM 4125 CA LYS A 261 -6.076 39.088 94.000 1.00 67.51

ATOM 4126 C LYS A 261 -7.517 39.012 94.553 1.00 68.37

ATOM 4127 O LYS A 261 -8.133 40.048 94.800 1.00 71.35

ATOM 4128 CB LYS A 261 -6.025 38.924 92.478 1.00 68.78

ATOM 4129 CG LYS A 261 -7.005 39.810 91.704 1.00 74.19

ATOM 4130 CD LYS A 261 -6.795 39.715 90.196 1.00 76.82

ATOM 4131 CE LYS A 261 -7.544 40.834 89.471 1.00 80.89

ATOM 4132 NZ LYS A 261 -7.462 40.668 87.981 1.00 81.81

ATOM 4133 H LYS A 261 -4.750 37.387 94.058 1.00 0.00

ATOM 4134 H LYS A 261 -5.716 40.088 94.243 1.00 0.00

ATOM 4135 H LYS A 261 -6.294 37.890 92.261 1.00 0.00

ATOM 4136 H LYS A 261 -5.022 39.211 92.162 1.00 0.00

ATOM 4137 H LYS A 261 -6.844 40.845 92.006 1.00 0.00

ATOM 4138 H LYS A 261 -8.017 39.472 91.926 1.00 0.00

ATOM 4139 H LYS A 261 -7.177 38.755 89.850 1.00 0.00

ATOM 4140 H LYS A 261 -5.730 39.808 89.983 1.00 0.00

ATOM 4141 H LYS A 261 -7.104 41.795 89.738 1.00 0.00

ATOM 4142 H LYS A 261 -8.595 40.788 89.754 1.00 0.00

ATOM 4143 H LYS A 261 -7.984 41.451 87.562 1.00 0.00

ATOM 4144 H LYS A 261 -6.464 40.695 87.729 1.00 0.00

ATOM 4145 H LYS A 261 -7.889 39.758 87.755 1.00 0.00

ATOM 4146 N TYR A 262 -8.067 37.827 94.768 1.00 66.94

ATOM 4147 CA TYR A 262 -9.441 37.757 95.265 1.00 68.09

ATOM 4148 C TYR A 262 -9.570 38.261 96.702 1.00 68.36

ATOM 4149 O TYR A 262 -10.620 38.780 97.107 1.00 69.22

ATOM 4150 CB TYR A 262 -9.954 36.322 95.235 1.00 67.56

ATOM 4151 CG TYR A 262 -11.342 36.168 95.772 1.00 65.25

ATOM 4152 CD1 TYR A 262 -12.441 36.136 94.918 1.00 70.09

ATOM 4153 CD2 TYR A 262 -11.560 36.049 97.132 1.00 64.43

ATOM 4154 CE1 TYR A 262 -13.781 36.007 95.419 1.00 70.79

ATOM 4155 CE2 TYR A 262 -12.857 35.923 97.666 1.00 66.82

ATOM 4156 CZ TYR A 262 -13.970 35.884 96.795 1.00 70.85

ATOM 4157 OH TYR A 262 -15.240 35.738 97.317 1.00 71.16

ATOM 4158 H TYR A 262 -7.534 36.965 94.587 1.00 0.00

ATOM 4159 H TYR A 262 -10.029 38.396 94.606 1.00 0.00

ATOM 4160 H TYR A 262 -9.296 35.712 95.854 1.00 0.00

ATOM 4161 H TYR A 262 -9.987 36.010 94.191 1.00 0.00

ATOM 4162 H TYR A 262 -12.281 36.211 93.842 1.00 0.00

ATOM 4163 H TYR A 262 -10.706 36.053 97.809 1.00 0.00

ATOM 4164 H TYR A 262 -14.632 36.005 94.738 1.00 0.00

ATOM 4165 H TYR A 262 -13.002 35.856 98.744 1.00 0.00

ATOM 4166 H TYR A 262 -15.895 35.746 96.522 1.00 0.00

ATOM 4167 N ILE A 263 -8.530 38.052 97.492 1.00 66.66

ATOM 4168 CA ILE A 263 -8.601 38.401 98.906 1.00 67.33

ATOM 4169 C ILE A 263 -8.632 39.920 99.050 1.00 70.08

ATOM 4170 O ILE A 263 -9.547 40.496 99.662 1.00 72.60

ATOM 4171 CB ILE A 263 -7.424 37.723 99.718 1.00 65.42

ATOM 4172 CG1 ILE A 263 -7.850 36.323 100.230 1.00 63.49

ATOM 4173 CG2 ILE A 263 -7.010 38.585 100.864 1.00 63.10

ATOM 4174 CD1 ILE A 263 -6.708 35.458 100.837 1.00 60.97

ATOM 4175 H ILE A 263 -7.667 37.641 97.107 1.00 0.00

ATOM 4176 H ILE A 263 -9.522 38.010 99.338 1.00 0.00

ATOM 4177 H ILE A 263 -6.573 37.605 99.047 1.00 0.00

ATOM 4178 H ILE A 263 -8.225 35.779 99.363 1.00 0.00

ATOM 4179 H ILE A 263 -8.574 36.490 101.027 1.00 0.00

ATOM 4180 H ILE A 263 -6.202 38.065 101.379 1.00 0.00

ATOM 4181 H ILE A 263 -6.678 39.535 100.445 1.00 0.00

ATOM 4182 H ILE A 263 -7.885 38.708 101.503 1.00 0.00

ATOM 4183 H ILE A 263 -7.155 34.514 101.149 1.00 0.00

ATOM 4184 H ILE A 263 -5.965 35.315 100.052 1.00 0.00

ATOM 4185 H ILE A 263 -6.302 36.014 101.682 1.00 0.00

ATOM 4186 N CYS A 264 -7.658 40.579 98.433 1.00 71.17

ATOM 4187 CA CYS A 264 -7.569 42.025 98.446 1.00 73.75

ATOM 4188 C CYS A 264 -8.797 42.723 97.920 1.00 76.12

ATOM 4189 O CYS A 264 -9.125 43.804 98.369 1.00 78.31

ATOM 4190 CB CYS A 264 -6.383 42.421 97.591 1.00 75.30

ATOM 4191 SG CYS A 264 -4.893 41.888 98.368 1.00 74.91

ATOM 4192 H CYS A 264 -6.939 40.042 97.927 1.00 0.00

ATOM 4193 H CYS A 264 -7.464 42.333 99.486 1.00 0.00

ATOM 4194 H CYS A 264 -6.412 43.511 97.598 1.00 0.00

ATOM 4195 H CYS A 264 -6.546 42.059 96.576 1.00 0.00

ATOM 4196 N ASP A 265 -9.461 42.120 96.954 1.00 76.64

ATOM 4197 CA ASP A 265 -10.667 42.694 96.413 1.00 80.95

ATOM 4198 C ASP A 265 -11.895 42.343 97.223 1.00 80.78

ATOM 4199 O ASP A 265 -13.015 42.644 96.810 1.00 85.32

ATOM 4200 CB ASP A 265 -10.869 42.278 94.956 1.00 83.01

ATOM 4201 CG ASP A 265 -9.636 42.570 94.063 1.00 86.70

ATOM 4202 OD1 ASP A 265 -8.468 42.635 94.582 1.00 88.09

ATOM 4203 OD2 ASP A 265 -9.847 42.691 92.823 1.00 90.04

ATOM 4204 H ASP A 265 -9.113 41.226 96.580 1.00 0.00

ATOM 4205 H ASP A 265 -10.539 43.775 96.463 1.00 0.00

ATOM 4206 H ASP A 265 -11.704 42.862 94.569 1.00 0.00

ATOM 4207 H ASP A 265 -11.055 41.204 94.941 1.00 0.00

ATOM 4208 N ASN A 266 -11.713 41.722 98.375 1.00 77.48

ATOM 4209 CA ASN A 266 -12.846 41.409 99.215 1.00 77.61

ATOM 4210 C ASN A 266 -12.567 41.660 100.689 1.00 77.02

ATOM 4211 O ASN A 266 -13.240 41.096 101.564 1.00 77.32

ATOM 4212 CB ASN A 266 -13.354 39.987 98.954 1.00 76.13

ATOM 4213 CG ASN A 266 -14.048 39.859 97.597 1.00 78.44

ATOM 4214 OD1 ASN A 266 -15.252 40.052 97.507 1.00 82.96

ATOM 4215 ND2 ASN A 266 -13.284 39.582 96.535 1.00 74.60

ATOM 4216 H ASN A 266 -10.763 41.461 98.674 1.00 0.00

ATOM 4217 H ASN A 266 -13.646 42.096 98.942 1.00 0.00

ATOM 4218 H ASN A 266 -14.078 39.737 99.729 1.00 0.00

ATOM 4219 H ASN A 266 -12.496 39.315 98.958 1.00 0.00

ATOM 4220 H ASN A 266 -13.712 39.488 95.603 1.00 0.00

ATOM 4221 H ASN A 266 -12.267 39.463 96.649 1.00 0.00

ATOM 4222 N GLN A 267 -11.591 42.527 100.951 1.00 76.23

ATOM 4223 CA GLN A 267 -11.246 42.924 102.308 1.00 75.91

ATOM 4224 C GLN A 267 -12.400 43.059 103.297 1.00 77.73

ATOM 4225 O GLN A 267 -12.387 42.404 104.355 1.00 76.93

ATOM 4226 CB GLN A 267 -10.414 44.203 102.313 1.00 76.88

ATOM 4227 CG GLN A 267 -8.985 43.884 102.128 1.00 72.52

ATOM 4228 CD GLN A 267 -8.163 45.060 101.787 1.00 70.65

ATOM 4229 OE1 GLN A 267 -7.748 45.830 102.661 1.00 74.70

ATOM 4230 NE2 GLN A 267 -7.854 45.187 100.521 1.00 68.33

ATOM 4231 H GLN A 267 -11.061 42.931 100.165 1.00 0.00

ATOM 4232 H GLN A 267 -10.665 42.076 102.672 1.00 0.00

ATOM 4233 H GLN A 267 -10.540 44.736 103.255 1.00 0.00

ATOM 4234 H GLN A 267 -10.724 44.832 101.479 1.00 0.00

ATOM 4235 H GLN A 267 -8.909 43.179 101.301 1.00 0.00

ATOM 4236 H GLN A 267 -8.612 43.485 103.071 1.00 0.00

ATOM 4237 H GLN A 267 -7.282 45.984 100.208 1.00 0.00

ATOM 4238 H GLN A 267 -8.183 44.490 99.838 1.00 0.00

ATOM 4239 N ASP A 268 -13.387 43.884 102.963 1.00 80.92

ATOM 4240 CA ASP A 268 -14.456 44.227 103.903 1.00 82.69

ATOM 4241 C ASP A 268 -15.252 43.037 104.451 1.00 81.85

ATOM 4242 O ASP A 268 -15.766 43.075 105.576 1.00 82.95

ATOM 4243 CB ASP A 268 -15.377 45.292 103.291 1.00 86.97

ATOM 4244 CG ASP A 268 -14.631 46.602 103.015 1.00 90.39

ATOM 4245 OD1 ASP A 268 -15.279 47.609 102.611 1.00 92.64

ATOM 4246 OD2 ASP A 268 -13.378 46.607 103.221 1.00 88.88

ATOM 4247 H ASP A 268 -13.400 44.293 102.018 1.00 0.00

ATOM 4248 H ASP A 268 -13.956 44.634 104.781 1.00 0.00

ATOM 4249 H ASP A 268 -16.180 45.498 103.998 1.00 0.00

ATOM 4250 H ASP A 268 -15.758 44.911 102.344 1.00 0.00

ATOM 4251 N THR A 269 -15.336 41.967 103.676 1.00 80.29

ATOM 4252 CA THR A 269 -16.051 40.793 104.122 1.00 78.81

ATOM 4253 C THR A 269 -15.129 39.722 104.689 1.00 75.35

ATOM 4254 O THR A 269 -15.612 38.685 105.164 1.00 75.98

ATOM 4255 CB THR A 269 -16.823 40.177 102.974 1.00 80.57

ATOM 4256 OG1 THR A 269 -15.920 39.966 101.891 1.00 77.73

ATOM 4257 CG2 THR A 269 -17.995 41.099 102.546 1.00 83.03

ATOM 4258 H THR A 269 -14.888 41.971 102.748 1.00 0.00

ATOM 4259 H THR A 269 -16.722 41.131 104.911 1.00 0.00

ATOM 4260 H THR A 269 -17.255 39.225 103.283 1.00 0.00

ATOM 4261 H THR A 269 -15.171 39.350 102.239 1.00 0.00

ATOM 4262 H THR A 269 -18.507 40.602 101.722 1.00 0.00

ATOM 4263 H THR A 269 -18.643 41.215 103.415 1.00 0.00

ATOM 4264 H THR A 269 -17.557 42.048 102.237 1.00 0.00

ATOM 4265 N ILE A 270 -13.820 39.969 104.646 1.00 71.84

ATOM 4266 CA ILE A 270 -12.830 38.991 105.063 1.00 67.23

ATOM 4267 C ILE A 270 -12.069 39.433 106.336 1.00 66.45

ATOM 4268 O ILE A 270 -12.019 38.691 107.342 1.00 64.53

ATOM 4269 CB ILE A 270 -11.800 38.750 103.922 1.00 66.62

ATOM 4270 CG1 ILE A 270 -12.434 38.149 102.669 1.00 65.69

ATOM 4271 CG2 ILE A 270 -10.679 37.827 104.352 1.00 62.33

ATOM 4272 CD1 ILE A 270 -11.365 37.747 101.657 1.00 60.26

ATOM 4273 H ILE A 270 -13.499 40.886 104.305 1.00 0.00

ATOM 4274 H ILE A 270 -13.370 38.072 105.289 1.00 0.00

ATOM 4275 H ILE A 270 -11.407 39.740 103.692 1.00 0.00

ATOM 4276 H ILE A 270 -13.086 38.894 102.213 1.00 0.00

ATOM 4277 H ILE A 270 -12.998 37.261 102.953 1.00 0.00

ATOM 4278 H ILE A 270 -10.012 37.716 103.497 1.00 0.00

ATOM 4279 H ILE A 270 -10.181 38.303 105.196 1.00 0.00

ATOM 4280 H ILE A 270 -11.137 36.879 104.634 1.00 0.00

ATOM 4281 H ILE A 270 -11.884 37.329 100.794 1.00 0.00

ATOM 4282 H ILE A 270 -10.814 38.651 101.399 1.00 0.00

ATOM 4283 H ILE A 270 -10.727 37.009 102.143 1.00 0.00

ATOM 4284 N SER A 271 -11.445 40.614 106.291 1.00 66.59

ATOM 4285 CA SER A 271 -10.664 41.102 107.444 1.00 66.49

ATOM 4286 C SER A 271 -10.181 42.548 107.292 1.00 67.53

ATOM 4287 O SER A 271 -9.999 43.060 106.167 1.00 67.85

ATOM 4288 CB SER A 271 -9.480 40.158 107.748 1.00 64.84

ATOM 4289 OG SER A 271 -8.374 40.838 108.367 1.00 67.42

ATOM 4290 H SER A 271 -11.510 41.191 105.440 1.00 0.00

ATOM 4291 H SER A 271 -11.349 41.100 108.292 1.00 0.00

ATOM 4292 H SER A 271 -9.112 39.688 106.836 1.00 0.00

ATOM 4293 H SER A 271 -9.816 39.432 108.489 1.00 0.00

ATOM 4294 H SER A 271 -7.640 40.134 108.530 1.00 0.00

ATOM 4295 N SER A 272 -9.948 43.201 108.425 1.00 67.20

ATOM 4296 CA SER A 272 -9.582 44.618 108.380 1.00 68.60

ATOM 4297 C SER A 272 -8.090 44.948 108.424 1.00 68.38

ATOM 4298 O SER A 272 -7.753 46.104 108.611 1.00 69.73

ATOM 4299 CB SER A 272 -10.277 45.362 109.505 1.00 69.65

ATOM 4300 OG SER A 272 -9.893 44.795 110.718 1.00 68.00

ATOM 4301 H SER A 272 -10.024 42.714 109.329 1.00 0.00

ATOM 4302 H SER A 272 -9.912 44.940 107.393 1.00 0.00

ATOM 4303 H SER A 272 -11.364 45.353 109.424 1.00 0.00

ATOM 4304 H SER A 272 -9.937 46.398 109.525 1.00 0.00

ATOM 4305 H SER A 272 -10.378 45.318 111.462 1.00 0.00

ATOM 4306 N LYS A 273 -7.225 43.949 108.218 1.00 66.66

ATOM 4307 CA LYS A 273 -5.769 44.059 108.448 1.00 66.80

ATOM 4308 C LYS A 273 -4.924 43.973 107.192 1.00 66.19

ATOM 4309 O LYS A 273 -3.702 44.011 107.235 1.00 66.35

ATOM 4310 CB LYS A 273 -5.318 42.997 109.442 1.00 65.15

ATOM 4311 CG LYS A 273 -6.301 42.796 110.566 1.00 64.92

ATOM 4312 CD LYS A 273 -6.313 43.955 111.547 1.00 64.32

ATOM 4313 CE LYS A 273 -6.733 43.479 112.938 1.00 65.64

ATOM 4314 NZ LYS A 273 -6.449 44.491 114.004 1.00 73.55

ATOM 4315 H LYS A 273 -7.595 43.050 107.879 1.00 0.00

ATOM 4316 H LYS A 273 -5.610 45.060 108.848 1.00 0.00

ATOM 4317 H LYS A 273 -4.372 43.317 109.879 1.00 0.00

ATOM 4318 H LYS A 273 -5.237 42.051 108.907 1.00 0.00

ATOM 4319 H LYS A 273 -6.001 41.900 111.110 1.00 0.00

ATOM 4320 H LYS A 273 -7.296 42.709 110.129 1.00 0.00

ATOM 4321 H LYS A 273 -7.029 44.699 111.198 1.00 0.00

ATOM 4322 H LYS A 273 -5.311 44.382 111.602 1.00 0.00

ATOM 4323 H LYS A 273 -6.179 42.572 113.181 1.00 0.00

ATOM 4324 H LYS A 273 -7.812 43.322 112.926 1.00 0.00

ATOM 4325 H LYS A 273 -6.763 44.084 114.897 1.00 0.00

ATOM 4326 H LYS A 273 -5.433 44.659 113.996 1.00 0.00

ATOM 4327 H LYS A 273 -6.980 45.340 113.762 1.00 0.00

ATOM 4328 N LEU A 274 -5.612 43.983 106.077 1.00 66.77

ATOM 4329 CA LEU A 274 -5.108 43.492 104.814 1.00 67.79

ATOM 4330 C LEU A 274 -4.747 44.652 103.945 1.00 70.97

ATOM 4331 O LEU A 274 -4.011 44.505 102.977 1.00 71.47

ATOM 4332 CB LEU A 274 -6.226 42.659 104.134 1.00 66.13

ATOM 4333 CG LEU A 274 -6.540 41.446 105.001 1.00 65.36

ATOM 4334 CD1 LEU A 274 -7.605 40.647 104.386 1.00 68.65

ATOM 4335 CD2 LEU A 274 -5.272 40.621 105.107 1.00 64.68

ATOM 4336 H LEU A 274 -6.569 44.363 106.102 1.00 0.00

ATOM 4337 H LEU A 274 -4.224 42.874 104.971 1.00 0.00

ATOM 4338 H LEU A 274 -5.907 42.330 103.145 1.00 0.00

ATOM 4339 H LEU A 274 -7.126 43.266 104.034 1.00 0.00

ATOM 4340 H LEU A 274 -6.880 41.761 105.988 1.00 0.00

ATOM 4341 H LEU A 274 -7.787 39.798 105.045 1.00 0.00

ATOM 4342 H LEU A 274 -8.480 41.292 104.304 1.00 0.00

ATOM 4343 H LEU A 274 -7.242 40.331 103.408 1.00 0.00

ATOM 4344 H LEU A 274 -5.505 39.756 105.728 1.00 0.00

ATOM 4345 H LEU A 274 -4.996 40.329 104.094 1.00 0.00

ATOM 4346 H LEU A 274 -4.515 41.257 105.566 1.00 0.00

ATOM 4347 N LYS A 275 -5.330 45.795 104.289 1.00 74.53

ATOM 4348 CA LYS A 275 -5.139 47.037 103.584 1.00 78.61

ATOM 4349 C LYS A 275 -3.676 47.339 103.340 1.00 79.38

ATOM 4350 O LYS A 275 -3.328 47.771 102.267 1.00 80.37

ATOM 4351 CB LYS A 275 -5.802 48.195 104.336 1.00 82.01

ATOM 4352 CG LYS A 275 -7.340 48.042 104.570 1.00 84.93

ATOM 4353 CD LYS A 275 -8.210 48.798 103.534 1.00 88.22

ATOM 4354 CE LYS A 275 -9.478 49.323 104.173 1.00 90.06

ATOM 4355 NZ LYS A 275 -9.141 50.436 105.122 1.00 94.86

ATOM 4356 H LYS A 275 -5.955 45.793 105.108 1.00 0.00

ATOM 4357 H LYS A 275 -5.616 46.926 102.610 1.00 0.00

ATOM 4358 H LYS A 275 -5.667 49.074 103.706 1.00 0.00

ATOM 4359 H LYS A 275 -5.324 48.247 105.314 1.00 0.00

ATOM 4360 H LYS A 275 -7.569 48.458 105.551 1.00 0.00

ATOM 4361 H LYS A 275 -7.579 46.983 104.474 1.00 0.00

ATOM 4362 H LYS A 275 -8.481 48.125 102.720 1.00 0.00

ATOM 4363 H LYS A 275 -7.646 49.653 103.161 1.00 0.00

ATOM 4364 H LYS A 275 -9.975 48.521 104.718 1.00 0.00

ATOM 4365 H LYS A 275 -10.142 49.705 103.398 1.00 0.00

ATOM 4366 H LYS A 275 -10.028 50.761 105.532 1.00 0.00

ATOM 4367 H LYS A 275 -8.511 50.043 105.836 1.00 0.00

ATOM 4368 H LYS A 275 -8.681 51.174 104.570 1.00 0.00

ATOM 4369 N GLU A 276 -2.807 47.119 104.310 1.00 79.84

ATOM 4370 CA GLU A 276 -1.382 47.258 103.987 1.00 82.43

ATOM 4371 C GLU A 276 -0.883 46.167 103.039 1.00 80.29

ATOM 4372 O GLU A 276 -0.162 46.477 102.108 1.00 82.12

ATOM 4373 CB GLU A 276 -0.490 47.369 105.223 1.00 83.74

ATOM 4374 CG GLU A 276 1.033 47.218 104.927 1.00 87.55

ATOM 4375 CD GLU A 276 1.631 48.345 104.083 1.00 93.61

ATOM 4376 OE1 GLU A 276 1.338 49.534 104.390 1.00 97.16

ATOM 4377 OE2 GLU A 276 2.399 48.036 103.121 1.00 93.60

ATOM 4378 H GLU A 276 -3.118 46.860 105.257 1.00 0.00

ATOM 4379 H GLU A 276 -1.303 48.208 103.459 1.00 0.00

ATOM 4380 H GLU A 276 -0.773 46.547 105.880 1.00 0.00

ATOM 4381 H GLU A 276 -0.654 48.358 105.650 1.00 0.00

ATOM 4382 H GLU A 276 1.174 46.293 104.368 1.00 0.00

ATOM 4383 H GLU A 276 1.549 47.250 105.887 1.00 0.00

ATOM 4384 N CYS A 277 -1.276 44.915 103.264 1.00 77.32

ATOM 4385 CA CYS A 277 -0.905 43.807 102.358 1.00 76.54

ATOM 4386 C CYS A 277 -1.339 43.811 100.950 1.00 75.97

ATOM 4387 O CYS A 277 -0.764 43.121 100.105 1.00 74.35

ATOM 4388 CB CYS A 277 -1.701 42.637 102.971 1.00 73.56

ATOM 4389 SG CYS A 277 -0.802 41.552 104.080 1.00 75.78

ATOM 4390 H CYS A 277 -1.854 44.713 104.092 1.00 0.00

ATOM 4391 H CYS A 277 0.184 43.814 102.302 1.00 0.00

ATOM 4392 H CYS A 277 -1.887 42.042 102.077 1.00 0.00

ATOM 4393 H CYS A 277 -2.572 43.097 103.438 1.00 0.00

ATOM 4394 N CYS A 278 -2.355 44.633 100.713 1.00 77.56

ATOM 4395 CA CYS A 278 -3.138 44.537 99.508 1.00 78.28

ATOM 4396 C CYS A 278 -2.640 45.605 98.613 1.00 80.86

ATOM 4397 O CYS A 278 -3.330 46.054 97.731 1.00 83.02

ATOM 4398 CB CYS A 278 -4.646 44.526 99.667 1.00 77.96

ATOM 4399 SG CYS A 278 -5.068 42.838 100.134 1.00 77.54

ATOM 4400 H CYS A 278 -2.589 45.357 101.408 1.00 0.00

ATOM 4401 H CYS A 278 -2.993 43.545 99.080 1.00 0.00

ATOM 4402 H CYS A 278 -5.040 44.763 98.678 1.00 0.00

ATOM 4403 H CYS A 278 -4.972 45.341 100.313 1.00 0.00

ATOM 4404 N ASP A 279 -1.386 45.957 98.801 1.00 82.04

ATOM 4405 CA ASP A 279 -0.857 47.131 98.191 1.00 85.55

ATOM 4406 C ASP A 279 0.589 46.930 97.720 1.00 86.23

ATOM 4407 O ASP A 279 1.205 47.879 97.179 1.00 89.00

ATOM 4408 CB ASP A 279 -0.948 48.238 99.219 1.00 88.21

ATOM 4409 CG ASP A 279 -0.608 49.594 98.650 1.00 94.36

ATOM 4410 OD1 ASP A 279 -1.486 50.495 98.686 1.00 98.09

ATOM 4411 OD2 ASP A 279 0.540 49.764 98.184 1.00 95.54

ATOM 4412 H ASP A 279 -0.779 45.376 99.397 1.00 0.00

ATOM 4413 H ASP A 279 -1.428 47.381 97.297 1.00 0.00

ATOM 4414 H ASP A 279 -0.233 48.020 100.012 1.00 0.00

ATOM 4415 H ASP A 279 -1.977 48.277 99.577 1.00 0.00

ATOM 4416 N LYS A 280 1.137 45.719 97.905 1.00 82.45

ATOM 4417 CA LYS A 280 2.526 45.471 97.479 1.00 83.35

ATOM 4418 C LYS A 280 2.599 44.960 96.037 1.00 82.58

ATOM 4419 O LYS A 280 1.561 44.742 95.421 1.00 81.13

ATOM 4420 CB LYS A 280 3.240 44.501 98.421 1.00 81.51

ATOM 4421 CG LYS A 280 3.017 44.754 99.883 1.00 81.54

ATOM 4422 CD LYS A 280 4.347 44.967 100.560 1.00 83.25

ATOM 4423 CE LYS A 280 4.138 45.238 102.039 1.00 85.41

ATOM 4424 NZ LYS A 280 3.487 44.091 102.734 1.00 77.22

ATOM 4425 H LYS A 280 0.589 44.965 98.343 1.00 0.00

ATOM 4426 H LYS A 280 3.040 46.431 97.523 1.00 0.00

ATOM 4427 H LYS A 280 4.311 44.600 98.245 1.00 0.00

ATOM 4428 H LYS A 280 2.829 43.511 98.222 1.00 0.00

ATOM 4429 H LYS A 280 2.515 43.894 100.326 1.00 0.00

ATOM 4430 H LYS A 280 2.393 45.639 100.009 1.00 0.00

ATOM 4431 H LYS A 280 4.843 45.824 100.105 1.00 0.00

ATOM 4432 H LYS A 280 4.960 44.074 100.436 1.00 0.00

ATOM 4433 H LYS A 280 3.496 46.111 102.151 1.00 0.00

ATOM 4434 H LYS A 280 5.115 45.385 102.498 1.00 0.00

ATOM 4435 H LYS A 280 3.384 44.358 103.724 1.00 0.00

ATOM 4436 H LYS A 280 2.574 43.943 102.280 1.00 0.00

ATOM 4437 H LYS A 280 4.108 43.277 102.619 1.00 0.00

ATOM 4438 N PRO A 281 3.822 44.756 95.495 1.00 83.48

ATOM 4439 CA PRO A 281 3.903 44.010 94.228 1.00 82.61

ATOM 4440 C PRO A 281 3.554 42.547 94.481 1.00 78.91

ATOM 4441 O PRO A 281 3.469 42.115 95.645 1.00 77.56

ATOM 4442 CB PRO A 281 5.375 44.174 93.799 1.00 85.43

ATOM 4443 CG PRO A 281 6.042 45.039 94.856 1.00 87.37

ATOM 4444 CD PRO A 281 5.153 45.001 96.069 1.00 85.16

ATOM 4445 H PRO A 281 3.215 44.365 93.461 1.00 0.00

ATOM 4446 H PRO A 281 5.455 44.633 92.814 1.00 0.00

ATOM 4447 H PRO A 281 5.871 43.206 93.733 1.00 0.00

ATOM 4448 H PRO A 281 6.175 46.062 94.504 1.00 0.00

ATOM 4449 H PRO A 281 7.035 44.660 95.099 1.00 0.00

ATOM 4450 H PRO A 281 5.440 44.225 96.779 1.00 0.00

ATOM 4451 H PRO A 281 5.193 45.909 96.670 1.00 0.00

ATOM 4452 N LEU A 282 3.331 41.781 93.423 1.00 78.15

ATOM 4453 CA LEU A 282 2.670 40.459 93.573 1.00 74.49

ATOM 4454 C LEU A 282 3.306 39.509 94.608 1.00 72.21

ATOM 4455 O LEU A 282 2.637 39.031 95.524 1.00 69.87

ATOM 4456 CB LEU A 282 2.520 39.781 92.205 1.00 75.05

ATOM 4457 CG LEU A 282 1.480 38.651 91.998 1.00 74.77

ATOM 4458 CD1 LEU A 282 0.575 38.961 90.804 1.00 79.36

ATOM 4459 CD2 LEU A 282 2.109 37.277 91.817 1.00 75.55

ATOM 4460 H LEU A 282 3.618 42.105 92.488 1.00 0.00

ATOM 4461 H LEU A 282 1.686 40.677 93.989 1.00 0.00

ATOM 4462 H LEU A 282 3.482 39.293 92.050 1.00 0.00

ATOM 4463 H LEU A 282 2.194 40.583 91.542 1.00 0.00

ATOM 4464 H LEU A 282 0.890 38.616 92.914 1.00 0.00

ATOM 4465 H LEU A 282 -0.128 38.133 90.714 1.00 0.00

ATOM 4466 H LEU A 282 0.069 39.901 91.023 1.00 0.00

ATOM 4467 H LEU A 282 1.219 39.043 89.929 1.00 0.00

ATOM 4468 H LEU A 282 1.292 36.568 91.681 1.00 0.00

ATOM 4469 H LEU A 282 2.748 37.333 90.936 1.00 0.00

ATOM 4470 H LEU A 282 2.682 37.067 92.720 1.00 0.00

ATOM 4471 N LEU A 283 4.596 39.225 94.478 1.00 72.63

ATOM 4472 CA LEU A 283 5.183 38.309 95.425 1.00 71.20

ATOM 4473 C LEU A 283 4.951 38.727 96.886 1.00 71.23

ATOM 4474 O LEU A 283 4.594 37.882 97.720 1.00 70.07

ATOM 4475 CB LEU A 283 6.661 38.059 95.125 1.00 72.84

ATOM 4476 CG LEU A 283 6.860 37.228 93.845 1.00 72.94

ATOM 4477 CD1 LEU A 283 8.325 37.187 93.396 1.00 70.80

ATOM 4478 CD2 LEU A 283 6.243 35.796 93.949 1.00 68.87

ATOM 4479 H LEU A 283 5.160 39.645 93.725 1.00 0.00

ATOM 4480 H LEU A 283 4.661 37.360 95.300 1.00 0.00

ATOM 4481 H LEU A 283 7.083 37.501 95.961 1.00 0.00

ATOM 4482 H LEU A 283 7.148 39.025 94.988 1.00 0.00

ATOM 4483 H LEU A 283 6.304 37.748 93.065 1.00 0.00

ATOM 4484 H LEU A 283 8.364 36.581 92.491 1.00 0.00

ATOM 4485 H LEU A 283 8.630 38.217 93.208 1.00 0.00

ATOM 4486 H LEU A 283 8.895 36.738 94.209 1.00 0.00

ATOM 4487 H LEU A 283 6.437 35.296 93.000 1.00 0.00

ATOM 4488 H LEU A 283 6.739 35.295 94.780 1.00 0.00

ATOM 4489 H LEU A 283 5.176 35.921 94.130 1.00 0.00

ATOM 4490 N GLU A 284 5.118 40.018 97.182 1.00 72.92

ATOM 4491 CA GLU A 284 5.182 40.476 98.552 1.00 73.73

ATOM 4492 C GLU A 284 3.825 40.496 99.206 1.00 72.07

ATOM 4493 O GLU A 284 3.733 40.348 100.430 1.00 73.03

ATOM 4494 CB GLU A 284 5.829 41.846 98.668 1.00 77.51

ATOM 4495 CG GLU A 284 7.241 41.964 98.054 1.00 82.14

ATOM 4496 CD GLU A 284 7.778 43.390 98.112 1.00 90.85

ATOM 4497 OE1 GLU A 284 7.458 44.133 99.075 1.00 93.59

ATOM 4498 OE2 GLU A 284 8.525 43.779 97.184 1.00 96.78

ATOM 4499 H GLU A 284 5.203 40.703 96.418 1.00 0.00

ATOM 4500 H GLU A 284 5.808 39.755 99.079 1.00 0.00

ATOM 4501 H GLU A 284 5.937 42.037 99.736 1.00 0.00

ATOM 4502 H GLU A 284 5.183 42.542 98.133 1.00 0.00

ATOM 4503 H GLU A 284 7.192 41.663 97.008 1.00 0.00

ATOM 4504 H GLU A 284 7.916 41.334 98.633 1.00 0.00

ATOM 4505 N LYS A 285 2.781 40.668 98.403 1.00 70.59

ATOM 4506 CA LYS A 285 1.402 40.747 98.862 1.00 68.15

ATOM 4507 C LYS A 285 0.972 39.382 99.393 1.00 65.91

ATOM 4508 O LYS A 285 0.444 39.228 100.517 1.00 64.57

ATOM 4509 CB LYS A 285 0.540 41.197 97.682 1.00 68.31

ATOM 4510 CG LYS A 285 -0.989 41.080 97.827 1.00 67.59

ATOM 4511 CD LYS A 285 -1.761 41.956 96.764 1.00 68.04

ATOM 4512 CE LYS A 285 -1.727 41.348 95.367 1.00 69.79

ATOM 4513 NZ LYS A 285 -2.106 42.251 94.203 1.00 71.33

ATOM 4514 H LYS A 285 2.958 40.751 97.392 1.00 0.00

ATOM 4515 H LYS A 285 1.290 41.466 99.674 1.00 0.00

ATOM 4516 H LYS A 285 0.807 40.530 96.862 1.00 0.00

ATOM 4517 H LYS A 285 0.747 42.260 97.558 1.00 0.00

ATOM 4518 H LYS A 285 -1.245 41.450 98.820 1.00 0.00

ATOM 4519 H LYS A 285 -1.262 40.033 97.693 1.00 0.00

ATOM 4520 H LYS A 285 -1.305 42.945 96.710 1.00 0.00

ATOM 4521 H LYS A 285 -2.810 41.993 97.059 1.00 0.00

ATOM 4522 H LYS A 285 -2.468 40.549 95.374 1.00 0.00

ATOM 4523 H LYS A 285 -0.689 41.066 95.193 1.00 0.00

ATOM 4524 H LYS A 285 -2.029 41.685 93.346 1.00 0.00

ATOM 4525 H LYS A 285 -3.072 42.566 94.369 1.00 0.00

ATOM 4526 H LYS A 285 -1.441 43.038 94.203 1.00 0.00

ATOM 4527 N SER A 286 1.198 38.379 98.563 1.00 65.05

ATOM 4528 CA SER A 286 0.976 36.990 98.943 1.00 63.69

ATOM 4529 C SER A 286 1.721 36.564 100.215 1.00 63.49

ATOM 4530 O SER A 286 1.145 35.958 101.099 1.00 62.08

ATOM 4531 CB SER A 286 1.359 36.121 97.761 1.00 63.31

ATOM 4532 OG SER A 286 0.591 36.587 96.648 1.00 66.80

ATOM 4533 H SER A 286 1.543 38.586 97.615 1.00 0.00

ATOM 4534 H SER A 286 -0.078 36.869 99.193 1.00 0.00

ATOM 4535 H SER A 286 1.188 35.058 97.933 1.00 0.00

ATOM 4536 H SER A 286 2.418 36.230 97.526 1.00 0.00

ATOM 4537 H SER A 286 0.846 36.001 95.840 1.00 0.00

ATOM 4538 N HIS A 287 3.002 36.899 100.307 1.00 65.48

ATOM 4539 CA HIS A 287 3.734 36.664 101.526 1.00 66.31

ATOM 4540 C HIS A 287 3.153 37.442 102.716 1.00 67.17

ATOM 4541 O HIS A 287 3.018 36.892 103.795 1.00 66.47

ATOM 4542 CB HIS A 287 5.207 37.005 101.345 1.00 68.89

ATOM 4543 CG HIS A 287 6.003 36.768 102.580 1.00 70.35

ATOM 4544 ND1 HIS A 287 6.515 35.523 102.908 1.00 72.96

ATOM 4545 CD2 HIS A 287 6.325 37.592 103.599 1.00 68.07

ATOM 4546 CE1 HIS A 287 7.159 35.606 104.060 1.00 72.30

ATOM 4547 NE2 HIS A 287 7.061 36.852 104.496 1.00 73.65

ATOM 4548 H HIS A 287 3.477 37.330 99.501 1.00 0.00

ATOM 4549 H HIS A 287 3.638 35.602 101.752 1.00 0.00

ATOM 4550 H HIS A 287 5.296 38.060 101.085 1.00 0.00

ATOM 4551 H HIS A 287 5.611 36.349 100.574 1.00 0.00

ATOM 4552 H HIS A 287 6.410 34.668 102.344 1.00 0.00

ATOM 4553 H HIS A 287 6.054 38.643 103.694 1.00 0.00

ATOM 4554 H HIS A 287 7.680 34.791 104.563 1.00 0.00

ATOM 4555 N CYS A 288 2.841 38.725 102.504 1.00 68.67

ATOM 4556 CA CYS A 288 2.135 39.550 103.472 1.00 70.36

ATOM 4557 C CYS A 288 0.841 38.887 103.945 1.00 68.47

ATOM 4558 O CYS A 288 0.626 38.761 105.142 1.00 68.42

ATOM 4559 CB CYS A 288 1.812 40.906 102.864 1.00 71.72

ATOM 4560 SG CYS A 288 1.120 42.139 103.999 1.00 78.94

ATOM 4561 H CYS A 288 3.115 39.152 101.608 1.00 0.00

ATOM 4562 H CYS A 288 2.789 39.675 104.335 1.00 0.00

ATOM 4563 H CYS A 288 0.985 40.621 102.213 1.00 0.00

ATOM 4564 H CYS A 288 2.727 41.219 102.362 1.00 0.00

ATOM 4565 N ILE A 289 -0.010 38.451 103.007 1.00 67.84

ATOM 4566 CA ILE A 289 -1.308 37.880 103.369 1.00 66.61

ATOM 4567 C ILE A 289 -1.116 36.590 104.175 1.00 66.88

ATOM 4568 O ILE A 289 -1.908 36.309 105.093 1.00 66.71

ATOM 4569 CB ILE A 289 -2.204 37.617 102.150 1.00 65.72

ATOM 4570 CG1 ILE A 289 -2.325 38.867 101.279 1.00 65.10

ATOM 4571 CG2 ILE A 289 -3.602 37.217 102.581 1.00 65.37

ATOM 4572 CD1 ILE A 289 -2.648 38.524 99.862 1.00 66.99

ATOM 4573 H ILE A 289 0.254 38.519 102.014 1.00 0.00

ATOM 4574 H ILE A 289 -1.819 38.622 103.983 1.00 0.00

ATOM 4575 H ILE A 289 -1.739 36.809 101.584 1.00 0.00

ATOM 4576 H ILE A 289 -1.380 39.410 101.294 1.00 0.00

ATOM 4577 H ILE A 289 -3.145 39.474 101.663 1.00 0.00

ATOM 4578 H ILE A 289 -4.179 37.047 101.672 1.00 0.00

ATOM 4579 H ILE A 289 -3.505 36.308 103.175 1.00 0.00

ATOM 4580 H ILE A 289 -4.003 38.043 103.167 1.00 0.00

ATOM 4581 H ILE A 289 -2.716 39.464 99.314 1.00 0.00

ATOM 4582 H ILE A 289 -1.836 37.897 99.493 1.00 0.00

ATOM 4583 H ILE A 289 -3.599 37.991 99.872 1.00 0.00

ATOM 4584 N ALA A 290 -0.061 35.823 103.865 1.00 66.69

ATOM 4585 CA ALA A 290 0.191 34.575 104.624 1.00 66.39

ATOM 4586 C ALA A 290 0.781 34.837 106.020 1.00 67.59

ATOM 4587 O ALA A 290 0.549 34.057 106.910 1.00 67.58

ATOM 4588 CB ALA A 290 1.050 33.578 103.816 1.00 66.11

ATOM 4589 H ALA A 290 0.573 36.100 103.102 1.00 0.00

ATOM 4590 H ALA A 290 -0.782 34.111 104.785 1.00 0.00

ATOM 4591 H ALA A 290 1.187 32.696 104.441 1.00 0.00

ATOM 4592 H ALA A 290 0.498 33.347 102.905 1.00 0.00

ATOM 4593 H ALA A 290 1.997 34.074 103.602 1.00 0.00

ATOM 4594 N GLU A 291 1.521 35.949 106.175 1.00 69.18

ATOM 4595 CA GLU A 291 2.109 36.458 107.460 1.00 71.13

ATOM 4596 C GLU A 291 1.205 37.351 108.387 1.00 70.99

ATOM 4597 O GLU A 291 1.601 37.707 109.494 1.00 72.65

ATOM 4598 CB GLU A 291 3.317 37.326 107.126 1.00 73.50

ATOM 4599 CG GLU A 291 4.746 36.767 107.263 1.00 76.57

ATOM 4600 CD GLU A 291 5.750 37.954 107.378 1.00 86.87

ATOM 4601 OE1 GLU A 291 7.013 37.784 107.221 1.00 89.29

ATOM 4602 OE2 GLU A 291 5.246 39.098 107.635 1.00 88.83

ATOM 4603 H GLU A 291 1.703 36.507 105.329 1.00 0.00

ATOM 4604 H GLU A 291 2.308 35.542 108.017 1.00 0.00

ATOM 4605 H GLU A 291 3.272 38.128 107.863 1.00 0.00

ATOM 4606 H GLU A 291 3.206 37.542 106.063 1.00 0.00

ATOM 4607 H GLU A 291 4.983 36.171 106.382 1.00 0.00

ATOM 4608 H GLU A 291 4.809 36.135 108.149 1.00 0.00

ATOM 4609 N VAL A 292 -0.003 37.687 107.936 1.00 69.51

ATOM 4610 CA VAL A 292 -0.844 38.743 108.508 1.00 68.50

ATOM 4611 C VAL A 292 -1.492 38.499 109.885 1.00 69.06

ATOM 4612 O VAL A 292 -1.903 37.397 110.209 1.00 67.85

ATOM 4613 CB VAL A 292 -1.947 39.091 107.498 1.00 67.35

ATOM 4614 CG1 VAL A 292 -3.231 38.264 107.752 1.00 64.89

ATOM 4615 CG2 VAL A 292 -2.200 40.589 107.471 1.00 66.88

ATOM 4616 H VAL A 292 -0.373 37.167 107.127 1.00 0.00

ATOM 4617 H VAL A 292 -0.141 39.554 108.700 1.00 0.00

ATOM 4618 H VAL A 292 -1.603 38.812 106.502 1.00 0.00

ATOM 4619 H VAL A 292 -3.959 38.567 107.000 1.00 0.00

ATOM 4620 H VAL A 292 -2.958 37.213 107.652 1.00 0.00

ATOM 4621 H VAL A 292 -3.568 38.501 108.761 1.00 0.00

ATOM 4622 H VAL A 292 -2.988 40.766 106.739 1.00 0.00

ATOM 4623 H VAL A 292 -2.508 40.878 108.476 1.00 0.00

ATOM 4624 H VAL A 292 -1.263 41.065 107.181 1.00 0.00

ATOM 4625 N GLU A 293 -1.604 39.573 110.666 1.00 71.03

ATOM 4626 CA GLU A 293 -2.136 39.538 112.009 1.00 72.16

ATOM 4627 C GLU A 293 -3.580 39.131 112.004 1.00 69.82

ATOM 4628 O GLU A 293 -4.273 39.242 111.011 1.00 69.14

ATOM 4629 CB GLU A 293 -1.969 40.894 112.724 1.00 75.29

ATOM 4630 CG GLU A 293 -2.946 41.097 113.930 1.00 79.72

ATOM 4631 CD GLU A 293 -2.322 41.673 115.240 1.00 87.69

ATOM 4632 OE1 GLU A 293 -2.297 40.934 116.266 1.00 88.56

ATOM 4633 OE2 GLU A 293 -1.894 42.860 115.259 1.00 91.80

ATOM 4634 H GLU A 293 -1.293 40.480 110.291 1.00 0.00

ATOM 4635 H GLU A 293 -1.563 38.793 112.562 1.00 0.00

ATOM 4636 H GLU A 293 -2.196 41.666 111.989 1.00 0.00

ATOM 4637 H GLU A 293 -0.949 40.936 113.106 1.00 0.00

ATOM 4638 H GLU A 293 -3.325 40.107 114.185 1.00 0.00

ATOM 4639 H GLU A 293 -3.684 41.829 113.602 1.00 0.00

ATOM 4640 N LYS A 294 -4.028 38.679 113.149 1.00 69.76

ATOM 4641 CA LYS A 294 -5.318 38.089 113.251 1.00 68.50

ATOM 4642 C LYS A 294 -6.371 39.164 113.522 1.00 68.87

ATOM 4643 O LYS A 294 -6.078 40.230 114.019 1.00 70.09

ATOM 4644 CB LYS A 294 -5.246 36.968 114.294 1.00 69.46

ATOM 4645 CG LYS A 294 -4.115 35.957 113.940 1.00 69.53

ATOM 4646 CD LYS A 294 -4.328 34.562 114.510 1.00 70.90

ATOM 4647 CE LYS A 294 -2.966 33.886 114.815 1.00 75.43

ATOM 4648 NZ LYS A 294 -2.116 33.529 113.608 1.00 73.73

ATOM 4649 H LYS A 294 -3.436 38.752 113.989 1.00 0.00

ATOM 4650 H LYS A 294 -5.634 37.629 112.315 1.00 0.00

ATOM 4651 H LYS A 294 -6.201 36.442 114.295 1.00 0.00

ATOM 4652 H LYS A 294 -5.048 37.409 115.271 1.00 0.00

ATOM 4653 H LYS A 294 -3.179 36.334 114.353 1.00 0.00

ATOM 4654 H LYS A 294 -4.110 35.846 112.856 1.00 0.00

ATOM 4655 H LYS A 294 -4.859 33.968 113.766 1.00 0.00

ATOM 4656 H LYS A 294 -4.914 34.640 115.426 1.00 0.00

ATOM 4657 H LYS A 294 -3.179 32.948 115.328 1.00 0.00

ATOM 4658 H LYS A 294 -2.381 34.615 115.376 1.00 0.00

ATOM 4659 H LYS A 294 -1.254 33.093 113.965 1.00 0.00

ATOM 4660 H LYS A 294 -2.667 32.874 113.035 1.00 0.00

ATOM 4661 H LYS A 294 -1.922 34.407 113.106 1.00 0.00

ATOM 4662 N ASP A 295 -7.606 38.911 113.142 1.00 68.33

ATOM 4663 CA ASP A 295 -8.622 39.953 113.271 1.00 69.37

ATOM 4664 C ASP A 295 -9.341 39.770 114.636 1.00 70.15

ATOM 4665 O ASP A 295 -9.204 38.694 115.281 1.00 69.89

ATOM 4666 CB ASP A 295 -9.563 39.912 112.027 1.00 68.43

ATOM 4667 CG ASP A 295 -10.358 41.229 111.826 1.00 71.68

ATOM 4668 OD1 ASP A 295 -9.937 42.262 112.403 1.00 74.53

ATOM 4669 OD2 ASP A 295 -11.387 41.242 111.089 1.00 69.04

ATOM 4670 H ASP A 295 -7.853 37.988 112.758 1.00 0.00

ATOM 4671 H ASP A 295 -8.190 40.954 113.279 1.00 0.00

ATOM 4672 H ASP A 295 -10.284 39.108 112.176 1.00 0.00

ATOM 4673 H ASP A 295 -8.941 39.766 111.144 1.00 0.00

ATOM 4674 N ALA A 296 -10.111 40.771 115.081 1.00 74.26

ATOM 4675 CA ALA A 296 -10.849 40.622 116.364 1.00 75.60

ATOM 4676 C ALA A 296 -12.076 39.715 116.296 1.00 74.58

ATOM 4677 O ALA A 296 -12.805 39.675 115.327 1.00 73.40

ATOM 4678 CB ALA A 296 -11.234 41.974 116.970 1.00 78.71

ATOM 4679 H ALA A 296 -10.192 41.642 114.537 1.00 0.00

ATOM 4680 H ALA A 296 -10.134 40.123 117.018 1.00 0.00

ATOM 4681 H ALA A 296 -11.766 41.767 117.899 1.00 0.00

ATOM 4682 H ALA A 296 -10.307 42.520 117.147 1.00 0.00

ATOM 4683 H ALA A 296 -11.870 42.480 116.243 1.00 0.00

ATOM 4684 N ILE A 297 -12.265 38.964 117.361 1.00 75.67

ATOM 4685 CA ILE A 297 -13.429 38.123 117.545 1.00 75.36

ATOM 4686 C ILE A 297 -14.668 39.024 117.583 1.00 77.61

ATOM 4687 O ILE A 297 -14.764 39.883 118.450 1.00 78.68

ATOM 4688 CB ILE A 297 -13.347 37.394 118.922 1.00 76.40

ATOM 4689 CG1 ILE A 297 -11.910 36.962 119.285 1.00 75.80

ATOM 4690 CG2 ILE A 297 -14.411 36.311 119.057 1.00 75.83

ATOM 4691 CD1 ILE A 297 -11.254 36.030 118.389 1.00 71.38

ATOM 4692 H ILE A 297 -11.548 38.975 118.101 1.00 0.00

ATOM 4693 H ILE A 297 -13.479 37.395 116.735 1.00 0.00

ATOM 4694 H ILE A 297 -13.597 38.119 119.697 1.00 0.00

ATOM 4695 H ILE A 297 -11.930 36.470 120.257 1.00 0.00

ATOM 4696 H ILE A 297 -11.301 37.862 119.196 1.00 0.00

ATOM 4697 H ILE A 297 -14.281 35.858 120.040 1.00 0.00

ATOM 4698 H ILE A 297 -15.379 36.802 118.962 1.00 0.00

ATOM 4699 H ILE A 297 -14.239 35.594 118.254 1.00 0.00

ATOM 4700 H ILE A 297 -10.261 35.841 118.797 1.00 0.00

ATOM 4701 H ILE A 297 -11.863 35.126 118.368 1.00 0.00

ATOM 4702 H ILE A 297 -11.205 36.509 117.411 1.00 0.00

ATOM 4703 N PRO A 298 -15.618 38.835 116.630 1.00 77.99

ATOM 4704 CA PRO A 298 -16.923 39.525 116.764 1.00 80.41

ATOM 4705 C PRO A 298 -17.471 39.372 118.203 1.00 83.66

ATOM 4706 O PRO A 298 -17.208 38.367 118.884 1.00 82.25

ATOM 4707 CB PRO A 298 -17.806 38.806 115.739 1.00 79.26

ATOM 4708 CG PRO A 298 -16.836 38.331 114.675 1.00 74.80

ATOM 4709 CD PRO A 298 -15.576 37.950 115.437 1.00 74.45

ATOM 4710 H PRO A 298 -16.871 40.599 116.587 1.00 0.00

ATOM 4711 H PRO A 298 -18.580 39.454 115.328 1.00 0.00

ATOM 4712 H PRO A 298 -18.356 37.976 116.182 1.00 0.00

ATOM 4713 H PRO A 298 -16.642 39.105 113.933 1.00 0.00

ATOM 4714 H PRO A 298 -17.237 37.492 114.107 1.00 0.00

ATOM 4715 H PRO A 298 -15.557 36.896 115.716 1.00 0.00

ATOM 4716 H PRO A 298 -14.662 38.072 114.856 1.00 0.00

ATOM 4717 N GLU A 299 -18.220 40.376 118.658 1.00 87.48

ATOM 4718 CA GLU A 299 -18.393 40.645 120.070 1.00 90.27

ATOM 4719 C GLU A 299 -19.487 39.822 120.758 1.00 92.70

ATOM 4720 O GLU A 299 -19.424 39.570 121.980 1.00 94.72

ATOM 4721 CB GLU A 299 -18.724 42.111 120.218 1.00 94.04

ATOM 4722 CG GLU A 299 -19.785 42.589 119.209 1.00 95.25

ATOM 4723 CD GLU A 299 -19.193 43.162 117.910 1.00 92.48

ATOM 4724 OE1 GLU A 299 -18.445 42.441 117.208 1.00 85.09

ATOM 4725 OE2 GLU A 299 -19.490 44.343 117.599 1.00 94.48

ATOM 4726 H GLU A 299 -18.695 40.986 117.977 1.00 0.00

ATOM 4727 H GLU A 299 -17.462 40.361 120.560 1.00 0.00

ATOM 4728 H GLU A 299 -17.806 42.666 120.025 1.00 0.00

ATOM 4729 H GLU A 299 -19.107 42.266 121.227 1.00 0.00

ATOM 4730 H GLU A 299 -20.353 43.387 119.687 1.00 0.00

ATOM 4731 H GLU A 299 -20.387 41.723 118.936 1.00 0.00

ATOM 4732 N ASN A 300 -20.516 39.442 120.007 1.00 92.26

ATOM 4733 CA ASN A 300 -21.675 38.827 120.632 1.00 94.51

ATOM 4734 C ASN A 300 -21.821 37.444 120.075 1.00 91.38

ATOM 4735 O ASN A 300 -22.864 37.081 119.544 1.00 91.88

ATOM 4736 CB ASN A 300 -22.933 39.663 120.396 1.00 97.97

ATOM 4737 CG ASN A 300 -22.955 40.951 121.231 1.00102.79

ATOM 4738 OD1 ASN A 300 -22.537 40.971 122.394 1.00103.07

ATOM 4739 ND2 ASN A 300 -23.470 42.027 120.639 1.00104.67

ATOM 4740 H ASN A 300 -20.492 39.583 118.987 1.00 0.00

ATOM 4741 H ASN A 300 -21.536 38.774 121.712 1.00 0.00

ATOM 4742 H ASN A 300 -23.792 39.059 120.690 1.00 0.00

ATOM 4743 H ASN A 300 -22.955 39.943 119.343 1.00 0.00

ATOM 4744 H ASN A 300 -23.514 42.922 121.146 1.00 0.00

ATOM 4745 H ASN A 300 -23.825 41.964 119.674 1.00 0.00

ATOM 4746 N LEU A 301 -20.746 36.674 120.186 1.00 88.26

ATOM 4747 CA LEU A 301 -20.733 35.376 119.580 1.00 85.06

ATOM 4748 C LEU A 301 -21.528 34.403 120.382 1.00 85.65

ATOM 4749 O LEU A 301 -21.301 34.265 121.574 1.00 87.15

ATOM 4750 CB LEU A 301 -19.314 34.854 119.349 1.00 82.42

ATOM 4751 CG LEU A 301 -18.678 35.374 118.065 1.00 80.73

ATOM 4752 CD1 LEU A 301 -17.508 36.145 118.548 1.00 83.19

ATOM 4753 CD2 LEU A 301 -18.207 34.283 117.130 1.00 75.43

ATOM 4754 H LEU A 301 -19.923 37.009 120.707 1.00 0.00

ATOM 4755 H LEU A 301 -21.199 35.482 118.600 1.00 0.00

ATOM 4756 H LEU A 301 -19.370 33.768 119.271 1.00 0.00

ATOM 4757 H LEU A 301 -18.698 35.198 120.180 1.00 0.00

ATOM 4758 H LEU A 301 -19.400 35.947 117.484 1.00 0.00

ATOM 4759 H LEU A 301 -17.006 36.547 117.668 1.00 0.00

ATOM 4760 H LEU A 301 -17.892 36.936 119.192 1.00 0.00

ATOM 4761 H LEU A 301 -16.872 35.449 119.095 1.00 0.00

ATOM 4762 H LEU A 301 -17.776 34.775 116.258 1.00 0.00

ATOM 4763 H LEU A 301 -17.465 33.697 117.672 1.00 0.00

ATOM 4764 H LEU A 301 -19.082 33.688 116.868 1.00 0.00

ATOM 4765 N PRO A 302 -22.456 33.706 119.706 1.00 85.01

ATOM 4766 CA PRO A 302 -23.360 32.699 120.270 1.00 86.52

ATOM 4767 C PRO A 302 -22.577 31.498 120.787 1.00 85.00

ATOM 4768 O PRO A 302 -21.867 30.854 119.978 1.00 82.56

ATOM 4769 CB PRO A 302 -24.222 32.303 119.059 1.00 85.65

ATOM 4770 CG PRO A 302 -23.335 32.622 117.841 1.00 82.10

ATOM 4771 CD PRO A 302 -22.655 33.881 118.254 1.00 82.79

ATOM 4772 H PRO A 302 -23.940 33.064 121.118 1.00 0.00

ATOM 4773 H PRO A 302 -25.166 32.847 119.033 1.00 0.00

ATOM 4774 H PRO A 302 -24.517 31.254 119.090 1.00 0.00

ATOM 4775 H PRO A 302 -23.881 32.721 116.903 1.00 0.00

ATOM 4776 H PRO A 302 -22.619 31.831 117.618 1.00 0.00

ATOM 4777 H PRO A 302 -21.715 34.040 117.726 1.00 0.00

ATOM 4778 H PRO A 302 -23.235 34.773 118.017 1.00 0.00

ATOM 4779 N PRO A 303 -22.716 31.163 122.091 1.00 87.22

ATOM 4780 CA PRO A 303 -21.842 30.139 122.698 1.00 86.34

ATOM 4781 C PRO A 303 -21.796 28.828 121.889 1.00 83.90

ATOM 4782 O PRO A 303 -22.854 28.327 121.471 1.00 84.06

ATOM 4783 CB PRO A 303 -22.461 29.906 124.096 1.00 90.56

ATOM 4784 CG PRO A 303 -23.897 30.370 124.005 1.00 92.44

ATOM 4785 CD PRO A 303 -23.916 31.435 122.915 1.00 92.26

ATOM 4786 H PRO A 303 -20.804 30.471 122.732 1.00 0.00

ATOM 4787 H PRO A 303 -21.923 30.445 124.876 1.00 0.00

ATOM 4788 H PRO A 303 -22.422 28.852 124.372 1.00 0.00

ATOM 4789 H PRO A 303 -24.242 30.770 124.959 1.00 0.00

ATOM 4790 H PRO A 303 -24.569 29.543 123.774 1.00 0.00

ATOM 4791 H PRO A 303 -24.820 31.375 122.309 1.00 0.00

ATOM 4792 H PRO A 303 -23.910 32.449 123.314 1.00 0.00

ATOM 4793 N LEU A 304 -20.581 28.314 121.649 1.00 81.21

ATOM 4794 CA LEU A 304 -20.345 27.012 120.949 1.00 79.43

ATOM 4795 C LEU A 304 -21.379 25.899 121.198 1.00 80.61

ATOM 4796 O LEU A 304 -21.919 25.322 120.254 1.00 80.30

ATOM 4797 CB LEU A 304 -18.908 26.469 121.226 1.00 77.73

ATOM 4798 CG LEU A 304 -17.785 27.310 120.556 1.00 76.49

ATOM 4799 CD1 LEU A 304 -16.333 26.861 120.887 1.00 74.75

ATOM 4800 CD2 LEU A 304 -18.006 27.446 119.045 1.00 70.51

ATOM 4801 H LEU A 304 -19.759 28.848 121.966 1.00 0.00

ATOM 4802 H LEU A 304 -20.464 27.274 119.898 1.00 0.00

ATOM 4803 H LEU A 304 -18.851 25.461 120.815 1.00 0.00

ATOM 4804 H LEU A 304 -18.745 26.511 122.303 1.00 0.00

ATOM 4805 H LEU A 304 -17.874 28.296 121.011 1.00 0.00

ATOM 4806 H LEU A 304 -15.662 27.534 120.354 1.00 0.00

ATOM 4807 H LEU A 304 -16.213 26.941 121.967 1.00 0.00

ATOM 4808 H LEU A 304 -16.232 25.832 120.543 1.00 0.00

ATOM 4809 H LEU A 304 -17.182 28.044 118.655 1.00 0.00

ATOM 4810 H LEU A 304 -18.006 26.438 118.631 1.00 0.00

ATOM 4811 H LEU A 304 -18.967 27.941 118.907 1.00 0.00

ATOM 4812 N THR A 305 -21.645 25.611 122.463 1.00 82.64

ATOM 4813 CA THR A 305 -22.643 24.625 122.851 1.00 84.74

ATOM 4814 C THR A 305 -23.968 24.684 122.066 1.00 85.88

ATOM 4815 O THR A 305 -24.628 23.641 121.886 1.00 86.47

ATOM 4816 CB THR A 305 -22.955 24.706 124.372 1.00 87.81

ATOM 4817 OG1 THR A 305 -22.285 25.849 124.926 1.00 89.73

ATOM 4818 CG2 THR A 305 -22.473 23.453 125.088 1.00 86.39

ATOM 4819 H THR A 305 -21.125 26.105 123.202 1.00 0.00

ATOM 4820 H THR A 305 -22.178 23.672 122.600 1.00 0.00

ATOM 4821 H THR A 305 -24.033 24.793 124.506 1.00 0.00

ATOM 4822 H THR A 305 -22.633 26.676 124.421 1.00 0.00

ATOM 4823 H THR A 305 -22.721 23.573 126.143 1.00 0.00

ATOM 4824 H THR A 305 -22.999 22.609 124.642 1.00 0.00

ATOM 4825 H THR A 305 -21.397 23.389 124.929 1.00 0.00

ATOM 4826 N ALA A 306 -24.355 25.883 121.616 1.00 86.04

ATOM 4827 CA ALA A 306 -25.614 26.071 120.889 1.00 87.00

ATOM 4828 C ALA A 306 -25.965 25.121 119.733 1.00 85.66

ATOM 4829 O ALA A 306 -27.044 24.470 119.733 1.00 87.42

ATOM 4830 CB ALA A 306 -25.679 27.474 120.284 1.00 86.81

ATOM 4831 H ALA A 306 -23.750 26.699 121.786 1.00 0.00

ATOM 4832 H ALA A 306 -26.331 25.863 121.683 1.00 0.00

ATOM 4833 H ALA A 306 -26.632 27.551 119.760 1.00 0.00

ATOM 4834 H ALA A 306 -25.613 28.181 121.111 1.00 0.00

ATOM 4835 H ALA A 306 -24.833 27.567 119.603 1.00 0.00

ATOM 4836 N ASP A 307 -25.035 25.015 118.786 1.00 81.92

ATOM 4837 CA ASP A 307 -25.302 24.319 117.534 1.00 80.61

ATOM 4838 C ASP A 307 -24.844 22.890 117.666 1.00 79.03

ATOM 4839 O ASP A 307 -25.372 22.021 116.974 1.00 78.48

ATOM 4840 CB ASP A 307 -24.599 24.926 116.314 1.00 78.68

ATOM 4841 CG ASP A 307 -25.387 26.065 115.659 1.00 81.07

ATOM 4842 OD1 ASP A 307 -25.210 26.249 114.445 1.00 80.11

ATOM 4843 OD2 ASP A 307 -26.178 26.773 116.332 1.00 87.45

ATOM 4844 H ASP A 307 -24.107 25.434 118.942 1.00 0.00

ATOM 4845 H ASP A 307 -26.375 24.404 117.362 1.00 0.00

ATOM 4846 H ASP A 307 -24.488 24.135 115.572 1.00 0.00

ATOM 4847 H ASP A 307 -23.649 25.339 116.655 1.00 0.00

ATOM 4848 N PHE A 308 -23.861 22.658 118.539 1.00 78.06

ATOM 4849 CA PHE A 308 -23.070 21.430 118.484 1.00 76.41

ATOM 4850 C PHE A 308 -23.283 20.453 119.619 1.00 78.02

ATOM 4851 O PHE A 308 -22.761 19.349 119.598 1.00 78.48

ATOM 4852 CB PHE A 308 -21.602 21.770 118.389 1.00 74.14

ATOM 4853 CG PHE A 308 -21.273 22.619 117.208 1.00 72.90

ATOM 4854 CD1 PHE A 308 -21.244 22.068 115.922 1.00 70.03

ATOM 4855 CD2 PHE A 308 -20.991 23.988 117.367 1.00 70.70

ATOM 4856 CE1 PHE A 308 -20.970 22.858 114.829 1.00 64.22

ATOM 4857 CE2 PHE A 308 -20.714 24.761 116.270 1.00 65.08

ATOM 4858 CZ PHE A 308 -20.729 24.188 114.997 1.00 61.67

ATOM 4859 H PHE A 308 -23.656 23.358 119.266 1.00 0.00

ATOM 4860 H PHE A 308 -23.427 20.914 117.593 1.00 0.00

ATOM 4861 H PHE A 308 -21.042 20.839 118.294 1.00 0.00

ATOM 4862 H PHE A 308 -21.336 22.340 119.279 1.00 0.00

ATOM 4863 H PHE A 308 -21.440 21.005 115.785 1.00 0.00

ATOM 4864 H PHE A 308 -20.993 24.433 118.362 1.00 0.00

ATOM 4865 H PHE A 308 -20.946 22.420 113.831 1.00 0.00

ATOM 4866 H PHE A 308 -20.483 25.819 116.391 1.00 0.00

ATOM 4867 H PHE A 308 -20.545 24.815 114.124 1.00 0.00

ATOM 4868 N ALA A 309 -24.028 20.841 120.624 1.00 79.91

ATOM 4869 CA ALA A 309 -24.304 19.897 121.669 1.00 81.69

ATOM 4870 C ALA A 309 -25.773 19.840 122.031 1.00 84.06

ATOM 4871 O ALA A 309 -26.298 18.756 122.170 1.00 85.23

ATOM 4872 CB ALA A 309 -23.426 20.154 122.890 1.00 82.62

ATOM 4873 H ALA A 309 -24.406 21.798 120.663 1.00 0.00

ATOM 4874 H ALA A 309 -24.051 18.911 121.280 1.00 0.00

ATOM 4875 H ALA A 309 -23.690 19.404 123.636 1.00 0.00

ATOM 4876 H ALA A 309 -22.390 20.055 122.564 1.00 0.00

ATOM 4877 H ALA A 309 -23.647 21.164 123.235 1.00 0.00

ATOM 4878 N GLU A 310 -26.456 20.973 122.168 1.00 85.84

ATOM 4879 CA GLU A 310 -27.861 20.904 122.644 1.00 90.26

ATOM 4880 C GLU A 310 -28.770 20.687 121.455 1.00 90.03

ATOM 4881 O GLU A 310 -29.882 20.196 121.602 1.00 92.00

ATOM 4882 CB GLU A 310 -28.326 22.142 123.425 1.00 92.59

ATOM 4883 CG GLU A 310 -27.269 22.822 124.284 1.00 94.35

ATOM 4884 CD GLU A 310 -27.423 24.355 124.309 1.00 97.58

ATOM 4885 OE1 GLU A 310 -26.690 25.045 123.533 1.00 97.66

ATOM 4886 OE2 GLU A 310 -28.262 24.858 125.094 1.00 99.03

ATOM 4887 H GLU A 310 -26.019 21.880 121.948 1.00 0.00

ATOM 4888 H GLU A 310 -27.911 20.072 123.346 1.00 0.00

ATOM 4889 H GLU A 310 -29.107 21.807 124.107 1.00 0.00

ATOM 4890 H GLU A 310 -28.627 22.879 122.681 1.00 0.00

ATOM 4891 H GLU A 310 -26.297 22.589 123.850 1.00 0.00

ATOM 4892 H GLU A 310 -27.373 22.448 125.303 1.00 0.00

ATOM 4893 N ASP A 311 -28.276 21.036 120.268 1.00 87.51

ATOM 4894 CA ASP A 311 -29.109 20.923 119.085 1.00 88.11

ATOM 4895 C ASP A 311 -29.694 19.507 118.891 1.00 89.21

ATOM 4896 O ASP A 311 -28.992 18.528 118.590 1.00 87.14

ATOM 4897 CB ASP A 311 -28.392 21.431 117.848 1.00 84.95

ATOM 4898 CG ASP A 311 -29.265 22.329 117.017 1.00 87.61

ATOM 4899 OD1 ASP A 311 -30.471 22.003 116.873 1.00 93.02

ATOM 4900 OD2 ASP A 311 -28.767 23.378 116.539 1.00 85.79

ATOM 4901 H ASP A 311 -27.310 21.384 120.190 1.00 0.00

ATOM 4902 H ASP A 311 -29.969 21.573 119.247 1.00 0.00

ATOM 4903 H ASP A 311 -28.106 20.575 117.237 1.00 0.00

ATOM 4904 H ASP A 311 -27.528 22.012 118.171 1.00 0.00

ATOM 4905 N LYS A 312 -31.000 19.425 119.093 1.00 92.48

ATOM 4906 CA LYS A 312 -31.738 18.179 119.000 1.00 94.30

ATOM 4907 C LYS A 312 -31.694 17.604 117.593 1.00 92.49

ATOM 4908 O LYS A 312 -32.388 16.634 117.311 1.00 93.96

ATOM 4909 CB LYS A 312 -33.178 18.427 119.461 1.00 99.21

ATOM 4910 CG LYS A 312 -33.683 19.855 119.192 1.00100.61

ATOM 4911 CD LYS A 312 -34.499 19.909 117.920 1.00100.75

ATOM 4912 CE LYS A 312 -35.735 20.747 118.158 1.00104.72

ATOM 4913 NZ LYS A 312 -36.948 20.010 117.743 1.00106.83

ATOM 4914 H LYS A 312 -31.516 20.285 119.327 1.00 0.00

ATOM 4915 H LYS A 312 -31.273 17.436 119.648 1.00 0.00

ATOM 4916 H LYS A 312 -33.200 18.274 120.540 1.00 0.00

ATOM 4917 H LYS A 312 -33.819 17.744 118.904 1.00 0.00

ATOM 4918 H LYS A 312 -32.832 20.528 119.091 1.00 0.00

ATOM 4919 H LYS A 312 -34.325 20.161 120.018 1.00 0.00

ATOM 4920 H LYS A 312 -34.791 18.900 117.630 1.00 0.00

ATOM 4921 H LYS A 312 -33.904 20.353 117.122 1.00 0.00

ATOM 4922 H LYS A 312 -35.671 21.673 117.587 1.00 0.00

ATOM 4923 H LYS A 312 -35.818 20.961 119.224 1.00 0.00

ATOM 4924 H LYS A 312 -37.753 20.626 117.927 1.00 0.00

ATOM 4925 H LYS A 312 -36.846 19.803 116.739 1.00 0.00

ATOM 4926 H LYS A 312 -36.986 19.150 118.309 1.00 0.00

ATOM 4927 N ASP A 313 -30.846 18.207 116.743 1.00 89.12

ATOM 4928 CA ASP A 313 -30.671 17.862 115.333 1.00 87.01

ATOM 4929 C ASP A 313 -29.209 17.680 114.936 1.00 83.50

ATOM 4930 O ASP A 313 -28.937 17.568 113.736 1.00 82.58

ATOM 4931 CB ASP A 313 -31.235 18.966 114.409 1.00 87.38

ATOM 4932 CG ASP A 313 -32.757 19.012 114.389 1.00 92.10

ATOM 4933 OD1 ASP A 313 -33.309 20.096 114.048 1.00 91.85

ATOM 4934 OD2 ASP A 313 -33.404 17.972 114.712 1.00 94.47

ATOM 4935 H ASP A 313 -30.271 18.976 117.116 1.00 0.00

ATOM 4936 H ASP A 313 -31.208 16.921 115.212 1.00 0.00

ATOM 4937 H ASP A 313 -30.897 18.766 113.392 1.00 0.00

ATOM 4938 H ASP A 313 -30.892 19.926 114.794 1.00 0.00

ATOM 4939 N VAL A 314 -28.280 17.651 115.893 1.00 81.35

ATOM 4940 CA VAL A 314 -26.866 17.437 115.563 1.00 78.92

ATOM 4941 C VAL A 314 -26.600 16.232 114.641 1.00 78.25

ATOM 4942 O VAL A 314 -25.885 16.386 113.646 1.00 76.28

ATOM 4943 CB VAL A 314 -25.914 17.290 116.804 1.00 78.85

ATOM 4944 CG1 VAL A 314 -24.492 17.086 116.329 1.00 76.41

ATOM 4945 CG2 VAL A 314 -25.939 18.498 117.701 1.00 80.20

ATOM 4946 H VAL A 314 -28.559 17.780 116.876 1.00 0.00

ATOM 4947 H VAL A 314 -26.632 18.362 115.036 1.00 0.00

ATOM 4948 H VAL A 314 -26.271 16.433 117.375 1.00 0.00

ATOM 4949 H VAL A 314 -23.870 16.989 117.219 1.00 0.00

ATOM 4950 H VAL A 314 -24.484 16.177 115.728 1.00 0.00

ATOM 4951 H VAL A 314 -24.224 17.963 115.740 1.00 0.00

ATOM 4952 H VAL A 314 -25.252 18.298 118.524 1.00 0.00

ATOM 4953 H VAL A 314 -25.615 19.348 117.100 1.00 0.00

ATOM 4954 H VAL A 314 -26.965 18.615 118.049 1.00 0.00

ATOM 4955 N CYS A 315 -27.123 15.047 114.987 1.00 79.37

ATOM 4956 CA CYS A 315 -26.788 13.814 114.252 1.00 79.83

ATOM 4957 C CYS A 315 -27.327 13.824 112.840 1.00 78.99

ATOM 4958 O CYS A 315 -26.652 13.386 111.898 1.00 76.87

ATOM 4959 CB CYS A 315 -27.340 12.578 114.938 1.00 82.76

ATOM 4960 SG CYS A 315 -26.391 11.941 116.305 1.00 88.35

ATOM 4961 H CYS A 315 -27.773 14.997 115.784 1.00 0.00

ATOM 4962 H CYS A 315 -25.699 13.781 114.233 1.00 0.00

ATOM 4963 H CYS A 315 -27.213 11.848 114.138 1.00 0.00

ATOM 4964 H CYS A 315 -28.366 12.833 115.202 1.00 0.00

ATOM 4965 N LYS A 316 -28.571 14.292 112.733 1.00 80.02

ATOM 4966 CA LYS A 316 -29.223 14.650 111.472 1.00 80.33

ATOM 4967 C LYS A 316 -28.359 15.624 110.628 1.00 78.30

ATOM 4968 O LYS A 316 -28.002 15.335 109.490 1.00 78.06

ATOM 4969 CB LYS A 316 -30.581 15.283 111.795 1.00 82.37

ATOM 4970 CG LYS A 316 -31.620 15.150 110.712 1.00 83.79

ATOM 4971 CD LYS A 316 -32.113 16.523 110.278 1.00 83.39

ATOM 4972 CE LYS A 316 -33.065 16.430 109.074 1.00 87.47

ATOM 4973 NZ LYS A 316 -34.118 15.333 109.202 1.00 86.60

ATOM 4974 H LYS A 316 -29.113 14.410 113.601 1.00 0.00

ATOM 4975 H LYS A 316 -29.355 13.748 110.874 1.00 0.00

ATOM 4976 H LYS A 316 -30.418 16.350 111.945 1.00 0.00

ATOM 4977 H LYS A 316 -30.978 14.751 112.660 1.00 0.00

ATOM 4978 H LYS A 316 -32.461 14.579 111.105 1.00 0.00

ATOM 4979 H LYS A 316 -31.179 14.637 109.858 1.00 0.00

ATOM 4980 H LYS A 316 -31.246 17.115 109.985 1.00 0.00

ATOM 4981 H LYS A 316 -32.646 16.977 111.113 1.00 0.00

ATOM 4982 H LYS A 316 -32.463 16.201 108.194 1.00 0.00

ATOM 4983 H LYS A 316 -33.599 17.378 109.009 1.00 0.00

ATOM 4984 H LYS A 316 -34.692 15.365 108.347 1.00 0.00

ATOM 4985 H LYS A 316 -33.613 14.440 109.291 1.00 0.00

ATOM 4986 H LYS A 316 -34.673 15.545 110.044 1.00 0.00

ATOM 4987 N ASN A 317 -28.008 16.769 111.204 1.00 77.39

ATOM 4988 CA ASN A 317 -27.122 17.721 110.544 1.00 76.25

ATOM 4989 C ASN A 317 -25.893 17.005 109.997 1.00 75.37

ATOM 4990 O ASN A 317 -25.534 17.178 108.842 1.00 75.40

ATOM 4991 CB ASN A 317 -26.670 18.852 111.505 1.00 74.61

ATOM 4992 CG ASN A 317 -27.839 19.711 112.061 1.00 75.85

ATOM 4993 OD1 ASN A 317 -28.964 19.718 111.538 1.00 72.86

ATOM 4994 ND2 ASN A 317 -27.547 20.444 113.147 1.00 74.31

ATOM 4995 H ASN A 317 -28.372 16.990 112.142 1.00 0.00

ATOM 4996 H ASN A 317 -27.684 18.172 109.726 1.00 0.00

ATOM 4997 H ASN A 317 -26.020 19.518 110.937 1.00 0.00

ATOM 4998 H ASN A 317 -26.182 18.378 112.356 1.00 0.00

ATOM 4999 H ASN A 317 -28.269 21.040 113.575 1.00 0.00

ATOM 5000 H ASN A 317 -26.601 20.410 113.552 1.00 0.00

ATOM 5001 N TYR A 318 -25.277 16.185 110.845 1.00 75.84

ATOM 5002 CA TYR A 318 -23.933 15.654 110.612 1.00 75.08

ATOM 5003 C TYR A 318 -24.010 14.631 109.487 1.00 76.54

ATOM 5004 O TYR A 318 -23.271 14.725 108.482 1.00 75.96

ATOM 5005 CB TYR A 318 -23.378 15.091 111.930 1.00 74.77

ATOM 5006 CG TYR A 318 -22.154 14.217 111.861 1.00 73.86

ATOM 5007 CD1 TYR A 318 -20.953 14.681 111.332 1.00 75.22

ATOM 5008 CD2 TYR A 318 -22.194 12.916 112.371 1.00 76.95

ATOM 5009 CE1 TYR A 318 -19.819 13.833 111.286 1.00 76.88

ATOM 5010 CE2 TYR A 318 -21.084 12.078 112.360 1.00 76.66

ATOM 5011 CZ TYR A 318 -19.916 12.526 111.808 1.00 77.14

ATOM 5012 OH TYR A 318 -18.854 11.658 111.773 1.00 78.29

ATOM 5013 H TYR A 318 -25.770 15.911 111.707 1.00 0.00

ATOM 5014 H TYR A 318 -23.236 16.429 110.293 1.00 0.00

ATOM 5015 H TYR A 318 -24.162 14.460 112.349 1.00 0.00

ATOM 5016 H TYR A 318 -23.062 15.960 112.507 1.00 0.00

ATOM 5017 H TYR A 318 -20.885 15.700 110.952 1.00 0.00

ATOM 5018 H TYR A 318 -23.128 12.545 112.792 1.00 0.00

ATOM 5019 H TYR A 318 -18.883 14.186 110.853 1.00 0.00

ATOM 5020 H TYR A 318 -21.144 11.077 112.786 1.00 0.00

ATOM 5021 H TYR A 318 -18.073 12.156 111.322 1.00 0.00

ATOM 5022 N GLN A 319 -24.950 13.706 109.611 1.00 77.95

ATOM 5023 CA GLN A 319 -25.139 12.717 108.552 1.00 80.50

ATOM 5024 C GLN A 319 -25.413 13.244 107.140 1.00 81.08

ATOM 5025 O GLN A 319 -25.016 12.614 106.170 1.00 82.67

ATOM 5026 CB GLN A 319 -26.155 11.663 108.963 1.00 82.79

ATOM 5027 CG GLN A 319 -25.484 10.587 109.805 1.00 84.40

ATOM 5028 CD GLN A 319 -26.344 10.142 110.952 1.00 85.83

ATOM 5029 OE1 GLN A 319 -26.960 10.957 111.623 1.00 85.85

ATOM 5030 NE2 GLN A 319 -26.381 8.843 111.191 1.00 87.78

ATOM 5031 H GLN A 319 -25.546 13.684 110.451 1.00 0.00

ATOM 5032 H GLN A 319 -24.152 12.266 108.451 1.00 0.00

ATOM 5033 H GLN A 319 -26.575 11.205 108.068 1.00 0.00

ATOM 5034 H GLN A 319 -26.943 12.136 109.549 1.00 0.00

ATOM 5035 H GLN A 319 -24.558 10.991 110.214 1.00 0.00

ATOM 5036 H GLN A 319 -25.311 9.719 109.169 1.00 0.00

ATOM 5037 H GLN A 319 -26.954 8.478 111.965 1.00 0.00

ATOM 5038 H GLN A 319 -25.837 8.195 110.603 1.00 0.00

ATOM 5039 N GLU A 320 -26.034 14.418 107.023 1.00 80.68

ATOM 5040 CA GLU A 320 -26.361 14.961 105.704 1.00 80.63

ATOM 5041 C GLU A 320 -25.146 15.527 104.948 1.00 78.06

ATOM 5042 O GLU A 320 -25.056 15.313 103.768 1.00 79.00

ATOM 5043 CB GLU A 320 -27.667 15.813 105.728 1.00 81.85

ATOM 5044 CG GLU A 320 -28.888 14.964 106.312 1.00 85.06

ATOM 5045 CD GLU A 320 -30.299 15.572 106.112 1.00 89.58

ATOM 5046 OE1 GLU A 320 -30.398 16.774 105.740 1.00 89.73

ATOM 5047 OE2 GLU A 320 -31.311 14.836 106.338 1.00 90.64

ATOM 5048 H GLU A 320 -26.286 14.948 107.869 1.00 0.00

ATOM 5049 H GLU A 320 -26.630 14.129 105.053 1.00 0.00

ATOM 5050 H GLU A 320 -27.898 16.099 104.702 1.00 0.00

ATOM 5051 H GLU A 320 -27.500 16.694 106.348 1.00 0.00

ATOM 5052 H GLU A 320 -28.744 14.877 107.389 1.00 0.00

ATOM 5053 H GLU A 320 -28.903 14.028 105.753 1.00 0.00

ATOM 5054 N ALA A 321 -24.251 16.257 105.621 1.00 75.71

ATOM 5055 CA ALA A 321 -22.843 16.474 105.144 1.00 74.63

ATOM 5056 C ALA A 321 -21.785 16.444 106.280 1.00 72.33

ATOM 5057 O ALA A 321 -21.576 17.419 106.971 1.00 70.58

ATOM 5058 CB ALA A 321 -22.699 17.736 104.304 1.00 73.74

ATOM 5059 H ALA A 321 -24.540 16.691 106.509 1.00 0.00

ATOM 5060 H ALA A 321 -22.635 15.617 104.503 1.00 0.00

ATOM 5061 H ALA A 321 -21.652 17.804 104.009 1.00 0.00

ATOM 5062 H ALA A 321 -23.357 17.622 103.442 1.00 0.00

ATOM 5063 H ALA A 321 -22.998 18.574 104.934 1.00 0.00

ATOM 5064 N LYS A 322 -21.128 15.307 106.453 1.00 73.59

ATOM 5065 CA LYS A 322 -20.248 15.074 107.598 1.00 73.57

ATOM 5066 C LYS A 322 -19.096 16.076 107.544 1.00 72.78

ATOM 5067 O LYS A 322 -18.854 16.836 108.499 1.00 72.24

ATOM 5068 CB LYS A 322 -19.744 13.625 107.615 1.00 75.09

ATOM 5069 CG LYS A 322 -20.849 12.535 107.865 1.00 76.44

ATOM 5070 CD LYS A 322 -20.233 11.118 107.884 1.00 74.57

ATOM 5071 CE LYS A 322 -21.220 10.030 108.303 1.00 77.06

ATOM 5072 NZ LYS A 322 -20.759 9.024 109.387 1.00 76.32

ATOM 5073 H LYS A 322 -21.241 14.558 105.755 1.00 0.00

ATOM 5074 H LYS A 322 -20.801 15.223 108.526 1.00 0.00

ATOM 5075 H LYS A 322 -19.050 13.565 108.453 1.00 0.00

ATOM 5076 H LYS A 322 -19.306 13.435 106.635 1.00 0.00

ATOM 5077 H LYS A 322 -21.593 12.583 107.069 1.00 0.00

ATOM 5078 H LYS A 322 -21.307 12.717 108.837 1.00 0.00

ATOM 5079 H LYS A 322 -19.417 11.116 108.607 1.00 0.00

ATOM 5080 H LYS A 322 -19.915 10.886 106.868 1.00 0.00

ATOM 5081 H LYS A 322 -21.384 9.437 107.404 1.00 0.00

ATOM 5082 H LYS A 322 -22.077 10.557 108.722 1.00 0.00

ATOM 5083 H LYS A 322 -21.540 8.371 109.543 1.00 0.00

ATOM 5084 H LYS A 322 -19.926 8.544 109.017 1.00 0.00

ATOM 5085 H LYS A 322 -20.542 9.571 110.233 1.00 0.00

ATOM 5086 N ASP A 323 -18.429 16.139 106.408 1.00 73.18

ATOM 5087 CA ASP A 323 -17.333 17.068 106.272 1.00 72.47

ATOM 5088 C ASP A 323 -17.713 18.584 106.386 1.00 70.98

ATOM 5089 O ASP A 323 -16.905 19.414 106.848 1.00 69.61

ATOM 5090 CB ASP A 323 -16.495 16.707 105.020 1.00 73.77

ATOM 5091 CG ASP A 323 -15.668 15.397 105.211 1.00 77.63

ATOM 5092 OD1 ASP A 323 -15.796 14.712 106.259 1.00 78.81

ATOM 5093 OD2 ASP A 323 -14.867 15.033 104.312 1.00 82.51

ATOM 5094 H ASP A 323 -18.689 15.528 105.621 1.00 0.00

ATOM 5095 H ASP A 323 -16.701 16.944 107.151 1.00 0.00

ATOM 5096 H ASP A 323 -15.795 17.524 104.844 1.00 0.00

ATOM 5097 H ASP A 323 -17.182 16.559 104.187 1.00 0.00

ATOM 5098 N ALA A 324 -18.925 18.956 105.979 1.00 70.81

ATOM 5099 CA ALA A 324 -19.279 20.370 106.032 1.00 69.30

ATOM 5100 C ALA A 324 -19.603 20.722 107.467 1.00 68.08

ATOM 5101 O ALA A 324 -19.155 21.728 108.009 1.00 66.59

ATOM 5102 CB ALA A 324 -20.446 20.695 105.103 1.00 69.94

ATOM 5103 H ALA A 324 -19.600 18.259 105.633 1.00 0.00

ATOM 5104 H ALA A 324 -18.437 20.969 105.685 1.00 0.00

ATOM 5105 H ALA A 324 -20.645 21.762 105.202 1.00 0.00

ATOM 5106 H ALA A 324 -20.131 20.434 104.093 1.00 0.00

ATOM 5107 H ALA A 324 -21.293 20.093 105.432 1.00 0.00

ATOM 5108 N PHE A 325 -20.387 19.866 108.087 1.00 69.17

ATOM 5109 CA PHE A 325 -20.750 20.063 109.478 1.00 68.97

ATOM 5110 C PHE A 325 -19.546 20.101 110.411 1.00 67.45

ATOM 5111 O PHE A 325 -19.576 20.785 111.424 1.00 67.53

ATOM 5112 CB PHE A 325 -21.724 18.999 109.922 1.00 69.89

ATOM 5113 CG PHE A 325 -22.257 19.232 111.283 1.00 71.25

ATOM 5114 CD1 PHE A 325 -23.200 20.246 111.508 1.00 70.90

ATOM 5115 CD2 PHE A 325 -21.828 18.440 112.354 1.00 70.95

ATOM 5116 CE1 PHE A 325 -23.712 20.473 112.785 1.00 71.41

ATOM 5117 CE2 PHE A 325 -22.334 18.649 113.633 1.00 71.47

ATOM 5118 CZ PHE A 325 -23.277 19.683 113.846 1.00 73.42

ATOM 5119 H PHE A 325 -20.747 19.046 107.579 1.00 0.00

ATOM 5120 H PHE A 325 -21.224 21.042 109.541 1.00 0.00

ATOM 5121 H PHE A 325 -21.209 18.039 109.928 1.00 0.00

ATOM 5122 H PHE A 325 -22.574 19.023 109.240 1.00 0.00

ATOM 5123 H PHE A 325 -23.536 20.863 110.675 1.00 0.00

ATOM 5124 H PHE A 325 -21.092 17.654 112.185 1.00 0.00

ATOM 5125 H PHE A 325 -24.446 21.262 112.951 1.00 0.00

ATOM 5126 H PHE A 325 -22.007 18.023 114.463 1.00 0.00

ATOM 5127 H PHE A 325 -23.666 19.862 114.848 1.00 0.00

ATOM 5128 N LEU A 326 -18.489 19.384 110.055 1.00 66.66

ATOM 5129 CA LEU A 326 -17.283 19.347 110.854 1.00 65.87

ATOM 5130 C LEU A 326 -16.376 20.522 110.567 1.00 65.44

ATOM 5131 O LEU A 326 -15.791 21.100 111.512 1.00 65.47

ATOM 5132 CB LEU A 326 -16.534 18.058 110.585 1.00 67.19

ATOM 5133 CG LEU A 326 -17.068 16.813 111.283 1.00 68.63

ATOM 5134 CD1 LEU A 326 -16.259 15.625 110.720 1.00 73.00

ATOM 5135 CD2 LEU A 326 -16.908 16.980 112.825 1.00 67.35

ATOM 5136 H LEU A 326 -18.527 18.837 109.183 1.00 0.00

ATOM 5137 H LEU A 326 -17.580 19.402 111.901 1.00 0.00

ATOM 5138 H LEU A 326 -15.525 18.212 110.966 1.00 0.00

ATOM 5139 H LEU A 326 -16.620 17.873 109.514 1.00 0.00

ATOM 5140 H LEU A 326 -18.130 16.645 111.102 1.00 0.00

ATOM 5141 H LEU A 326 -16.630 14.725 111.211 1.00 0.00

ATOM 5142 H LEU A 326 -16.434 15.601 109.644 1.00 0.00

ATOM 5143 H LEU A 326 -15.211 15.812 110.956 1.00 0.00

ATOM 5144 H LEU A 326 -17.299 16.073 113.286 1.00 0.00

ATOM 5145 H LEU A 326 -15.844 17.108 113.023 1.00 0.00

ATOM 5146 H LEU A 326 -17.483 17.861 113.111 1.00 0.00

ATOM 5147 N GLY A 327 -16.220 20.851 109.274 1.00 64.50

ATOM 5148 CA GLY A 327 -15.628 22.118 108.851 1.00 63.21

ATOM 5149 C GLY A 327 -16.302 23.264 109.600 1.00 63.42

ATOM 5150 O GLY A 327 -15.636 24.121 110.214 1.00 62.63

ATOM 5151 H GLY A 327 -16.530 20.184 108.553 1.00 0.00

ATOM 5152 H GLY A 327 -15.805 22.228 107.781 1.00 0.00

ATOM 5153 H GLY A 327 -14.554 22.099 109.038 1.00 0.00

ATOM 5154 N SER A 328 -17.632 23.235 109.611 1.00 63.77

ATOM 5155 CA SER A 328 -18.370 24.207 110.344 1.00 64.81

ATOM 5156 C SER A 328 -17.983 24.243 111.821 1.00 65.15

ATOM 5157 O SER A 328 -17.783 25.328 112.378 1.00 65.42

ATOM 5158 CB SER A 328 -19.842 23.962 110.213 1.00 66.31

ATOM 5159 OG SER A 328 -20.497 24.683 111.246 1.00 68.68

ATOM 5160 H SER A 328 -18.132 22.504 109.085 1.00 0.00

ATOM 5161 H SER A 328 -18.121 25.177 109.914 1.00 0.00

ATOM 5162 H SER A 328 -20.095 22.904 110.274 1.00 0.00

ATOM 5163 H SER A 328 -20.202 24.337 109.255 1.00 0.00

ATOM 5164 H SER A 328 -21.506 24.506 111.144 1.00 0.00

ATOM 5165 N PHE A 329 -17.835 23.078 112.445 1.00 64.65

ATOM 5166 CA PHE A 329 -17.353 23.070 113.816 1.00 65.03

ATOM 5167 C PHE A 329 -15.953 23.656 113.923 1.00 63.77

ATOM 5168 O PHE A 329 -15.647 24.337 114.868 1.00 64.08

ATOM 5169 CB PHE A 329 -17.401 21.644 114.442 1.00 66.66

ATOM 5170 CG PHE A 329 -16.704 21.531 115.793 1.00 66.86

ATOM 5171 CD1 PHE A 329 -17.345 21.912 116.955 1.00 69.28

ATOM 5172 CD2 PHE A 329 -15.399 21.059 115.885 1.00 67.43

ATOM 5173 CE1 PHE A 329 -16.717 21.791 118.204 1.00 71.37

ATOM 5174 CE2 PHE A 329 -14.752 20.959 117.127 1.00 68.60

ATOM 5175 CZ PHE A 329 -15.409 21.316 118.286 1.00 70.65

ATOM 5176 H PHE A 329 -18.059 22.195 111.964 1.00 0.00

ATOM 5177 H PHE A 329 -18.029 23.706 114.387 1.00 0.00

ATOM 5178 H PHE A 329 -16.896 20.958 113.762 1.00 0.00

ATOM 5179 H PHE A 329 -18.450 21.406 114.619 1.00 0.00

ATOM 5180 H PHE A 329 -18.357 22.314 116.901 1.00 0.00

ATOM 5181 H PHE A 329 -14.870 20.762 114.979 1.00 0.00

ATOM 5182 H PHE A 329 -17.253 22.069 119.112 1.00 0.00

ATOM 5183 H PHE A 329 -13.725 20.597 117.176 1.00 0.00

ATOM 5184 H PHE A 329 -14.912 21.228 119.252 1.00 0.00

ATOM 5185 N LEU A 330 -15.098 23.400 112.944 1.00 63.25

ATOM 5186 CA LEU A 330 -13.722 23.854 113.035 1.00 61.92

ATOM 5187 C LEU A 330 -13.675 25.372 112.854 1.00 62.13

ATOM 5188 O LEU A 330 -13.085 26.108 113.680 1.00 62.19

ATOM 5189 CB LEU A 330 -12.904 23.123 111.989 1.00 61.96

ATOM 5190 CG LEU A 330 -11.402 23.006 112.040 1.00 60.29

ATOM 5191 CD1 LEU A 330 -10.815 22.842 113.468 1.00 58.11

ATOM 5192 CD2 LEU A 330 -11.118 21.843 111.146 1.00 59.98

ATOM 5193 H LEU A 330 -15.411 22.876 112.114 1.00 0.00

ATOM 5194 H LEU A 330 -13.298 23.632 114.014 1.00 0.00

ATOM 5195 H LEU A 330 -13.073 23.710 111.086 1.00 0.00

ATOM 5196 H LEU A 330 -13.229 22.089 112.104 1.00 0.00

ATOM 5197 H LEU A 330 -10.914 23.924 111.713 1.00 0.00

ATOM 5198 H LEU A 330 -9.733 22.770 113.361 1.00 0.00

ATOM 5199 H LEU A 330 -11.108 23.725 114.036 1.00 0.00

ATOM 5200 H LEU A 330 -11.243 21.931 113.886 1.00 0.00

ATOM 5201 H LEU A 330 -10.037 21.703 111.139 1.00 0.00

ATOM 5202 H LEU A 330 -11.635 20.982 111.569 1.00 0.00

ATOM 5203 H LEU A 330 -11.500 22.100 110.158 1.00 0.00

ATOM 5204 N TYR A 331 -14.363 25.857 111.818 1.00 61.77

ATOM 5205 CA TYR A 331 -14.596 27.294 111.697 1.00 61.48

ATOM 5206 C TYR A 331 -15.012 27.901 113.037 1.00 61.62

ATOM 5207 O TYR A 331 -14.253 28.695 113.593 1.00 62.17

ATOM 5208 CB TYR A 331 -15.648 27.567 110.639 1.00 62.48

ATOM 5209 CG TYR A 331 -16.274 28.955 110.643 1.00 63.75

ATOM 5210 CD1 TYR A 331 -15.628 30.030 110.068 1.00 61.25

ATOM 5211 CD2 TYR A 331 -17.556 29.158 111.213 1.00 65.58

ATOM 5212 CE1 TYR A 331 -16.202 31.271 110.060 1.00 65.38

ATOM 5213 CE2 TYR A 331 -18.145 30.383 111.202 1.00 66.25

ATOM 5214 CZ TYR A 331 -17.475 31.458 110.635 1.00 67.28

ATOM 5215 OH TYR A 331 -18.088 32.715 110.653 1.00 67.52

ATOM 5216 H TYR A 331 -14.731 25.215 111.101 1.00 0.00

ATOM 5217 H TYR A 331 -13.661 27.765 111.393 1.00 0.00

ATOM 5218 H TYR A 331 -16.461 26.869 110.840 1.00 0.00

ATOM 5219 H TYR A 331 -15.143 27.464 109.679 1.00 0.00

ATOM 5220 H TYR A 331 -14.647 29.888 109.614 1.00 0.00

ATOM 5221 H TYR A 331 -18.079 28.317 111.668 1.00 0.00

ATOM 5222 H TYR A 331 -15.676 32.113 109.610 1.00 0.00

ATOM 5223 H TYR A 331 -19.136 30.520 111.635 1.00 0.00

ATOM 5224 H TYR A 331 -17.441 33.372 110.194 1.00 0.00

ATOM 5225 N GLU A 332 -16.198 27.521 113.536 1.00 60.62

ATOM 5226 CA GLU A 332 -16.733 28.025 114.801 1.00 61.51

ATOM 5227 C GLU A 332 -15.758 28.022 115.963 1.00 62.38

ATOM 5228 O GLU A 332 -15.752 28.941 116.751 1.00 63.60

ATOM 5229 CB GLU A 332 -17.987 27.241 115.256 1.00 61.61

ATOM 5230 CG GLU A 332 -19.155 27.311 114.323 1.00 58.57

ATOM 5231 CD GLU A 332 -19.534 28.736 113.941 1.00 61.86

ATOM 5232 OE1 GLU A 332 -19.094 29.728 114.598 1.00 62.88

ATOM 5233 OE2 GLU A 332 -20.289 28.861 112.966 1.00 62.26

ATOM 5234 H GLU A 332 -16.759 26.842 113.003 1.00 0.00

ATOM 5235 H GLU A 332 -16.974 29.061 114.564 1.00 0.00

ATOM 5236 H GLU A 332 -18.321 27.657 116.206 1.00 0.00

ATOM 5237 H GLU A 332 -17.704 26.189 115.284 1.00 0.00

ATOM 5238 H GLU A 332 -20.005 26.873 114.846 1.00 0.00

ATOM 5239 H GLU A 332 -18.885 26.772 113.415 1.00 0.00

ATOM 5240 N TYR A 333 -14.964 26.965 116.077 1.00 62.80

ATOM 5241 CA TYR A 333 -14.066 26.800 117.194 1.00 63.70

ATOM 5242 C TYR A 333 -12.854 27.668 116.998 1.00 63.43

ATOM 5243 O TYR A 333 -12.415 28.324 117.917 1.00 64.15

ATOM 5244 CB TYR A 333 -13.656 25.322 117.360 1.00 64.31

ATOM 5245 CG TYR A 333 -13.411 24.984 118.781 1.00 64.60

ATOM 5246 CD1 TYR A 333 -12.309 25.479 119.438 1.00 67.03

ATOM 5247 CD2 TYR A 333 -14.343 24.235 119.508 1.00 68.88

ATOM 5248 CE1 TYR A 333 -12.102 25.204 120.805 1.00 69.80

ATOM 5249 CE2 TYR A 333 -14.159 23.948 120.859 1.00 68.18

ATOM 5250 CZ TYR A 333 -13.039 24.447 121.497 1.00 70.71

ATOM 5251 OH TYR A 333 -12.829 24.198 122.825 1.00 76.14

ATOM 5252 H TYR A 333 -14.990 26.240 115.346 1.00 0.00

ATOM 5253 H TYR A 333 -14.580 27.105 118.106 1.00 0.00

ATOM 5254 H TYR A 333 -12.746 25.118 116.795 1.00 0.00

ATOM 5255 H TYR A 333 -14.482 24.695 117.024 1.00 0.00

ATOM 5256 H TYR A 333 -11.588 26.091 118.896 1.00 0.00

ATOM 5257 H TYR A 333 -15.236 23.866 119.004 1.00 0.00

ATOM 5258 H TYR A 333 -11.215 25.582 121.314 1.00 0.00

ATOM 5259 H TYR A 333 -14.884 23.342 121.402 1.00 0.00

ATOM 5260 H TYR A 333 -11.943 24.657 123.081 1.00 0.00

ATOM 5261 N SER A 334 -12.327 27.681 115.777 1.00 63.45

ATOM 5262 CA SER A 334 -11.043 28.335 115.479 1.00 62.66

ATOM 5263 C SER A 334 -11.120 29.837 115.548 1.00 64.09

ATOM 5264 O SER A 334 -10.109 30.525 115.863 1.00 65.93

ATOM 5265 CB SER A 334 -10.603 27.967 114.094 1.00 61.98

ATOM 5266 OG SER A 334 -10.482 26.574 113.968 1.00 62.46

ATOM 5267 H SER A 334 -12.836 27.216 115.012 1.00 0.00

ATOM 5268 H SER A 334 -10.338 27.991 116.235 1.00 0.00

ATOM 5269 H SER A 334 -9.648 28.427 113.840 1.00 0.00

ATOM 5270 H SER A 334 -11.375 28.272 113.388 1.00 0.00

ATOM 5271 H SER A 334 -10.179 26.384 113.002 1.00 0.00

ATOM 5272 N ARG A 335 -12.300 30.378 115.252 1.00 63.68

ATOM 5273 CA ARG A 335 -12.405 31.812 115.195 1.00 63.85

ATOM 5274 C ARG A 335 -12.488 32.378 116.624 1.00 65.99

ATOM 5275 O ARG A 335 -12.109 33.523 116.836 1.00 67.03

ATOM 5276 CB ARG A 335 -13.575 32.246 114.303 1.00 64.09

ATOM 5277 CG ARG A 335 -15.005 32.018 114.852 1.00 63.63

ATOM 5278 CD ARG A 335 -16.051 31.884 113.732 1.00 62.89

ATOM 5279 NE ARG A 335 -16.324 33.144 113.010 1.00 65.40

ATOM 5280 CZ ARG A 335 -17.494 33.798 113.038 1.00 66.25

ATOM 5281 NH1 ARG A 335 -18.524 33.306 113.742 1.00 62.13

ATOM 5282 NH2 ARG A 335 -17.627 34.939 112.357 1.00 63.93

ATOM 5283 H ARG A 335 -13.120 29.783 115.068 1.00 0.00

ATOM 5284 H ARG A 335 -11.511 32.228 114.730 1.00 0.00

ATOM 5285 H ARG A 335 -13.500 31.629 113.408 1.00 0.00

ATOM 5286 H ARG A 335 -13.467 33.323 114.176 1.00 0.00

ATOM 5287 H ARG A 335 -15.277 32.877 115.465 1.00 0.00

ATOM 5288 H ARG A 335 -15.004 31.086 115.418 1.00 0.00

ATOM 5289 H ARG A 335 -16.994 31.550 114.166 1.00 0.00

ATOM 5290 H ARG A 335 -15.640 31.203 112.987 1.00 0.00

ATOM 5291 H ARG A 335 -15.563 33.548 112.446 1.00 0.00

ATOM 5292 H ARG A 335 -19.422 33.810 113.762 1.00 0.00

ATOM 5293 H ARG A 335 -18.417 32.424 114.263 1.00 0.00

ATOM 5294 H ARG A 335 -18.523 35.447 112.374 1.00 0.00

ATOM 5295 H ARG A 335 -16.834 35.311 111.815 1.00 0.00

ATOM 5296 N ARG A 336 -12.949 31.570 117.587 1.00 66.31

ATOM 5297 CA ARG A 336 -13.093 32.003 119.009 1.00 68.64

ATOM 5298 C ARG A 336 -11.855 31.766 119.860 1.00 69.22

ATOM 5299 O ARG A 336 -11.784 32.265 120.975 1.00 71.19

ATOM 5300 CB ARG A 336 -14.270 31.315 119.709 1.00 68.90

ATOM 5301 CG ARG A 336 -15.564 31.413 118.928 1.00 70.00

ATOM 5302 CD ARG A 336 -16.706 30.781 119.671 1.00 70.34

ATOM 5303 NE ARG A 336 -17.744 30.463 118.711 1.00 72.91

ATOM 5304 CZ ARG A 336 -18.953 31.000 118.718 1.00 76.95

ATOM 5305 NH1 ARG A 336 -19.309 31.867 119.655 1.00 80.14

ATOM 5306 NH2 ARG A 336 -19.826 30.638 117.804 1.00 80.42

ATOM 5307 H ARG A 336 -13.216 30.607 117.336 1.00 0.00

ATOM 5308 H ARG A 336 -13.264 33.077 118.932 1.00 0.00

ATOM 5309 H ARG A 336 -14.426 31.802 120.671 1.00 0.00

ATOM 5310 H ARG A 336 -14.028 30.256 119.801 1.00 0.00

ATOM 5311 H ARG A 336 -15.441 30.893 117.978 1.00 0.00

ATOM 5312 H ARG A 336 -15.798 32.467 118.780 1.00 0.00

ATOM 5313 H ARG A 336 -17.098 31.442 120.443 1.00 0.00

ATOM 5314 H ARG A 336 -16.378 29.859 120.151 1.00 0.00

ATOM 5315 H ARG A 336 -17.527 29.775 117.976 1.00 0.00

ATOM 5316 H ARG A 336 -20.254 32.276 119.646 1.00 0.00

ATOM 5317 H ARG A 336 -18.640 32.132 120.392 1.00 0.00

ATOM 5318 H ARG A 336 -20.770 31.051 117.801 1.00 0.00

ATOM 5319 H ARG A 336 -19.566 29.942 117.091 1.00 0.00

ATOM 5320 N HIS A 337 -10.911 30.988 119.333 1.00 70.81

ATOM 5321 CA HIS A 337 -9.708 30.629 120.057 1.00 71.37

ATOM 5322 C HIS A 337 -8.460 30.775 119.211 1.00 69.17

ATOM 5323 O HIS A 337 -7.816 29.812 118.949 1.00 65.82

ATOM 5324 CB HIS A 337 -9.772 29.202 120.572 1.00 70.41

ATOM 5325 CG HIS A 337 -10.798 28.992 121.623 1.00 75.06

ATOM 5326 ND1 HIS A 337 -10.738 29.607 122.849 1.00 82.11

ATOM 5327 CD2 HIS A 337 -11.916 28.231 121.636 1.00 78.15

ATOM 5328 CE1 HIS A 337 -11.782 29.240 123.572 1.00 87.23

ATOM 5329 NE2 HIS A 337 -12.507 28.395 122.864 1.00 82.97

ATOM 5330 H HIS A 337 -11.041 30.629 118.376 1.00 0.00

ATOM 5331 H HIS A 337 -9.652 31.324 120.895 1.00 0.00

ATOM 5332 H HIS A 337 -8.805 28.950 121.007 1.00 0.00

ATOM 5333 H HIS A 337 -10.055 28.566 119.733 1.00 0.00

ATOM 5334 H HIS A 337 -9.997 30.252 123.157 1.00 0.00

ATOM 5335 H HIS A 337 -12.280 27.604 120.822 1.00 0.00

ATOM 5336 H HIS A 337 -12.006 29.579 124.583 1.00 0.00

ATOM 5337 N PRO A 338 -8.092 32.017 118.846 1.00 71.61

ATOM 5338 CA PRO A 338 -6.862 32.332 118.083 1.00 70.23

ATOM 5339 C PRO A 338 -5.620 32.236 118.930 1.00 72.64

ATOM 5340 O PRO A 338 -4.530 32.529 118.454 1.00 73.23

ATOM 5341 CB PRO A 338 -7.061 33.802 117.725 1.00 71.33

ATOM 5342 CG PRO A 338 -7.874 34.350 118.828 1.00 73.88

ATOM 5343 CD PRO A 338 -8.801 33.248 119.264 1.00 74.17

ATOM 5344 H PRO A 338 -6.726 31.649 117.245 1.00 0.00

ATOM 5345 H PRO A 338 -7.540 33.947 116.757 1.00 0.00

ATOM 5346 H PRO A 338 -6.112 34.333 117.647 1.00 0.00

ATOM 5347 H PRO A 338 -8.435 35.220 118.486 1.00 0.00

ATOM 5348 H PRO A 338 -7.241 34.692 119.646 1.00 0.00

ATOM 5349 H PRO A 338 -8.994 33.263 120.337 1.00 0.00

ATOM 5350 H PRO A 338 -9.796 33.326 118.827 1.00 0.00

ATOM 5351 N GLU A 339 -5.816 31.950 120.217 1.00 75.97

ATOM 5352 CA GLU A 339 -4.739 31.601 121.127 1.00 77.64

ATOM 5353 C GLU A 339 -4.167 30.222 120.753 1.00 74.52

ATOM 5354 O GLU A 339 -2.965 30.034 120.848 1.00 74.74

ATOM 5355 CB GLU A 339 -5.232 31.543 122.583 1.00 81.24

ATOM 5356 CG GLU A 339 -6.189 32.643 123.001 1.00 86.31

ATOM 5357 CD GLU A 339 -7.666 32.234 122.915 1.00 87.29

ATOM 5358 OE1 GLU A 339 -7.991 31.429 122.041 1.00 78.63

ATOM 5359 OE2 GLU A 339 -8.493 32.734 123.728 1.00 91.54

ATOM 5360 H GLU A 339 -6.778 31.978 120.584 1.00 0.00

ATOM 5361 H GLU A 339 -3.972 32.371 121.041 1.00 0.00

ATOM 5362 H GLU A 339 -4.354 31.641 123.221 1.00 0.00

ATOM 5363 H GLU A 339 -5.789 30.612 122.682 1.00 0.00

ATOM 5364 H GLU A 339 -6.039 33.475 122.313 1.00 0.00

ATOM 5365 H GLU A 339 -5.968 32.893 124.039 1.00 0.00

ATOM 5366 N TYR A 340 -5.027 29.278 120.363 1.00 71.02

ATOM 5367 CA TYR A 340 -4.639 27.863 120.313 1.00 70.14

ATOM 5368 C TYR A 340 -3.703 27.569 119.139 1.00 68.31

ATOM 5369 O TYR A 340 -3.679 28.289 118.118 1.00 65.84

ATOM 5370 CB TYR A 340 -5.868 26.957 120.159 1.00 68.21

ATOM 5371 CG TYR A 340 -6.837 26.900 121.337 1.00 73.46

ATOM 5372 CD1 TYR A 340 -6.792 27.826 122.412 1.00 79.87

ATOM 5373 CD2 TYR A 340 -7.804 25.936 121.370 1.00 72.82

ATOM 5374 CE1 TYR A 340 -7.706 27.754 123.478 1.00 81.51

ATOM 5375 CE2 TYR A 340 -8.713 25.858 122.407 1.00 77.75

ATOM 5376 CZ TYR A 340 -8.678 26.762 123.448 1.00 82.32

ATOM 5377 OH TYR A 340 -9.642 26.606 124.438 1.00 85.71

ATOM 5378 H TYR A 340 -5.983 29.548 120.091 1.00 0.00

ATOM 5379 H TYR A 340 -4.127 27.658 121.254 1.00 0.00

ATOM 5380 H TYR A 340 -5.484 25.944 120.040 1.00 0.00

ATOM 5381 H TYR A 340 -6.440 27.369 119.328 1.00 0.00

ATOM 5382 H TYR A 340 -6.033 28.609 122.411 1.00 0.00

ATOM 5383 H TYR A 340 -7.860 25.211 120.558 1.00 0.00

ATOM 5384 H TYR A 340 -7.653 28.459 124.307 1.00 0.00

ATOM 5385 H TYR A 340 -9.468 25.072 122.403 1.00 0.00

ATOM 5386 H TYR A 340 -9.475 27.342 125.139 1.00 0.00

ATOM 5387 N ALA A 341 -2.953 26.487 119.252 1.00 68.59

ATOM 5388 CA ALA A 341 -2.220 26.031 118.093 1.00 67.44

ATOM 5389 C ALA A 341 -3.185 25.262 117.202 1.00 64.84

ATOM 5390 O ALA A 341 -4.109 24.632 117.689 1.00 64.52

ATOM 5391 CB ALA A 341 -0.969 25.152 118.497 1.00 69.28

ATOM 5392 H ALA A 341 -2.892 25.980 120.146 1.00 0.00

ATOM 5393 H ALA A 341 -1.818 26.887 117.551 1.00 0.00

ATOM 5394 H ALA A 341 -0.478 24.853 117.571 1.00 0.00

ATOM 5395 H ALA A 341 -0.325 25.779 119.113 1.00 0.00

ATOM 5396 H ALA A 341 -1.348 24.293 119.051 1.00 0.00

ATOM 5397 N VAL A 342 -2.969 25.350 115.893 1.00 63.92

ATOM 5398 CA VAL A 342 -3.727 24.559 114.929 1.00 63.11

ATOM 5399 C VAL A 342 -3.764 23.054 115.308 1.00 64.38

ATOM 5400 O VAL A 342 -4.857 22.479 115.561 1.00 63.88

ATOM 5401 CB VAL A 342 -3.142 24.705 113.523 1.00 62.52

ATOM 5402 CG1 VAL A 342 -3.901 23.808 112.602 1.00 61.91

ATOM 5403 CG2 VAL A 342 -3.240 26.138 113.037 1.00 62.12

ATOM 5404 H VAL A 342 -2.245 25.996 115.549 1.00 0.00

ATOM 5405 H VAL A 342 -4.746 24.944 114.946 1.00 0.00

ATOM 5406 H VAL A 342 -2.087 24.430 113.543 1.00 0.00

ATOM 5407 H VAL A 342 -3.467 23.929 111.610 1.00 0.00

ATOM 5408 H VAL A 342 -3.780 22.791 112.976 1.00 0.00

ATOM 5409 H VAL A 342 -4.942 24.129 112.631 1.00 0.00

ATOM 5410 H VAL A 342 -2.807 26.166 112.037 1.00 0.00

ATOM 5411 H VAL A 342 -4.298 26.400 113.028 1.00 0.00

ATOM 5412 H VAL A 342 -2.676 26.752 113.739 1.00 0.00

ATOM 5413 N SER A 343 -2.570 22.447 115.391 1.00 65.36

ATOM 5414 CA SER A 343 -2.425 21.067 115.695 1.00 66.58

ATOM 5415 C SER A 343 -3.424 20.744 116.760 1.00 67.74

ATOM 5416 O SER A 343 -4.087 19.705 116.700 1.00 68.71

ATOM 5417 CB SER A 343 -1.029 20.828 116.211 1.00 70.74

ATOM 5418 OG SER A 343 -0.099 21.666 115.528 1.00 70.69

ATOM 5419 H SER A 343 -1.721 23.007 115.227 1.00 0.00

ATOM 5420 H SER A 343 -2.588 20.443 114.816 1.00 0.00

ATOM 5421 H SER A 343 -0.730 19.788 116.081 1.00 0.00

ATOM 5422 H SER A 343 -0.989 21.104 117.265 1.00 0.00

ATOM 5423 H SER A 343 0.832 21.462 115.918 1.00 0.00

ATOM 5424 N VAL A 344 -3.582 21.657 117.718 1.00 68.17

ATOM 5425 CA VAL A 344 -4.536 21.476 118.797 1.00 68.54

ATOM 5426 C VAL A 344 -5.993 21.542 118.278 1.00 67.06

ATOM 5427 O VAL A 344 -6.832 20.718 118.691 1.00 67.89

ATOM 5428 CB VAL A 344 -4.298 22.503 119.893 1.00 70.49

ATOM 5429 CG1 VAL A 344 -5.240 22.299 121.086 1.00 73.23

ATOM 5430 CG2 VAL A 344 -2.896 22.416 120.364 1.00 73.73

ATOM 5431 H VAL A 344 -3.012 22.514 117.692 1.00 0.00

ATOM 5432 H VAL A 344 -4.386 20.482 119.219 1.00 0.00

ATOM 5433 H VAL A 344 -4.497 23.487 119.468 1.00 0.00

ATOM 5434 H VAL A 344 -5.001 23.072 121.817 1.00 0.00

ATOM 5435 H VAL A 344 -6.258 22.398 120.709 1.00 0.00

ATOM 5436 H VAL A 344 -5.048 21.299 121.476 1.00 0.00

ATOM 5437 H VAL A 344 -2.775 23.167 121.145 1.00 0.00

ATOM 5438 H VAL A 344 -2.751 21.406 120.747 1.00 0.00

ATOM 5439 H VAL A 344 -2.258 22.619 119.504 1.00 0.00

ATOM 5440 N LEU A 345 -6.312 22.487 117.384 1.00 64.49

ATOM 5441 CA LEU A 345 -7.724 22.639 116.996 1.00 63.42

ATOM 5442 C LEU A 345 -8.082 21.434 116.154 1.00 63.33

ATOM 5443 O LEU A 345 -9.110 20.821 116.379 1.00 64.09

ATOM 5444 CB LEU A 345 -8.031 23.939 116.251 1.00 61.19

ATOM 5445 CG LEU A 345 -7.830 25.289 116.939 1.00 62.13

ATOM 5446 CD1 LEU A 345 -7.655 26.369 115.901 1.00 60.18

ATOM 5447 CD2 LEU A 345 -8.968 25.731 117.945 1.00 64.88

ATOM 5448 H LEU A 345 -5.586 23.094 116.977 1.00 0.00

ATOM 5449 H LEU A 345 -8.329 22.697 117.901 1.00 0.00

ATOM 5450 H LEU A 345 -9.102 23.892 116.056 1.00 0.00

ATOM 5451 H LEU A 345 -7.321 23.950 115.424 1.00 0.00

ATOM 5452 H LEU A 345 -6.937 25.153 117.549 1.00 0.00

ATOM 5453 H LEU A 345 -7.516 27.307 116.438 1.00 0.00

ATOM 5454 H LEU A 345 -6.777 26.105 115.312 1.00 0.00

ATOM 5455 H LEU A 345 -8.561 26.379 115.296 1.00 0.00

ATOM 5456 H LEU A 345 -8.679 26.702 118.346 1.00 0.00

ATOM 5457 H LEU A 345 -9.894 25.788 117.373 1.00 0.00

ATOM 5458 H LEU A 345 -9.019 24.970 118.723 1.00 0.00

ATOM 5459 N LEU A 346 -7.224 21.062 115.201 1.00 62.86

ATOM 5460 CA LEU A 346 -7.494 19.868 114.446 1.00 63.08

ATOM 5461 C LEU A 346 -7.605 18.645 115.288 1.00 65.02

ATOM 5462 O LEU A 346 -8.221 17.679 114.863 1.00 67.16

ATOM 5463 CB LEU A 346 -6.439 19.612 113.420 1.00 64.07

ATOM 5464 CG LEU A 346 -6.040 20.804 112.584 1.00 65.38

ATOM 5465 CD1 LEU A 346 -4.839 20.317 111.774 1.00 67.83

ATOM 5466 CD2 LEU A 346 -7.207 21.276 111.713 1.00 65.80

ATOM 5467 H LEU A 346 -6.381 21.622 115.009 1.00 0.00

ATOM 5468 H LEU A 346 -8.457 20.059 113.972 1.00 0.00

ATOM 5469 H LEU A 346 -6.859 18.878 112.732 1.00 0.00

ATOM 5470 H LEU A 346 -5.550 19.293 113.963 1.00 0.00

ATOM 5471 H LEU A 346 -5.777 21.678 113.180 1.00 0.00

ATOM 5472 H LEU A 346 -4.515 21.151 111.151 1.00 0.00

ATOM 5473 H LEU A 346 -4.071 20.023 112.489 1.00 0.00

ATOM 5474 H LEU A 346 -5.180 19.473 111.175 1.00 0.00

ATOM 5475 H LEU A 346 -6.851 22.133 111.140 1.00 0.00

ATOM 5476 H LEU A 346 -7.480 20.442 111.067 1.00 0.00

ATOM 5477 H LEU A 346 -8.018 21.548 112.388 1.00 0.00

ATOM 5478 N ARG A 347 -6.991 18.647 116.462 1.00 66.37

ATOM 5479 CA ARG A 347 -7.126 17.540 117.386 1.00 67.85

ATOM 5480 C ARG A 347 -8.443 17.619 118.153 1.00 68.34

ATOM 5481 O ARG A 347 -9.002 16.621 118.584 1.00 70.69

ATOM 5482 CB ARG A 347 -5.981 17.626 118.371 1.00 70.64

ATOM 5483 CG ARG A 347 -5.412 16.338 118.837 1.00 70.56

ATOM 5484 CD ARG A 347 -4.409 16.683 119.937 1.00 74.57

ATOM 5485 NE ARG A 347 -3.048 16.796 119.444 1.00 76.77

ATOM 5486 CZ ARG A 347 -2.084 17.528 120.013 1.00 80.00

ATOM 5487 NH1 ARG A 347 -2.348 18.256 121.093 1.00 79.57

ATOM 5488 NH2 ARG A 347 -0.847 17.545 119.497 1.00 78.91

ATOM 5489 H ARG A 347 -6.405 19.453 116.723 1.00 0.00

ATOM 5490 H ARG A 347 -7.112 16.602 116.832 1.00 0.00

ATOM 5491 H ARG A 347 -6.364 18.126 119.261 1.00 0.00

ATOM 5492 H ARG A 347 -5.167 18.125 117.845 1.00 0.00

ATOM 5493 H ARG A 347 -4.922 15.827 118.008 1.00 0.00

ATOM 5494 H ARG A 347 -6.197 15.669 119.190 1.00 0.00

ATOM 5495 H ARG A 347 -4.420 15.911 120.707 1.00 0.00

ATOM 5496 H ARG A 347 -4.666 17.669 120.323 1.00 0.00

ATOM 5497 H ARG A 347 -2.806 16.272 118.591 1.00 0.00

ATOM 5498 H ARG A 347 -1.604 18.819 121.529 1.00 0.00

ATOM 5499 H ARG A 347 -3.297 18.257 121.494 1.00 0.00

ATOM 5500 H ARG A 347 -0.111 18.112 119.942 1.00 0.00

ATOM 5501 H ARG A 347 -0.632 16.991 118.655 1.00 0.00

ATOM 5502 N LEU A 348 -8.895 18.834 118.394 1.00 67.46

ATOM 5503 CA LEU A 348 -10.206 19.057 118.980 1.00 67.32

ATOM 5504 C LEU A 348 -11.354 18.562 118.065 1.00 65.35

ATOM 5505 O LEU A 348 -12.267 17.956 118.549 1.00 66.67

ATOM 5506 CB LEU A 348 -10.394 20.541 119.328 1.00 66.70

ATOM 5507 CG LEU A 348 -9.652 21.170 120.500 1.00 70.62

ATOM 5508 CD1 LEU A 348 -10.178 22.577 120.580 1.00 73.50

ATOM 5509 CD2 LEU A 348 -9.825 20.465 121.891 1.00 74.46

ATOM 5510 H LEU A 348 -8.304 19.645 118.161 1.00 0.00

ATOM 5511 H LEU A 348 -10.252 18.468 119.896 1.00 0.00

ATOM 5512 H LEU A 348 -11.445 20.624 119.604 1.00 0.00

ATOM 5513 H LEU A 348 -10.000 21.069 118.460 1.00 0.00

ATOM 5514 H LEU A 348 -8.582 21.087 120.308 1.00 0.00

ATOM 5515 H LEU A 348 -9.667 23.061 121.412 1.00 0.00

ATOM 5516 H LEU A 348 -9.949 23.057 119.629 1.00 0.00

ATOM 5517 H LEU A 348 -11.252 22.506 120.750 1.00 0.00

ATOM 5518 H LEU A 348 -9.237 21.033 122.612 1.00 0.00

ATOM 5519 H LEU A 348 -10.888 20.486 122.129 1.00 0.00

ATOM 5520 H LEU A 348 -9.455 19.446 121.780 1.00 0.00

ATOM 5521 N ALA A 349 -11.277 18.819 116.767 1.00 62.81

ATOM 5522 CA ALA A 349 -12.307 18.454 115.830 1.00 62.41

ATOM 5523 C ALA A 349 -12.338 16.960 115.579 1.00 63.78

ATOM 5524 O ALA A 349 -13.397 16.387 115.582 1.00 64.40

ATOM 5525 CB ALA A 349 -12.070 19.174 114.548 1.00 61.56

ATOM 5526 H ALA A 349 -10.440 19.303 116.412 1.00 0.00

ATOM 5527 H ALA A 349 -13.271 18.735 116.254 1.00 0.00

ATOM 5528 H ALA A 349 -12.864 18.876 113.864 1.00 0.00

ATOM 5529 H ALA A 349 -12.105 20.241 114.770 1.00 0.00

ATOM 5530 H ALA A 349 -11.088 18.867 114.187 1.00 0.00

ATOM 5531 N LYS A 350 -11.176 16.349 115.302 1.00 64.36

ATOM 5532 CA LYS A 350 -11.016 14.877 115.238 1.00 65.81

ATOM 5533 C LYS A 350 -11.705 14.111 116.385 1.00 67.24

ATOM 5534 O LYS A 350 -12.319 13.063 116.188 1.00 68.84

ATOM 5535 CB LYS A 350 -9.537 14.550 115.290 1.00 66.21

ATOM 5536 CG LYS A 350 -9.180 13.103 115.088 1.00 68.71

ATOM 5537 CD LYS A 350 -8.985 12.706 113.608 1.00 68.90

ATOM 5538 CE LYS A 350 -8.674 11.167 113.462 1.00 66.96

ATOM 5539 NZ LYS A 350 -7.186 10.902 113.577 1.00 67.96

ATOM 5540 H LYS A 350 -10.349 16.936 115.124 1.00 0.00

ATOM 5541 H LYS A 350 -11.492 14.560 114.310 1.00 0.00

ATOM 5542 H LYS A 350 -9.191 14.816 116.289 1.00 0.00

ATOM 5543 H LYS A 350 -9.072 15.094 114.467 1.00 0.00

ATOM 5544 H LYS A 350 -10.023 12.526 115.469 1.00 0.00

ATOM 5545 H LYS A 350 -8.245 12.924 115.619 1.00 0.00

ATOM 5546 H LYS A 350 -8.135 13.269 113.223 1.00 0.00

ATOM 5547 H LYS A 350 -9.898 12.944 113.063 1.00 0.00

ATOM 5548 H LYS A 350 -9.021 10.799 112.496 1.00 0.00

ATOM 5549 H LYS A 350 -9.160 10.633 114.279 1.00 0.00

ATOM 5550 H LYS A 350 -7.052 9.886 113.474 1.00 0.00

ATOM 5551 H LYS A 350 -6.727 11.428 112.819 1.00 0.00

ATOM 5552 H LYS A 350 -6.892 11.234 114.506 1.00 0.00

ATOM 5553 N GLU A 351 -11.611 14.665 117.582 1.00 67.62

ATOM 5554 CA GLU A 351 -12.136 14.029 118.786 1.00 69.67

ATOM 5555 C GLU A 351 -13.635 14.228 118.762 1.00 69.19

ATOM 5556 O GLU A 351 -14.420 13.326 119.149 1.00 71.58

ATOM 5557 CB GLU A 351 -11.490 14.669 120.011 1.00 70.88

ATOM 5558 CG GLU A 351 -12.100 14.317 121.374 1.00 77.01

ATOM 5559 CD GLU A 351 -11.426 13.135 122.099 1.00 82.59

ATOM 5560 OE1 GLU A 351 -10.408 12.592 121.600 1.00 84.45

ATOM 5561 OE2 GLU A 351 -11.940 12.728 123.169 1.00 85.95

ATOM 5562 H GLU A 351 -11.149 15.582 117.668 1.00 0.00

ATOM 5563 H GLU A 351 -11.911 12.963 118.827 1.00 0.00

ATOM 5564 H GLU A 351 -11.626 15.744 119.890 1.00 0.00

ATOM 5565 H GLU A 351 -10.463 14.305 120.034 1.00 0.00

ATOM 5566 H GLU A 351 -13.134 14.026 121.188 1.00 0.00

ATOM 5567 H GLU A 351 -11.979 15.194 122.010 1.00 0.00

ATOM 5568 N TYR A 352 -14.020 15.402 118.255 1.00 66.42

ATOM 5569 CA TYR A 352 -15.395 15.776 118.100 1.00 65.32

ATOM 5570 C TYR A 352 -16.055 14.835 117.068 1.00 64.60

ATOM 5571 O TYR A 352 -17.111 14.270 117.325 1.00 65.63

ATOM 5572 CB TYR A 352 -15.548 17.277 117.779 1.00 63.26

ATOM 5573 CG TYR A 352 -16.993 17.622 117.610 1.00 66.05

ATOM 5574 CD1 TYR A 352 -17.571 17.704 116.363 1.00 66.86

ATOM 5575 CD2 TYR A 352 -17.829 17.765 118.714 1.00 70.69

ATOM 5576 CE1 TYR A 352 -18.934 17.959 116.229 1.00 67.59

ATOM 5577 CE2 TYR A 352 -19.182 18.030 118.574 1.00 69.73

ATOM 5578 CZ TYR A 352 -19.720 18.127 117.336 1.00 68.31

ATOM 5579 OH TYR A 352 -21.061 18.387 117.200 1.00 71.53

ATOM 5580 H TYR A 352 -13.294 16.071 117.961 1.00 0.00

ATOM 5581 H TYR A 352 -15.927 15.650 119.043 1.00 0.00

ATOM 5582 H TYR A 352 -15.012 17.527 116.864 1.00 0.00

ATOM 5583 H TYR A 352 -15.159 17.862 118.613 1.00 0.00

ATOM 5584 H TYR A 352 -16.957 17.568 115.473 1.00 0.00

ATOM 5585 H TYR A 352 -17.408 17.666 119.715 1.00 0.00

ATOM 5586 H TYR A 352 -19.377 18.025 115.235 1.00 0.00

ATOM 5587 H TYR A 352 -19.809 18.160 119.456 1.00 0.00

ATOM 5588 H TYR A 352 -21.262 18.413 116.190 1.00 0.00

ATOM 5589 N GLU A 353 -15.391 14.596 115.944 1.00 63.53

ATOM 5590 CA GLU A 353 -15.833 13.557 115.015 1.00 63.90

ATOM 5591 C GLU A 353 -16.023 12.217 115.684 1.00 65.64

ATOM 5592 O GLU A 353 -17.049 11.560 115.490 1.00 67.29

ATOM 5593 CB GLU A 353 -14.830 13.360 113.905 1.00 63.65

ATOM 5594 CG GLU A 353 -15.309 12.452 112.775 1.00 64.89

ATOM 5595 CD GLU A 353 -14.349 12.500 111.599 1.00 71.37

ATOM 5596 OE1 GLU A 353 -13.117 12.576 111.874 1.00 71.01

ATOM 5597 OE2 GLU A 353 -14.804 12.456 110.412 1.00 74.28

ATOM 5598 H GLU A 353 -14.552 15.151 115.723 1.00 0.00

ATOM 5599 H GLU A 353 -16.789 13.907 114.625 1.00 0.00

ATOM 5600 H GLU A 353 -13.960 12.880 114.354 1.00 0.00

ATOM 5601 H GLU A 353 -14.631 14.342 113.476 1.00 0.00

ATOM 5602 H GLU A 353 -16.290 12.789 112.441 1.00 0.00

ATOM 5603 H GLU A 353 -15.355 11.427 113.144 1.00 0.00

ATOM 5604 N ALA A 354 -15.036 11.797 116.467 1.00 65.11

ATOM 5605 CA ALA A 354 -15.109 10.461 117.012 1.00 66.74

ATOM 5606 C ALA A 354 -16.386 10.302 117.881 1.00 66.86

ATOM 5607 O ALA A 354 -16.978 9.223 118.003 1.00 68.37

ATOM 5608 CB ALA A 354 -13.822 10.129 117.778 1.00 67.39

ATOM 5609 H ALA A 354 -14.236 12.410 116.681 1.00 0.00

ATOM 5610 H ALA A 354 -15.189 9.739 116.199 1.00 0.00

ATOM 5611 H ALA A 354 -13.933 9.116 118.164 1.00 0.00

ATOM 5612 H ALA A 354 -13.000 10.203 117.067 1.00 0.00

ATOM 5613 H ALA A 354 -13.731 10.861 118.581 1.00 0.00

ATOM 5614 N THR A 355 -16.814 11.417 118.434 1.00 65.26

ATOM 5615 CA THR A 355 -17.894 11.439 119.403 1.00 66.53

ATOM 5616 C THR A 355 -19.267 11.300 118.698 1.00 66.19

ATOM 5617 O THR A 355 -20.129 10.565 119.154 1.00 67.04

ATOM 5618 CB THR A 355 -17.801 12.718 120.343 1.00 66.99

ATOM 5619 OG1 THR A 355 -16.537 12.737 121.024 1.00 67.93

ATOM 5620 CG2 THR A 355 -18.919 12.766 121.375 1.00 67.80

ATOM 5621 H THR A 355 -16.366 12.306 118.169 1.00 0.00

ATOM 5622 H THR A 355 -17.791 10.575 120.059 1.00 0.00

ATOM 5623 H THR A 355 -17.901 13.591 119.698 1.00 0.00

ATOM 5624 H THR A 355 -15.805 12.704 120.301 1.00 0.00

ATOM 5625 H THR A 355 -18.769 13.670 121.965 1.00 0.00

ATOM 5626 H THR A 355 -19.860 12.792 120.825 1.00 0.00

ATOM 5627 H THR A 355 -18.830 11.866 121.983 1.00 0.00

ATOM 5628 N LEU A 356 -19.461 11.978 117.584 1.00 64.03

ATOM 5629 CA LEU A 356 -20.684 11.740 116.871 1.00 65.92

ATOM 5630 C LEU A 356 -20.709 10.310 116.268 1.00 67.43

ATOM 5631 O LEU A 356 -21.728 9.647 116.350 1.00 67.82

ATOM 5632 CB LEU A 356 -20.916 12.810 115.800 1.00 65.01

ATOM 5633 CG LEU A 356 -20.660 14.295 116.080 1.00 63.03

ATOM 5634 CD1 LEU A 356 -21.338 15.049 114.977 1.00 61.90

ATOM 5635 CD2 LEU A 356 -21.222 14.759 117.412 1.00 63.67

ATOM 5636 H LEU A 356 -18.763 12.652 117.239 1.00 0.00

ATOM 5637 H LEU A 356 -21.507 11.808 117.582 1.00 0.00

ATOM 5638 H LEU A 356 -21.989 12.762 115.614 1.00 0.00

ATOM 5639 H LEU A 356 -20.191 12.563 115.025 1.00 0.00

ATOM 5640 H LEU A 356 -19.585 14.469 116.125 1.00 0.00

ATOM 5641 H LEU A 356 -21.165 16.109 115.162 1.00 0.00

ATOM 5642 H LEU A 356 -20.884 14.722 114.041 1.00 0.00

ATOM 5643 H LEU A 356 -22.398 14.797 115.023 1.00 0.00

ATOM 5644 H LEU A 356 -20.985 15.819 117.506 1.00 0.00

ATOM 5645 H LEU A 356 -22.298 14.586 117.382 1.00 0.00

ATOM 5646 H LEU A 356 -20.737 14.165 118.187 1.00 0.00

ATOM 5647 N GLU A 357 -19.610 9.828 115.679 1.00 67.63

ATOM 5648 CA GLU A 357 -19.641 8.487 115.090 1.00 72.99

ATOM 5649 C GLU A 357 -20.255 7.459 116.048 1.00 76.70

ATOM 5650 O GLU A 357 -21.144 6.665 115.686 1.00 78.78

ATOM 5651 CB GLU A 357 -18.253 8.030 114.692 1.00 73.59

ATOM 5652 CG GLU A 357 -17.644 8.847 113.516 1.00 74.11

ATOM 5653 CD GLU A 357 -18.308 8.475 112.226 1.00 76.32

ATOM 5654 OE1 GLU A 357 -18.291 7.273 111.949 1.00 81.92

ATOM 5655 OE2 GLU A 357 -18.873 9.338 111.515 1.00 76.72

ATOM 5656 H GLU A 357 -18.750 10.394 115.640 1.00 0.00

ATOM 5657 H GLU A 357 -20.267 8.553 114.200 1.00 0.00

ATOM 5658 H GLU A 357 -18.354 6.998 114.356 1.00 0.00

ATOM 5659 H GLU A 357 -17.609 8.142 115.564 1.00 0.00

ATOM 5660 H GLU A 357 -16.574 8.659 113.431 1.00 0.00

ATOM 5661 H GLU A 357 -17.830 9.907 113.687 1.00 0.00

ATOM 5662 N GLU A 358 -19.814 7.531 117.294 1.00 78.37

ATOM 5663 CA GLU A 358 -20.141 6.540 118.272 1.00 81.99

ATOM 5664 C GLU A 358 -21.401 6.911 119.080 1.00 84.30

ATOM 5665 O GLU A 358 -22.163 6.011 119.495 1.00 87.25

ATOM 5666 CB GLU A 358 -18.905 6.302 119.128 1.00 82.68

ATOM 5667 CG GLU A 358 -18.837 7.123 120.363 1.00 84.11

ATOM 5668 CD GLU A 358 -19.312 6.316 121.531 1.00 87.44

ATOM 5669 OE1 GLU A 358 -18.531 5.474 122.011 1.00 88.67

ATOM 5670 OE2 GLU A 358 -20.474 6.506 121.949 1.00 91.13

ATOM 5671 H GLU A 358 -19.217 8.325 117.567 1.00 0.00

ATOM 5672 H GLU A 358 -20.411 5.602 117.786 1.00 0.00

ATOM 5673 H GLU A 358 -18.029 6.555 118.531 1.00 0.00

ATOM 5674 H GLU A 358 -18.946 5.265 119.462 1.00 0.00

ATOM 5675 H GLU A 358 -19.476 7.998 120.244 1.00 0.00

ATOM 5676 H GLU A 358 -17.808 7.441 120.530 1.00 0.00

ATOM 5677 N CYS A 359 -21.644 8.213 119.276 1.00 83.27

ATOM 5678 CA CYS A 359 -22.870 8.665 119.943 1.00 85.78

ATOM 5679 C CYS A 359 -24.068 8.477 119.034 1.00 86.77

ATOM 5680 O CYS A 359 -25.135 8.007 119.463 1.00 89.63

ATOM 5681 CB CYS A 359 -22.806 10.130 120.389 1.00 84.77

ATOM 5682 SG CYS A 359 -21.764 10.580 121.874 1.00 88.50

ATOM 5683 H CYS A 359 -20.958 8.910 118.953 1.00 0.00

ATOM 5684 H CYS A 359 -22.972 8.051 120.838 1.00 0.00

ATOM 5685 H CYS A 359 -23.826 10.206 120.766 1.00 0.00

ATOM 5686 H CYS A 359 -22.540 10.660 119.474 1.00 0.00

ATOM 5687 N CYS A 360 -23.902 8.842 117.774 1.00 85.54

ATOM 5688 CA CYS A 360 -24.999 8.705 116.801 1.00 87.75

ATOM 5689 C CYS A 360 -25.428 7.257 116.628 1.00 89.33

ATOM 5690 O CYS A 360 -26.596 6.990 116.339 1.00 92.87

ATOM 5691 CB CYS A 360 -24.711 9.415 115.463 1.00 86.50

ATOM 5692 SG CYS A 360 -24.602 11.300 115.577 1.00 88.88

ATOM 5693 H CYS A 360 -22.996 9.226 117.470 1.00 0.00

ATOM 5694 H CYS A 360 -25.855 9.231 117.224 1.00 0.00

ATOM 5695 H CYS A 360 -25.650 9.209 114.949 1.00 0.00

ATOM 5696 H CYS A 360 -23.842 8.906 115.045 1.00 0.00

ATOM 5697 N ALA A 361 -24.519 6.329 116.899 1.00 88.29

ATOM 5698 CA ALA A 361 -24.877 4.914 116.976 1.00 90.80

ATOM 5699 C ALA A 361 -25.924 4.625 118.064 1.00 93.12

ATOM 5700 O ALA A 361 -26.950 4.053 117.773 1.00 95.89

ATOM 5701 CB ALA A 361 -23.632 4.032 117.151 1.00 90.32

ATOM 5702 H ALA A 361 -23.541 6.612 117.058 1.00 0.00

ATOM 5703 H ALA A 361 -25.341 4.659 116.023 1.00 0.00

ATOM 5704 H ALA A 361 -23.977 2.999 117.200 1.00 0.00

ATOM 5705 H ALA A 361 -22.994 4.205 116.284 1.00 0.00

ATOM 5706 H ALA A 361 -23.149 4.342 118.078 1.00 0.00

ATOM 5707 N LYS A 362 -25.670 5.044 119.299 1.00 93.04

ATOM 5708 CA LYS A 362 -26.550 4.761 120.455 1.00 96.43

ATOM 5709 C LYS A 362 -28.024 5.216 120.321 1.00 99.10

ATOM 5710 O LYS A 362 -28.328 6.183 119.605 1.00 98.31

ATOM 5711 CB LYS A 362 -26.064 5.543 121.685 1.00 95.52

ATOM 5712 CG LYS A 362 -24.748 5.068 122.305 1.00 94.54

ATOM 5713 CD LYS A 362 -24.143 6.106 123.310 1.00 95.07

ATOM 5714 CE LYS A 362 -24.728 6.038 124.762 1.00 96.49

ATOM 5715 NZ LYS A 362 -25.722 7.121 125.142 1.00 95.65

ATOM 5716 H LYS A 362 -24.815 5.595 119.461 1.00 0.00

ATOM 5717 H LYS A 362 -26.505 3.674 120.528 1.00 0.00

ATOM 5718 H LYS A 362 -26.827 5.417 122.453 1.00 0.00

ATOM 5719 H LYS A 362 -25.889 6.565 121.348 1.00 0.00

ATOM 5720 H LYS A 362 -24.039 4.943 121.487 1.00 0.00

ATOM 5721 H LYS A 362 -24.946 4.137 122.837 1.00 0.00

ATOM 5722 H LYS A 362 -24.380 7.096 122.921 1.00 0.00

ATOM 5723 H LYS A 362 -23.078 5.886 123.388 1.00 0.00

ATOM 5724 H LYS A 362 -23.894 6.138 125.456 1.00 0.00

ATOM 5725 H LYS A 362 -25.296 5.109 124.815 1.00 0.00

ATOM 5726 H LYS A 362 -26.009 6.939 126.114 1.00 0.00

ATOM 5727 H LYS A 362 -25.234 8.023 125.050 1.00 0.00

ATOM 5728 H LYS A 362 -26.514 7.045 124.488 1.00 0.00

ATOM 5729 N ASP A 363 -28.958 4.612 121.039 1.00102.84

ATOM 5730 CA ASP A 363 -30.341 5.112 120.948 1.00105.86

ATOM 5731 C ASP A 363 -30.599 6.524 121.437 1.00105.61

ATOM 5732 O ASP A 363 -31.474 7.193 120.930 1.00106.76

ATOM 5733 CB ASP A 363 -31.404 4.173 121.500 1.00110.44

ATOM 5734 CG ASP A 363 -32.771 4.449 120.886 1.00114.94

ATOM 5735 OD1 ASP A 363 -32.951 4.135 119.694 1.00115.43

ATOM 5736 OD2 ASP A 363 -33.660 5.001 121.569 1.00118.38

ATOM 5737 H ASP A 363 -28.724 3.813 121.646 1.00 0.00

ATOM 5738 H ASP A 363 -30.443 5.149 119.863 1.00 0.00

ATOM 5739 H ASP A 363 -31.473 4.328 122.577 1.00 0.00

ATOM 5740 H ASP A 363 -31.118 3.149 121.259 1.00 0.00

ATOM 5741 N ASP A 364 -29.903 6.944 122.474 1.00104.90

ATOM 5742 CA ASP A 364 -30.087 8.291 123.023 1.00104.74

ATOM 5743 C ASP A 364 -28.811 9.126 122.799 1.00100.56

ATOM 5744 O ASP A 364 -28.087 9.448 123.759 1.00 99.98

ATOM 5745 CB ASP A 364 -30.437 8.204 124.507 1.00108.47

ATOM 5746 CG ASP A 364 -30.832 9.541 125.100 1.00110.66

ATOM 5747 OD1 ASP A 364 -30.754 10.577 124.394 1.00107.87

ATOM 5748 OD2 ASP A 364 -31.229 9.543 126.287 1.00116.05

ATOM 5749 H ASP A 364 -29.215 6.312 122.908 1.00 0.00

ATOM 5750 H ASP A 364 -30.911 8.785 122.509 1.00 0.00

ATOM 5751 H ASP A 364 -29.556 7.850 125.043 1.00 0.00

ATOM 5752 H ASP A 364 -31.293 7.536 124.608 1.00 0.00

ATOM 5753 N PRO A 365 -28.530 9.476 121.521 1.00 97.53

ATOM 5754 CA PRO A 365 -27.325 10.228 121.175 1.00 93.78

ATOM 5755 C PRO A 365 -27.181 11.502 122.007 1.00 94.44

ATOM 5756 O PRO A 365 -26.081 11.815 122.471 1.00 92.41

ATOM 5757 CB PRO A 365 -27.536 10.555 119.685 1.00 92.47

ATOM 5758 CG PRO A 365 -29.023 10.273 119.432 1.00 95.76

ATOM 5759 CD PRO A 365 -29.305 9.137 120.315 1.00 97.63

ATOM 5760 H PRO A 365 -26.411 9.668 121.372 1.00 0.00

ATOM 5761 H PRO A 365 -26.893 9.957 119.040 1.00 0.00

ATOM 5762 H PRO A 365 -27.275 11.588 119.454 1.00 0.00

ATOM 5763 H PRO A 365 -29.272 10.066 118.391 1.00 0.00

ATOM 5764 H PRO A 365 -29.658 11.124 119.677 1.00 0.00

ATOM 5765 H PRO A 365 -30.369 8.999 120.510 1.00 0.00

ATOM 5766 H PRO A 365 -29.022 8.179 119.879 1.00 0.00

ATOM 5767 N HIS A 366 -28.293 12.200 122.225 1.00 97.46

ATOM 5768 CA HIS A 366 -28.268 13.478 122.933 1.00 98.66

ATOM 5769 C HIS A 366 -27.708 13.368 124.371 1.00100.33

ATOM 5770 O HIS A 366 -27.048 14.302 124.846 1.00100.15

ATOM 5771 CB HIS A 366 -29.663 14.143 122.924 1.00102.91

ATOM 5772 CG HIS A 366 -29.633 15.592 123.297 1.00103.32

ATOM 5773 ND1 HIS A 366 -29.701 16.025 124.602 1.00105.93

ATOM 5774 CD2 HIS A 366 -29.503 16.705 122.537 1.00101.36

ATOM 5775 CE1 HIS A 366 -29.619 17.343 124.629 1.00107.66

ATOM 5776 NE2 HIS A 366 -29.497 17.780 123.389 1.00104.37

ATOM 5777 H HIS A 366 -29.193 11.830 121.887 1.00 0.00

ATOM 5778 H HIS A 366 -27.575 14.118 122.387 1.00 0.00

ATOM 5779 H HIS A 366 -30.303 13.632 123.644 1.00 0.00

ATOM 5780 H HIS A 366 -30.040 14.099 121.902 1.00 0.00

ATOM 5781 H HIS A 366 -29.800 15.417 125.427 1.00 0.00

ATOM 5782 H HIS A 366 -29.419 16.740 121.451 1.00 0.00

ATOM 5783 H HIS A 366 -29.647 17.965 125.524 1.00 0.00

ATOM 5784 N ALA A 367 -27.977 12.246 125.054 1.00 98.92

ATOM 5785 CA ALA A 367 -27.371 11.968 126.372 1.00103.07

ATOM 5786 C ALA A 367 -25.868 11.761 126.233 1.00102.80

ATOM 5787 O ALA A 367 -25.075 12.111 127.112 1.00103.73

ATOM 5788 CB ALA A 367 -27.994 10.747 127.011 1.00109.26

ATOM 5789 H ALA A 367 -28.626 11.557 124.647 1.00 0.00

ATOM 5790 H ALA A 367 -27.559 12.830 127.012 1.00 0.00

ATOM 5791 H ALA A 367 -27.501 10.604 127.973 1.00 0.00

ATOM 5792 H ALA A 367 -29.057 10.956 127.128 1.00 0.00

ATOM 5793 H ALA A 367 -27.817 9.909 126.337 1.00 0.00

ATOM 5794 N CYS A 368 -25.483 11.207 125.094 1.00101.85

ATOM 5795 CA CYS A 368 -24.087 10.912 124.843 1.00102.33

ATOM 5796 C CYS A 368 -23.265 12.141 124.416 1.00 96.66

ATOM 5797 O CYS A 368 -22.173 12.357 124.940 1.00 96.71

ATOM 5798 CB CYS A 368 -23.955 9.775 123.831 1.00105.69

ATOM 5799 SG CYS A 368 -22.244 9.358 123.454 1.00109.83

ATOM 5800 H CYS A 368 -26.186 10.982 124.376 1.00 0.00

ATOM 5801 H CYS A 368 -23.661 10.592 125.794 1.00 0.00

ATOM 5802 H CYS A 368 -24.371 10.236 122.935 1.00 0.00

ATOM 5803 H CYS A 368 -24.558 8.948 124.207 1.00 0.00

ATOM 5804 N TYR A 369 -23.772 12.933 123.475 1.00 92.44

ATOM 5805 CA TYR A 369 -22.961 14.010 122.894 1.00 89.28

ATOM 5806 C TYR A 369 -23.164 15.395 123.550 1.00 87.89

ATOM 5807 O TYR A 369 -22.327 16.264 123.391 1.00 87.24

ATOM 5808 CB TYR A 369 -23.122 14.076 121.360 1.00 89.37

ATOM 5809 CG TYR A 369 -24.445 14.650 120.894 1.00 88.81

ATOM 5810 CD1 TYR A 369 -25.360 13.855 120.237 1.00 90.29

ATOM 5811 CD2 TYR A 369 -24.793 15.992 121.142 1.00 88.13

ATOM 5812 CE1 TYR A 369 -26.596 14.362 119.831 1.00 91.60

ATOM 5813 CE2 TYR A 369 -26.029 16.507 120.732 1.00 88.34

ATOM 5814 CZ TYR A 369 -26.923 15.683 120.073 1.00 89.83

ATOM 5815 OH TYR A 369 -28.152 16.146 119.655 1.00 89.80

ATOM 5816 H TYR A 369 -24.740 12.790 123.152 1.00 0.00

ATOM 5817 H TYR A 369 -21.929 13.742 123.121 1.00 0.00

ATOM 5818 H TYR A 369 -23.061 13.059 120.973 1.00 0.00

ATOM 5819 H TYR A 369 -22.349 14.747 120.986 1.00 0.00

ATOM 5820 H TYR A 369 -25.114 12.813 120.031 1.00 0.00

ATOM 5821 H TYR A 369 -24.088 16.641 121.662 1.00 0.00

ATOM 5822 H TYR A 369 -27.307 13.712 119.320 1.00 0.00

ATOM 5823 H TYR A 369 -26.284 17.548 120.931 1.00 0.00

ATOM 5824 H TYR A 369 -28.634 15.355 119.204 1.00 0.00

ATOM 5825 N SER A 370 -24.273 15.589 124.273 1.00 88.63

ATOM 5826 CA SER A 370 -24.491 16.773 125.149 1.00 89.45

ATOM 5827 C SER A 370 -23.298 17.137 126.053 1.00 91.18

ATOM 5828 O SER A 370 -23.130 18.317 126.428 1.00 92.97

ATOM 5829 CB SER A 370 -25.692 16.537 126.072 1.00 92.12

ATOM 5830 OG SER A 370 -25.405 15.502 126.991 1.00 90.74

ATOM 5831 H SER A 370 -25.018 14.879 124.221 1.00 0.00

ATOM 5832 H SER A 370 -24.650 17.598 124.454 1.00 0.00

ATOM 5833 H SER A 370 -26.597 16.280 125.522 1.00 0.00

ATOM 5834 H SER A 370 -25.858 17.432 126.672 1.00 0.00

ATOM 5835 H SER A 370 -26.240 15.385 127.583 1.00 0.00

ATOM 5836 N THR A 371 -22.500 16.127 126.415 1.00 90.89

ATOM 5837 CA THR A 371 -21.401 16.308 127.364 1.00 93.02

ATOM 5838 C THR A 371 -20.013 16.453 126.722 1.00 90.04

ATOM 5839 O THR A 371 -19.032 16.692 127.420 1.00 91.73

ATOM 5840 CB THR A 371 -21.403 15.189 128.442 1.00 97.81

ATOM 5841 OG1 THR A 371 -21.672 13.923 127.834 1.00 98.45

ATOM 5842 CG2 THR A 371 -22.487 15.444 129.473 1.00102.32

ATOM 5843 H THR A 371 -22.664 15.193 126.013 1.00 0.00

ATOM 5844 H THR A 371 -21.595 17.268 127.842 1.00 0.00

ATOM 5845 H THR A 371 -20.423 15.186 128.920 1.00 0.00

ATOM 5846 H THR A 371 -20.931 13.760 127.137 1.00 0.00

ATOM 5847 H THR A 371 -22.437 14.630 130.196 1.00 0.00

ATOM 5848 H THR A 371 -22.269 16.408 129.932 1.00 0.00

ATOM 5849 H THR A 371 -23.438 15.454 128.940 1.00 0.00

ATOM 5850 N VAL A 372 -19.941 16.359 125.391 1.00 86.94

ATOM 5851 CA VAL A 372 -18.660 16.361 124.649 1.00 84.59

ATOM 5852 C VAL A 372 -17.665 17.485 125.038 1.00 85.05

ATOM 5853 O VAL A 372 -16.470 17.230 125.187 1.00 84.74

ATOM 5854 CB VAL A 372 -18.892 16.285 123.110 1.00 83.33

ATOM 5855 CG1 VAL A 372 -19.649 17.475 122.600 1.00 82.67

ATOM 5856 CG2 VAL A 372 -17.585 16.135 122.371 1.00 83.50

ATOM 5857 H VAL A 372 -20.818 16.282 124.856 1.00 0.00

ATOM 5858 H VAL A 372 -18.156 15.448 124.968 1.00 0.00

ATOM 5859 H VAL A 372 -19.500 15.400 122.922 1.00 0.00

ATOM 5860 H VAL A 372 -19.766 17.341 121.525 1.00 0.00

ATOM 5861 H VAL A 372 -20.610 17.486 123.114 1.00 0.00

ATOM 5862 H VAL A 372 -19.053 18.357 122.836 1.00 0.00

ATOM 5863 H VAL A 372 -17.823 16.089 121.308 1.00 0.00

ATOM 5864 H VAL A 372 -16.980 17.009 122.613 1.00 0.00

ATOM 5865 H VAL A 372 -17.124 15.212 122.723 1.00 0.00

ATOM 5866 N PHE A 373 -18.161 18.711 125.242 1.00 86.08

ATOM 5867 CA PHE A 373 -17.276 19.852 125.486 1.00 87.03

ATOM 5868 C PHE A 373 -16.368 19.755 126.721 1.00 88.51

ATOM 5869 O PHE A 373 -15.273 20.305 126.762 1.00 87.87

ATOM 5870 CB PHE A 373 -18.073 21.164 125.476 1.00 90.46

ATOM 5871 CG PHE A 373 -18.376 21.685 124.083 1.00 89.48

ATOM 5872 CD1 PHE A 373 -19.567 21.358 123.442 1.00 87.55

ATOM 5873 CD2 PHE A 373 -17.463 22.481 123.406 1.00 89.67

ATOM 5874 CE1 PHE A 373 -19.837 21.829 122.160 1.00 88.58

ATOM 5875 CE2 PHE A 373 -17.732 22.951 122.120 1.00 90.19

ATOM 5876 CZ PHE A 373 -18.915 22.617 121.497 1.00 89.88

ATOM 5877 H PHE A 373 -19.181 18.854 125.227 1.00 0.00

ATOM 5878 H PHE A 373 -16.573 19.836 124.653 1.00 0.00

ATOM 5879 H PHE A 373 -17.476 21.921 125.984 1.00 0.00

ATOM 5880 H PHE A 373 -19.032 20.966 125.956 1.00 0.00

ATOM 5881 H PHE A 373 -20.296 20.726 123.949 1.00 0.00

ATOM 5882 H PHE A 373 -16.520 22.743 123.887 1.00 0.00

ATOM 5883 H PHE A 373 -20.780 21.575 121.677 1.00 0.00

ATOM 5884 H PHE A 373 -17.006 23.583 121.608 1.00 0.00

ATOM 5885 H PHE A 373 -19.122 22.972 120.488 1.00 0.00

ATOM 5886 N ASP A 374 -16.818 19.057 127.737 1.00 91.19

ATOM 5887 CA ASP A 374 -15.967 18.888 128.888 1.00 95.50

ATOM 5888 C ASP A 374 -14.813 18.002 128.444 1.00 93.43

ATOM 5889 O ASP A 374 -13.636 18.391 128.556 1.00 93.60

ATOM 5890 CB ASP A 374 -16.796 18.325 130.003 1.00100.24

ATOM 5891 CG ASP A 374 -18.190 18.964 130.040 1.00103.66

ATOM 5892 OD1 ASP A 374 -18.657 19.382 128.957 1.00 99.94

ATOM 5893 OD2 ASP A 374 -18.812 19.071 131.128 1.00111.64

ATOM 5894 H ASP A 374 -17.759 18.639 127.713 1.00 0.00

ATOM 5895 H ASP A 374 -15.542 19.816 129.271 1.00 0.00

ATOM 5896 H ASP A 374 -16.286 18.551 130.940 1.00 0.00

ATOM 5897 H ASP A 374 -16.893 17.250 129.854 1.00 0.00

ATOM 5898 N LYS A 375 -15.161 16.854 127.853 1.00 92.46

ATOM 5899 CA LYS A 375 -14.202 15.968 127.209 1.00 90.74

ATOM 5900 C LYS A 375 -13.199 16.798 126.459 1.00 88.49

ATOM 5901 O LYS A 375 -12.006 16.592 126.585 1.00 89.40

ATOM 5902 CB LYS A 375 -14.903 15.020 126.222 1.00 89.89

ATOM 5903 CG LYS A 375 -15.230 13.635 126.781 1.00 93.73

ATOM 5904 CD LYS A 375 -15.485 12.551 125.680 1.00 92.61

ATOM 5905 CE LYS A 375 -14.309 12.342 124.677 1.00 92.32

ATOM 5906 NZ LYS A 375 -12.934 12.111 125.255 1.00 86.51

ATOM 5907 H LYS A 375 -16.155 16.584 127.853 1.00 0.00

ATOM 5908 H LYS A 375 -13.711 15.370 127.977 1.00 0.00

ATOM 5909 H LYS A 375 -14.222 14.869 125.385 1.00 0.00

ATOM 5910 H LYS A 375 -15.855 15.483 125.962 1.00 0.00

ATOM 5911 H LYS A 375 -16.154 13.744 127.348 1.00 0.00

ATOM 5912 H LYS A 375 -14.378 13.319 127.382 1.00 0.00

ATOM 5913 H LYS A 375 -16.342 12.887 125.095 1.00 0.00

ATOM 5914 H LYS A 375 -15.625 11.602 126.198 1.00 0.00

ATOM 5915 H LYS A 375 -14.228 13.253 124.084 1.00 0.00

ATOM 5916 H LYS A 375 -14.536 11.416 124.148 1.00 0.00

ATOM 5917 H LYS A 375 -12.289 11.995 124.460 1.00 0.00

ATOM 5918 H LYS A 375 -12.701 12.941 125.819 1.00 0.00

ATOM 5919 H LYS A 375 -12.989 11.259 125.832 1.00 0.00

ATOM 5920 N LEU A 376 -13.693 17.736 125.663 1.00 87.71

ATOM 5921 CA LEU A 376 -12.835 18.578 124.835 1.00 87.08

ATOM 5922 C LEU A 376 -12.018 19.490 125.716 1.00 88.33

ATOM 5923 O LEU A 376 -10.807 19.474 125.656 1.00 87.83

ATOM 5924 CB LEU A 376 -13.675 19.442 123.879 1.00 88.40

ATOM 5925 CG LEU A 376 -14.409 18.821 122.683 1.00 88.64

ATOM 5926 CD1 LEU A 376 -15.422 19.823 122.078 1.00 91.78

ATOM 5927 CD2 LEU A 376 -13.436 18.376 121.634 1.00 89.96

ATOM 5928 H LEU A 376 -14.713 17.875 125.628 1.00 0.00

ATOM 5929 H LEU A 376 -12.183 17.929 124.251 1.00 0.00

ATOM 5930 H LEU A 376 -12.948 20.113 123.421 1.00 0.00

ATOM 5931 H LEU A 376 -14.479 19.824 124.508 1.00 0.00

ATOM 5932 H LEU A 376 -14.955 17.949 123.043 1.00 0.00

ATOM 5933 H LEU A 376 -15.904 19.321 121.239 1.00 0.00

ATOM 5934 H LEU A 376 -16.134 20.071 122.865 1.00 0.00

ATOM 5935 H LEU A 376 -14.854 20.695 121.756 1.00 0.00

ATOM 5936 H LEU A 376 -14.020 17.948 120.819 1.00 0.00

ATOM 5937 H LEU A 376 -12.884 19.260 121.316 1.00 0.00

ATOM 5938 H LEU A 376 -12.784 17.636 122.098 1.00 0.00

ATOM 5939 N LYS A 377 -12.693 20.278 126.544 1.00 91.38

ATOM 5940 CA LYS A 377 -12.013 21.155 127.488 1.00 95.18

ATOM 5941 C LYS A 377 -10.844 20.551 128.259 1.00 94.87

ATOM 5942 O LYS A 377 -9.863 21.238 128.513 1.00 96.39

ATOM 5943 CB LYS A 377 -12.994 21.876 128.418 1.00100.26

ATOM 5944 CG LYS A 377 -13.288 23.280 127.931 1.00104.90

ATOM 5945 CD LYS A 377 -11.981 24.075 127.670 1.00107.72

ATOM 5946 CE LYS A 377 -12.078 24.952 126.408 1.00110.67

ATOM 5947 NZ LYS A 377 -11.217 26.187 126.463 1.00117.39

ATOM 5948 H LYS A 377 -13.723 20.269 126.518 1.00 0.00

ATOM 5949 H LYS A 377 -11.538 21.891 126.840 1.00 0.00

ATOM 5950 H LYS A 377 -12.556 21.940 129.414 1.00 0.00

ATOM 5951 H LYS A 377 -13.931 21.320 128.430 1.00 0.00

ATOM 5952 H LYS A 377 -13.851 23.788 128.714 1.00 0.00

ATOM 5953 H LYS A 377 -13.863 23.212 127.008 1.00 0.00

ATOM 5954 H LYS A 377 -11.167 23.364 127.528 1.00 0.00

ATOM 5955 H LYS A 377 -11.808 24.733 128.521 1.00 0.00

ATOM 5956 H LYS A 377 -13.112 25.280 126.296 1.00 0.00

ATOM 5957 H LYS A 377 -11.715 24.358 125.569 1.00 0.00

ATOM 5958 H LYS A 377 -11.359 26.693 125.577 1.00 0.00

ATOM 5959 H LYS A 377 -11.533 26.738 127.274 1.00 0.00

ATOM 5960 H LYS A 377 -10.243 25.870 126.571 1.00 0.00

ATOM 5961 N HIS A 378 -10.936 19.273 128.599 1.00 94.20

ATOM 5962 CA HIS A 378 -9.772 18.533 129.099 1.00 95.25

ATOM 5963 C HIS A 378 -8.642 18.259 128.077 1.00 91.57

ATOM 5964 O HIS A 378 -7.490 18.108 128.447 1.00 91.83

ATOM 5965 CB HIS A 378 -10.238 17.249 129.789 1.00 98.39

ATOM 5966 CG HIS A 378 -11.321 17.489 130.799 1.00104.55

ATOM 5967 ND1 HIS A 378 -12.570 16.911 130.706 1.00106.89

ATOM 5968 CD2 HIS A 378 -11.354 18.281 131.901 1.00110.19

ATOM 5969 CE1 HIS A 378 -13.320 17.320 131.716 1.00112.38

ATOM 5970 NE2 HIS A 378 -12.606 18.154 132.453 1.00115.89

ATOM 5971 H HIS A 378 -11.842 18.790 128.510 1.00 0.00

ATOM 5972 H HIS A 378 -9.293 19.200 129.816 1.00 0.00

ATOM 5973 H HIS A 378 -9.389 16.806 130.310 1.00 0.00

ATOM 5974 H HIS A 378 -10.656 16.593 129.025 1.00 0.00

ATOM 5975 H HIS A 378 -12.872 16.262 129.965 1.00 0.00

ATOM 5976 H HIS A 378 -10.540 18.901 132.277 1.00 0.00

ATOM 5977 H HIS A 378 -14.350 17.021 131.908 1.00 0.00

ATOM 5978 N LEU A 379 -8.946 18.209 126.793 1.00 89.60

ATOM 5979 CA LEU A 379 -7.861 18.012 125.825 1.00 88.49

ATOM 5980 C LEU A 379 -6.740 19.050 125.879 1.00 88.41

ATOM 5981 O LEU A 379 -5.580 18.678 125.830 1.00 87.19

ATOM 5982 CB LEU A 379 -8.387 17.854 124.407 1.00 88.09

ATOM 5983 CG LEU A 379 -8.778 16.406 124.115 1.00 89.20

ATOM 5984 CD1 LEU A 379 -8.351 16.042 122.702 1.00 89.17

ATOM 5985 CD2 LEU A 379 -8.145 15.441 125.121 1.00 92.74

ATOM 5986 H LEU A 379 -9.922 18.306 126.478 1.00 0.00

ATOM 5987 H LEU A 379 -7.397 17.077 126.138 1.00 0.00

ATOM 5988 H LEU A 379 -7.597 18.146 123.714 1.00 0.00

ATOM 5989 H LEU A 379 -9.269 18.484 124.293 1.00 0.00

ATOM 5990 H LEU A 379 -9.860 16.317 124.208 1.00 0.00

ATOM 5991 H LEU A 379 -8.648 15.006 122.536 1.00 0.00

ATOM 5992 H LEU A 379 -8.868 16.724 122.027 1.00 0.00

ATOM 5993 H LEU A 379 -7.269 16.166 122.654 1.00 0.00

ATOM 5994 H LEU A 379 -8.469 14.437 124.847 1.00 0.00

ATOM 5995 H LEU A 379 -7.064 15.555 125.036 1.00 0.00

ATOM 5996 H LEU A 379 -8.506 15.728 126.109 1.00 0.00

ATOM 5997 N VAL A 380 -7.102 20.327 126.030 1.00 90.59

ATOM 5998 CA VAL A 380 -6.151 21.476 126.127 1.00 92.87

ATOM 5999 C VAL A 380 -4.971 21.374 127.117 1.00 93.84

ATOM 6000 O VAL A 380 -3.913 21.975 126.917 1.00 94.17

ATOM 6001 CB VAL A 380 -6.876 22.802 126.530 1.00 96.86

ATOM 6002 CG1 VAL A 380 -6.069 24.010 126.032 1.00 98.86

ATOM 6003 CG2 VAL A 380 -8.317 22.844 126.007 1.00 96.68

ATOM 6004 H VAL A 380 -8.109 20.535 126.083 1.00 0.00

ATOM 6005 H VAL A 380 -5.744 21.460 125.116 1.00 0.00

ATOM 6006 H VAL A 380 -6.934 22.842 127.618 1.00 0.00

ATOM 6007 H VAL A 380 -6.613 24.904 126.336 1.00 0.00

ATOM 6008 H VAL A 380 -5.087 23.953 126.502 1.00 0.00

ATOM 6009 H VAL A 380 -6.004 23.925 124.947 1.00 0.00

ATOM 6010 H VAL A 380 -8.746 23.793 126.330 1.00 0.00

ATOM 6011 H VAL A 380 -8.264 22.772 124.921 1.00 0.00

ATOM 6012 H VAL A 380 -8.840 21.994 126.445 1.00 0.00

ATOM 6013 N ASP A 381 -5.175 20.623 128.190 1.00 95.44

ATOM 6014 CA ASP A 381 -4.398 20.777 129.400 1.00 98.62

ATOM 6015 C ASP A 381 -3.482 19.590 129.555 1.00 97.44

ATOM 6016 O ASP A 381 -2.546 19.617 130.352 1.00 99.18

ATOM 6017 CB ASP A 381 -5.372 20.903 130.562 1.00103.61

ATOM 6018 CG ASP A 381 -6.671 21.614 130.147 1.00106.08

ATOM 6019 OD1 ASP A 381 -7.223 21.277 129.072 1.00103.72

ATOM 6020 OD2 ASP A 381 -7.143 22.517 130.876 1.00113.64

ATOM 6021 H ASP A 381 -5.913 19.905 128.161 1.00 0.00

ATOM 6022 H ASP A 381 -3.772 21.669 129.367 1.00 0.00

ATOM 6023 H ASP A 381 -4.890 21.497 131.338 1.00 0.00

ATOM 6024 H ASP A 381 -5.618 19.900 130.912 1.00 0.00

ATOM 6025 N GLU A 382 -3.744 18.559 128.752 1.00 95.64

ATOM 6026 CA GLU A 382 -2.899 17.371 128.716 1.00 96.08

ATOM 6027 C GLU A 382 -1.410 17.748 128.468 1.00 95.16

ATOM 6028 O GLU A 382 -0.553 17.365 129.270 1.00 97.44

ATOM 6029 CB GLU A 382 -3.444 16.301 127.735 1.00 95.23

ATOM 6030 CG GLU A 382 -3.342 14.860 128.318 1.00 99.52

ATOM 6031 CD GLU A 382 -3.760 13.708 127.379 1.00 99.58

ATOM 6032 OE1 GLU A 382 -4.741 13.839 126.610 1.00 94.54

ATOM 6033 OE2 GLU A 382 -3.115 12.632 127.463 1.00105.11

ATOM 6034 H GLU A 382 -4.569 18.603 128.137 1.00 0.00

ATOM 6035 H GLU A 382 -2.932 16.901 129.699 1.00 0.00

ATOM 6036 H GLU A 382 -2.843 16.342 126.827 1.00 0.00

ATOM 6037 H GLU A 382 -4.495 16.520 127.545 1.00 0.00

ATOM 6038 H GLU A 382 -4.024 14.821 129.167 1.00 0.00

ATOM 6039 H GLU A 382 -2.287 14.696 128.536 1.00 0.00

ATOM 6040 N PRO A 383 -1.104 18.498 127.373 1.00 93.05

ATOM 6041 CA PRO A 383 0.189 19.142 127.058 1.00 91.99

ATOM 6042 C PRO A 383 0.935 19.937 128.142 1.00 93.15

ATOM 6043 O PRO A 383 2.145 19.732 128.318 1.00 92.87

ATOM 6044 CB PRO A 383 -0.201 20.096 125.945 1.00 92.29

ATOM 6045 CG PRO A 383 -1.179 19.279 125.147 1.00 93.35

ATOM 6046 CD PRO A 383 -1.928 18.409 126.151 1.00 92.11

ATOM 6047 H PRO A 383 0.909 18.350 126.852 1.00 0.00

ATOM 6048 H PRO A 383 0.647 20.444 125.355 1.00 0.00

ATOM 6049 H PRO A 383 -0.630 21.028 126.312 1.00 0.00

ATOM 6050 H PRO A 383 -0.656 18.668 124.411 1.00 0.00

ATOM 6051 H PRO A 383 -1.858 19.920 124.584 1.00 0.00

ATOM 6052 H PRO A 383 -2.943 18.763 126.333 1.00 0.00

ATOM 6053 H PRO A 383 -2.070 17.380 125.819 1.00 0.00

ATOM 6054 N GLN A 384 0.223 20.826 128.846 1.00 94.86

ATOM 6055 CA GLN A 384 0.825 21.855 129.724 1.00 97.23

ATOM 6056 C GLN A 384 1.996 21.480 130.680 1.00 97.66

ATOM 6057 O GLN A 384 2.939 22.252 130.879 1.00 98.56

ATOM 6058 CB GLN A 384 -0.281 22.575 130.512 1.00102.18

ATOM 6059 CG GLN A 384 -0.064 24.082 130.661 1.00106.74

ATOM 6060 CD GLN A 384 -0.011 24.790 129.319 1.00106.01

ATOM 6061 OE1 GLN A 384 -0.880 24.583 128.465 1.00105.66

ATOM 6062 NE2 GLN A 384 1.017 25.613 129.117 1.00106.24

ATOM 6063 H GLN A 384 -0.804 20.792 128.772 1.00 0.00

ATOM 6064 H GLN A 384 1.334 22.490 128.999 1.00 0.00

ATOM 6065 H GLN A 384 -0.294 22.151 131.516 1.00 0.00

ATOM 6066 H GLN A 384 -1.210 22.440 129.959 1.00 0.00

ATOM 6067 H GLN A 384 0.887 24.245 131.168 1.00 0.00

ATOM 6068 H GLN A 384 -0.905 24.492 131.221 1.00 0.00

ATOM 6069 H GLN A 384 1.103 26.120 128.224 1.00 0.00

ATOM 6070 H GLN A 384 1.725 25.743 129.853 1.00 0.00

ATOM 6071 N ASN A 385 1.947 20.308 131.277 1.00 97.23

ATOM 6072 CA ASN A 385 3.011 19.932 132.199 1.00 99.45

ATOM 6073 C ASN A 385 4.226 19.374 131.498 1.00 95.28

ATOM 6074 O ASN A 385 5.312 19.345 132.076 1.00 96.61

ATOM 6075 CB ASN A 385 2.455 18.952 133.198 1.00104.82

ATOM 6076 CG ASN A 385 1.022 18.621 132.884 1.00105.88

ATOM 6077 OD1 ASN A 385 0.642 18.551 131.694 1.00101.36

ATOM 6078 ND2 ASN A 385 0.199 18.483 133.925 1.00110.28

ATOM 6079 H ASN A 385 1.165 19.663 131.094 1.00 0.00

ATOM 6080 H ASN A 385 3.358 20.830 132.710 1.00 0.00

ATOM 6081 H ASN A 385 2.502 19.398 134.192 1.00 0.00

ATOM 6082 H ASN A 385 3.043 18.035 133.155 1.00 0.00

ATOM 6083 H ASN A 385 -0.794 18.256 133.770 1.00 0.00

ATOM 6084 H ASN A 385 0.556 18.603 134.884 1.00 0.00

ATOM 6085 N LEU A 386 4.041 18.954 130.244 1.00 90.91

ATOM 6086 CA LEU A 386 5.155 18.596 129.388 1.00 87.32

ATOM 6087 C LEU A 386 6.009 19.835 129.350 1.00 86.00

ATOM 6088 O LEU A 386 7.176 19.799 129.723 1.00 86.94

ATOM 6089 CB LEU A 386 4.682 18.225 127.972 1.00 85.61

ATOM 6090 CG LEU A 386 5.751 17.916 126.890 1.00 84.24

ATOM 6091 CD1 LEU A 386 5.211 17.024 125.774 1.00 84.50

ATOM 6092 CD2 LEU A 386 6.354 19.156 126.303 1.00 79.71

ATOM 6093 H LEU A 386 3.082 18.883 129.875 1.00 0.00

ATOM 6094 H LEU A 386 5.690 17.723 129.762 1.00 0.00

ATOM 6095 H LEU A 386 4.161 19.112 127.612 1.00 0.00

ATOM 6096 H LEU A 386 4.115 17.302 128.094 1.00 0.00

ATOM 6097 H LEU A 386 6.538 17.372 127.413 1.00 0.00

ATOM 6098 H LEU A 386 6.028 16.865 125.070 1.00 0.00

ATOM 6099 H LEU A 386 4.889 16.092 126.239 1.00 0.00

ATOM 6100 H LEU A 386 4.378 17.557 125.315 1.00 0.00

ATOM 6101 H LEU A 386 7.085 18.834 125.561 1.00 0.00

ATOM 6102 H LEU A 386 5.540 19.723 125.851 1.00 0.00

ATOM 6103 H LEU A 386 6.821 19.702 127.123 1.00 0.00

ATOM 6104 N ILE A 387 5.389 20.927 128.914 1.00 85.12

ATOM 6105 CA ILE A 387 6.028 22.222 128.699 1.00 85.12

ATOM 6106 C ILE A 387 6.976 22.702 129.778 1.00 85.97

ATOM 6107 O ILE A 387 8.158 22.942 129.502 1.00 84.33

ATOM 6108 CB ILE A 387 4.962 23.366 128.560 1.00 88.43

ATOM 6109 CG1 ILE A 387 3.720 22.887 127.786 1.00 89.03

ATOM 6110 CG2 ILE A 387 5.584 24.665 127.975 1.00 90.55

ATOM 6111 CD1 ILE A 387 3.951 22.584 126.273 1.00 90.73

ATOM 6112 H ILE A 387 4.382 20.854 128.713 1.00 0.00

ATOM 6113 H ILE A 387 6.605 22.036 127.793 1.00 0.00

ATOM 6114 H ILE A 387 4.617 23.625 129.561 1.00 0.00

ATOM 6115 H ILE A 387 3.001 23.705 127.834 1.00 0.00

ATOM 6116 H ILE A 387 3.400 21.957 128.255 1.00 0.00

ATOM 6117 H ILE A 387 4.783 25.402 127.912 1.00 0.00

ATOM 6118 H ILE A 387 6.370 24.979 128.661 1.00 0.00

ATOM 6119 H ILE A 387 5.981 24.413 126.992 1.00 0.00

ATOM 6120 H ILE A 387 2.995 22.259 125.863 1.00 0.00

ATOM 6121 H ILE A 387 4.296 23.509 125.812 1.00 0.00

ATOM 6122 H ILE A 387 4.704 21.798 126.217 1.00 0.00

ATOM 6123 N LYS A 388 6.445 22.823 131.000 1.00 88.51

ATOM 6124 CA LYS A 388 7.185 23.368 132.147 1.00 92.14

ATOM 6125 C LYS A 388 8.536 22.653 132.281 1.00 89.89

ATOM 6126 O LYS A 388 9.606 23.291 132.350 1.00 90.74

ATOM 6127 CB LYS A 388 6.351 23.224 133.429 1.00 97.71

ATOM 6128 CG LYS A 388 7.142 23.287 134.739 1.00103.35

ATOM 6129 CD LYS A 388 6.205 23.236 135.952 1.00111.70

ATOM 6130 CE LYS A 388 5.921 24.627 136.518 1.00118.33

ATOM 6131 NZ LYS A 388 4.711 24.656 137.400 1.00125.72

ATOM 6132 H LYS A 388 5.471 22.520 131.144 1.00 0.00

ATOM 6133 H LYS A 388 7.374 24.429 131.986 1.00 0.00

ATOM 6134 H LYS A 388 5.888 22.238 133.392 1.00 0.00

ATOM 6135 H LYS A 388 5.659 24.066 133.446 1.00 0.00

ATOM 6136 H LYS A 388 7.697 24.225 134.766 1.00 0.00

ATOM 6137 H LYS A 388 7.816 22.431 134.781 1.00 0.00

ATOM 6138 H LYS A 388 6.685 22.643 136.731 1.00 0.00

ATOM 6139 H LYS A 388 5.258 22.802 135.632 1.00 0.00

ATOM 6140 H LYS A 388 5.747 25.313 135.689 1.00 0.00

ATOM 6141 H LYS A 388 6.772 24.918 137.134 1.00 0.00

ATOM 6142 H LYS A 388 4.602 25.626 137.730 1.00 0.00

ATOM 6143 H LYS A 388 3.911 24.355 136.824 1.00 0.00

ATOM 6144 H LYS A 388 4.889 24.004 138.178 1.00 0.00

ATOM 6145 N GLN A 389 8.464 21.324 132.278 1.00 87.31

ATOM 6146 CA GLN A 389 9.606 20.454 132.476 1.00 86.34

ATOM 6147 C GLN A 389 10.722 20.725 131.504 1.00 81.68

ATOM 6148 O GLN A 389 11.864 20.942 131.906 1.00 82.01

ATOM 6149 CB GLN A 389 9.146 19.003 132.356 1.00 87.67

ATOM 6150 CG GLN A 389 8.147 18.632 133.473 1.00 95.34

ATOM 6151 CD GLN A 389 7.569 17.249 133.323 1.00 99.92

ATOM 6152 OE1 GLN A 389 8.278 16.300 132.946 1.00 99.62

ATOM 6153 NE2 GLN A 389 6.268 17.110 133.636 1.00104.09

ATOM 6154 H GLN A 389 7.543 20.888 132.126 1.00 0.00

ATOM 6155 H GLN A 389 10.007 20.650 133.470 1.00 0.00

ATOM 6156 H GLN A 389 10.023 18.362 132.450 1.00 0.00

ATOM 6157 H GLN A 389 8.659 18.874 131.389 1.00 0.00

ATOM 6158 H GLN A 389 7.318 19.339 133.447 1.00 0.00

ATOM 6159 H GLN A 389 8.692 18.640 134.417 1.00 0.00

ATOM 6160 H GLN A 389 5.814 16.189 133.554 1.00 0.00

ATOM 6161 H GLN A 389 5.726 17.925 133.957 1.00 0.00

ATOM 6162 N ASN A 390 10.366 20.741 130.224 1.00 77.46

ATOM 6163 CA ASN A 390 11.310 20.924 129.166 1.00 74.17

ATOM 6164 C ASN A 390 11.940 22.327 129.148 1.00 75.06

ATOM 6165 O ASN A 390 13.154 22.462 128.935 1.00 74.39

ATOM 6166 CB ASN A 390 10.625 20.561 127.859 1.00 73.56

ATOM 6167 CG ASN A 390 10.515 19.046 127.671 1.00 71.42

ATOM 6168 OD1 ASN A 390 11.509 18.370 127.628 1.00 72.86

ATOM 6169 ND2 ASN A 390 9.324 18.536 127.548 1.00 68.07

ATOM 6170 H ASN A 390 9.372 20.617 129.985 1.00 0.00

ATOM 6171 H ASN A 390 12.161 20.262 129.326 1.00 0.00

ATOM 6172 H ASN A 390 11.225 20.963 127.043 1.00 0.00

ATOM 6173 H ASN A 390 9.620 20.982 127.873 1.00 0.00

ATOM 6174 H ASN A 390 9.209 17.520 127.420 1.00 0.00

ATOM 6175 H ASN A 390 8.496 19.148 127.579 1.00 0.00

ATOM 6176 N CYS A 391 11.148 23.361 129.426 1.00 76.56

ATOM 6177 CA CYS A 391 11.721 24.697 129.560 1.00 79.49

ATOM 6178 C CYS A 391 12.656 24.860 130.761 1.00 79.92

ATOM 6179 O CYS A 391 13.621 25.580 130.678 1.00 80.23

ATOM 6180 CB CYS A 391 10.657 25.780 129.495 1.00 84.59

ATOM 6181 SG CYS A 391 9.822 25.800 127.865 1.00 89.12

ATOM 6182 H CYS A 391 10.135 23.219 129.546 1.00 0.00

ATOM 6183 H CYS A 391 12.366 24.825 128.690 1.00 0.00

ATOM 6184 H CYS A 391 11.262 26.684 129.562 1.00 0.00

ATOM 6185 H CYS A 391 10.018 25.663 130.370 1.00 0.00

ATOM 6186 N ASP A 392 12.422 24.129 131.840 1.00 80.45

ATOM 6187 CA ASP A 392 13.352 24.135 132.987 1.00 83.48

ATOM 6188 C ASP A 392 14.694 23.456 132.659 1.00 79.22

ATOM 6189 O ASP A 392 15.767 24.039 132.809 1.00 79.40

ATOM 6190 CB ASP A 392 12.691 23.494 134.218 1.00 87.68

ATOM 6191 CG ASP A 392 11.426 24.249 134.677 1.00 96.32

ATOM 6192 OD1 ASP A 392 11.332 25.504 134.550 1.00101.07

ATOM 6193 OD2 ASP A 392 10.503 23.580 135.194 1.00103.37

ATOM 6194 H ASP A 392 11.575 23.545 131.880 1.00 0.00

ATOM 6195 H ASP A 392 13.579 25.176 133.215 1.00 0.00

ATOM 6196 H ASP A 392 13.412 23.523 135.035 1.00 0.00

ATOM 6197 H ASP A 392 12.399 22.479 133.950 1.00 0.00

ATOM 6198 N GLN A 393 14.608 22.217 132.207 1.00 75.51

ATOM 6199 CA GLN A 393 15.742 21.493 131.676 1.00 73.54

ATOM 6200 C GLN A 393 16.481 22.386 130.643 1.00 71.44

ATOM 6201 O GLN A 393 17.727 22.464 130.658 1.00 70.67

ATOM 6202 CB GLN A 393 15.233 20.204 131.023 1.00 72.35

ATOM 6203 CG GLN A 393 16.149 19.012 131.084 1.00 75.35

ATOM 6204 CD GLN A 393 15.642 17.801 130.251 1.00 78.52

ATOM 6205 OE1 GLN A 393 14.733 17.919 129.415 1.00 78.27

ATOM 6206 NE2 GLN A 393 16.227 16.634 130.503 1.00 80.58

ATOM 6207 H GLN A 393 13.692 21.746 132.234 1.00 0.00

ATOM 6208 H GLN A 393 16.444 21.236 132.470 1.00 0.00

ATOM 6209 H GLN A 393 15.091 20.421 129.964 1.00 0.00

ATOM 6210 H GLN A 393 14.345 19.913 131.584 1.00 0.00

ATOM 6211 H GLN A 393 16.177 18.703 132.129 1.00 0.00

ATOM 6212 H GLN A 393 17.120 19.324 130.700 1.00 0.00

ATOM 6213 H GLN A 393 15.938 15.791 129.986 1.00 0.00

ATOM 6214 H GLN A 393 16.969 16.573 131.215 1.00 0.00

ATOM 6215 N PHE A 394 15.716 23.075 129.784 1.00 70.59

ATOM 6216 CA PHE A 394 16.319 23.954 128.784 1.00 70.87

ATOM 6217 C PHE A 394 17.034 25.132 129.437 1.00 74.94

ATOM 6218 O PHE A 394 18.198 25.361 129.089 1.00 75.80

ATOM 6219 CB PHE A 394 15.331 24.465 127.677 1.00 71.08

ATOM 6220 CG PHE A 394 15.900 25.606 126.819 1.00 68.87

ATOM 6221 CD1 PHE A 394 16.758 25.352 125.732 1.00 66.53

ATOM 6222 CD2 PHE A 394 15.618 26.930 127.118 1.00 71.85

ATOM 6223 CE1 PHE A 394 17.279 26.412 124.934 1.00 67.87

ATOM 6224 CE2 PHE A 394 16.160 27.992 126.339 1.00 73.57

ATOM 6225 CZ PHE A 394 16.988 27.714 125.255 1.00 71.40

ATOM 6226 H PHE A 394 14.691 22.984 129.829 1.00 0.00

ATOM 6227 H PHE A 394 17.045 23.325 128.269 1.00 0.00

ATOM 6228 H PHE A 394 14.434 24.843 128.168 1.00 0.00

ATOM 6229 H PHE A 394 15.143 23.632 126.999 1.00 0.00

ATOM 6230 H PHE A 394 17.030 24.323 125.496 1.00 0.00

ATOM 6231 H PHE A 394 14.971 27.162 127.964 1.00 0.00

ATOM 6232 H PHE A 394 17.907 26.189 124.071 1.00 0.00

ATOM 6233 H PHE A 394 15.926 29.026 126.592 1.00 0.00

ATOM 6234 H PHE A 394 17.404 28.529 124.663 1.00 0.00

ATOM 6235 N GLU A 395 16.351 25.902 130.310 1.00 78.74

ATOM 6236 CA GLU A 395 17.005 27.056 130.941 1.00 84.38

ATOM 6237 C GLU A 395 18.209 26.546 131.688 1.00 82.89

ATOM 6238 O GLU A 395 19.269 27.168 131.680 1.00 84.74

ATOM 6239 CB GLU A 395 16.146 27.831 131.954 1.00 90.59

ATOM 6240 CG GLU A 395 14.634 27.883 131.763 1.00 95.50

ATOM 6241 CD GLU A 395 14.126 28.774 130.617 1.00100.21

ATOM 6242 OE1 GLU A 395 14.964 29.318 129.856 1.00101.01

ATOM 6243 OE2 GLU A 395 12.870 28.897 130.476 1.00102.18

ATOM 6244 H GLU A 395 15.370 25.680 130.534 1.00 0.00

ATOM 6245 H GLU A 395 17.233 27.749 130.131 1.00 0.00

ATOM 6246 H GLU A 395 16.473 28.868 131.872 1.00 0.00

ATOM 6247 H GLU A 395 16.283 27.303 132.897 1.00 0.00

ATOM 6248 H GLU A 395 14.232 28.309 132.682 1.00 0.00

ATOM 6249 H GLU A 395 14.321 26.865 131.529 1.00 0.00

ATOM 6250 N LYS A 396 18.029 25.406 132.337 1.00 81.03

ATOM 6251 CA LYS A 396 19.071 24.796 133.120 1.00 81.02

ATOM 6252 C LYS A 396 20.309 24.544 132.245 1.00 78.02

ATOM 6253 O LYS A 396 21.403 25.018 132.589 1.00 79.57

ATOM 6254 CB LYS A 396 18.541 23.514 133.768 1.00 80.77

ATOM 6255 CG LYS A 396 19.218 23.099 135.061 1.00 85.19

ATOM 6256 CD LYS A 396 18.773 21.698 135.477 1.00 88.26

ATOM 6257 CE LYS A 396 19.779 21.051 136.429 1.00 95.70

ATOM 6258 NZ LYS A 396 21.130 20.846 135.798 1.00 93.21

ATOM 6259 H LYS A 396 17.112 24.940 132.280 1.00 0.00

ATOM 6260 H LYS A 396 19.379 25.467 133.922 1.00 0.00

ATOM 6261 H LYS A 396 18.715 22.707 133.057 1.00 0.00

ATOM 6262 H LYS A 396 17.499 23.706 134.022 1.00 0.00

ATOM 6263 H LYS A 396 18.937 23.805 135.843 1.00 0.00

ATOM 6264 H LYS A 396 20.298 23.101 134.910 1.00 0.00

ATOM 6265 H LYS A 396 18.697 21.080 134.582 1.00 0.00

ATOM 6266 H LYS A 396 17.814 21.778 135.989 1.00 0.00

ATOM 6267 H LYS A 396 19.397 20.074 136.724 1.00 0.00

ATOM 6268 H LYS A 396 19.917 21.723 137.276 1.00 0.00

ATOM 6269 H LYS A 396 21.732 20.409 136.511 1.00 0.00

ATOM 6270 H LYS A 396 20.992 20.230 134.984 1.00 0.00

ATOM 6271 H LYS A 396 21.477 21.775 135.521 1.00 0.00

ATOM 6272 N LEU A 397 20.148 23.851 131.105 1.00 74.29

ATOM 6273 CA LEU A 397 21.312 23.342 130.387 1.00 71.25

ATOM 6274 C LEU A 397 21.806 24.206 129.242 1.00 71.92

ATOM 6275 O LEU A 397 22.957 24.082 128.849 1.00 71.44

ATOM 6276 CB LEU A 397 21.042 21.928 129.886 1.00 69.79

ATOM 6277 CG LEU A 397 20.888 20.787 130.872 1.00 70.58

ATOM 6278 CD1 LEU A 397 19.975 19.694 130.306 1.00 68.14

ATOM 6279 CD2 LEU A 397 22.284 20.224 131.292 1.00 71.10

ATOM 6280 H LEU A 397 19.201 23.679 130.737 1.00 0.00

ATOM 6281 H LEU A 397 22.118 23.354 131.121 1.00 0.00

ATOM 6282 H LEU A 397 21.927 21.667 129.306 1.00 0.00

ATOM 6283 H LEU A 397 20.068 21.994 129.400 1.00 0.00

ATOM 6284 H LEU A 397 20.409 21.172 131.773 1.00 0.00

ATOM 6285 H LEU A 397 19.909 18.911 131.061 1.00 0.00

ATOM 6286 H LEU A 397 19.006 20.155 130.115 1.00 0.00

ATOM 6287 H LEU A 397 20.440 19.334 129.388 1.00 0.00

ATOM 6288 H LEU A 397 22.102 19.413 131.997 1.00 0.00

ATOM 6289 H LEU A 397 22.771 19.870 130.384 1.00 0.00

ATOM 6290 H LEU A 397 22.833 21.045 131.753 1.00 0.00

ATOM 6291 N GLY A 398 20.953 25.083 128.712 1.00 74.62

ATOM 6292 CA GLY A 398 21.265 25.844 127.491 1.00 77.83

ATOM 6293 C GLY A 398 21.053 24.953 126.263 1.00 78.14

ATOM 6294 O GLY A 398 21.019 23.707 126.387 1.00 75.84

ATOM 6295 H GLY A 398 20.044 25.233 129.173 1.00 0.00

ATOM 6296 H GLY A 398 22.311 26.145 127.543 1.00 0.00

ATOM 6297 H GLY A 398 20.632 26.730 127.446 1.00 0.00

ATOM 6298 N GLU A 399 20.933 25.581 125.096 1.00 69.41

ATOM 6299 CA GLU A 399 20.584 24.936 123.850 1.00 68.11

ATOM 6300 C GLU A 399 21.395 23.636 123.474 1.00 66.90

ATOM 6301 O GLU A 399 20.782 22.600 123.169 1.00 63.73

ATOM 6302 CB GLU A 399 20.593 25.971 122.716 1.00 69.81

ATOM 6303 CG GLU A 399 20.296 25.405 121.259 1.00 70.72

ATOM 6304 CD GLU A 399 19.915 26.540 120.281 1.00 76.27

ATOM 6305 OE1 GLU A 399 19.404 27.566 120.750 1.00 81.91

ATOM 6306 OE2 GLU A 399 20.109 26.445 119.062 1.00 76.96

ATOM 6307 H GLU A 399 21.100 26.597 125.079 1.00 0.00

ATOM 6308 H GLU A 399 19.578 24.545 124.002 1.00 0.00

ATOM 6309 H GLU A 399 21.613 26.354 122.694 1.00 0.00

ATOM 6310 H GLU A 399 19.818 26.698 122.958 1.00 0.00

ATOM 6311 H GLU A 399 19.471 24.694 121.302 1.00 0.00

ATOM 6312 H GLU A 399 21.204 24.938 120.877 1.00 0.00

ATOM 6313 N TYR A 400 22.732 23.707 123.495 1.00 68.26

ATOM 6314 CA TYR A 400 23.585 22.564 123.187 1.00 68.25

ATOM 6315 C TYR A 400 23.347 21.440 124.184 1.00 68.00

ATOM 6316 O TYR A 400 23.116 20.288 123.769 1.00 67.04

ATOM 6317 CB TYR A 400 25.061 22.963 123.172 1.00 70.48

ATOM 6318 CG TYR A 400 26.055 21.911 122.717 1.00 72.34

ATOM 6319 CD1 TYR A 400 25.823 21.108 121.587 1.00 68.41

ATOM 6320 CD2 TYR A 400 27.287 21.778 123.368 1.00 75.49

ATOM 6321 CE1 TYR A 400 26.763 20.187 121.164 1.00 66.22

ATOM 6322 CE2 TYR A 400 28.225 20.872 122.964 1.00 73.00

ATOM 6323 CZ TYR A 400 27.977 20.066 121.856 1.00 73.54

ATOM 6324 OH TYR A 400 28.977 19.160 121.432 1.00 69.47

ATOM 6325 H TYR A 400 23.176 24.604 123.738 1.00 0.00

ATOM 6326 H TYR A 400 23.324 22.209 122.190 1.00 0.00

ATOM 6327 H TYR A 400 25.324 23.193 124.204 1.00 0.00

ATOM 6328 H TYR A 400 25.143 23.773 122.448 1.00 0.00

ATOM 6329 H TYR A 400 24.888 21.213 121.036 1.00 0.00

ATOM 6330 H TYR A 400 27.504 22.417 124.224 1.00 0.00

ATOM 6331 H TYR A 400 26.562 19.558 120.297 1.00 0.00

ATOM 6332 H TYR A 400 29.165 20.780 123.507 1.00 0.00

ATOM 6333 H TYR A 400 28.615 18.683 120.594 1.00 0.00

ATOM 6334 N GLY A 401 23.360 21.775 125.477 1.00 69.81

ATOM 6335 CA GLY A 401 23.234 20.801 126.529 1.00 69.99

ATOM 6336 C GLY A 401 21.861 20.176 126.481 1.00 69.05

ATOM 6337 O GLY A 401 21.650 19.085 127.026 1.00 69.81

ATOM 6338 H GLY A 401 23.464 22.768 125.731 1.00 0.00

ATOM 6339 H GLY A 401 23.358 21.325 127.477 1.00 0.00

ATOM 6340 H GLY A 401 24.016 20.049 126.420 1.00 0.00

ATOM 6341 N PHE A 402 20.904 20.880 125.874 1.00 67.10

ATOM 6342 CA PHE A 402 19.541 20.407 125.846 1.00 64.24

ATOM 6343 C PHE A 402 19.406 19.469 124.658 1.00 61.98

ATOM 6344 O PHE A 402 18.675 18.500 124.718 1.00 60.15

ATOM 6345 CB PHE A 402 18.557 21.577 125.732 1.00 64.46

ATOM 6346 CG PHE A 402 17.112 21.188 125.841 1.00 60.10

ATOM 6347 CD1 PHE A 402 16.642 20.470 126.921 1.00 57.75

ATOM 6348 CD2 PHE A 402 16.219 21.553 124.864 1.00 61.89

ATOM 6349 CE1 PHE A 402 15.302 20.105 127.040 1.00 58.98

ATOM 6350 CE2 PHE A 402 14.826 21.220 124.970 1.00 62.79

ATOM 6351 CZ PHE A 402 14.373 20.479 126.072 1.00 61.28

ATOM 6352 H PHE A 402 21.141 21.773 125.419 1.00 0.00

ATOM 6353 H PHE A 402 19.304 19.883 126.772 1.00 0.00

ATOM 6354 H PHE A 402 18.691 22.034 124.751 1.00 0.00

ATOM 6355 H PHE A 402 18.755 22.243 126.572 1.00 0.00

ATOM 6356 H PHE A 402 17.340 20.179 127.706 1.00 0.00

ATOM 6357 H PHE A 402 16.573 22.105 123.993 1.00 0.00

ATOM 6358 H PHE A 402 14.977 19.520 127.901 1.00 0.00

ATOM 6359 H PHE A 402 14.125 21.541 124.199 1.00 0.00

ATOM 6360 H PHE A 402 13.323 20.204 126.168 1.00 0.00

ATOM 6361 N GLN A 403 20.127 19.766 123.576 1.00 62.14

ATOM 6362 CA GLN A 403 20.144 18.890 122.398 1.00 59.80

ATOM 6363 C GLN A 403 20.700 17.516 122.750 1.00 60.15

ATOM 6364 O GLN A 403 20.086 16.545 122.368 1.00 60.66

ATOM 6365 CB GLN A 403 20.916 19.474 121.221 1.00 59.23

ATOM 6366 CG GLN A 403 20.352 20.716 120.701 1.00 57.01

ATOM 6367 CD GLN A 403 21.235 21.387 119.680 1.00 59.23

ATOM 6368 OE1 GLN A 403 22.461 21.246 119.675 1.00 63.65

ATOM 6369 NE2 GLN A 403 20.618 22.135 118.799 1.00 62.12

ATOM 6370 H GLN A 403 20.686 20.631 123.567 1.00 0.00

ATOM 6371 H GLN A 403 19.105 18.796 122.083 1.00 0.00

ATOM 6372 H GLN A 403 20.919 18.754 120.403 1.00 0.00

ATOM 6373 H GLN A 403 21.910 19.735 121.584 1.00 0.00

ATOM 6374 H GLN A 403 20.260 21.391 121.552 1.00 0.00

ATOM 6375 H GLN A 403 19.394 20.475 120.239 1.00 0.00

ATOM 6376 H GLN A 403 21.158 22.625 118.071 1.00 0.00

ATOM 6377 H GLN A 403 19.593 22.234 118.834 1.00 0.00

ATOM 6378 N ASN A 404 21.829 17.441 123.459 1.00 61.61

ATOM 6379 CA ASN A 404 22.359 16.204 124.003 1.00 61.82

ATOM 6380 C ASN A 404 21.373 15.622 125.041 1.00 64.05

ATOM 6381 O ASN A 404 21.314 14.434 125.295 1.00 65.10

ATOM 6382 CB ASN A 404 23.735 16.473 124.662 1.00 63.92

ATOM 6383 CG ASN A 404 24.717 17.222 123.735 1.00 62.53

ATOM 6384 OD1 ASN A 404 24.565 17.234 122.538 1.00 62.79

ATOM 6385 ND2 ASN A 404 25.708 17.841 124.301 1.00 67.13

ATOM 6386 H ASN A 404 22.354 18.311 123.630 1.00 0.00

ATOM 6387 H ASN A 404 22.487 15.480 123.198 1.00 0.00

ATOM 6388 H ASN A 404 24.181 15.509 124.906 1.00 0.00

ATOM 6389 H ASN A 404 23.565 17.101 125.536 1.00 0.00

ATOM 6390 H ASN A 404 26.390 18.355 123.726 1.00 0.00

ATOM 6391 H ASN A 404 25.812 17.818 125.325 1.00 0.00

ATOM 6392 N ALA A 405 20.572 16.447 125.666 1.00 65.66

ATOM 6393 CA ALA A 405 19.624 15.867 126.552 1.00 67.98

ATOM 6394 C ALA A 405 18.592 15.098 125.716 1.00 66.56

ATOM 6395 O ALA A 405 18.159 14.034 126.113 1.00 67.20

ATOM 6396 CB ALA A 405 18.957 16.925 127.371 1.00 69.39

ATOM 6397 H ALA A 405 20.626 17.466 125.523 1.00 0.00

ATOM 6398 H ALA A 405 20.125 15.185 127.240 1.00 0.00

ATOM 6399 H ALA A 405 18.246 16.421 128.026 1.00 0.00

ATOM 6400 H ALA A 405 19.740 17.431 127.936 1.00 0.00

ATOM 6401 H ALA A 405 18.458 17.600 126.675 1.00 0.00

ATOM 6402 N LEU A 406 18.220 15.649 124.559 1.00 65.54

ATOM 6403 CA LEU A 406 17.112 15.101 123.756 1.00 64.78

ATOM 6404 C LEU A 406 17.629 13.891 122.982 1.00 63.67

ATOM 6405 O LEU A 406 16.974 12.860 122.909 1.00 64.38

ATOM 6406 CB LEU A 406 16.480 16.174 122.837 1.00 63.71

ATOM 6407 CG LEU A 406 15.499 17.088 123.584 1.00 64.88

ATOM 6408 CD1 LEU A 406 15.394 18.385 122.903 1.00 65.34

ATOM 6409 CD2 LEU A 406 14.103 16.444 123.748 1.00 61.56

ATOM 6410 H LEU A 406 18.722 16.481 124.217 1.00 0.00

ATOM 6411 H LEU A 406 16.305 14.779 124.414 1.00 0.00

ATOM 6412 H LEU A 406 15.928 15.665 122.047 1.00 0.00

ATOM 6413 H LEU A 406 17.284 16.800 122.451 1.00 0.00

ATOM 6414 H LEU A 406 15.897 17.241 124.587 1.00 0.00

ATOM 6415 H LEU A 406 14.689 18.989 123.474 1.00 0.00

ATOM 6416 H LEU A 406 16.392 18.824 122.898 1.00 0.00

ATOM 6417 H LEU A 406 15.033 18.187 121.894 1.00 0.00

ATOM 6418 H LEU A 406 13.482 17.161 124.285 1.00 0.00

ATOM 6419 H LEU A 406 13.722 16.249 122.745 1.00 0.00

ATOM 6420 H LEU A 406 14.241 15.523 124.315 1.00 0.00

ATOM 6421 N ILE A 407 18.838 14.021 122.478 1.00 62.36

ATOM 6422 CA ILE A 407 19.559 12.936 121.881 1.00 62.15

ATOM 6423 C ILE A 407 19.579 11.666 122.728 1.00 63.09

ATOM 6424 O ILE A 407 19.515 10.565 122.180 1.00 63.85

ATOM 6425 CB ILE A 407 21.012 13.309 121.673 1.00 62.46

ATOM 6426 CG1 ILE A 407 21.139 14.262 120.519 1.00 62.17

ATOM 6427 CG2 ILE A 407 21.799 12.085 121.413 1.00 61.71

ATOM 6428 CD1 ILE A 407 22.456 14.119 119.800 1.00 64.73

ATOM 6429 H ILE A 407 19.287 14.947 122.514 1.00 0.00

ATOM 6430 H ILE A 407 19.033 12.744 120.946 1.00 0.00

ATOM 6431 H ILE A 407 21.393 13.798 122.570 1.00 0.00

ATOM 6432 H ILE A 407 21.078 15.276 120.914 1.00 0.00

ATOM 6433 H ILE A 407 20.339 14.044 119.811 1.00 0.00

ATOM 6434 H ILE A 407 22.834 12.396 121.270 1.00 0.00

ATOM 6435 H ILE A 407 21.686 11.442 122.286 1.00 0.00

ATOM 6436 H ILE A 407 21.386 11.625 120.515 1.00 0.00

ATOM 6437 H ILE A 407 22.455 14.844 118.986 1.00 0.00

ATOM 6438 H ILE A 407 23.241 14.327 120.528 1.00 0.00

ATOM 6439 H ILE A 407 22.507 13.095 119.430 1.00 0.00

ATOM 6440 N VAL A 408 19.745 11.808 124.030 1.00 63.64

ATOM 6441 CA VAL A 408 19.708 10.670 124.926 1.00 64.81

ATOM 6442 C VAL A 408 18.235 10.214 125.101 1.00 65.07

ATOM 6443 O VAL A 408 17.925 9.023 125.003 1.00 64.77

ATOM 6444 CB VAL A 408 20.299 11.083 126.293 1.00 67.75

ATOM 6445 CG1 VAL A 408 19.824 10.184 127.377 1.00 69.72

ATOM 6446 CG2 VAL A 408 21.849 11.148 126.255 1.00 69.25

ATOM 6447 H VAL A 408 19.904 12.749 124.418 1.00 0.00

ATOM 6448 H VAL A 408 20.294 9.848 124.516 1.00 0.00

ATOM 6449 H VAL A 408 19.939 12.089 126.511 1.00 0.00

ATOM 6450 H VAL A 408 20.279 10.534 128.304 1.00 0.00

ATOM 6451 H VAL A 408 18.737 10.263 127.404 1.00 0.00

ATOM 6452 H VAL A 408 20.151 9.176 127.121 1.00 0.00

ATOM 6453 H VAL A 408 22.180 11.444 127.250 1.00 0.00

ATOM 6454 H VAL A 408 22.203 10.152 125.990 1.00 0.00

ATOM 6455 H VAL A 408 22.119 11.888 125.502 1.00 0.00

ATOM 6456 N ARG A 409 17.325 11.167 125.362 1.00 64.93

ATOM 6457 CA ARG A 409 15.901 10.836 125.625 1.00 66.48

ATOM 6458 C ARG A 409 15.326 10.121 124.392 1.00 64.43

ATOM 6459 O ARG A 409 14.781 9.042 124.481 1.00 66.80

ATOM 6460 CB ARG A 409 15.113 12.088 126.094 1.00 66.90

ATOM 6461 CG ARG A 409 13.656 12.193 125.732 1.00 66.34

ATOM 6462 CD ARG A 409 12.806 12.730 126.877 1.00 69.19

ATOM 6463 NE ARG A 409 12.315 14.119 126.773 1.00 70.49

ATOM 6464 CZ ARG A 409 12.977 15.228 127.146 1.00 72.25

ATOM 6465 NH1 ARG A 409 14.250 15.205 127.583 1.00 71.48

ATOM 6466 NH2 ARG A 409 12.381 16.393 127.032 1.00 70.66

ATOM 6467 H ARG A 409 17.622 12.153 125.380 1.00 0.00

ATOM 6468 H ARG A 409 15.806 10.141 126.459 1.00 0.00

ATOM 6469 H ARG A 409 15.587 12.949 125.622 1.00 0.00

ATOM 6470 H ARG A 409 15.109 12.031 127.183 1.00 0.00

ATOM 6471 H ARG A 409 13.305 11.188 125.496 1.00 0.00

ATOM 6472 H ARG A 409 13.568 12.876 124.887 1.00 0.00

ATOM 6473 H ARG A 409 13.428 12.707 127.772 1.00 0.00

ATOM 6474 H ARG A 409 11.899 12.125 126.876 1.00 0.00

ATOM 6475 H ARG A 409 11.374 14.251 126.376 1.00 0.00

ATOM 6476 H ARG A 409 14.716 16.081 127.858 1.00 0.00

ATOM 6477 H ARG A 409 14.758 14.311 127.642 1.00 0.00

ATOM 6478 H ARG A 409 12.873 17.253 127.313 1.00 0.00

ATOM 6479 H ARG A 409 11.421 16.447 126.662 1.00 0.00

ATOM 6480 N TYR A 410 15.593 10.663 123.234 1.00 61.01

ATOM 6481 CA TYR A 410 15.132 10.084 121.997 1.00 60.10

ATOM 6482 C TYR A 410 15.876 8.835 121.491 1.00 58.67

ATOM 6483 O TYR A 410 15.330 8.054 120.769 1.00 59.37

ATOM 6484 CB TYR A 410 15.010 11.203 120.912 1.00 58.94

ATOM 6485 CG TYR A 410 13.596 11.747 120.874 1.00 60.12

ATOM 6486 CD1 TYR A 410 13.142 12.747 121.769 1.00 59.81

ATOM 6487 CD2 TYR A 410 12.683 11.198 119.979 1.00 60.52

ATOM 6488 CE1 TYR A 410 11.776 13.191 121.719 1.00 63.53

ATOM 6489 CE2 TYR A 410 11.376 11.630 119.907 1.00 65.11

ATOM 6490 CZ TYR A 410 10.908 12.620 120.751 1.00 66.90

ATOM 6491 OH TYR A 410 9.553 12.962 120.573 1.00 72.16

ATOM 6492 H TYR A 410 16.149 11.529 123.204 1.00 0.00

ATOM 6493 H TYR A 410 14.149 9.669 122.220 1.00 0.00

ATOM 6494 H TYR A 410 15.265 10.807 119.929 1.00 0.00

ATOM 6495 H TYR A 410 15.676 12.025 121.173 1.00 0.00

ATOM 6496 H TYR A 410 13.829 13.180 122.496 1.00 0.00

ATOM 6497 H TYR A 410 13.012 10.400 119.313 1.00 0.00

ATOM 6498 H TYR A 410 11.413 13.951 122.411 1.00 0.00

ATOM 6499 H TYR A 410 10.702 11.185 119.175 1.00 0.00

ATOM 6500 H TYR A 410 9.332 13.688 121.269 1.00 0.00

ATOM 6501 N THR A 411 17.105 8.615 121.868 1.00 58.11

ATOM 6502 CA THR A 411 17.735 7.364 121.450 1.00 58.06

ATOM 6503 C THR A 411 17.302 6.113 122.231 1.00 61.59

ATOM 6504 O THR A 411 17.287 5.004 121.669 1.00 62.00

ATOM 6505 CB THR A 411 19.228 7.475 121.459 1.00 57.21

ATOM 6506 OG1 THR A 411 19.559 8.472 120.519 1.00 52.84

ATOM 6507 CG2 THR A 411 19.877 6.159 121.043 1.00 55.94

ATOM 6508 H THR A 411 17.616 9.301 122.442 1.00 0.00

ATOM 6509 H THR A 411 17.373 7.216 120.432 1.00 0.00

ATOM 6510 H THR A 411 19.585 7.718 122.460 1.00 0.00

ATOM 6511 H THR A 411 19.106 9.343 120.831 1.00 0.00

ATOM 6512 H THR A 411 20.956 6.312 121.072 1.00 0.00

ATOM 6513 H THR A 411 19.556 5.404 121.761 1.00 0.00

ATOM 6514 H THR A 411 19.527 5.935 120.035 1.00 0.00

ATOM 6515 N ARG A 412 16.999 6.287 123.516 1.00 64.06

ATOM 6516 CA ARG A 412 16.475 5.204 124.304 1.00 68.29

ATOM 6517 C ARG A 412 15.101 4.898 123.817 1.00 67.99

ATOM 6518 O ARG A 412 14.623 3.790 123.986 1.00 70.96

ATOM 6519 CB ARG A 412 16.339 5.596 125.768 1.00 71.71

ATOM 6520 CG ARG A 412 17.621 5.953 126.407 1.00 74.37

ATOM 6521 CD ARG A 412 17.375 6.202 127.838 1.00 76.69

ATOM 6522 NE ARG A 412 18.642 6.005 128.560 1.00 86.05

ATOM 6523 CZ ARG A 412 19.285 7.000 129.136 1.00 89.09

ATOM 6524 NH1 ARG A 412 18.723 8.207 129.077 1.00 89.69

ATOM 6525 NH2 ARG A 412 20.435 6.801 129.783 1.00 93.57

ATOM 6526 H ARG A 412 17.142 7.209 123.951 1.00 0.00

ATOM 6527 H ARG A 412 17.156 4.358 124.212 1.00 0.00

ATOM 6528 H ARG A 412 15.924 4.752 126.319 1.00 0.00

ATOM 6529 H ARG A 412 15.720 6.492 125.809 1.00 0.00

ATOM 6530 H ARG A 412 18.036 6.846 125.939 1.00 0.00

ATOM 6531 H ARG A 412 18.335 5.138 126.290 1.00 0.00

ATOM 6532 H ARG A 412 16.617 5.516 128.215 1.00 0.00

ATOM 6533 H ARG A 412 17.013 7.220 127.986 1.00 0.00

ATOM 6534 H ARG A 412 19.040 5.057 128.617 1.00 0.00

ATOM 6535 H ARG A 412 19.190 9.015 129.514 1.00 0.00

ATOM 6536 H ARG A 412 17.822 8.332 128.595 1.00 0.00

ATOM 6537 H ARG A 412 20.921 7.594 130.226 1.00 0.00

ATOM 6538 H ARG A 412 20.837 5.854 129.839 1.00 0.00

ATOM 6539 N LYS A 413 14.460 5.923 123.266 1.00 65.98

ATOM 6540 CA LYS A 413 13.059 5.888 122.869 1.00 65.69

ATOM 6541 C LYS A 413 12.856 5.025 121.595 1.00 65.16

ATOM 6542 O LYS A 413 11.974 4.120 121.568 1.00 67.72

ATOM 6543 CB LYS A 413 12.593 7.319 122.623 1.00 64.07

ATOM 6544 CG LYS A 413 11.580 7.876 123.609 1.00 63.85

ATOM 6545 CD LYS A 413 11.006 9.229 123.085 1.00 61.71

ATOM 6546 CE LYS A 413 10.296 10.091 124.147 1.00 65.84

ATOM 6547 NZ LYS A 413 8.834 9.680 124.486 1.00 62.04

ATOM 6548 H LYS A 413 14.985 6.795 123.110 1.00 0.00

ATOM 6549 H LYS A 413 12.470 5.432 123.665 1.00 0.00

ATOM 6550 H LYS A 413 12.101 7.318 121.650 1.00 0.00

ATOM 6551 H LYS A 413 13.479 7.947 122.715 1.00 0.00

ATOM 6552 H LYS A 413 12.091 8.048 124.556 1.00 0.00

ATOM 6553 H LYS A 413 10.776 7.152 123.741 1.00 0.00

ATOM 6554 H LYS A 413 10.263 8.993 122.324 1.00 0.00

ATOM 6555 H LYS A 413 11.855 9.816 122.734 1.00 0.00

ATOM 6556 H LYS A 413 10.244 11.097 123.731 1.00 0.00

ATOM 6557 H LYS A 413 10.874 9.986 125.065 1.00 0.00

ATOM 6558 H LYS A 413 8.494 10.338 125.202 1.00 0.00

ATOM 6559 H LYS A 413 8.291 9.750 123.613 1.00 0.00

ATOM 6560 H LYS A 413 8.867 8.713 124.840 1.00 0.00

ATOM 6561 N VAL A 414 13.685 5.307 120.593 1.00 60.93

ATOM 6562 CA VAL A 414 13.637 4.715 119.251 1.00 60.32

ATOM 6563 C VAL A 414 15.029 4.404 118.682 1.00 58.90

ATOM 6564 O VAL A 414 15.433 4.932 117.678 1.00 56.53

ATOM 6565 CB VAL A 414 12.825 5.584 118.241 1.00 60.15

ATOM 6566 CG1 VAL A 414 11.326 5.511 118.559 1.00 60.29

ATOM 6567 CG2 VAL A 414 13.253 7.100 118.279 1.00 59.68

ATOM 6568 H VAL A 414 14.424 6.001 120.775 1.00 0.00

ATOM 6569 H VAL A 414 13.114 3.768 119.380 1.00 0.00

ATOM 6570 H VAL A 414 13.034 5.181 117.250 1.00 0.00

ATOM 6571 H VAL A 414 10.814 6.133 117.825 1.00 0.00

ATOM 6572 H VAL A 414 11.033 4.464 118.477 1.00 0.00

ATOM 6573 H VAL A 414 11.196 5.890 119.573 1.00 0.00

ATOM 6574 H VAL A 414 12.638 7.623 117.547 1.00 0.00

ATOM 6575 H VAL A 414 13.070 7.457 119.292 1.00 0.00

ATOM 6576 H VAL A 414 14.311 7.139 118.020 1.00 0.00

ATOM 6577 N PRO A 415 15.735 3.454 119.320 1.00 60.19

ATOM 6578 CA PRO A 415 17.096 3.119 119.076 1.00 58.54

ATOM 6579 C PRO A 415 17.336 2.663 117.641 1.00 58.70

ATOM 6580 O PRO A 415 18.458 2.795 117.136 1.00 57.03

ATOM 6581 CB PRO A 415 17.341 1.984 120.023 1.00 61.49

ATOM 6582 CG PRO A 415 16.239 2.054 121.045 1.00 64.43

ATOM 6583 CD PRO A 415 15.107 2.639 120.379 1.00 62.87

ATOM 6584 H PRO A 415 17.757 3.973 119.219 1.00 0.00

ATOM 6585 H PRO A 415 18.321 2.069 120.492 1.00 0.00

ATOM 6586 H PRO A 415 17.341 1.027 119.502 1.00 0.00

ATOM 6587 H PRO A 415 16.515 2.632 121.927 1.00 0.00

ATOM 6588 H PRO A 415 15.973 1.059 121.402 1.00 0.00

ATOM 6589 H PRO A 415 14.404 1.895 120.005 1.00 0.00

ATOM 6590 H PRO A 415 14.495 3.229 121.061 1.00 0.00

ATOM 6591 N GLN A 416 16.319 2.141 116.967 1.00 60.21

ATOM 6592 CA GLN A 416 16.551 1.624 115.603 1.00 60.60

ATOM 6593 C GLN A 416 16.752 2.692 114.543 1.00 59.21

ATOM 6594 O GLN A 416 17.465 2.445 113.550 1.00 58.13

ATOM 6595 CB GLN A 416 15.433 0.725 115.123 1.00 63.24

ATOM 6596 CG GLN A 416 15.194 -0.464 116.022 1.00 67.88

ATOM 6597 CD GLN A 416 14.050 -0.241 117.031 1.00 72.33

ATOM 6598 OE1 GLN A 416 13.368 0.868 117.141 1.00 68.16

ATOM 6599 NE2 GLN A 416 13.861 -1.293 117.833 1.00 73.46

ATOM 6600 H GLN A 416 15.381 2.097 117.389 1.00 0.00

ATOM 6601 H GLN A 416 17.480 1.065 115.715 1.00 0.00

ATOM 6602 H GLN A 416 15.713 0.344 114.141 1.00 0.00

ATOM 6603 H GLN A 416 14.517 1.316 115.111 1.00 0.00

ATOM 6604 H GLN A 416 16.111 -0.618 116.591 1.00 0.00

ATOM 6605 H GLN A 416 14.936 -1.312 115.387 1.00 0.00

ATOM 6606 H GLN A 416 13.124 -1.267 118.552 1.00 0.00

ATOM 6607 H GLN A 416 14.453 -2.130 117.732 1.00 0.00

ATOM 6608 N VAL A 417 16.077 3.845 114.718 1.00 57.21

ATOM 6609 CA VAL A 417 16.246 4.947 113.795 1.00 55.92

ATOM 6610 C VAL A 417 17.740 5.213 113.389 1.00 53.99

ATOM 6611 O VAL A 417 18.621 5.025 114.174 1.00 51.26

ATOM 6612 CB VAL A 417 15.638 6.234 114.435 1.00 55.93

ATOM 6613 CG1 VAL A 417 15.897 7.438 113.546 1.00 55.01

ATOM 6614 CG2 VAL A 417 14.112 6.107 114.688 1.00 56.83

ATOM 6615 H VAL A 417 15.434 3.943 115.517 1.00 0.00

ATOM 6616 H VAL A 417 15.728 4.676 112.875 1.00 0.00

ATOM 6617 H VAL A 417 16.128 6.366 115.400 1.00 0.00

ATOM 6618 H VAL A 417 15.452 8.301 114.041 1.00 0.00

ATOM 6619 H VAL A 417 16.978 7.537 113.451 1.00 0.00

ATOM 6620 H VAL A 417 15.423 7.233 112.586 1.00 0.00

ATOM 6621 H VAL A 417 13.783 7.047 115.132 1.00 0.00

ATOM 6622 H VAL A 417 13.642 5.930 113.721 1.00 0.00

ATOM 6623 H VAL A 417 13.970 5.266 115.367 1.00 0.00

ATOM 6624 N SER A 418 18.053 5.714 112.204 1.00 49.21

ATOM 6625 CA SER A 418 19.482 5.783 111.890 1.00 51.44

ATOM 6626 C SER A 418 20.180 6.919 112.648 1.00 51.97

ATOM 6627 O SER A 418 19.533 7.867 113.143 1.00 52.00

ATOM 6628 CB SER A 418 19.741 5.898 110.387 1.00 51.57

ATOM 6629 OG SER A 418 19.135 7.075 109.897 1.00 52.15

ATOM 6630 H SER A 418 17.333 6.036 111.541 1.00 0.00

ATOM 6631 H SER A 418 19.913 4.840 112.226 1.00 0.00

ATOM 6632 H SER A 418 19.370 5.040 109.826 1.00 0.00

ATOM 6633 H SER A 418 20.811 6.007 110.212 1.00 0.00

ATOM 6634 H SER A 418 19.331 7.116 108.887 1.00 0.00

ATOM 6635 N THR A 419 21.497 6.860 112.726 1.00 52.27

ATOM 6636 CA THR A 419 22.160 7.832 113.539 1.00 52.68

ATOM 6637 C THR A 419 22.050 9.268 112.999 1.00 52.10

ATOM 6638 O THR A 419 21.766 10.183 113.785 1.00 52.92

ATOM 6639 CB THR A 419 23.605 7.482 113.732 1.00 54.23

ATOM 6640 OG1 THR A 419 23.666 6.304 114.529 1.00 55.28

ATOM 6641 CG2 THR A 419 24.317 8.640 114.417 1.00 52.27

ATOM 6642 H THR A 419 22.029 6.138 112.219 1.00 0.00

ATOM 6643 H THR A 419 21.641 7.807 114.497 1.00 0.00

ATOM 6644 H THR A 419 24.097 7.301 112.776 1.00 0.00

ATOM 6645 H THR A 419 23.169 5.564 114.012 1.00 0.00

ATOM 6646 H THR A 419 25.360 8.346 114.538 1.00 0.00

ATOM 6647 H THR A 419 24.213 9.508 113.766 1.00 0.00

ATOM 6648 H THR A 419 23.825 8.793 115.378 1.00 0.00

ATOM 6649 N PRO A 420 22.277 9.474 111.681 1.00 51.76

ATOM 6650 CA PRO A 420 22.065 10.849 111.149 1.00 50.88

ATOM 6651 C PRO A 420 20.634 11.466 111.397 1.00 50.09

ATOM 6652 O PRO A 420 20.568 12.661 111.672 1.00 50.60

ATOM 6653 CB PRO A 420 22.447 10.764 109.663 1.00 49.71

ATOM 6654 CG PRO A 420 22.859 9.269 109.381 1.00 52.87

ATOM 6655 CD PRO A 420 22.646 8.466 110.643 1.00 52.80

ATOM 6656 H PRO A 420 22.687 11.558 111.695 1.00 0.00

ATOM 6657 H PRO A 420 23.277 11.439 109.456 1.00 0.00

ATOM 6658 H PRO A 420 21.617 11.081 109.032 1.00 0.00

ATOM 6659 H PRO A 420 23.899 9.191 109.063 1.00 0.00

ATOM 6660 H PRO A 420 22.225 8.851 108.599 1.00 0.00

ATOM 6661 H PRO A 420 21.863 7.718 110.520 1.00 0.00

ATOM 6662 H PRO A 420 23.533 7.899 110.926 1.00 0.00

ATOM 6663 N THR A 421 19.557 10.666 111.407 1.00 49.78

ATOM 6664 CA THR A 421 18.171 11.144 111.653 1.00 48.85

ATOM 6665 C THR A 421 17.944 11.506 113.104 1.00 48.97

ATOM 6666 O THR A 421 17.398 12.550 113.400 1.00 48.51

ATOM 6667 CB THR A 421 17.088 10.068 111.316 1.00 48.86

ATOM 6668 OG1 THR A 421 17.071 9.830 109.922 1.00 49.73

ATOM 6669 CG2 THR A 421 15.687 10.536 111.681 1.00 46.81

ATOM 6670 H THR A 421 19.699 9.661 111.234 1.00 0.00

ATOM 6671 H THR A 421 18.070 12.012 111.002 1.00 0.00

ATOM 6672 H THR A 421 17.347 9.176 111.887 1.00 0.00

ATOM 6673 H THR A 421 18.015 9.512 109.659 1.00 0.00

ATOM 6674 H THR A 421 15.002 9.731 111.414 1.00 0.00

ATOM 6675 H THR A 421 15.685 10.731 112.753 1.00 0.00

ATOM 6676 H THR A 421 15.493 11.441 111.105 1.00 0.00

ATOM 6677 N LEU A 422 18.318 10.619 114.017 1.00 50.25

ATOM 6678 CA LEU A 422 18.368 10.939 115.458 1.00 50.48

ATOM 6679 C LEU A 422 19.173 12.237 115.806 1.00 50.74

ATOM 6680 O LEU A 422 18.679 13.068 116.564 1.00 49.12

ATOM 6681 CB LEU A 422 18.866 9.723 116.276 1.00 52.76

ATOM 6682 CG LEU A 422 17.835 8.605 116.551 1.00 53.57

ATOM 6683 CD1 LEU A 422 18.432 7.238 117.005 1.00 52.27

ATOM 6684 CD2 LEU A 422 16.707 9.094 117.513 1.00 49.77

ATOM 6685 H LEU A 422 18.583 9.672 113.710 1.00 0.00

ATOM 6686 H LEU A 422 17.341 11.162 115.746 1.00 0.00

ATOM 6687 H LEU A 422 19.155 10.113 117.252 1.00 0.00

ATOM 6688 H LEU A 422 19.655 9.263 115.681 1.00 0.00

ATOM 6689 H LEU A 422 17.397 8.391 115.576 1.00 0.00

ATOM 6690 H LEU A 422 17.593 6.559 117.158 1.00 0.00

ATOM 6691 H LEU A 422 19.089 6.896 116.205 1.00 0.00

ATOM 6692 H LEU A 422 18.980 7.421 117.929 1.00 0.00

ATOM 6693 H LEU A 422 16.025 8.256 117.657 1.00 0.00

ATOM 6694 H LEU A 422 17.189 9.389 118.445 1.00 0.00

ATOM 6695 H LEU A 422 16.216 9.937 117.026 1.00 0.00

ATOM 6696 N VAL A 423 20.396 12.401 115.287 1.00 50.99

ATOM 6697 CA VAL A 423 21.089 13.695 115.430 1.00 51.52

ATOM 6698 C VAL A 423 20.223 14.905 114.902 1.00 53.05

ATOM 6699 O VAL A 423 19.778 15.774 115.698 1.00 53.78

ATOM 6700 CB VAL A 423 22.569 13.717 114.799 1.00 53.39

ATOM 6701 CG1 VAL A 423 23.222 15.053 115.007 1.00 49.69

ATOM 6702 CG2 VAL A 423 23.493 12.610 115.377 1.00 48.78

ATOM 6703 H VAL A 423 20.853 11.624 114.788 1.00 0.00

ATOM 6704 H VAL A 423 21.218 13.822 116.505 1.00 0.00

ATOM 6705 H VAL A 423 22.439 13.524 113.734 1.00 0.00

ATOM 6706 H VAL A 423 24.214 14.996 114.558 1.00 0.00

ATOM 6707 H VAL A 423 22.595 15.795 114.512 1.00 0.00

ATOM 6708 H VAL A 423 23.273 15.219 116.083 1.00 0.00

ATOM 6709 H VAL A 423 24.461 12.714 114.886 1.00 0.00

ATOM 6710 H VAL A 423 23.563 12.782 116.451 1.00 0.00

ATOM 6711 H VAL A 423 23.024 11.653 115.149 1.00 0.00

ATOM 6712 N GLU A 424 19.944 14.960 113.599 1.00 52.49

ATOM 6713 CA GLU A 424 19.213 16.101 113.048 1.00 53.50

ATOM 6714 C GLU A 424 17.903 16.419 113.808 1.00 53.62

ATOM 6715 O GLU A 424 17.595 17.593 114.078 1.00 53.49

ATOM 6716 CB GLU A 424 19.039 15.950 111.523 1.00 54.18

ATOM 6717 CG GLU A 424 17.764 16.493 110.877 1.00 55.58

ATOM 6718 CD GLU A 424 17.874 16.685 109.347 1.00 59.60

ATOM 6719 OE1 GLU A 424 18.525 15.857 108.577 1.00 61.03

ATOM 6720 OE2 GLU A 424 17.267 17.687 108.904 1.00 57.49

ATOM 6721 H GLU A 424 20.245 14.195 112.978 1.00 0.00

ATOM 6722 H GLU A 424 19.821 16.991 113.208 1.00 0.00

ATOM 6723 H GLU A 424 19.018 14.876 111.336 1.00 0.00

ATOM 6724 H GLU A 424 19.845 16.538 111.085 1.00 0.00

ATOM 6725 H GLU A 424 17.579 17.474 111.315 1.00 0.00

ATOM 6726 H GLU A 424 16.970 15.771 111.067 1.00 0.00

ATOM 6727 N VAL A 425 17.142 15.399 114.186 1.00 52.93

ATOM 6728 CA VAL A 425 15.858 15.674 114.845 1.00 52.24

ATOM 6729 C VAL A 425 15.959 16.274 116.235 1.00 52.18

ATOM 6730 O VAL A 425 15.112 17.128 116.658 1.00 51.70

ATOM 6731 CB VAL A 425 15.059 14.427 114.972 1.00 53.41

ATOM 6732 CG1 VAL A 425 13.728 14.712 115.734 1.00 52.52

ATOM 6733 CG2 VAL A 425 14.802 13.911 113.582 1.00 55.57

ATOM 6734 H VAL A 425 17.447 14.429 114.020 1.00 0.00

ATOM 6735 H VAL A 425 15.386 16.413 114.197 1.00 0.00

ATOM 6736 H VAL A 425 15.598 13.675 115.549 1.00 0.00

ATOM 6737 H VAL A 425 13.188 13.767 115.796 1.00 0.00

ATOM 6738 H VAL A 425 14.000 15.089 116.720 1.00 0.00

ATOM 6739 H VAL A 425 13.181 15.454 115.153 1.00 0.00

ATOM 6740 H VAL A 425 14.216 12.998 113.684 1.00 0.00

ATOM 6741 H VAL A 425 14.252 14.688 113.050 1.00 0.00

ATOM 6742 H VAL A 425 15.774 13.720 113.128 1.00 0.00

ATOM 6743 N SER A 426 16.976 15.807 116.962 1.00 52.35

ATOM 6744 CA SER A 426 17.202 16.197 118.346 1.00 52.48

ATOM 6745 C SER A 426 17.772 17.649 118.396 1.00 52.42

ATOM 6746 O SER A 426 17.511 18.444 119.314 1.00 53.90

ATOM 6747 CB SER A 426 18.212 15.239 118.918 1.00 54.86

ATOM 6748 OG SER A 426 17.693 13.942 119.202 1.00 55.65

ATOM 6749 H SER A 426 17.629 15.142 116.525 1.00 0.00

ATOM 6750 H SER A 426 16.272 16.170 118.914 1.00 0.00

ATOM 6751 H SER A 426 18.603 15.639 119.854 1.00 0.00

ATOM 6752 H SER A 426 18.968 15.076 118.150 1.00 0.00

ATOM 6753 H SER A 426 18.470 13.384 119.584 1.00 0.00

ATOM 6754 N ARG A 427 18.578 17.973 117.406 1.00 50.77

ATOM 6755 CA ARG A 427 19.142 19.301 117.260 1.00 50.83

ATOM 6756 C ARG A 427 17.992 20.340 117.110 1.00 51.07

ATOM 6757 O ARG A 427 17.858 21.258 117.928 1.00 51.39

ATOM 6758 CB ARG A 427 20.141 19.295 116.105 1.00 48.65

ATOM 6759 CG ARG A 427 21.402 18.562 116.535 1.00 50.27

ATOM 6760 CD ARG A 427 22.698 18.709 115.651 1.00 50.45

ATOM 6761 NE ARG A 427 22.837 20.051 115.133 1.00 49.20

ATOM 6762 CZ ARG A 427 23.561 21.035 115.630 1.00 52.06

ATOM 6763 NH1 ARG A 427 24.344 20.833 116.680 1.00 60.41

ATOM 6764 NH2 ARG A 427 23.567 22.217 115.021 1.00 61.42

ATOM 6765 H ARG A 427 18.817 17.255 116.708 1.00 0.00

ATOM 6766 H ARG A 427 19.701 19.599 118.147 1.00 0.00

ATOM 6767 H ARG A 427 20.391 20.320 115.829 1.00 0.00

ATOM 6768 H ARG A 427 19.702 18.787 115.246 1.00 0.00

ATOM 6769 H ARG A 427 21.125 17.510 116.459 1.00 0.00

ATOM 6770 H ARG A 427 21.645 18.968 117.517 1.00 0.00

ATOM 6771 H ARG A 427 22.702 18.017 114.809 1.00 0.00

ATOM 6772 H ARG A 427 23.567 18.562 116.293 1.00 0.00

ATOM 6773 H ARG A 427 22.309 20.264 114.275 1.00 0.00

ATOM 6774 H ARG A 427 24.905 21.608 117.060 1.00 0.00

ATOM 6775 H ARG A 427 24.391 19.901 117.116 1.00 0.00

ATOM 6776 H ARG A 427 24.130 22.990 115.403 1.00 0.00

ATOM 6777 H ARG A 427 23.009 22.359 114.167 1.00 0.00

ATOM 6778 N SER A 428 17.169 20.116 116.082 1.00 49.98

ATOM 6779 CA SER A 428 15.935 20.778 115.817 1.00 50.70

ATOM 6780 C SER A 428 14.986 20.720 116.994 1.00 52.12

ATOM 6781 O SER A 428 14.487 21.731 117.397 1.00 53.73

ATOM 6782 CB SER A 428 15.281 20.129 114.608 1.00 50.15

ATOM 6783 OG SER A 428 16.146 20.183 113.478 1.00 48.49

ATOM 6784 H SER A 428 17.456 19.389 115.411 1.00 0.00

ATOM 6785 H SER A 428 16.152 21.829 115.627 1.00 0.00

ATOM 6786 H SER A 428 14.340 20.615 114.352 1.00 0.00

ATOM 6787 H SER A 428 15.123 19.072 114.821 1.00 0.00

ATOM 6788 H SER A 428 15.650 19.731 112.697 1.00 0.00

ATOM 6789 N LEU A 429 14.752 19.577 117.607 1.00 53.10

ATOM 6790 CA LEU A 429 13.911 19.664 118.811 1.00 54.75

ATOM 6791 C LEU A 429 14.452 20.633 119.855 1.00 56.15

ATOM 6792 O LEU A 429 13.655 21.359 120.517 1.00 57.51

ATOM 6793 CB LEU A 429 13.691 18.315 119.467 1.00 55.16

ATOM 6794 CG LEU A 429 12.869 17.297 118.693 1.00 57.09

ATOM 6795 CD1 LEU A 429 12.955 15.952 119.432 1.00 54.57

ATOM 6796 CD2 LEU A 429 11.382 17.768 118.492 1.00 57.86

ATOM 6797 H LEU A 429 15.132 18.683 117.266 1.00 0.00

ATOM 6798 H LEU A 429 12.958 20.046 118.446 1.00 0.00

ATOM 6799 H LEU A 429 13.131 18.518 120.380 1.00 0.00

ATOM 6800 H LEU A 429 14.682 17.875 119.582 1.00 0.00

ATOM 6801 H LEU A 429 13.276 17.187 117.688 1.00 0.00

ATOM 6802 H LEU A 429 12.361 15.237 118.863 1.00 0.00

ATOM 6803 H LEU A 429 14.008 15.671 119.461 1.00 0.00

ATOM 6804 H LEU A 429 12.550 16.111 120.431 1.00 0.00

ATOM 6805 H LEU A 429 10.872 16.984 117.933 1.00 0.00

ATOM 6806 H LEU A 429 10.953 17.902 119.485 1.00 0.00

ATOM 6807 H LEU A 429 11.417 18.705 117.936 1.00 0.00

ATOM 6808 N GLY A 430 15.778 20.633 120.023 1.00 55.72

ATOM 6809 CA GLY A 430 16.445 21.484 121.043 1.00 56.51

ATOM 6810 C GLY A 430 16.363 22.982 120.741 1.00 56.57

ATOM 6811 O GLY A 430 16.221 23.802 121.648 1.00 58.14

ATOM 6812 H GLY A 430 16.356 20.024 119.427 1.00 0.00

ATOM 6813 H GLY A 430 17.499 21.206 121.030 1.00 0.00

ATOM 6814 H GLY A 430 15.955 21.293 121.998 1.00 0.00

ATOM 6815 N LYS A 431 16.447 23.372 119.471 1.00 55.03

ATOM 6816 CA LYS A 431 16.126 24.765 119.109 1.00 55.26

ATOM 6817 C LYS A 431 14.679 25.206 119.449 1.00 56.18

ATOM 6818 O LYS A 431 14.430 26.413 119.653 1.00 59.39

ATOM 6819 CB LYS A 431 16.509 25.080 117.690 1.00 54.79

ATOM 6820 CG LYS A 431 17.990 24.880 117.406 1.00 56.26

ATOM 6821 CD LYS A 431 18.420 25.021 115.937 1.00 60.23

ATOM 6822 CE LYS A 431 20.021 25.177 115.830 1.00 67.48

ATOM 6823 NZ LYS A 431 20.822 24.164 114.906 1.00 67.26

ATOM 6824 H LYS A 431 16.735 22.703 118.743 1.00 0.00

ATOM 6825 H LYS A 431 16.751 25.377 119.759 1.00 0.00

ATOM 6826 H LYS A 431 16.282 26.133 117.524 1.00 0.00

ATOM 6827 H LYS A 431 15.947 24.409 117.041 1.00 0.00

ATOM 6828 H LYS A 431 18.212 23.852 117.692 1.00 0.00

ATOM 6829 H LYS A 431 18.516 25.656 117.963 1.00 0.00

ATOM 6830 H LYS A 431 17.951 25.926 115.552 1.00 0.00

ATOM 6831 H LYS A 431 18.076 24.148 115.383 1.00 0.00

ATOM 6832 H LYS A 431 20.404 25.007 116.836 1.00 0.00

ATOM 6833 H LYS A 431 20.182 26.152 115.369 1.00 0.00

ATOM 6834 H LYS A 431 21.817 24.422 114.968 1.00 0.00

ATOM 6835 H LYS A 431 20.648 23.218 115.275 1.00 0.00

ATOM 6836 H LYS A 431 20.456 24.275 113.950 1.00 0.00

ATOM 6837 N VAL A 432 13.738 24.270 119.604 1.00 54.63

ATOM 6838 CA VAL A 432 12.394 24.619 120.143 1.00 55.40

ATOM 6839 C VAL A 432 12.434 25.266 121.564 1.00 57.53

ATOM 6840 O VAL A 432 11.492 25.950 121.969 1.00 58.27

ATOM 6841 CB VAL A 432 11.423 23.434 120.100 1.00 53.40

ATOM 6842 CG1 VAL A 432 10.076 23.825 120.538 1.00 53.80

ATOM 6843 CG2 VAL A 432 11.324 22.915 118.700 1.00 55.19

ATOM 6844 H VAL A 432 13.945 23.294 119.347 1.00 0.00

ATOM 6845 H VAL A 432 12.013 25.387 119.470 1.00 0.00

ATOM 6846 H VAL A 432 11.809 22.669 120.773 1.00 0.00

ATOM 6847 H VAL A 432 9.451 22.934 120.479 1.00 0.00

ATOM 6848 H VAL A 432 10.167 24.189 121.561 1.00 0.00

ATOM 6849 H VAL A 432 9.734 24.606 119.859 1.00 0.00

ATOM 6850 H VAL A 432 10.626 22.078 118.719 1.00 0.00

ATOM 6851 H VAL A 432 10.957 23.734 118.082 1.00 0.00

ATOM 6852 H VAL A 432 12.325 22.601 118.405 1.00 0.00

ATOM 6853 N GLY A 433 13.551 25.091 122.266 1.00 58.50

ATOM 6854 CA GLY A 433 13.778 25.690 123.552 1.00 60.65

ATOM 6855 C GLY A 433 13.901 27.180 123.402 1.00 63.44

ATOM 6856 O GLY A 433 13.148 27.949 124.048 1.00 64.63

ATOM 6857 H GLY A 433 14.291 24.496 121.867 1.00 0.00

ATOM 6858 H GLY A 433 14.714 25.289 123.941 1.00 0.00

ATOM 6859 H GLY A 433 12.959 25.427 124.221 1.00 0.00

ATOM 6860 N THR A 434 14.836 27.567 122.532 1.00 63.68

ATOM 6861 CA THR A 434 15.163 28.947 122.164 1.00 65.97

ATOM 6862 C THR A 434 13.970 29.602 121.448 1.00 66.79

ATOM 6863 O THR A 434 13.742 30.808 121.547 1.00 69.60

ATOM 6864 CB THR A 434 16.381 28.957 121.155 1.00 66.49

ATOM 6865 OG1 THR A 434 17.371 27.983 121.529 1.00 65.84

ATOM 6866 CG2 THR A 434 17.049 30.345 120.991 1.00 66.50

ATOM 6867 H THR A 434 15.381 26.823 122.074 1.00 0.00

ATOM 6868 H THR A 434 15.408 29.492 123.076 1.00 0.00

ATOM 6869 H THR A 434 15.954 28.697 120.186 1.00 0.00

ATOM 6870 H THR A 434 16.884 27.081 121.626 1.00 0.00

ATOM 6871 H THR A 434 17.865 30.224 120.279 1.00 0.00

ATOM 6872 H THR A 434 16.283 31.025 120.618 1.00 0.00

ATOM 6873 H THR A 434 17.411 30.643 121.975 1.00 0.00

ATOM 6874 N ARG A 435 13.224 28.818 120.691 1.00 65.45

ATOM 6875 CA ARG A 435 12.065 29.350 119.990 1.00 66.46

ATOM 6876 C ARG A 435 10.993 29.651 120.996 1.00 67.30

ATOM 6877 O ARG A 435 10.429 30.724 120.957 1.00 70.37

ATOM 6878 CB ARG A 435 11.510 28.357 118.936 1.00 64.73

ATOM 6879 CG ARG A 435 12.354 28.214 117.632 1.00 67.49

ATOM 6880 CD ARG A 435 11.979 26.927 116.751 1.00 69.82

ATOM 6881 NE ARG A 435 12.880 26.713 115.595 1.00 69.79

ATOM 6882 CZ ARG A 435 12.967 27.543 114.547 1.00 71.54

ATOM 6883 NH1 ARG A 435 12.218 28.662 114.504 1.00 69.84

ATOM 6884 NH2 ARG A 435 13.813 27.274 113.556 1.00 70.00

ATOM 6885 H ARG A 435 13.465 27.821 120.596 1.00 0.00

ATOM 6886 H ARG A 435 12.374 30.252 119.461 1.00 0.00

ATOM 6887 H ARG A 435 10.538 28.746 118.634 1.00 0.00

ATOM 6888 H ARG A 435 11.499 27.376 119.411 1.00 0.00

ATOM 6889 H ARG A 435 13.389 28.095 117.953 1.00 0.00

ATOM 6890 H ARG A 435 12.176 29.107 117.032 1.00 0.00

ATOM 6891 H ARG A 435 10.965 27.008 116.358 1.00 0.00

ATOM 6892 H ARG A 435 12.138 26.050 117.379 1.00 0.00

ATOM 6893 H ARG A 435 13.477 25.874 115.597 1.00 0.00

ATOM 6894 H ARG A 435 12.288 29.298 113.697 1.00 0.00

ATOM 6895 H ARG A 435 11.576 28.882 115.279 1.00 0.00

ATOM 6896 H ARG A 435 13.881 27.911 112.750 1.00 0.00

ATOM 6897 H ARG A 435 14.400 26.429 113.595 1.00 0.00

ATOM 6898 N CYS A 436 10.710 28.737 121.915 1.00 66.09

ATOM 6899 CA CYS A 436 9.459 28.862 122.676 1.00 67.06

ATOM 6900 C CYS A 436 9.513 29.136 124.166 1.00 69.39

ATOM 6901 O CYS A 436 8.484 29.481 124.740 1.00 71.52

ATOM 6902 CB CYS A 436 8.587 27.631 122.487 1.00 65.64

ATOM 6903 SG CYS A 436 8.234 27.268 120.773 1.00 65.31

ATOM 6904 H CYS A 436 11.355 27.954 122.092 1.00 0.00

ATOM 6905 H CYS A 436 9.058 29.777 122.240 1.00 0.00

ATOM 6906 H CYS A 436 7.650 27.961 122.936 1.00 0.00

ATOM 6907 H CYS A 436 9.067 26.821 123.035 1.00 0.00

ATOM 6908 N CYS A 437 10.665 28.974 124.807 1.00 69.41

ATOM 6909 CA CYS A 437 10.678 28.989 126.249 1.00 71.07

ATOM 6910 C CYS A 437 10.896 30.332 126.812 1.00 73.83

ATOM 6911 O CYS A 437 10.721 30.499 128.003 1.00 76.60

ATOM 6912 CB CYS A 437 11.706 28.018 126.814 1.00 71.19

ATOM 6913 SG CYS A 437 11.297 26.295 126.463 1.00 70.95

ATOM 6914 H CYS A 437 11.540 28.839 124.280 1.00 0.00

ATOM 6915 H CYS A 437 9.682 28.666 126.551 1.00 0.00

ATOM 6916 H CYS A 437 11.576 28.164 127.886 1.00 0.00

ATOM 6917 H CYS A 437 12.683 28.349 126.463 1.00 0.00

ATOM 6918 N THR A 438 11.292 31.301 125.981 1.00 74.24

ATOM 6919 CA THR A 438 11.369 32.715 126.452 1.00 76.55

ATOM 6920 C THR A 438 10.105 33.518 126.252 1.00 75.93

ATOM 6921 O THR A 438 9.984 34.592 126.825 1.00 80.10

ATOM 6922 CB THR A 438 12.467 33.539 125.798 1.00 77.04

ATOM 6923 OG1 THR A 438 13.339 32.680 125.088 1.00 78.44

ATOM 6924 CG2 THR A 438 13.238 34.281 126.859 1.00 82.62

ATOM 6925 H THR A 438 11.546 31.070 125.010 1.00 0.00

ATOM 6926 H THR A 438 11.570 32.563 127.513 1.00 0.00

ATOM 6927 H THR A 438 12.024 34.256 125.107 1.00 0.00

ATOM 6928 H THR A 438 12.772 32.196 124.377 1.00 0.00

ATOM 6929 H THR A 438 14.011 34.856 126.348 1.00 0.00

ATOM 6930 H THR A 438 12.528 34.927 127.376 1.00 0.00

ATOM 6931 H THR A 438 13.664 33.532 127.526 1.00 0.00

ATOM 6932 N LYS A 439 9.226 33.038 125.386 1.00 72.14

ATOM 6933 CA LYS A 439 7.858 33.533 125.222 1.00 72.32

ATOM 6934 C LYS A 439 7.035 33.573 126.496 1.00 74.01

ATOM 6935 O LYS A 439 7.278 32.784 127.396 1.00 73.62

ATOM 6936 CB LYS A 439 7.120 32.663 124.199 1.00 68.95

ATOM 6937 CG LYS A 439 7.622 32.933 122.810 1.00 66.62

ATOM 6938 CD LYS A 439 6.553 32.743 121.791 1.00 66.27

ATOM 6939 CE LYS A 439 7.038 33.147 120.434 1.00 67.58

ATOM 6940 NZ LYS A 439 8.248 32.368 120.139 1.00 67.84

ATOM 6941 H LYS A 439 9.527 32.259 124.783 1.00 0.00

ATOM 6942 H LYS A 439 7.963 34.565 124.889 1.00 0.00

ATOM 6943 H LYS A 439 6.052 32.880 124.235 1.00 0.00

ATOM 6944 H LYS A 439 7.309 31.614 124.428 1.00 0.00

ATOM 6945 H LYS A 439 8.433 32.239 122.590 1.00 0.00

ATOM 6946 H LYS A 439 7.954 33.970 122.761 1.00 0.00

ATOM 6947 H LYS A 439 5.700 33.366 122.060 1.00 0.00

ATOM 6948 H LYS A 439 6.270 31.691 121.769 1.00 0.00

ATOM 6949 H LYS A 439 7.257 34.214 120.398 1.00 0.00

ATOM 6950 H LYS A 439 6.277 32.938 119.683 1.00 0.00

ATOM 6951 H LYS A 439 8.571 32.655 119.204 1.00 0.00

ATOM 6952 H LYS A 439 8.938 32.596 120.869 1.00 0.00

ATOM 6953 H LYS A 439 7.979 31.374 120.163 1.00 0.00

ATOM 6954 N PRO A 440 6.043 34.489 126.567 1.00 76.51

ATOM 6955 CA PRO A 440 5.071 34.541 127.659 1.00 78.76

ATOM 6956 C PRO A 440 4.570 33.171 128.033 1.00 77.01

ATOM 6957 O PRO A 440 4.473 32.317 127.175 1.00 75.02

ATOM 6958 CB PRO A 440 3.937 35.379 127.055 1.00 79.98

ATOM 6959 CG PRO A 440 4.660 36.430 126.298 1.00 80.23

ATOM 6960 CD PRO A 440 5.890 35.673 125.694 1.00 78.63

ATOM 6961 H PRO A 440 5.491 34.952 128.577 1.00 0.00

ATOM 6962 H PRO A 440 3.248 35.791 127.793 1.00 0.00

ATOM 6963 H PRO A 440 3.285 34.797 126.404 1.00 0.00

ATOM 6964 H PRO A 440 4.958 37.240 126.963 1.00 0.00

ATOM 6965 H PRO A 440 4.025 36.921 125.561 1.00 0.00

ATOM 6966 H PRO A 440 5.717 35.361 124.664 1.00 0.00

ATOM 6967 H PRO A 440 6.803 36.265 125.639 1.00 0.00

ATOM 6968 N GLU A 441 4.263 32.953 129.302 1.00 79.57

ATOM 6969 CA GLU A 441 3.823 31.646 129.747 1.00 79.14

ATOM 6970 C GLU A 441 2.648 31.087 128.958 1.00 77.51

ATOM 6971 O GLU A 441 2.716 29.957 128.458 1.00 75.25

ATOM 6972 CB GLU A 441 3.540 31.611 131.242 1.00 82.01

ATOM 6973 CG GLU A 441 4.777 31.322 132.076 1.00 85.85

ATOM 6974 CD GLU A 441 5.494 32.595 132.584 1.00 91.28

ATOM 6975 OE1 GLU A 441 5.344 32.916 133.793 1.00 93.82

ATOM 6976 OE2 GLU A 441 6.206 33.260 131.779 1.00 91.18

ATOM 6977 H GLU A 441 4.338 33.723 129.982 1.00 0.00

ATOM 6978 H GLU A 441 4.667 30.986 129.545 1.00 0.00

ATOM 6979 H GLU A 441 2.822 30.812 131.426 1.00 0.00

ATOM 6980 H GLU A 441 3.169 32.594 131.532 1.00 0.00

ATOM 6981 H GLU A 441 5.474 30.791 131.428 1.00 0.00

ATOM 6982 H GLU A 441 4.458 30.743 132.942 1.00 0.00

ATOM 6983 N SER A 442 1.577 31.840 128.806 1.00 79.27

ATOM 6984 CA SER A 442 0.392 31.194 128.225 1.00 79.29

ATOM 6985 C SER A 442 0.517 30.985 126.702 1.00 76.39

ATOM 6986 O SER A 442 -0.405 30.505 126.036 1.00 76.06

ATOM 6987 CB SER A 442 -0.889 31.937 128.596 1.00 82.01

ATOM 6988 OG SER A 442 -1.052 33.045 127.754 1.00 82.51

ATOM 6989 H SER A 442 1.571 32.833 129.081 1.00 0.00

ATOM 6990 H SER A 442 0.331 30.198 128.664 1.00 0.00

ATOM 6991 H SER A 442 -0.901 32.271 129.633 1.00 0.00

ATOM 6992 H SER A 442 -1.745 31.289 128.409 1.00 0.00

ATOM 6993 H SER A 442 -1.924 33.514 128.039 1.00 0.00

ATOM 6994 N GLU A 443 1.681 31.339 126.183 1.00 74.97

ATOM 6995 CA GLU A 443 1.971 31.280 124.782 1.00 73.29

ATOM 6996 C GLU A 443 2.999 30.166 124.447 1.00 70.03

ATOM 6997 O GLU A 443 3.127 29.739 123.267 1.00 67.49

ATOM 6998 CB GLU A 443 2.453 32.672 124.317 1.00 75.11

ATOM 6999 CG GLU A 443 2.882 32.711 122.845 1.00 76.84

ATOM 7000 CD GLU A 443 3.380 34.076 122.331 1.00 83.93

ATOM 7001 OE1 GLU A 443 4.097 34.007 121.293 1.00 85.44

ATOM 7002 OE2 GLU A 443 3.078 35.177 122.907 1.00 84.86

ATOM 7003 H GLU A 443 2.417 31.675 126.820 1.00 0.00

ATOM 7004 H GLU A 443 1.063 31.016 124.239 1.00 0.00

ATOM 7005 H GLU A 443 3.321 32.942 124.918 1.00 0.00

ATOM 7006 H GLU A 443 1.616 33.362 124.427 1.00 0.00

ATOM 7007 H GLU A 443 1.994 32.466 122.262 1.00 0.00

ATOM 7008 H GLU A 443 3.717 32.017 122.748 1.00 0.00

ATOM 7009 N ARG A 444 3.715 29.702 125.480 1.00 69.53

ATOM 7010 CA ARG A 444 4.794 28.720 125.326 1.00 66.85

ATOM 7011 C ARG A 444 4.232 27.418 124.732 1.00 65.75

ATOM 7012 O ARG A 444 4.780 26.843 123.796 1.00 62.72

ATOM 7013 CB ARG A 444 5.518 28.477 126.671 1.00 68.05

ATOM 7014 CG ARG A 444 6.760 29.370 126.888 1.00 70.39

ATOM 7015 CD ARG A 444 7.595 29.165 128.186 1.00 71.78

ATOM 7016 NE ARG A 444 7.957 30.474 128.749 1.00 76.46

ATOM 7017 CZ ARG A 444 8.237 30.727 130.045 1.00 83.30

ATOM 7018 NH1 ARG A 444 8.277 29.743 130.940 1.00 82.84

ATOM 7019 NH2 ARG A 444 8.494 31.981 130.461 1.00 83.62

ATOM 7020 H ARG A 444 3.498 30.050 126.425 1.00 0.00

ATOM 7021 H ARG A 444 5.539 29.114 124.634 1.00 0.00

ATOM 7022 H ARG A 444 5.860 27.442 126.672 1.00 0.00

ATOM 7023 H ARG A 444 4.810 28.712 127.466 1.00 0.00

ATOM 7024 H ARG A 444 6.369 30.385 126.955 1.00 0.00

ATOM 7025 H ARG A 444 7.433 29.131 126.065 1.00 0.00

ATOM 7026 H ARG A 444 8.501 28.590 127.998 1.00 0.00

ATOM 7027 H ARG A 444 6.986 28.652 128.930 1.00 0.00

ATOM 7028 H ARG A 444 8.001 31.269 128.096 1.00 0.00

ATOM 7029 H ARG A 444 8.492 29.952 131.925 1.00 0.00

ATOM 7030 H ARG A 444 8.093 28.773 130.647 1.00 0.00

ATOM 7031 H ARG A 444 8.706 32.161 131.453 1.00 0.00

ATOM 7032 H ARG A 444 8.478 32.760 129.788 1.00 0.00

ATOM 7033 N MET A 445 3.109 26.945 125.235 1.00 67.26

ATOM 7034 CA MET A 445 2.559 25.710 124.712 1.00 65.93

ATOM 7035 C MET A 445 2.107 25.718 123.275 1.00 63.04

ATOM 7036 O MET A 445 2.485 24.858 122.538 1.00 61.69

ATOM 7037 CB MET A 445 1.415 25.252 125.583 1.00 68.83

ATOM 7038 CG MET A 445 0.938 23.871 125.302 1.00 71.06

ATOM 7039 SD MET A 445 -0.446 23.856 124.184 1.00 77.20

ATOM 7040 CE MET A 445 -1.671 24.729 125.110 1.00 75.68

ATOM 7041 H MET A 445 2.627 27.449 125.993 1.00 0.00

ATOM 7042 H MET A 445 3.403 25.021 124.732 1.00 0.00

ATOM 7043 H MET A 445 0.575 25.922 125.402 1.00 0.00

ATOM 7044 H MET A 445 1.779 25.249 126.610 1.00 0.00

ATOM 7045 H MET A 445 0.564 23.569 126.280 1.00 0.00

ATOM 7046 H MET A 445 1.806 23.287 124.997 1.00 0.00

ATOM 7047 H MET A 445 -2.569 24.769 124.493 1.00 0.00

ATOM 7048 H MET A 445 -1.836 24.167 126.029 1.00 0.00

ATOM 7049 H MET A 445 -1.271 25.723 125.309 1.00 0.00

ATOM 7050 N PRO A 446 1.354 26.714 122.852 1.00 63.71

ATOM 7051 CA PRO A 446 1.013 26.854 121.415 1.00 62.11

ATOM 7052 C PRO A 446 2.244 26.910 120.524 1.00 60.55

ATOM 7053 O PRO A 446 2.338 26.191 119.534 1.00 59.93

ATOM 7054 CB PRO A 446 0.233 28.174 121.372 1.00 63.47

ATOM 7055 CG PRO A 446 -0.499 28.159 122.762 1.00 66.53

ATOM 7056 CD PRO A 446 0.581 27.663 123.700 1.00 65.97

ATOM 7057 H PRO A 446 0.449 26.001 121.038 1.00 0.00

ATOM 7058 H PRO A 446 -0.449 28.229 120.523 1.00 0.00

ATOM 7059 H PRO A 446 0.865 29.046 121.206 1.00 0.00

ATOM 7060 H PRO A 446 -1.400 27.547 122.790 1.00 0.00

ATOM 7061 H PRO A 446 -0.868 29.141 123.059 1.00 0.00

ATOM 7062 H PRO A 446 1.205 28.468 124.088 1.00 0.00

ATOM 7063 H PRO A 446 0.189 27.197 124.604 1.00 0.00

ATOM 7064 N CYS A 447 3.206 27.734 120.889 1.00 61.57

ATOM 7065 CA CYS A 447 4.462 27.785 120.181 1.00 61.50

ATOM 7066 C CYS A 447 5.028 26.394 120.037 1.00 59.28

ATOM 7067 O CYS A 447 5.302 25.965 118.899 1.00 58.33

ATOM 7068 CB CYS A 447 5.460 28.656 120.934 1.00 62.69

ATOM 7069 SG CYS A 447 7.041 28.816 120.122 1.00 71.02

ATOM 7070 H CYS A 447 3.058 28.355 121.697 1.00 0.00

ATOM 7071 H CYS A 447 4.285 28.213 119.194 1.00 0.00

ATOM 7072 H CYS A 447 5.632 28.055 121.827 1.00 0.00

ATOM 7073 H CYS A 447 4.957 29.603 121.127 1.00 0.00

ATOM 7074 N THR A 448 5.232 25.698 121.170 1.00 59.38

ATOM 7075 CA THR A 448 5.800 24.331 121.175 1.00 59.21

ATOM 7076 C THR A 448 5.028 23.385 120.296 1.00 58.12

ATOM 7077 O THR A 448 5.610 22.740 119.407 1.00 58.15

ATOM 7078 CB THR A 448 5.996 23.696 122.621 1.00 60.75

ATOM 7079 OG1 THR A 448 7.236 24.129 123.186 1.00 63.25

ATOM 7080 CG2 THR A 448 6.078 22.186 122.563 1.00 60.86

ATOM 7081 H THR A 448 4.983 26.133 122.070 1.00 0.00

ATOM 7082 H THR A 448 6.799 24.466 120.760 1.00 0.00

ATOM 7083 H THR A 448 5.138 24.014 123.214 1.00 0.00

ATOM 7084 H THR A 448 7.202 25.157 123.235 1.00 0.00

ATOM 7085 H THR A 448 6.211 21.835 123.586 1.00 0.00

ATOM 7086 H THR A 448 5.142 21.832 122.131 1.00 0.00

ATOM 7087 H THR A 448 6.933 21.937 121.935 1.00 0.00

ATOM 7088 N GLU A 449 3.723 23.302 120.494 1.00 59.52

ATOM 7089 CA GLU A 449 2.934 22.356 119.696 1.00 59.15

ATOM 7090 C GLU A 449 3.176 22.537 118.194 1.00 58.02

ATOM 7091 O GLU A 449 3.505 21.593 117.469 1.00 57.20

ATOM 7092 CB GLU A 449 1.443 22.411 120.073 1.00 61.38

ATOM 7093 CG GLU A 449 1.015 21.275 121.006 1.00 62.11

ATOM 7094 CD GLU A 449 1.318 19.842 120.463 1.00 66.35

ATOM 7095 OE1 GLU A 449 0.972 18.900 121.198 1.00 64.95

ATOM 7096 OE2 GLU A 449 1.850 19.616 119.305 1.00 64.81

ATOM 7097 H GLU A 449 3.265 23.895 121.201 1.00 0.00

ATOM 7098 H GLU A 449 3.278 21.350 119.937 1.00 0.00

ATOM 7099 H GLU A 449 0.862 22.321 119.155 1.00 0.00

ATOM 7100 H GLU A 449 1.270 23.349 120.600 1.00 0.00

ATOM 7101 H GLU A 449 -0.067 21.356 121.106 1.00 0.00

ATOM 7102 H GLU A 449 1.566 21.403 121.938 1.00 0.00

ATOM 7103 N ASP A 450 3.094 23.778 117.769 1.00 58.56

ATOM 7104 CA ASP A 450 3.244 24.173 116.381 1.00 59.08

ATOM 7105 C ASP A 450 4.530 23.684 115.654 1.00 56.82

ATOM 7106 O ASP A 450 4.380 23.101 114.570 1.00 57.23

ATOM 7107 CB ASP A 450 2.947 25.697 116.279 1.00 61.37

ATOM 7108 CG ASP A 450 3.690 26.386 115.158 1.00 65.40

ATOM 7109 OD1 ASP A 450 3.737 25.825 114.039 1.00 67.67

ATOM 7110 OD2 ASP A 450 4.197 27.529 115.383 1.00 70.30

ATOM 7111 H ASP A 450 2.912 24.516 118.464 1.00 0.00

ATOM 7112 H ASP A 450 2.506 23.628 115.793 1.00 0.00

ATOM 7113 H ASP A 450 3.249 26.174 117.211 1.00 0.00

ATOM 7114 H ASP A 450 1.886 25.808 116.054 1.00 0.00

ATOM 7115 N TYR A 451 5.735 23.878 116.227 1.00 55.98

ATOM 7116 CA TYR A 451 7.035 23.520 115.589 1.00 55.25

ATOM 7117 C TYR A 451 7.375 22.023 115.731 1.00 54.39

ATOM 7118 O TYR A 451 8.159 21.468 114.947 1.00 54.58

ATOM 7119 CB TYR A 451 8.223 24.273 116.211 1.00 55.49

ATOM 7120 CG TYR A 451 8.464 25.727 115.810 1.00 61.47

ATOM 7121 CD1 TYR A 451 8.103 26.777 116.676 1.00 68.80

ATOM 7122 CD2 TYR A 451 9.069 26.070 114.598 1.00 62.35

ATOM 7123 CE1 TYR A 451 8.318 28.123 116.330 1.00 70.48

ATOM 7124 CE2 TYR A 451 9.292 27.383 114.244 1.00 63.57

ATOM 7125 CZ TYR A 451 8.921 28.429 115.123 1.00 71.40

ATOM 7126 OH TYR A 451 9.114 29.801 114.800 1.00 75.25

ATOM 7127 H TYR A 451 5.759 24.301 117.166 1.00 0.00

ATOM 7128 H TYR A 451 6.897 23.793 114.543 1.00 0.00

ATOM 7129 H TYR A 451 9.111 23.737 115.876 1.00 0.00

ATOM 7130 H TYR A 451 8.005 24.313 117.278 1.00 0.00

ATOM 7131 H TYR A 451 7.646 26.541 117.637 1.00 0.00

ATOM 7132 H TYR A 451 9.373 25.278 113.914 1.00 0.00

ATOM 7133 H TYR A 451 8.012 28.920 117.008 1.00 0.00

ATOM 7134 H TYR A 451 9.755 27.617 113.285 1.00 0.00

ATOM 7135 H TYR A 451 8.740 30.351 115.586 1.00 0.00

ATOM 7136 N LEU A 452 6.864 21.391 116.774 1.00 54.04

ATOM 7137 CA LEU A 452 7.094 19.999 117.001 1.00 53.71

ATOM 7138 C LEU A 452 6.496 19.240 115.882 1.00 53.96

ATOM 7139 O LEU A 452 7.015 18.199 115.452 1.00 55.38

ATOM 7140 CB LEU A 452 6.453 19.544 118.325 1.00 55.20

ATOM 7141 CG LEU A 452 7.379 19.896 119.481 1.00 55.18

ATOM 7142 CD1 LEU A 452 6.871 19.265 120.772 1.00 59.78

ATOM 7143 CD2 LEU A 452 8.806 19.472 119.159 1.00 49.40

ATOM 7144 H LEU A 452 6.283 21.917 117.442 1.00 0.00

ATOM 7145 H LEU A 452 8.167 19.818 117.062 1.00 0.00

ATOM 7146 H LEU A 452 6.286 18.467 118.307 1.00 0.00

ATOM 7147 H LEU A 452 5.500 20.055 118.463 1.00 0.00

ATOM 7148 H LEU A 452 7.384 20.976 119.626 1.00 0.00

ATOM 7149 H LEU A 452 7.568 19.548 121.561 1.00 0.00

ATOM 7150 H LEU A 452 5.873 19.664 120.951 1.00 0.00

ATOM 7151 H LEU A 452 6.854 18.187 120.614 1.00 0.00

ATOM 7152 H LEU A 452 9.421 19.747 120.016 1.00 0.00

ATOM 7153 H LEU A 452 8.790 18.394 119.001 1.00 0.00

ATOM 7154 H LEU A 452 9.103 20.009 118.258 1.00 0.00

ATOM 7155 N SER A 453 5.415 19.755 115.363 1.00 54.45

ATOM 7156 CA SER A 453 4.767 19.029 114.340 1.00 54.70

ATOM 7157 C SER A 453 5.493 19.182 113.019 1.00 54.17

ATOM 7158 O SER A 453 5.367 18.344 112.149 1.00 55.39

ATOM 7159 CB SER A 453 3.301 19.417 114.272 1.00 56.47

ATOM 7160 OG SER A 453 3.135 20.513 113.429 1.00 57.86

ATOM 7161 H SER A 453 5.047 20.660 115.689 1.00 0.00

ATOM 7162 H SER A 453 4.801 17.965 114.575 1.00 0.00

ATOM 7163 H SER A 453 2.882 19.656 115.249 1.00 0.00

ATOM 7164 H SER A 453 2.740 18.603 113.813 1.00 0.00

ATOM 7165 H SER A 453 2.129 20.735 113.418 1.00 0.00

ATOM 7166 N LEU A 454 6.246 20.255 112.838 1.00 53.78

ATOM 7167 CA LEU A 454 7.145 20.332 111.686 1.00 52.12

ATOM 7168 C LEU A 454 8.294 19.364 111.868 1.00 50.09

ATOM 7169 O LEU A 454 8.902 18.884 110.874 1.00 47.91

ATOM 7170 CB LEU A 454 7.750 21.736 111.571 1.00 52.42

ATOM 7171 CG LEU A 454 6.687 22.834 111.561 1.00 54.74

ATOM 7172 CD1 LEU A 454 7.249 24.226 111.843 1.00 43.41

ATOM 7173 CD2 LEU A 454 5.774 22.743 110.282 1.00 52.79

ATOM 7174 H LEU A 454 6.199 21.036 113.508 1.00 0.00

ATOM 7175 H LEU A 454 6.567 20.092 110.794 1.00 0.00

ATOM 7176 H LEU A 454 8.299 21.792 110.631 1.00 0.00

ATOM 7177 H LEU A 454 8.389 21.898 112.439 1.00 0.00
[truncated: 691,968 more chars]
